# A Chemically Defined Four‐Component Self‐Adjuvanting Tn Vaccine Activating Mincle, FcγR, and CD206 for Enhanced Antitumor Immunity

**DOI:** 10.1002/advs.76235

**Published:** 2026-06-22

**Authors:** Wenbo Ming, Renyu Zhang, Xiaohui Li, Guiqi Li, Yu Niu, Deying Yang, Xiang Luo, Jun Liao, Zhongqiu Liu, Guochao Liao

**Affiliations:** ^1^ Guangdong Provincial Key Laboratory of Translational Chinese Medicine Joint International Research Laboratory of Translational Cancer Research of Chinese Medicines International Institute for Translational Chinese Medicine School of Pharmaceutical Sciences Guangzhou University of Chinese Medicine Guangzhou China; ^2^ Chinese Medicine Guangdong Laboratory Hengqin China; ^3^ Affiliated Hospital of Guangdong Medical University Zhanjiang China; ^4^ Department of Biochemistry and Molecular Biology College of Basic Medical Sciences Second Military Medical University Shanghai China

**Keywords:** antitumor agents, carbohydrates, immunological activity, TACA vaccines, total synthesis

## Abstract

Tumor‐associated carbohydrate antigens (TACAs), such as the Tn antigen, are promising targets for cancer vaccines but are limited by their low immunogenicity and a lack of T‐cell responses. Traditional carrier‐protein conjugates often encounter issues such as epitope suppression and heterogeneous formulations. Herein, we present the design, synthesis, and immunological evaluation of a chemically defined, four‐component self‐adjuvanting glycoconjugate. This molecule combines multiple innate immune activation strategies into a single, unimolecular framework, incorporating (1) a synthetic Tn as the B‐cell epitope; (2) vizantin, a strong Mincle agonist, as an internal adjuvant; (3) rhamnose to attract endogenous antibodies for Fcγ receptor–mediated uptake; and (4) mannose to target dendritic cells through CD206. By ensuring precise co‐delivery and synergistic activation of Mincle, Fcγ receptor, and CD206 pathways, this construct induces strong IgG switching, Th1‑type cytokine responses, and significant tumor suppression in mice. This research offers a versatile chemical approach for next‑generation TACA vaccines, illustrating how activating multiple innate pathways can overcome the longstanding limitations of carbohydrate‐based immunotherapies.

## Graphical abstract

A fully synthetic four‐component vaccine has been developed combining Tn antigen, vizantin (Mincle agonist), rhamnose (antibody recruitment), and mannose (CD206 targeting). By synergistically activating Mincle, FcγR, and CD206 pathways, it elicits strong IgG switching and cytotoxic T‐cells, significantly inhibiting tumors in mice. This approach offers a potent strategy for overcoming the longstanding limitations of carbohydrate‐based immunotherapies.

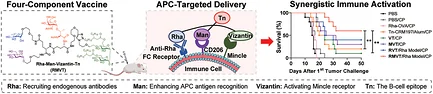

## 1 Introduction

Therapeutic cancer vaccines are a promising type of immunotherapy, capable of generating long‐lasting, antigen‐specific immune responses that target cancer cells while sparing healthy tissues [1, 2, 3]. Carbohydrate antigens like Tn (GalNAcαOSer/Thr), sTn, TF, and Globo H are among the various tumor‐associated targets. They are often overexpressed in carcinomas and have limited expression in normal tissues, which makes them appealing candidates for vaccine development [4, 5, 6, 7]. However, these tumor‐associated carbohydrate antigens (TACAs) are inherently poorly immunogenic because they do not depend on T cells for recognition. They usually trigger only short‐lived IgM responses and lack effective class switching to high‐affinity IgG subclasses. Overcoming these issues remains a key obstacle to developing carbohydrate‐based cancer vaccines [8, 9, 10].

Traditional approaches often involve attaching synthetic glycans to immunogenic protein carriers like keyhole limpet hemocyanin (KLH), CRM197, or tetanus toxoid, and then formulating these conjugates with strong external adjuvants such as QS‐21, monophosphoryl lipid A (MPLA), or CpG oligonucleotides [8, 11, 12, 13]. For example, clinical trials using a Tn cluster‐KLH conjugate vaccine plus QS‐21 adjuvant in patients with prostate cancer indicated its potential for inducing antibody production [14, 15]. A prominent example is the STn‐KLH vaccine (Theratope) [16], which progressed to Phase III clinical trials but ultimately failed to demonstrate significant clinical benefit in patients with metastatic breast cancer. Similar outcomes were observed for the Globo‐H‐KLH vaccine (GLORIA) in Phase III trials for high‐risk triple‐negative breast cancer [17]. These failures underscore a major limitation of conventional carrier–adjuvant approaches: the mere induction of a humoral immune response is often insufficient to achieve a therapeutic effect against solid tumors. There is an urgent need for developing chemically defined multi‐component vaccine platforms that can precisely orchestrate both innate and adaptive immunity [18, 19].

Self‐adjuvanting vaccine constructs overcome many of these issues by covalently combining the antigenic epitope with an immunostimulatory component into a single, chemically precise molecule [20, 21]. This design guarantees the precise co‐delivery of antigen and adjuvant to the same APC, enhancing strong innate immune activation via specific pattern recognition receptors (PRRs) [22, 23]. Self‐adjuvanting vaccines remove the need for separate carrier proteins. From a synthetic perspective, these vaccines use orthogonal ligation techniques—like amide bond formation, click chemistry, and selective O‐glycosylation—to attach each functional part while maintaining biological activity [7, 24]. Early self‐adjuvanting carbohydrate vaccines were usually simple, involving just two components: the antigen and an inherent adjuvant (like a TLR2 ligand Pam3Cys [25, 26], TLR4 ligand MPLA [27, 28], Mincle agonist vizantin/TDB [29, 30, 31], or α‐GalCer for invariant natural killer T (iNKT) activation) [32, 33]. In our previous work, we constructed a dual‐agonist self‐adjuvanting vaccine, MPLA–Tn–KRN7000. Combined with low‐dose cyclophosphamide, it achieved a 50‐day survival rate of 62.5% (mean 41.6 days) in tumor‐bearing mice, outperforming the conventional Tn–CRM197 vaccine (12.5%) and demonstrating potent antitumor efficacy [28].

Recently, the addition of extra targeting modules designed to improve APC uptake and antigen processing has led to multi‐component vaccine designs with enhanced efficacy. Targeting modules contain rhamnose (Rha) residues that attract naturally occurring anti‐Rha antibodies and enhance Fcγ receptor (FcγR)‐mediated responses internalization [34, 35, 36, 37, 38, 39, 40, 41, 42], and mannose (Man) residues that bind to the Man receptor (CD206), initiating receptor‐mediated endocytosis by dendritic cells [22, 43, 44, 45].

Combining these elements, such as constructs integrating PRR agonists with iNKT cell ligands [28, 46] or multivalent antigens [23, 47, 48, 49], demonstrates the power of synergistic activation. Despite these advances, a vaccine construct that combines a TACA B‐cell epitope with three immunomodulatory modules—namely, a built‐in adjuvant, a CD206‐targeting ligand, and an antibody‐recruiting glycan—within a single, chemically defined molecule remains unavailable.

**FIGURE 1:**
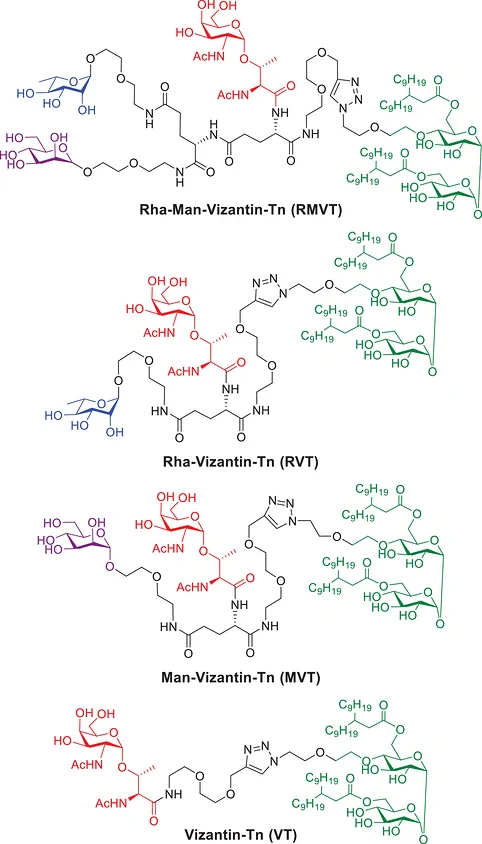
Structural designs of the four‐, three‐, and two‐component vaccines.

This paper details the design, synthesis, and immunological testing of a four‐component self‐adjuvanting carbohydrate cancer vaccine (Figure 1). The construct combines a synthetic Tn antigen for tumor targeting [50], vizantin as a powerful Mincle receptor agonist adjuvant, Rha to enable Fcγ receptor‐mediated targeting through the recruitment of endogenous antibodies, and Man to enhance dendritic cell uptake via CD206 engagement. This precisely defined architecture exemplifies targeted delivery, innate immune activation, and antigen‐specific immunity, providing a versatile and modular platform for next‐generation carbohydrate‐based cancer vaccines.

## 2 Results and Discussion

### 2.1 Retrosynthetic Analysis

**SCHEME 1:**
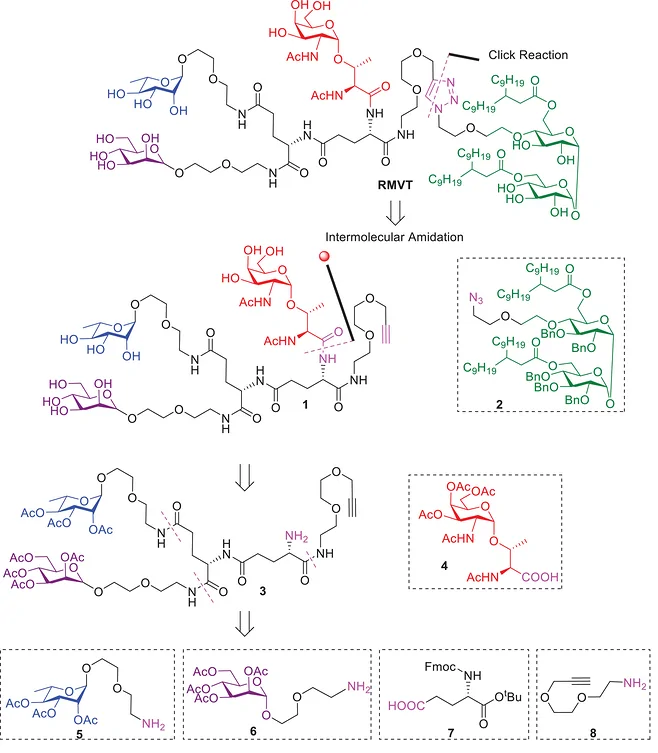
Retrosynthetic analysis of the designed vaccine Rha‐Man‐Vizantin‐Tn (**RMVT**).

The retrosynthetic analysis of the four‐component self‐adjuvanting vaccine Rha‐Man‐Vizantin‐Tn (**RMVT**) is shown in Scheme 1. The vizantin fragment, featuring a 2‐azidoethyl group (**2**), and the Rha‐Man‐Tn fragment, with a terminal alkyne group (**1**), were produced by breaking down the 1,2,3‐triazole ring. The construction of **1** involved a Tn Thr with a free carboxyl group (**4**) and a Rha‐Man moiety (**3**). Fragment **3** was broken down into a linker chain (**8**) with a propargyl and a free amino group, an L‐glutamic acid (**7**) with a protected amino group, a protected carboxyl group, and a free carboxyl group, a Man derivative (**6**), and an Rha derivative (**5**) with a free amino group. All these fragments are synthesizable from readily available starting materials such as D‐Man, L‐Rha, D‐galactosamine, and α,α'‐D‐trehalose. The choice of protecting groups, which are vital for coupling reactions, is critical during fragment preparation. The hydroxyl groups on vizantin's carbohydrate rings are protected by benzyl groups, which can be removed simultaneously after the final step.

### 2.2 Synthesis of the Designed Vaccines RMVT, RVT, MVT, VT

#### 2.2.1 Synthesis of Rha‐Man Building Block

**SCHEME 2:**
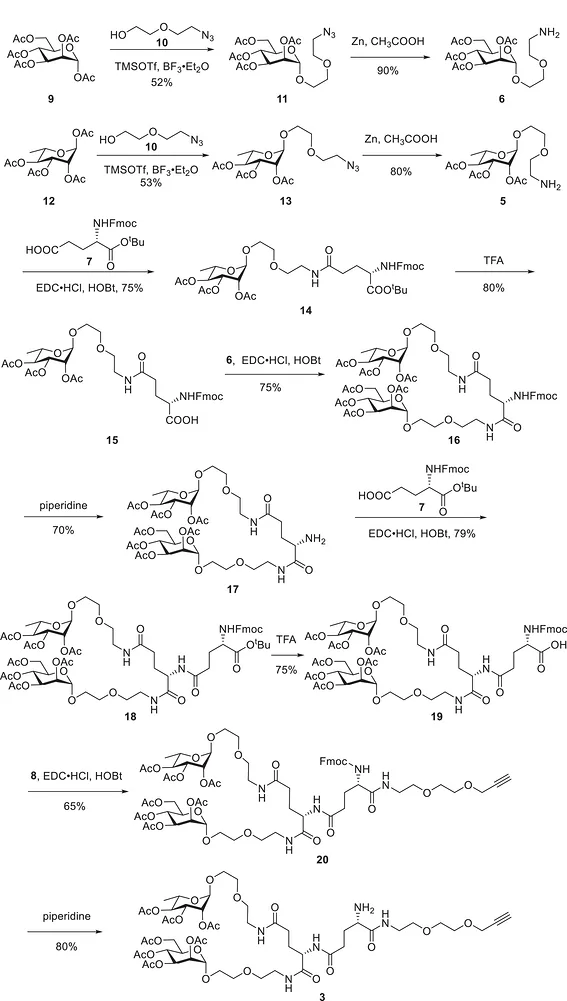
Synthesis of Rha‐Man fragment.

The synthesis of the Rha fragment (**15**) started with a properly protected L‐rhamnosamine derivative (**5**), made from acyl‐protected L‐rhamnose (Scheme 2). Acylation of compound **5** with protected L‐glutamic acid (**7**) yielded fragment **14**. Treating fragment **14** with 20% TFA produced the desired Rha fragment (**15**) with an 80% yield. The synthesis of the Man fragment (**6**) began from acyl‐protected D‐mannose **9**, prepared in two steps, and used for the next step without further purification. The free amino group in compound **6** was acetylated with the free carboxyl group in compound **15** using 1‐ethyl‐3‐(3‐dimethylaminopropyl)carbodiimide (EDC•HCl) and 1‐hydroxybenzotriazole (HOBt) to produce compound **16**. After selectively removing the Fmoc protecting group from compound **16** with piperidine, it reacted with compound **7** to produce an intermediate (**18**). The ^t^Bu group was then deprotected with TFA, yielding intermediate **19** bearing a carboxyl group. Finally, acylation with linker **8** followed by the removal of Fmoc protection produced the desired Rha‐Man fragment **3**.

#### 2.2.2 Synthesis of Rha‐Man‐Vizantin‐Tn (RMVT) Conjugate

**SCHEME 3:**
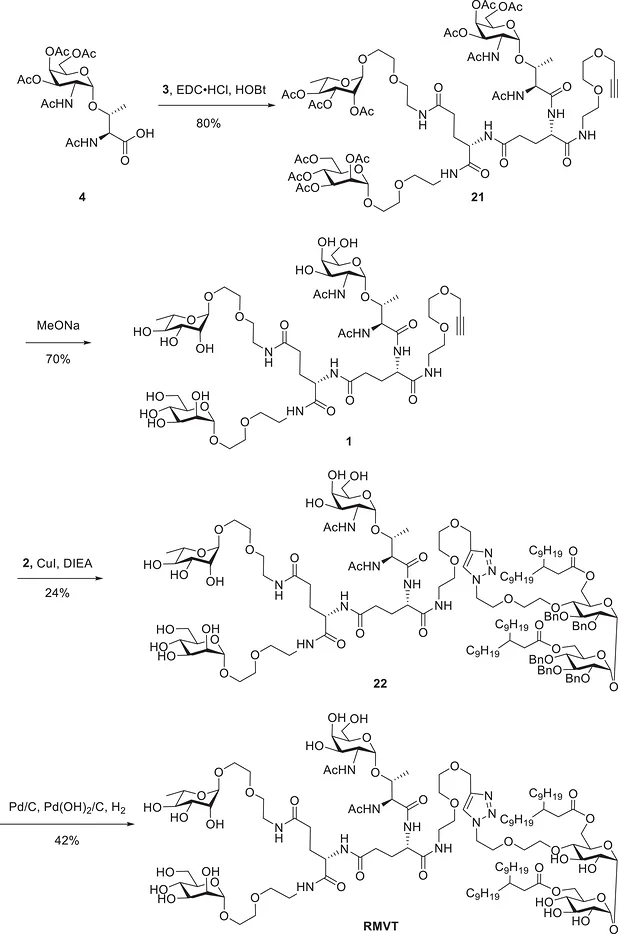
Synthesis of Rha‐Man‐Vizantin‐Tn (**RMVT**) conjugate.

The Tn fragment **4** [28] and the vizantin fragment **2** [46] were synthesized using methods similar to those described in the literature. Once the three key fragments were obtained, the **RMVT** conjugate was assembled as shown in Scheme 3. The intermediate **21**, containing fragments **3** and **4**, was synthesized with EDC•HCl and HOBt, with a yield of 80%. All acetyl groups on the methyl carboxylate in this intermediate were removed with sodium methoxide, resulting in compound **1**. This compound was then coupled with fragment **2** via a click reaction using cuprous iodide as the catalyst and DIEA as the base, forming the key intermediate **22**. Finally, all benzyl groups were removed via Pd‐catalyzed hydrogenolysis under an H_2_ atmosphere, producing the target **RMVT** conjugate with a 42% yield. The final product was characterized by NMR and HRMS.

#### 2.2.3 Synthesis of Rha‐Vizantin‐Tn (RVT) Conjugate

**SCHEME 4:**
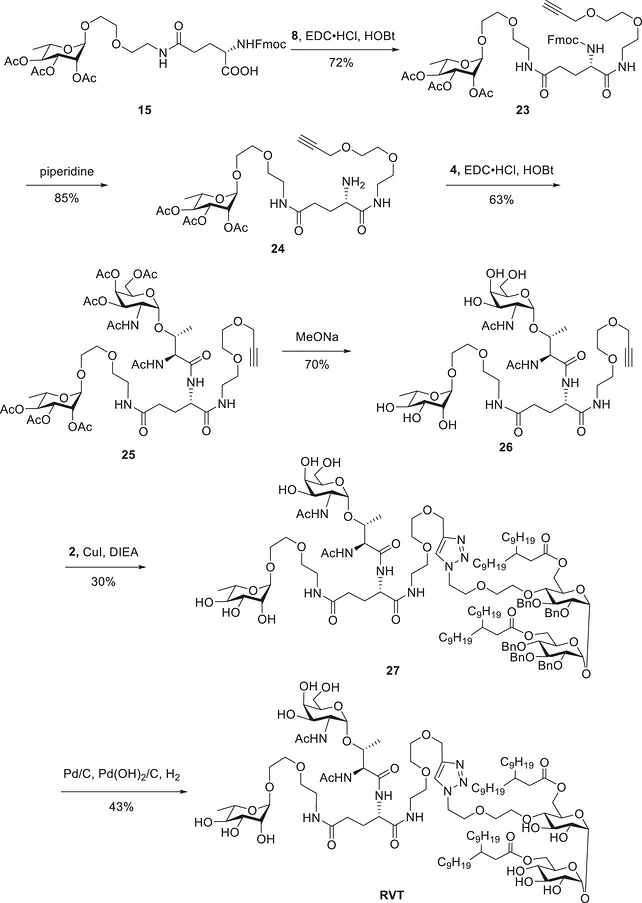
Synthesis of Rha‐Vizantin‐Tn (**RVT**) conjugate.

The synthesis of the **RVT** conjugate began directly from intermediate **15**, as illustrated in Scheme 4. A 1,2,3‐triazole linked vizantin and Rha‐Tn. Intermediate **23**, which includes fragments **15** and **8**, was obtained with a yield of 72% using EDC•HCl and HOBt. The Fmoc groups in fragment **23** were selectively deprotected with piperidine to yield Rha derivative **24**. After coupling with fragment **4**, all acetyl groups on the methyl carboxylate were deprotected using sodium methoxide, producing compound **26**. The final **RVT** conjugate was synthesized via a click reaction and hydrogenolysis, following the method described for the previous steps of **RMVT** production.

#### 2.2.4 Synthesis of Man‐Vizantin‐Tn (MVT) Conjugate

**SCHEME 5:**
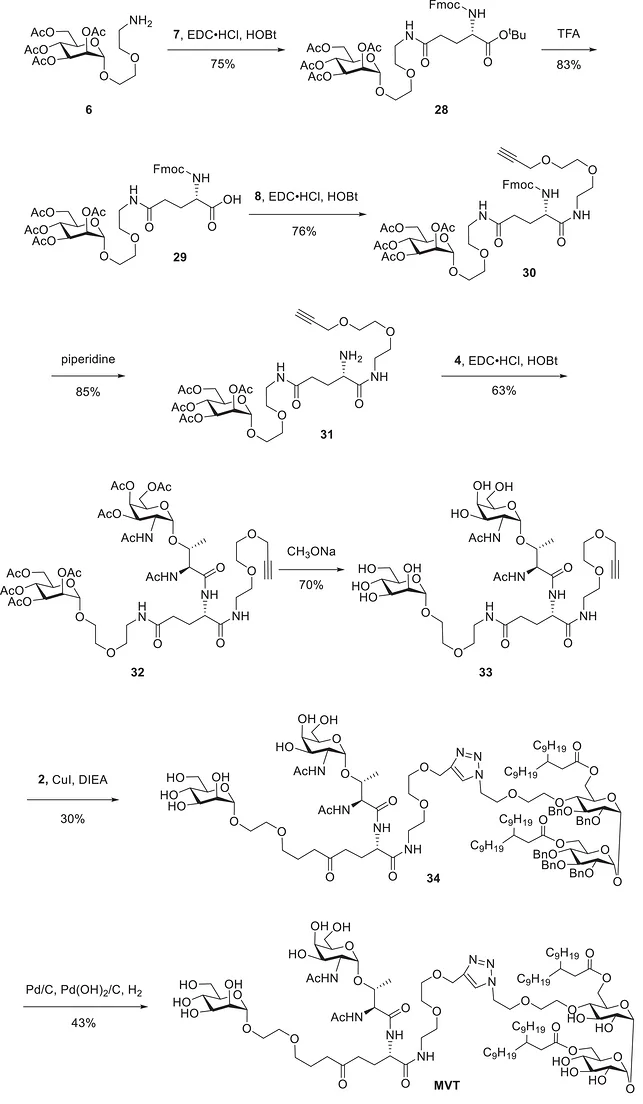
Synthesis of Man‐Vizantin‐Tn (**MVT**) conjugate.

The synthesis of the **MVT** conjugate is illustrated in Scheme 5. Initially, the free amino group in intermediate **6** was coupled with linker **7** using EDC•HCl and HOBt as catalysts, yielding intermediate **28** with a 75% yield. After removing the ^t^Bu protecting groups with TFA, the free carboxyl group in the resulting intermediate was linked to linker **8** to form compound **30**. Next, the Fmoc groups in **30** were selectively deprotected, and this product reacted with fragment **4** to produce the Rha‐Tn fragment **32**. All acetyl groups on the methyl carboxylate were then removed using sodium methylate, yielding compound **33** in a 70% yield. Finally, coupling **33** with **2** was achieved through a click reaction, followed by hydrogenolysis of **34** to obtain the final **MVT** conjugate.

#### 2.2.5 Synthesis of Vizantin‐Tn (VT) Conjugate

**SCHEME 6:**
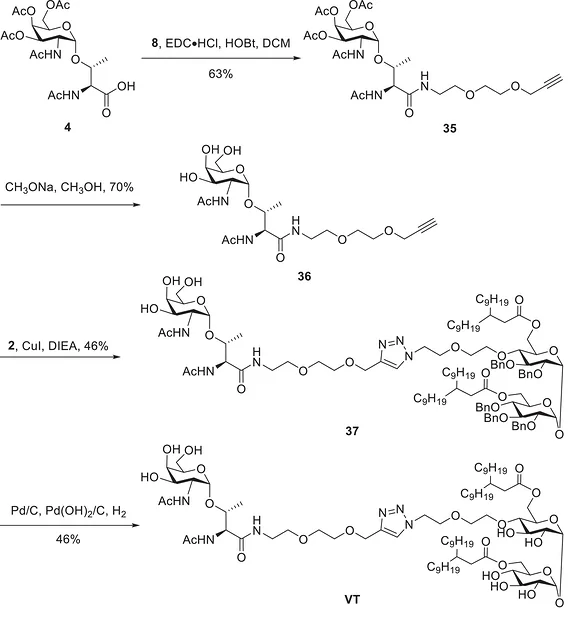
Synthesis of Vizantin‐Tn (**VT**) conjugate.

Scheme 6 illustrates the synthesis of the **VT** conjugate. Coupling of the Tn fragment **4** and the linker **8** produced an intermediate **35**, followed by the selective removal of Ac groups on the sugar ring with sodium methoxide. Intermediate **37**, which includes fragments **36** and **2**, was produced with a 46% yield using CuI and DIEA. Hydrogenolysis of compound **37** was performed to obtain the final VT conjugate **(48)**.

### 2.3 Binding Affinity of Conjugates for Human Mincle‐Fc

**FIGURE 2:**
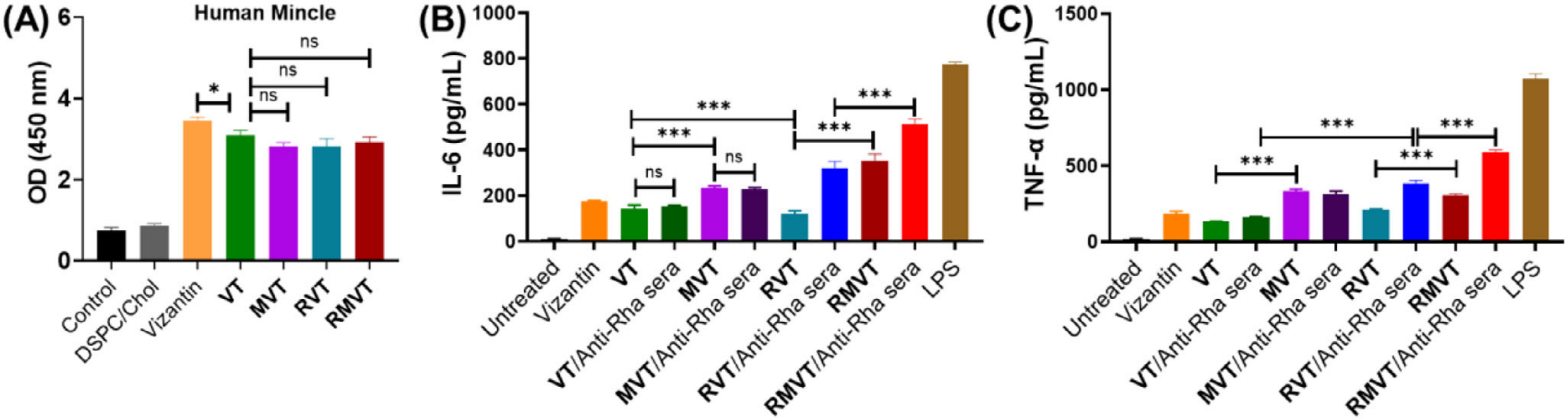
The binding affinity of target conjugates to human Mincle‐Fc (A) and their ability to stimulate BMDMs to produce inflammatory cytokines IL‐6 (B) and TNF‐α (C). BMDMs were treated with Vizantin, VT, MVT, RVT, or RMVT at 1 nmol/well, with or without anti‐rhamnose serum (1:100 dilution), and LPS was used at 100 ng/mL as a positive control. The data are shown as mean ± SD (n = 3). Significant differences between groups are indicated by brackets with asterisks, based on one‐way ANOVA and Tukey's multiple comparisons test. “*ns*” means not significant; ^***^p < 0.001, ^**^p < 0.01, ^*^p < 0.05 significant. SD, standard deviation; ANOVA, analysis of variance.

The binding affinities of **VT**, **MVT**, **RVT**, and **RMVT** for soluble human Mincle‐Fc were evaluated to determine whether these target conjugates could recognize and bind to Mincle. Because the conjugates were delivered as liposomes containing 1,2‐distearoyl‐sn‐glycero‐3‐phosphocholine (DSPC) and cholesterol (Chol), the binding capacity of the DSPC and cholesterol mixture was also assessed. DSPC/ Chol, vizantin, **VT**, **MVT**, **RVT**, and **RMVT** (50 µg/mL, 100 µL per well) were incubated with human Mincle‐Fc, and ligand binding was detected via ELISA. As shown in Figure 2A, the DSPC and cholesterol mixture alone did not exhibit significant binding to human Mincle‐Fc. In contrast to the Control and DSPC/Chol groups, vizantin and its derivatives displayed measurable binding to human Mincle‐Fc. Among them, VT showed only a marginal reduction in affinity compared to unmodified vizantin. Notably, there was no significant difference in binding affinity among **VT**, **MVT**, **RVT**, and **RMVT** for human Mincle‐Fc. These findings suggest that adding the Tn antigen, Rha, or Man to the **VT** structure does not substantially alter its binding ability to human Mincle‐Fc.

### 2.4 Abilities of Conjugates RMVT, RVT, MVT, and VT to Induce the Production of Inflammatory Cytokines

Next, the ability of the target conjugates to stimulate bone marrow‐derived macrophages (BMDMs) to produce inflammatory cytokines interleukin‐6 (IL‐6) and tumor necrosis factor‐α (TNF‐α) was assessed. This method is a standard approach for evaluating the functionality of Mincle ligands. Anti‐Rha sera collected from Balb/c mice immunized with Rha‐OVA, which contain high‐titer anti‐rhamnose antibodies. BMDMs were treated with Vizantin, VT, MVT, RVT, or RMVT at 1 nmol/well, with or without anti‐rhamnose serum (1:100 dilution), and LPS was used at 100 ng/mL as a positive control. As shown in Figure 2B,C, stimulating BMDMs with the conjugates significantly increased IL‐6 and TNF‐α production compared to the control. Cytokine levels followed a distinct pattern: **RMVT**/Anti‐Rha sera > **RMVT**, and **RVT**/Anti‐Rha sera > **RVT**. In contrast, **MVT** or **VT** induced comparable cytokine levels regardless of the presence of Anti‐Rha sera. This pattern suggests that the Rha moiety may enhance cytokine production via antibody‐mediated recruitment. Furthermore, **RMVT** consistently triggered higher cytokine levels than **RVT**, and **MVT** higher than **VT**, indicating that Man targeting alone is sufficient to amplify the inflammatory cytokine response. Overall, these findings show that the vizantin‐based conjugates trigger cytokine production in a manner dependent on Mincle, with effectiveness similar to or greater than vizantin alone. Adding Rha and Man further enhanced this activity, with **RMVT** showing the strongest effect. This finding might be attributed to a combination of Rha‐dependent antibody responses and Man directing the conjugates to antigen‐presenting cells.

### 2.5 Immunological Evaluation of VT, MVT, RVT, and RMVT in Balb/c Mice

Immunological studies on **VT**, **MVT**, **RVT**, and **RMVT** were carried out using Balb/c mice. All experiments adhered to the Guiding Principles for the Care and Use of Animals (China) and received approval from the Laboratory Animal Ethics Committee of Guangzhou University of Chinese Medicine (No. 20220426). The conjugates were delivered as liposomes, prepared by sonication of a mixture containing the conjugates, DSPC, and cholesterol in a molar ratio of 65/50/10 to enhance their solubility and immunogenicity. Tn‐CRM197 glycoprotein vaccine (using CRM197 as the carrier protein) was used as a positive control. The preparation and characterization of Tn‐CRM197 followed the protocol described previously [28] with a carbohydrate loading rate of 4.8% (Figure S1). Since the glycoprotein conjugate Tn‐CRM197 is more effective with an external adjuvant, it was administered as an emulsion with a clinically used alum adjuvant (Alum). In this study, Tn‐CRM197 was first dissolved in phosphate‐buffered saline (PBS, pH 7.4) and then thoroughly mixed with Alum before use.

**FIGURE 3:**
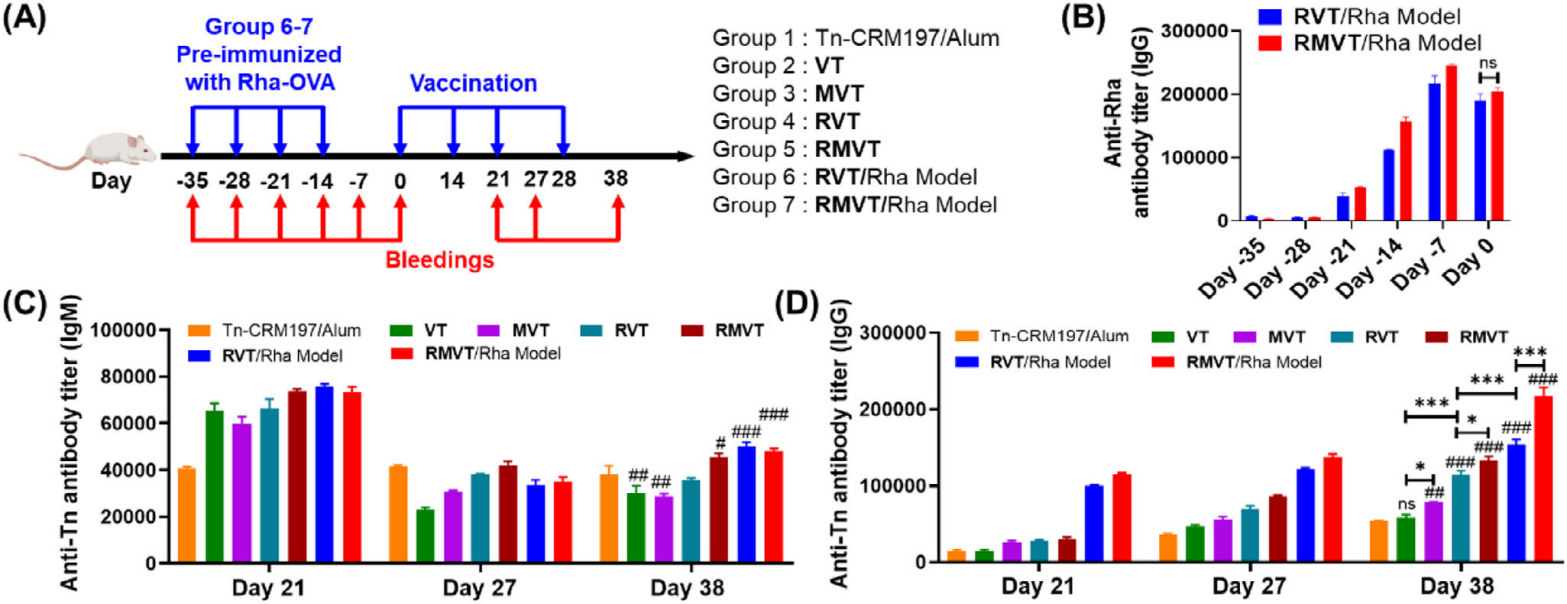
(A) Immunization schedule. (B) Anti‐Rha antibody titers in sera from mice immunized with Rha‐OVA. Tn‐specific IgM (C) and IgG (D) antibody titers in pooled sera collected on days 21, 27, and 38 from mice immunized with Tn‐CRM197/Alum, **VT**, **MVT**, **RVT**, and **RMVT**. Data are shown as mean ± SD (n = 3). Bracketed asterisks denote significant differences between groups, determined by one‐way ANOVA with Tukey's multiple comparisons test. ^***^p < 0.001, ^**^p < 0.01, ^*^p < 0.05; *ns* = not significant. Compared to Tn‐CRM197/Alum, *^###^p* < 0.001, *^##^p* < 0.01, *^#^p* < 0.05, *ns* = not significant.

For mouse immunization, **VT**, **MVT**, **RVT**, and **RMVT** were administered at a dose of 6 µg Tn per mouse, which corresponds to the optimal level established in our previous studies [28]. Since Balb/c mice do not have pre‐existing anti‐Rha antibodies, we induced a model with high anti‐Rha antibody titers (around 200 000) by pre‐immunizing the mice with Rha‐OVA, as shown in Figure 3A,B. These pre‐immunized mice were then used to assess the impact of Rha‐mediated antigen recruitment through **RVT** and **RMVT** immunizations (Groups 6 and 7). In the glycoprotein control group, the mice received a subcutaneous injection of 0.1 mL emulsion containing 1.7 µg of Tn conjugated to CRM197/Alum, a dose known to elicit robust immune responses. Booster doses were administered on days 14, 21, and 28 via subcutaneous injection of the same conjugate and protocol as on day 0. Blood was drawn from each mouse's eye socket on day 0 (before initial immunization, serving as blank controls) and on days 21, 27, and 38 post‐injections. The blood samples were processed to prepare antisera following standard protocols for ELISA of Tn‐specific antibodies, using Tn‐HSA as the capture reagent. Total antibodies (kappa, IgG, and IgM) and IgG antibody isotypes, including IgG1, IgG2a, IgG2b, and IgG3 titers, were measured. Importantly, all experimental groups exhibited steady body weight gain without any observable signs of distress, indicating normal physiological function throughout the study (Figure S6).

The IgM and IgG antibody titers in the pooled sera collected on days 21, 27, and 38 from each group of immunized mice are shown in Figure 3C. All conjugate vaccines successfully induced high Tn‐specific IgM titers, which are the primary antibody type produced by B cells in response to foreign antigens. Elevated IgG titers were also observed on day 21, with levels rising further after booster shots, indicating a strengthening of the immune response (Figure 3D). After four immunizations, IgM titers declined while IgG titers increased, demonstrating efficient class switching from IgM to IgG and the long‐term triggering of sustained secondary immune responses.

In the existing anti‐Rha antibody model, **RVT** produced higher antibody levels compared to **VT** (154 301 vs. 58 624). Similarly, **RMVT** exceeded **MVT** (217 097 vs. 78 515). This difference might be attributed to endogenous anti‐Rha antibodies recognizing the Rha residue in the vaccine construct, thereby forming antigen‐antibody complexes. These complexes are then taken up by antigen‐presenting cells through Fc receptor binding, enhancing the vaccine response.

Furthermore, even without pre‐existing antibodies, **RVT** generated higher IgG titers compared to **VT** (114 639 vs. 58 624). Similarly, **RMVT** surpassed **MVT** (133 173 vs. 78 515). Immunization of mice with the Rha‐containing vaccines (**RVT** and **RMVT**) similarly induced anti‐Rha antibody responses, whereas no corresponding anti‐Rha antibodies were detected in mice immunized with the Rha‐free vaccine (**VT**) (Figure S7). This finding indicates that the immunization itself prompted the production of anti‐Rha antibodies, which then boosted the vaccine's immunogenicity through an antigen‑recruitment mechanism. All vaccine molecules containing Mincle agonists produced significantly higher IgG antibody levels compared to the alum‐adjuvanted glycoprotein control. Notably, the four‐component vaccine **RMVT** in the pre‐existing antibody model generated the highest anti‐Tn IgG levels. Overall, these results suggest that Mincle agonists could serve as effective alternatives to traditional protein carriers and that combining Rha and Man in vaccine design can synergistically boost the immune response.

**FIGURE 4:**
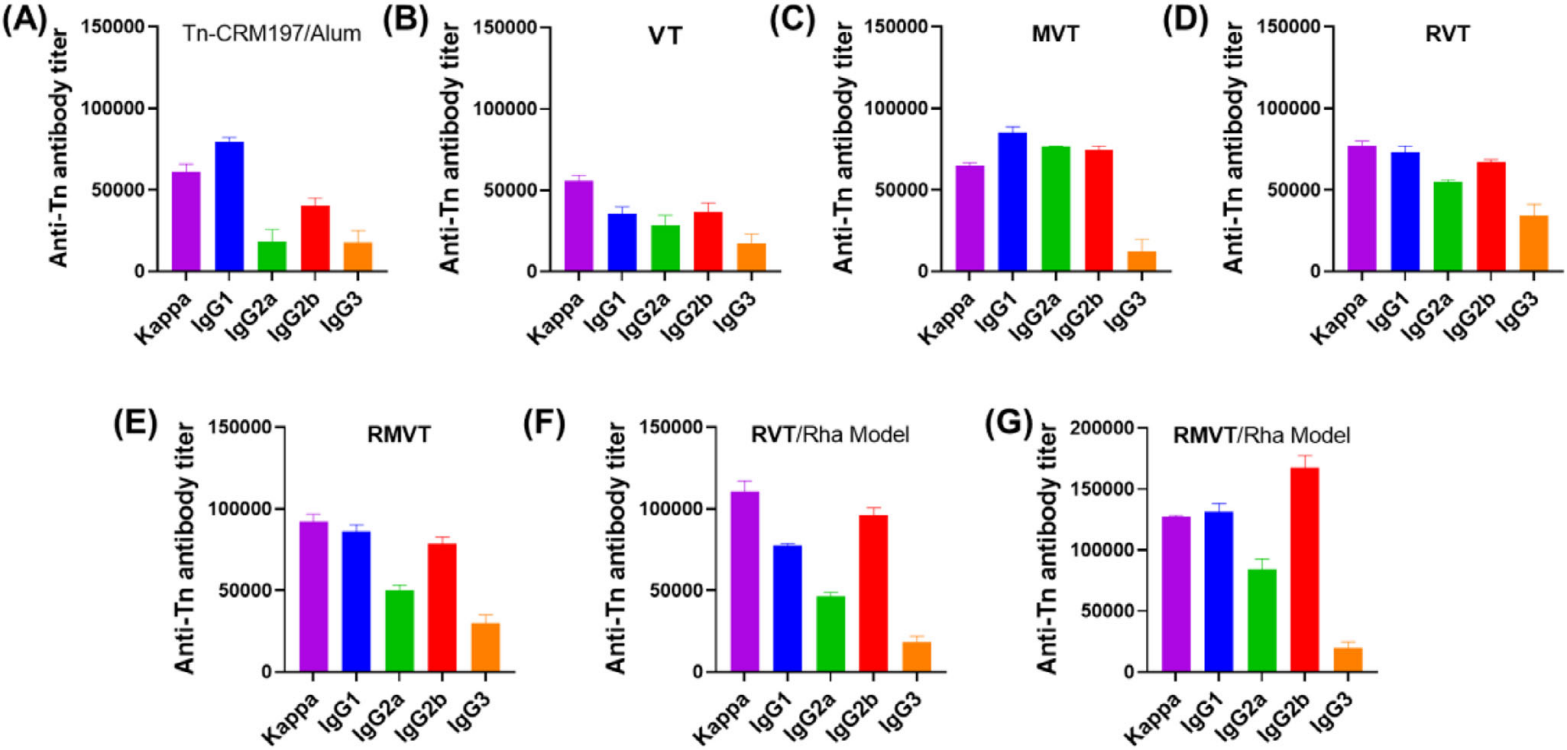
The titers of total antibody and antibody isotypes in pooled antisera collected from mice immunized with Tn‐CRM197/Alum (A), **VT** (B), **MVT** (C), **RVT** (D and F), **RMVT** (E and G). Statistical data are presented as mean ± SD (n = 3).

Figure 4A–G show the titers of kappa and IgG isotypes (IgG1, IgG2a, IgG2b, and IgG3) antibodies—induced by the vaccines in individual mice and across all experimental groups on day 38. Detection of kappa light chain titers provides a rapid and accurate measurement of total Tn‐specific antibody levels induced by the conjugates. Furthermore, the kappa antibody titers demonstrated that the **RMVT**/Rha Model exhibited the most potent immune activity. Generally, the kappa antibody titers ranked as follows: **RMVT**/Rha Model > **RVT**/Rha Model > **RMVT** > **RVT** > **MVT** > Tn‐CRM197/Alum > **VT**, which aligns well with the earlier observed IgG antibody levels. IgG3, a typical marker of T‐cell‐mediated anti‐carbohydrate immune responses, was found in all groups. The fully synthetic vaccines triggered different levels of IgG1 and IgG2a antibodies; however, the glycoprotein vaccine predominantly elicited an IgG1 response. In mice, the secretion of IgG1 and IgG2a reflects Th2 and Th1‐type immune responses. These findings demonstrate that fully synthetic vaccines elicit a mixed Th1/Th2 immune response, in sharp contrast to the strong Th2 bias observed with the traditional glycoprotein vaccine Tn‐CRM197/Alum. The Tn‐CRM197/Alum group showed a typical Th2‐dominant response, with the IgG1 titer (79 616) significantly higher than the IgG2a titer (18 119), resulting in an IgG1/IgG2a ratio of 4.39. In contrast, **VT** and its modified forms exhibited a more balanced immune profile. Man modification (**MVT**) notably increased Th1‐associated subclasses (IgG2a and IgG2b), while Rha modification (**RVT**) specifically elevated IgG3 levels. Remarkably, the four‐component vaccine **RMVT**, in the context of pre‐existing anti‐Rha antibodies, showed the strongest immunomodulatory synergy. It not only achieved the highest total IgG titers but also displayed a Th1‐biased profile, with IgG2b (167 230) and IgG2a (84 327) reaching peak levels, all while maintaining high IgG1 levels (131 254). This balanced immune phenotype is essential for triggering both effective cellular and humoral immunity. It offers an immunological basis for the superior protection provided by **RMVT** in tumor challenge studies.

Overall, the data show that fully synthetic vaccines elicit a mixed Th1/Th2 immune response, whereas the glycoprotein vaccine with alum primarily induces a Th2 response [31, 33]. A mixed Th1/Th2 response generally offers better anti‐tumor immunity than a pure Th2 response. These findings suggest that, through deliberate molecular design—integrating Mincle agonists with specific glycosylation modifications—researchers can precisely steer the immune response triggered by vaccines. This approach presents a promising strategy for developing next‐generation anticancer vaccines.

**FIGURE 5:**
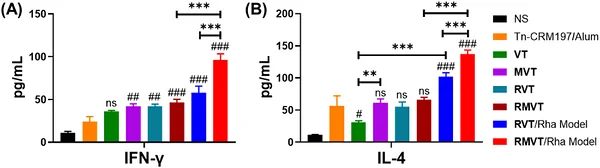
IFN‐γ (A) and IL‐4 (B) levels in pooled sera from mice immunized with Tn‐CRM197/Alum, **VT**, **MVT**, **RVT**, and **RMVT**. Data are shown as mean ± SD (n = 3). Asterisks with brackets denote significant differences between groups, determined by one‐way ANOVA and Tukey's multiple comparisons. ^***^p < 0.001, ^**^p < 0.01, ^*^p < 0.05; ns indicates no significance. Compared to Tn‐CRM197/Alum, *^###^p* < 0.001, *^##^p* < 0.01, *^#^p* < 0.05, ns indicates no difference significance.

It is well established that Th1‐type cells secrete interferon‐gamma (IFN‐γ). The production of IFN‐γ triggered by conjugates was measured using ELISA. As shown in Figure 5, all conjugates induced high levels of IFN‐γ compared to normal mouse sera (Control). Notably, the four‐component glycoconjugate **RMVT**/Rha Model produced the highest levels of IFN‐γ (96.32 pg/mL) and IL‐4 (137.07 pg/mL), indicating that **RMVT** may simultaneously target Man and recruit Rha‐based antigens. The release of IFN‐γ activates macrophages and promotes class switching to IgG2a and IgG3, while IL‐4 promotes class switching to IgG1 and IgG2b. These findings demonstrate that all fully synthetic conjugates, including Tn‐CRM197/Alum, can stimulate a mixed Th1/Th2 immune response.

#### 2.5.1 Ability of Antisera to Bind to Cancer Cells

The ability of antisera induced by the conjugates to recognize and bind to target cancer cells was then assessed using fluorescence‐activated cell sorting (FACS). The studies used the human breast cancer cell line MCF‐7 and the murine breast cancer cell line TA3Ha, both known for overexpressing the Tn antigen on their surfaces. As a control, the Tn‐negative cancer cell line MDA231 was included. In the experiments, each cancer cell line was treated with either normal mouse serum (negative control) or antisera from mice vaccinated with the conjugates. The tumor cells were then incubated with a fluorescein isothiocyanate‐labeled goat anti‐mouse IgG antibody, and the results were analyzed with FACS.

**FIGURE 6:**
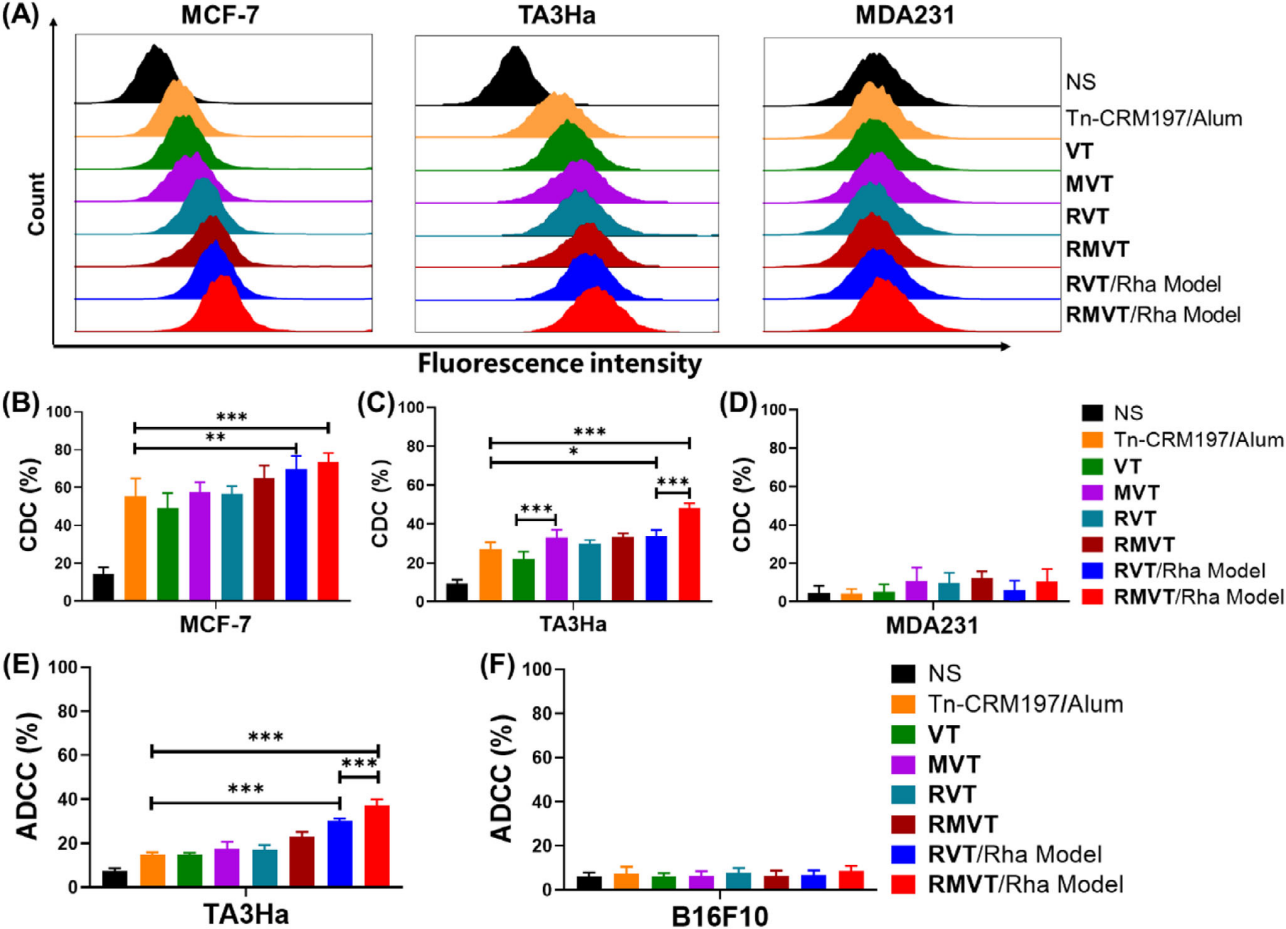
Flow cytometry analysis of antisera binding to Tn‐positive MCF‐7 cells, Tn‐positive TA3Ha cells, and Tn‐negative MDA231 cells (A). Lysis of MCF‐7 (B), TA3Ha (C), and MDA231 cancer cells (D) via antibody‐mediated CDC. Lysis of TA3Ha (E) and B16F10 cancer cells (F) via antibody‐dependent cell‐mediated cytotoxicity. Data are shown as mean ± SD (n = 6). Asterisks within brackets indicate significant differences between groups, determined by one‐way ANOVA with Tukey's multiple comparisons test. ^***^p < 0.001, ^**^p < 0.01, ^*^p < 0.05, not significant.

Significant positive shifts in fluorescent peaks were observed in MCF‐7 and TA3Ha cancer cells treated with antisera compared to cells with normal serum (Figure 6A). Conversely, the fluorescent profiles of MDA231 cancer cells treated with normal serum and corresponding antisera showed no noticeable differences. These FACS results demonstrate that all antisera generated by conjugates can specifically recognize and bind to Tn‐positive cancer cells, with little effect on Tn‐negative cells. Additionally, the binding affinity of the **RMVT**/Rha Model for MCF‐7 and TA3Ha was higher than that of the **RVT**/Rha Model, **RVT**, **MVT**, and **VT**. These findings suggest that the antibodies in the **RMVT**/Rha Model antiserum exhibited the strongest binding activity, aligning with the ELISA results, and providing further evidence that the **RMVT**/Rha Model induces a more robust immune response in mice compared to the other models.

#### 2.5.2 Antibody‐Mediated Complement‐Dependent Cytotoxicity (CDC) in CANCER cells

CDC was evaluated because the antibodies generated by all conjugates naturally showed high binding affinity for the Tn antigen. It was also tested in MCF‐7, TA3Ha, and MDA231 cancer cells. Each cell line was incubated with either normal mouse serum or antisera and then treated with rabbit complement serum. The lysis rates were determined using the lactate dehydrogenase assay.

The cell lysis assay results showed that all conjugate‐induced antisera effectively activated the complement system and caused significant cytotoxicity in Tn‐positive MCF‐7 and TA3Ha cells compared to normal mouse serum (Figure 6B,C). In contrast, Tn‐negative MDA231 cells exhibited almost no cytotoxicity across all groups (Figure 6D). Therefore, all antisera produced moderate to strong cytotoxic effects on tumor cells overexpressing the Tn antigen, whereas they did not impact Tn‐negative cells. Notably, the **RMVT**/Rha Model antiserum elicited a stronger CDC response than the other antisera, indicating that the four‐component conjugate containing Rha, Man, and vizantin is a more promising vaccine candidate for cancer immunotherapy.

#### 2.5.3 Antibody‐Dependent Cell‐Mediated Cytotoxicity (ADCC) in Cancer Cells

ADCC was tested in TA3Ha (Tn‑positive) and B16F10 (Tn‑negative) cancer cells. Each target cell line was incubated with either normal mouse serum or antisera in the presence of mouse splenocytes (as effector cells) at an effector‑to‑target (E/T) ratio of 2.5:1.

The ADCC assay results showed that all conjugate‐induced antisera effectively mediated ADCC and caused significant cytotoxicity in Tn‑positive TA3Ha cells compared to normal mouse serum (Figure 6E). In contrast, Tn‑negative B16F10 cells exhibited almost no cytotoxicity across all groups (Figure 6F). Therefore, all antisera produced moderate to strong cytotoxic effects on tumor cells overexpressing the Tn antigen, whereas they did not impact Tn‑negative cells. Notably, the RMVT/Rha Model antiserum elicited a stronger ADCC response than the other antisera, indicating that RMVT is a more promising vaccine candidate for cancer immunotherapy.

#### 2.5.4 Tumor Challenge Studies

To evaluate the immunotherapeutic effectiveness of the conjugates, we developed a tumor challenge model in Balb/c mice. In total, 80 mice were used: 50 naive mice divided into five groups (PBS, PBS/CP, Tn‐CRM197/Alum/CP, **VT**/CP, **MVT**/CP), and 30 mice with pre‐existing anti‐Rha antibodies allocated into three groups (Rha‐OVA/CP, **RVT**/Rha Model/CP, **RMVT**/Rha Model/CP). On day 0, all mice were intraperitoneally injected with TA3Ha cells (5.0 × 10^4^). One day before vaccine administration (day 1), the seven “CP” groups received cyclophosphamide (50 mg/kg, i.p.) to deplete regulatory T cells (Tregs) and thereby reduce tumor‐associated immunosuppression before immunization [51, 52, 53]. The initial vaccine dose was given subcutaneously on day 2, followed by booster doses on days 5, 7, and 11. Survival was tracked for 50 days after tumor challenge. The surviving mice were then rechallenged with a second dose of TA3Ha cells (5.0 × 10^4^) and observed for an additional 50 days to assess immune response.

**FIGURE 7:**
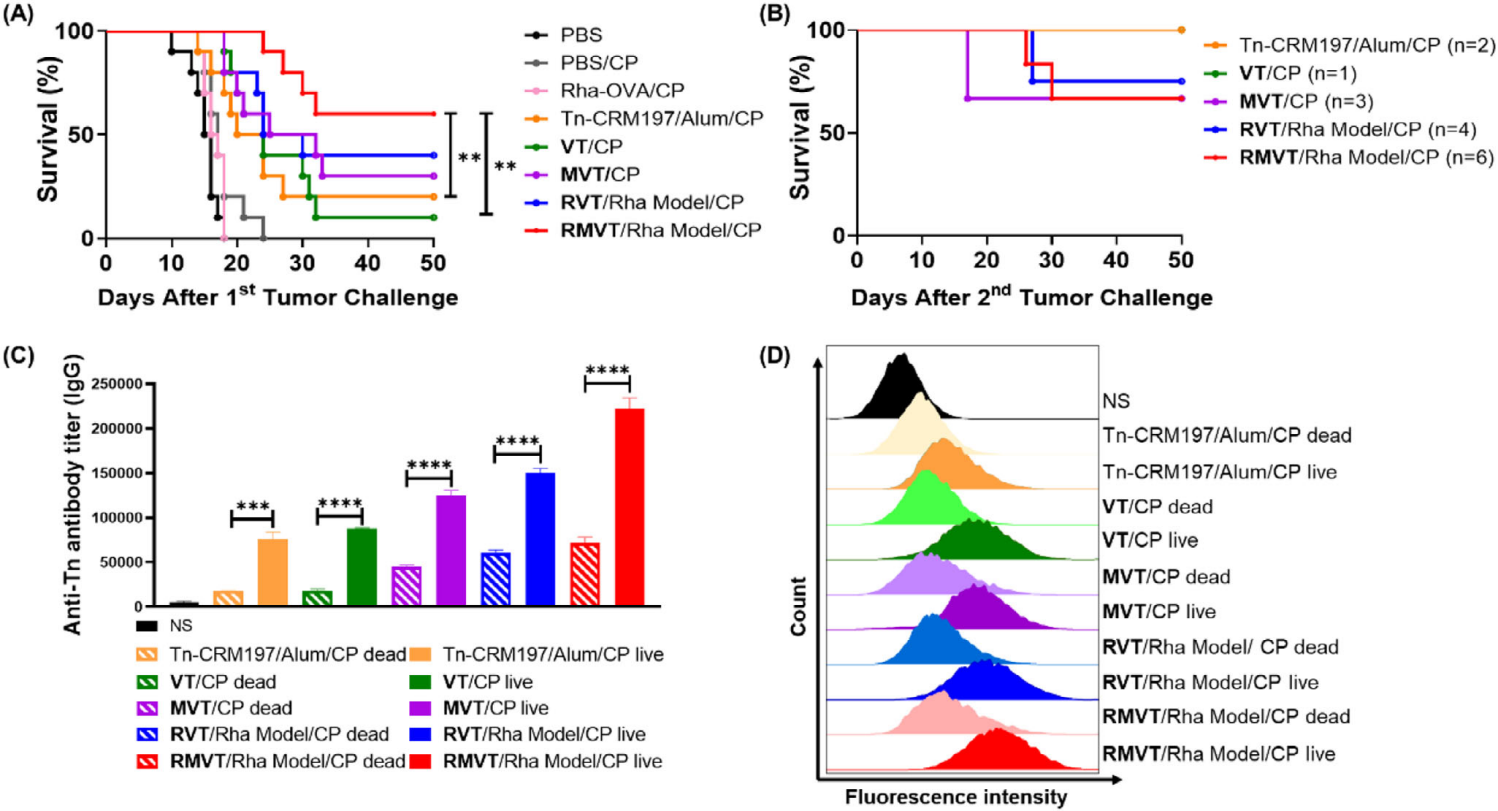
The effectiveness of various conjugates in protecting mice from tumor development. (A) Kaplan–Meier survival curves for mice immunized with Tn‐CRM197/Alum, **VT**, **MVT**, **RVT**, and **RMVT**. The Log‐rank (Mantel‐Cox) test for comparing survival curves. (B) Survival rate of mice that survived and were rechallenged with an additional 5.0 × 10^4^ TA3Ha cells. (C) IgG antibody titers in the sera of surviving and deceased mice immunized with PBS, Tn‐CRM197/Alum, **VT**, **MVT**, **RVT**, and **RMVT**. Data are shown as mean ± SD (n = 3). Statistical significance was determined using an unpaired two‐tailed Student's t‐test, with ^****^p < 0.0001. (D) FACS analysis comparing sera from live and dead mice immunized with Tn‐CRM197/Alum, **VT**, **MVT**, **RVT**, and **RMVT**.

As depicted in Figure 7A, all mice in the PBS, PBS/CP, and Rha‐OVA/CP groups died within 24 days, showing that neither CP nor Rha‐OVA could extend survival. Conversely, vaccination with the candidate vaccines protected mice against tumor growth. The average survival rates of the immunized groups, from highest to lowest, were: **RMVT**/Rha Model/CP (60%) > **RVT**/Rha Model/CP (40%) > **MVT**/CP (30%) > Tn‐CRM197/Alum/CP (20%) > **VT**/CP (10%). The tetra‐component glycoconjugate vaccine **RMVT**/CP notably increased survival time and rates, outperforming tri‐ and di‐component vaccines in protective efficacy. These findings imply that the four‐component vaccine may enhance antigen presentation through the antigen‐recruiting effects of Rha and Man targeting, thereby eliciting a strong immune response.

All surviving mice were rechallenged with 5.0 × 10^4^ TA3Ha cells on day 50. Some of these mice entirely rejected the tumor without further treatment, demonstrating the development of immune memory against TA3Ha cells (Figure 7B).

To evaluate immunoprotection levels, blood samples were taken from both surviving and deceased mice following tumor challenge. Sera from surviving mice were collected on day 30, while sera from dead mice were obtained immediately after sacrificing due to heavy tumor burden. The sera were prepared using standard protocols. ELISA measured total IgG antibody titers. Compared to normal mouse serum, sera from surviving mice showed significantly higher IgG levels (Figure 7C). The **RMVT** group exhibited the highest IgG titers, potentially explaining its superior efficacy. Sera from unprotected mice also contained IgG antibodies, but about three times lower than those from surviving mice. The capacity of the antisera to recognize and bind to TA3Ha cells was assessed via FACS. Consistent with ELISA results, sera from surviving mice bound to more TA3Ha cells than the sera from dead mice (Figure 7D). These findings suggest that high levels of Tn‐specific IgG antibodies are key to the surviving mice's resistance to the tumor challenge.

#### 2.5.5 Discussion

The comparative immunological evaluation of **VT**, **MVT**, **RVT**, and **RMVT** allowed us to dissect the contribution of each functional module. Although these vizantin‐containing derivatives displayed comparable Mincle‐binding affinity, they differed substantially in their ability to stimulate BMDMs to produce IL‐6 and TNF‐α. **RMVT** induced higher cytokine levels than **RVT** and **MVT**, and anti‐Rha serum further enhanced the cytokine responses elicited by **RMVT** and **RVT**. These observations suggest that Mincle activation is necessary but not sufficient for optimal immune stimulation, and that the Rha and Man modules play cooperative roles in enhancing APC engagement and activation.

The Rha motif may recruit endogenous anti‐Rha antibodies to form immune complexes and promote FcγR‐mediated uptake by APCs, whereas the Man motif may enhance APC targeting through mannose receptors such as CD206. These complementary uptake pathways may increase antigen capture and intracellular delivery, thereby enhancing the local availability of vizantin for Mincle activation. Subsequent activation of the Mincle‐associated Syk–CARD9–NF‐κB pathway may promote APC activation and Th1‐type cytokine production. Consistently, **RMVT** induced stronger cytokine responses than **VT** and generated a more balanced IgG subclass profile compared with the Th2‐biased Tn‐CRM197/Alum control.

Functionally, antisera from the **RMVT/**Rha model showed the strongest binding to Tn‐positive tumor cells and mediated the most potent CDC and ADCC effects. In vivo, the **RMVT**/Rha model/CP achieved the highest 50‐day survival rate and protected surviving mice against tumor rechallenge, supporting the induction of protective immune memory. Collectively, these results indicate that Rha‐mediated antibody recruitment, Man‐mediated APC targeting, and vizantin‐mediated Mincle activation act cooperatively to enhance the immunogenicity and antitumor efficacy of this fully synthetic glyco‐conjugate vaccine.

## 3 Conclusion

The comprehensive findings of this study validate the Tn antigen as a promising target for the development of synthetic anticancer vaccines. To overcome its low immunogenicity and immune‐tolerance issues, we designed and synthesized a series of fully synthetic multi‐component conjugates that incorporate built‐in adjuvants and functional sugars.

Immunological assessments revealed that the four‐component vaccine **RMVT**, particularly in the context of pre‐existing anti‐Rha antibodies, elicited the most potent and balanced Th1/Th2 immune response. It produced the highest levels of Tn‐specific IgG antibodies, including increased IgG2a, IgG2b, and IgG3 subclasses, suggesting strong T‐cell participation and a bias favoring Th1‐type immunity. This immune response was supported by elevated levels of IFN‐γ and IL‐4 in the antisera. The in vitro and in vivo tests showed that **RMVT** antiserum exhibited superior binding to Tn‐positive cancer cells and the most profound complement‐dependent cytotoxicity. Importantly, in tumor challenge studies, **RMVT** achieved the highest survival rates and elicited durable immune memory.

These findings highlight the potential of rational modular design for the creation of fully synthetic glycoconjugate vaccines. By integrating Mincle agonists with antigen‐recruiting (Rha) and ‐targeting (Man) groups, we can improve antigen presentation and steer immune responses without using traditional carriers or external adjuvants. This approach marks a significant advancement in the development of next‐generation anticancer vaccines.

## Author Contributions

**Wenbo Ming**: writing – review and editing, writing – original draft, project administration, methodology, validation, formal analysis. **Renyu Zhang**: methodology, validation, formal analysis. **XiaohuiLi**: methodology, validation, formal analysis, writing – original draft. **GuiqiLi**: methodology, validation. **Yu Niu**: data curation, writing – original draft. **DeyingYang**: formal analysis, investigation. **Xiang Luo**: supervision, formal analysis. **JunLiao**: supervision, writing – review and editing. **Zhongqiu Liu**: supervision, writing – review and editing, funding acquisition. **Guochao Liao**: supervision, conceptualization, writing – review and editing, funding acquisition, projectadministration, formal analysis.

## Funding

This work was supported by the National Natural Science Foundation of China (Nos. 92578128, 22577019, 22408066 and U22A20368), National Key Research and Development Program of China (No. 2023YFC3502800), the Department of Education of Guangdong Province (No. 2024KQNCX072), and Guangdong Basic and Applied Basic Research Foundation (Nos. 2025A1515011237 and 2023A1515110955).

## Conflicts of Interest

The authors declare no conflicts of interest.

## Supporting information




**Supporting File**: advs76235‐sup‐0001‐SuppMat.pdf.

## Data Availability

The data that support the findings of this study are available in the Supporting Information of this article.
