# Supplementary material for: A Chemically Defined Four‐Component Self‐Adjuvanting Tn Vaccine Activating Mincle, FcγR, and CD206 for Enhanced Antitumor Immunity
Source: Adv Sci (Weinh). 2026 Jun 22:e76235. Online ahead of print. doi: 10.1002/advs.76235 (PMC13336399; doi:10.1002/advs.76235)
Supplement: Supplementary file 1 — Supporting File: advs76235‐sup‐0001‐SuppMat.pdf. [file ADVS-9999-e76235-s001.pdf]

Supplementary Information

A Chemically Defined Four-Component Self-Adjuvanting Tn Vaccine Activating Mincle, FcγR, and CD206 for Enhanced Antitumor Immunity

Wenbo Ming,+[a] Renyu Zhang,+[a] Xiaohui Li,+[a] Guiqi Li,[a] Yu Niu,[a][b] Deying Yang,[c] Xiang Luo,[a][b] Jun Liao,*[d] Zhongqiu Liu,*[a][b] Guochao Liao,*[a]

[a] Dr. W. Ming, R. Zhang, X. Li, G. Li, Y. Niu, Prof. X. Luo, Prof. Z. Liu,* Prof. G. Liao*
Guangdong Provincial Key Laboratory of Translational Chinese Medicine, Joint International Research Laboratory of Translational Cancer Research of Chinese Medicines, International Institute for Translational Chinese Medicine, School of Pharmaceutical Sciences
Guangzhou University of Chinese Medicine
Guangzhou 510006, China
E-mail: liuzq@gzucm.edu.cn; liao@gzucm.edu.cn

[b] Y. Niu, Prof. X. Luo, Prof. Z. Liu
Chinese Medicine Guangdong Laboratory
Hengqin 519031, China

[c] Dr. D. Yang
Affiliated Hospital of Guangdong Medical University
Zhanjiang 524001, China

[d] Prof. J. Liao*
Department of Biochemistry and Molecular Biology, College of Basic Medical Sciences
Second Military Medical University
Shanghai 200433,China
E-mail: jliao@smmu.edu.cn

+These authors made equal contributions to this study.

Table of Contents

1. General Information S3

2. Experiment Section S4

2.1 Synthesis and Characterization of Rha-Man Building Block S4

2.2 Synthesis and Characterization of Four-Component Vaccine RMVT S15

2.3 Synthesis and Characterization of Three-Component Vaccine RVT S24

2.4 Synthesis and Characterization of Three-Component Vaccine MVT S31

2.5 Synthesis and Characterization of Two-Component Vaccine VT S40

2.6 Preparation of Tn-CRM197 .... S43

2.7 Cytokine of Murine Bone-Marrow-Derived Macrophage Analysis S43

2.8 Human Mincle Binding Assay S44

2.9 Liposome Size Analysis of the Conjugates VT, MVT, RVT, and RMVT .. S45

2.10 Mouse Immunization S47

2.11 ELISA Protocol and Results S49

2.12 Results of IFN-γ and IL-4 Levels Provoked by Conjugates S52

2.13 FACS Analyses S53

2.14 Complement-Dependent Cytotoxicity (CDC) S53

2.15 Antibody-dependent cell-mediated cytotoxicity assay (ADCC) S54

2.16 Tumor Challenge Study S55

3. NMR and MS Spectra of New Compounds S58

4. Reference S134

1. General Information

Unless noted otherwise, commercially available chemicals were used without further purification. 4 Å Molecular sieves were flame-dried under vacuum and cooled to room temperature under N_2 atmosphere before use. The reactions were monitored by thin-layer chromatography (TLC) on glass-packed precoated silica gel plates and visualized by a UV detector or staining with 25% H_2SO_4 in EtOH (v/v). Purification of products was accomplished by flash column chromatography on silica gel (200–300 mesh). NMR spectra were recorded on a Bruker Avance III 400 spectrometer (^{1}H at 400 MHz, ^{13}C{^{1}H} at 101 MHz) with chemical shifts reported in ppm using TMS as the internal standard. Signal splitting patterns are described as singlet (s), doublet (d), triplet (t), quartet (q), or multiplet (m), with coupling constants (*J*) in hertz. The high-resolution electron spray ionization mass spectra (HRMS (ESI)) were obtained using Agilent 6540 UHD Accurate-Mass Quadrupole Time-of-Flight (Q-TOF) mass spectrometer, Waters Micromass-LCT Premier-XE mass spectrometer, or Thermo Q-Exactive Orbitrap Mass Spectrometers.

Alum adjuvant was purchased from Thermo Fisher. TA3Ha was purchased from the European Collection of Authenticated Cell Cultures (ECACC). MCF-7 and MDA-231 cancer cells were purchased from the American Type Culture Collection (ATCC). DPBS, Minimum Eagle's medium (MEM), Dulbecco's modified Eagle medium (DMEM), and fetal bovine serum (FBS) were purchased from Procell Life Science & Technology Co., Ltd. Human Mincle-Fc protein was purchased from Sino Biological Inc. Trypsin-EDTA, HRP-linked goat anti-mouse kappa, IgM, and IgG were purchased from Invitrogen. HRP-linked goat anti-mouse IgG1, IgG2a, IgG2b, and IgG3 antibodies were purchased from Abcam. FITC-labeled goat anti-mouse IgG antibody was purchased from Beyotime Biotechnology. Rabbit complements and cyclophosphamide were purchased from Sigma-Aldrich. LDH Cytotoxicity Detection Kit was purchased from Roche Biotechnology. Recombinant Mouse M-CSF (carrier-free), Mouse IFN-γ, and Mouse IL-4 ELISA Kit were purchased from BioLegend.

Female Balb/c mice used in this study were bred and maintained in the animal facility

in the specific pathogen-free (SPF) animal laboratory (License number: SYXK (Guangzhou) 2019-0144) at Guangzhou University of Chinese Medicine (Guangzhou, China). The animal protocol for this study was approved by the Guangzhou University of Traditional Chinese Medicine Animal Care and Use Committee and was performed in compliance with relevant Chinese laws and institutional guidelines.

2. Experiment Section

2.1 Synthesis and Characterization of Rha-Man Building Block

(2*R*,3*R*,4*R*,5*S*,6*S*)-2-(2-(2-azidoethoxy)ethoxy)-6-methyltetrahydro-2H-pyran-3,4,5-triyl triacetate (13)

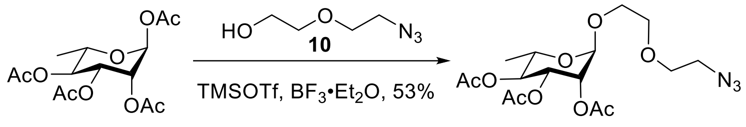

12 **13**

Compound **12** (40 g, 0.119 mol) was dissolved in acetonitrile. Boron trifluoride diethyl ether (58 mL, 0.476 mol) and trifluoromethanesulfonic acid trimethylsilyl ester (8.6 mL, 0.0476 mol) were added dropwise over an ice bath. The reaction was stirred for 4 hours and monitored by TLC staining with 25% sulfuric acid in ethanol (developing solvent: EA/PE = 1:1). Complete conversion was confirmed by TLC analysis. The reaction was quenched with water and extracted with ethyl acetate (200 mL × 3). The organic layer was washed with saturated sodium bicarbonate solution, and separated using a separatory funnel. The organic phase was collected and dried over anhydrous sodium sulfate, and filtered. Ethyl acetate was then distilled under reduced pressure to give a yellow viscous crude product. The crude product was purified by silica gel column chromatography (EA/PE, from 1:5 to 1:1) to afford compound **13** as a colorless, transparent, viscous liquid (22 g, 53% yield). **^{1}H NMR (400 MHz, CDCl$_3$)** δ 5.32 (dd, J = 10.0, 3.5 Hz, 1H), 5.27 (dd, J = 3.4, 1.7 Hz, 1H), 5.07 (t, J = 9.9 Hz, 1H), 4.80 (d, J = 2.0 Hz, 1H), 4.03–3.89 (m, 1H), 3.87–3.78 (m, 1H), 3.74–3.62 (m, 5H), 3.43–3.36 (t, J = 8.0 Hz, 2H), 2.15 (s, 3H), 2.05 (s, 3H), 1.99 (s, 3H), 1.22 (d, J = 6.2 Hz, 3H). **^{13}C{^{1}H} NMR (101 MHz, CDCl$_3$)** δ 170.16, 170.07, 170.05, 97.62, 77.37, 77.26, 77.06, 76.74, 71.12, 70.23, 70.18, 69.82, 69.12, 67.12, 66.35, 50.77, 20.93, 20.82, 20.75, 17.41.

(2*R*,3*R*,4*R*,5*S*,6*S*)-2-(((*S*)-5-(tert-butoxycarbonyl)-1-(9H-fluoren-9-yl)-3,8-dioxo-2,12-dioxa-4,9-diazatetradecan-14-yl)oxy)-6-methyltetrahydro-2H-pyran-3,4,5-triyl triacetate (14)

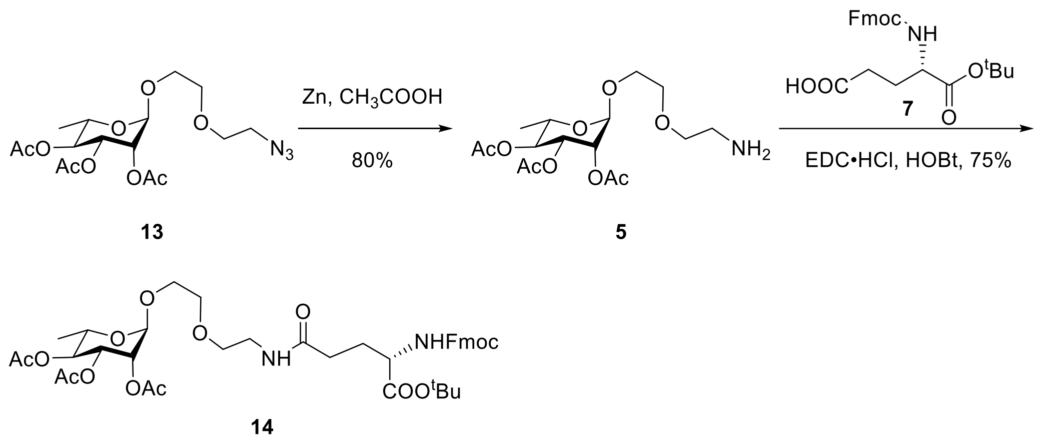

Compound **13** (18 g, 0.119 mol) was dissolved in DCM. 100 g zinc powder was added, and then 120 mL glacial acetic acid was added dropwise under an ice bath. The reaction was stirred for 2 hours and monitored by TLC staining with 25% sulfuric acid in ethanol (developing solvent: DCM/MeOH = 10:1). Complete conversion by confirmed by TLC analysis. Glacial acetic acid and DCM were removed by vacuum distillation to obtain a pale-yellow crude product. Purification by silica gel column chromatography yielded compound **5** as a colorless, transparent, viscous liquid (16 g, 90% yield). Due to the instability of compound **5**, it was used directly to the next step. Compound **5** (15.0 g, 0.0397 mol), compound **7** (22.5 g, 0.0595 mol), 1-(3-dimethylaminopropyl)-3-ethylcarbodiimide hydrochloride (30 g, 0.0804 mol), and HOBt (1 g, 0.0074 mol) were dissolved in DCM (30 mL). The reaction was stirred at room temperature for 3 hours and monitored by TLC staining with 25% sulfuric acid in ethanol (developing solvent: MeOH/DCM = 1:40). Complete conversion was confirmed by TLC analysis. DCM (200 mL) was added into the reaction and the mixture was transferred to a separatory funnel. It was washed sequentially with water (100 mL × 2) and saturated NaCl solution (100 mL). The organic layer was collected, dried over anhydrous sodium sulfate, and filtered. The filtrate was distilled under reduced pressure to remove organic solvent, yielding the crude product. The crude product was purified by silica gel column chromatography (MeOH/DCM, from 1:200 to 1:80) to afford compound **14** as a white

solid (20 g, yield: 70%). **^{1}H NMR (400 MHz, Chloroform-d)** *δ* 7.74 (d, J = 7.5 Hz, 2H), 7.60 (dd, J = 7.5, 4.8 Hz, 2H), 7.39 (d, J = 7.4 Hz, 2H), 7.31 – 7.27 (m, 2H), 6.42 (s, 1H), 5.85 (d, J = 8.2 Hz, 1H), 5.30 – 5.27 (m, 1H), 5.24 – 5.22 (m, 1H), 5.06 (t, J = 9.9 Hz, 1H), 4.81 (d, J = 1.9 Hz, 1H), 4.41 – 4.36 (m, 1H), 4.33 (t, J = 5.4 Hz, 1H), 4.21 (d, J = 7.3 Hz, 2H), 3.89 (dd, J = 10.0, 6.0 Hz, 1H), 3.78 – 3.73 (m, 1H), 3.66 – 3.62 (m, 1H), 3.62 – 3.56 (m, 2H), 3.53 – 3.51 (m, 2H), 3.49 – 3.44 (m, 1H), 3.42 – 3.37 (m, 1H), 2.28 (t, J = 6.6 Hz, 2H), 2.23 – 2.15 (m, 2H), 2.13 (s, 3H), 2.02 (s, 3H), 1.97 (s, 3H), 1.45 (s, 9H), 1.20 (d, J = 6.2 Hz, 3H). **^{13}C{^{1}H} NMR (101 MHz, CDCl$_3$)** *δ* 172.28, 171.22, 170.71, 170.29, 170.12, 169.75, 156.29, 143.98, 143.75, 141.29, 141.27, 127.70, 127.07, 125.20, 125.14, 119.97, 97.52, 82.19, 77.42, 77.30, 77.10, 76.78, 70.06, 69.94, 69.74, 68.96, 68.44, 67.04, 66.94, 66.26, 62.55, 54.21, 47.18, 39.35, 32.36, 28.47, 27.99, 20.91, 20.75, 20.69. **HRMS (ESI):** *m/z* calcd for $C_{40}H_{52}N_2O_{14}Na$ [M+Na]$^+$ 807.3331; Found, 807.3336.

N^2-(((9*H*-fluoren-9-yl)methoxy)carbonyl)-N^5-(2-(2-(((2*R*,3*R*,4*R*,5*S*,6*S*)-3,4,5-triacetoxy-6-methyltetrahydro-2*H*-pyran-2-yl)oxy)ethoxy)ethyl)-L-glutamine (15)

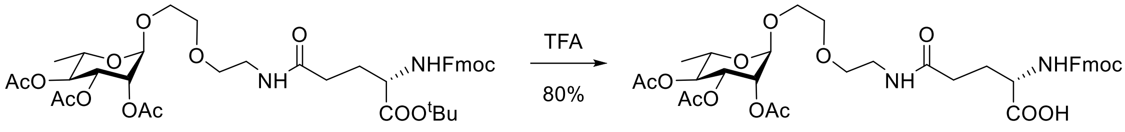

14 **15**

Compound **14** (18 g, 0.119 mol) was dissolved in 50 mL DCM. Under an ice bath, 12.5 mL TFA was added dropwise. The reaction was stirred for 10 hours and monitored by TLC staining with 25% sulfuric acid in ethanol (developing solvent: DCM/MeOH = 10:1). Complete conversion was confirmed by TLC analysis. Glacial acetic acid and DCM were removed by vacuum distillation to obtain a pale-yellow crude product. Further purification by silica gel column chromatography (MeOH/DCM, from 1:90 to 1:10) afforded a colorless oily compound **15** (14 g, 75% yield). **^{1}H NMR (400 MHz, CDCl$_3$)** *δ* 7.74 (d, J = 7.5 Hz, 2H), 7.59 (t, J = 6.9 Hz, 2H), 7.38 (t, J = 7.4 Hz, 2H), 7.31 – 7.27 (m, 2H), 6.84 (s, 1H), 6.13 (d, J = 7.1 Hz, 1H), 5.30 – 5.26 (m, 1H), 5.23 (dd, J = 3.5, 1.7 Hz, 1H), 5.08 (d, J = 9.9 Hz, 1H), 4.82 (d, J = 1.8 Hz, 1H), 4.39 – 4.34 (m, 2H), 4.31 (d, J = 3.3 Hz, 1H), 4.19 (t, J = 7.2 Hz, 1H), 3.89 (dd, J = 9.8, 6.2 Hz,

1H), 3.78 – 3.73 (m, 1H), 3.65 – 3.61 (m, 2H), 3.57 (t, J = 2.5 Hz, 1H), 3.53 (d, J = 4.6 Hz, 2H), 3.49 (t, J = 4.9 Hz, 1H), 3.41 (d, J = 5.1 Hz, 1H), 2.49 – 2.35 (m, 2H), 2.18 (d, J = 6.4 Hz, 1H), 2.13 (s, 3H), 2.10 – 2.06 (m, 1H), 2.03 (s, 3H), 1.98 (s, 3H), 1.20 (d, J = 6.2 Hz, 3H). **$^{13}C\{^1H\}$ NMR (101 MHz, CDCl₃)** *δ* 173.88, 173.45, 170.88, 170.53, 170.45, 169.82, 156.27, 143.91, 143.69, 141.28, 141.26, 127.73, 127.10, 125.20, 125.14, 119.98, 97.52, 77.38, 77.26, 77.06, 77.00, 76.74, 69.97, 69.74, 69.09, 68.46, 67.10, 67.04, 66.19, 62.59, 53.41, 47.11, 39.64, 32.29, 28.72, 20.93, 20.77, 20.73, 20.71. **HRMS (ESI):** *m/z* calcd for $C_{36}H_{44}N_2O_{14}Na$ $[M+Na]^+$ 751.2685; Found, 751.2690.

(2*R*,3*R*,4*S*,5*S*,6*S*)-2-(acetoxymethyl)-6-(2-(2-azidoethoxy)ethoxy)tetrahydro-2H-pyran-3,4,5-triyl triacetate (11)

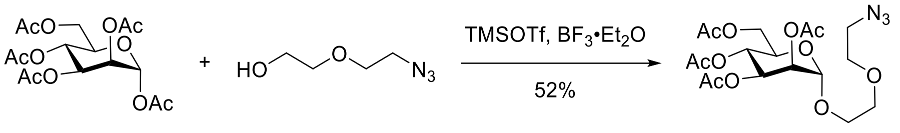

Compound **9** (40 g, 0.119 mol) was dissolved in acetonitrile. Boron trifluoride diethyl ether (58 mL, 0.476 mol) and trifluoromethanesulfonic acid trimethylsilyl ester (8.6 mL, 0.0476 mol) were added dropwise over an ice bath. The reaction was stirred for 4 hours and monitored by TLC staining with 25% sulfuric acid in ethanol (developing solvent: EA/PE = 1:1). Complete conversion was confirmed by TLC analysis. The reaction was quenched with water and extracted with ethyl acetate (200 mL × 3). Organic layers were collected, washed with saturated sodium bicarbonate solution, and separated using a separatory funnel. The organic phase was collected and dried over anhydrous sodium sulfate. Ethyl acetate was removed under reduced pressure, yielding a yellow viscous crude product. Further purification by column chromatography (EA/PE, from 1:5 to 1:1) gave compound **11** as a colorless, transparent, viscous liquid (22 g, 52% yield). **^1H NMR (400 MHz, CDCl₃)** *δ* 5.38 (dd, J = 10.0, 3.4 Hz, 1H), 5.34–5.22 (m, 2H), 4.89 (d, J = 1.8 Hz, 1H H-1), 4.30 (dd, J = 12.4, 5.2 Hz, 1H), 4.15–4.05 (m, 2H), 3.89–3.80 (m, 1H), 3.69 (td, J = 5.2, 2.6 Hz, 5H), 3.40 (d, J = 5.5, 2H), 2.16 (s, 3H), 2.11 (s, 3H), 2.04 (s, 3H), 2.00 (s, 3H). **$^{13}C\{^1H\}$ NMR (101 MHz, CDCl₃)** *δ* 170.69, 170.07, 169.94, 169.78, 97.73, 77.39, 77.27, 77.07, 76.75, 70.20, 70.06, 69.52, 69.08, 68.42, 67.31, 66.11, 62.44, 50.73, 20.90, 20.76, 20.70.

(2*S*,3*S*,4*S*,5*R*,6*R*)-2-(((*S*)-8-((((9*H*-fluoren-9-yl)methoxy)carbonyl)amino)-7,11-dioxo-17-(((2*R*,3*R*,4*R*,5*S*,6*S*)-3,4,5-triacetoxy-6-methyltetrahydro-2*H*-pyran-2-yl)oxy)-3,15-dioxa-6,12-diazaheptadecyl)oxy)-6-(acetoxymethyl)tetrahydro-2*H*-pyran-3,4,5-triyl triacetate (16)

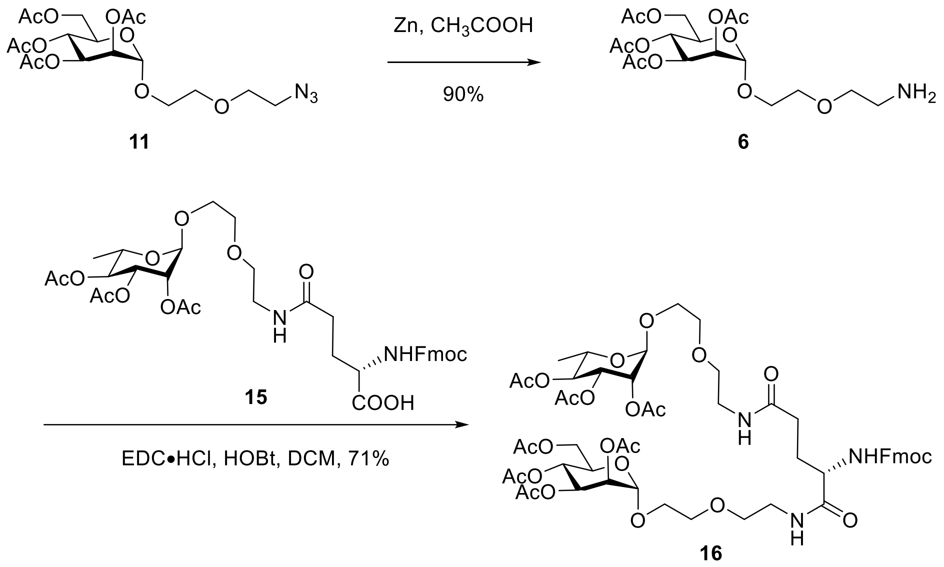

Compound **11** (18 g, 0.119 mol) was dissolved in DCM. 100 g of zinc powder was added into the solution, and then 120 mL of glacial acetic acid was added dropwise over an ice bath. The reaction was stirred for 2 hours and monitored by TLC staining with 25% sulfuric acid in ethanol (developing solvent: DCM/MeOH = 10:1). Complete conversion was confirmed by TLC analysis. Glacial acetic acid and DCM were removed by vacuum distillation to obtain a pale-yellow crude product. Further purification using column chromatography to yield compound **6** as a colorless oil (16 g, 90% yield). Due to the instability of compound **6**, it was used directly to the next step. Compound **15** (15.0 g, 0.0206 mol), compound **6** (9.4 g, 0.0206 mol), 1-(3-dimethylaminopropyl)-3-ethylcarbodiimide hydrochloride (EDC•HCl, 17.0 g, 0.089 mol), and 1-Hydroxybenzotriazole (HOBt, 1.0 g, 0.0074 mol) were dissolved in DCM (30 mL). The reaction mixture was stirred the at room temperature for 3 hours. The reaction was monitored by TLC staining with 25% sulfuric acid in ethanol (developing solvent: MeOH/DCM = 1:40). Complete conversion of starting materials was confirmed by TLC. Then the reaction was diluted with DCM (200 mL) and transferred

to a separatory funnel. It was washed sequentially with water (100 mL) and saturated NaCl solution (100 mL). The organic layer was collected, dried over anhydrous sodium sulfate, and filtered. The organic solvent was distilled under reduced pressure, yielding a crude product. Further purification by column chromatography (MeOH/DCM, from 1:200 to 1:80) gave compound **16** as a white solid (20 g, 71% yield). **^{1}H NMR (400 MHz, CDCl$_3$)** *δ* 1H NMR (400 MHz, Chloroform-d) δ 7.75 (d, J = 7.5 Hz, 2H), 7.60 (d, J = 7.5 Hz, 2H), 7.39 (t, J = 7.4 Hz, 2H), 7.30 (t, J = 7.4 Hz, 2H), 7.20 (s, 1H), 6.59 (s, 1H), 6.29 – 6.17 (m, 1H), 5.33 (dd, J = 10.0, 3.3 Hz, 1H), 5.29 (d, J = 1.8 Hz, 1H), 5.26 (d, J = 5.1 Hz, 1H), 5.23 (d, J = 3.1 Hz, 1H), 5.06 (t, J = 9.9 Hz, 1H), 4.90 (s, 1H), 4.81 (s, 1H), 4.34 (d, J = 7.2 Hz, 2H), 4.26 – 4.21 (m, 2H), 4.10 (dd, J = 12.3, 2.5 Hz, 1H), 4.02 (ddd, J = 8.1, 5.2, 2.5 Hz, 1H), 3.88 (dt, J = 12.4, 6.3 Hz, 1H), 3.77 (d, J = 10.6 Hz, 2H), 3.66 – 3.50 (m, 14H), 3.44 (s, 2H), 2.33 (dd, J = 19.9, 12.6 Hz, 4H), 2.15 (s, 3H), 2.13 (s, 3H), 2.08 (s, 3H), 2.02 (s, 6H), 1.98 (d, J = 1.9 Hz, 6H), 1.20 (d, J = 6.3 Hz, 3H). **^{13}C{^{1}H} NMR (101 MHz, CDCl$_3$)** *δ* 172.90, 171.59, 170.75, 170.42, 170.32, 170.29, 170.09, 170.00, 169.79, 156.28, 143.92, 143.81, 141.29, 141.26, 127.73, 127.09, 125.21, 119.99, 97.58, 97.52, 77.38, 77.26, 77.06, 77.06, 76.74, 71.01, 70.03, 69.96, 69.90, 69.69, 69.08, 69.03, 68.43, 67.12, 66.96, 66.46, 66.18, 62.52, 54.33, 47.15, 39.42, 39.36, 32.38, 29.25, 20.97, 20.95, 20.82, 20.78, 20.73, 17.43. **HRMS (ESI):** *m/z* calcd for $C_{54}H_{71}N_3O_{24}Na$ $[M+Na]^+$ 1168.4320; Found, 1168.4337.

(2*S*,3*S*,4*S*,5*R*,6*R*)-2-(((*S*)-8-((((9*H*-fluoren-9-yl)methoxy)carbonyl)amino)-7,11-dioxo-17-(((2*R*,3*R*,4*R*,5*S*,6*S*)-3,4,5-triacetoxy-6-methyltetrahydro-2*H*-pyran-2-yl)oxy)-3,15-dioxa-6,12-diazaheptadecyl)oxy)-6-(acetoxymethyl)tetrahydro-2*H*-pyran-3,4,5-triyl triacetate (17)

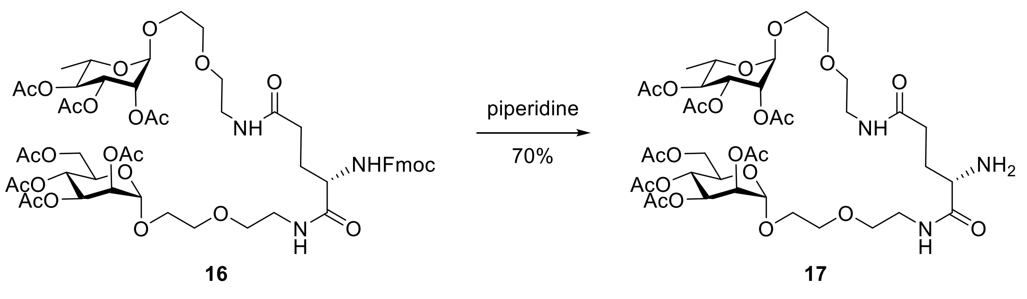

Compound **16** (10 g, 0.119 mol) was dissolved in 30 mL DMF. Under an ice bath, 0.6

mL piperidine was added dropwise. The reaction mixture was stirred for 10 hours and monitored by TLC staining with 25% sulfuric acid in ethanol (DCM/MeOH = 10:1). Complete conversion was confirmed by TLC. DMF was removed by vacuum distillation to obtain a pale-yellow crude product. Purification by column chromatography (MeOH/DCM, from 1:90 to 1:10) afforded a colorless oily compound **17** (7 g, 70% yield). **^{1}H NMR (400 MHz, CDCl$_3$)** δ 7.96 (s, 1H), 6.87 (s, 1H), 5.30 – 5.28 (m, 1H), 5.26 (d, J = 3.6 Hz, 1H), 5.25 – 5.23 (m, 1H), 5.21 (dd, J = 3.5, 1.8 Hz, 1H), 5.04 (t, J = 9.9 Hz, 1H), 4.87 (d, J = 1.7 Hz, 1H), 4.80 (d, J = 1.7 Hz, 1H), 4.25 (dd, J = 12.3, 5.1 Hz, 1H), 4.09 (dd, J = 12.3, 2.5 Hz, 1H), 4.04 – 3.99 (m, 1H), 3.88 (dd, J = 9.7, 6.2 Hz, 1H), 3.76 (tdd, J = 11.5, 8.8, 6.2 Hz, 4H), 3.64 (ddd, J = 14.6, 10.3, 5.6 Hz, 7H), 3.55 (dt, J = 10.9, 5.1 Hz, 4H), 3.43 (ddt, J = 13.2, 8.9, 5.2 Hz, 4H), 3.13 (s, 1H), 2.43 (t, J = 6.8 Hz, 2H), 2.14 (s, 3H), 2.13 (s, 3H), 2.08 (s, 3H), 2.03 (s, 3H), 2.03 (s, 3H), 1.97 (d, J = 1.2 Hz, 6H), 1.87 (s, 1H), 1.72 – 1.55 (m, 1H), 1.20 (d, J = 6.3 Hz, 3H). **^{13}C{^{1}H} NMR (101 MHz, CDCl$_3$)** δ 173.16, 172.77, 170.76, 170.39, 170.27, 170.23, 170.13, 170.02, 169.76, 97.64, 97.50, 77.39, 77.27, 77.07, 76.75, 70.99, 69.93, 69.91, 69.85, 69.70, 69.65, 69.54, 69.13, 69.11, 68.43, 67.22, 66.97, 66.43, 66.11, 62.47, 53.90, 44.60, 39.36, 39.09, 32.62, 29.86, 22.50, 22.39, 20.98, 20.94, 20.84, 20.79, 20.74, 17.43. **HRMS (ESI):** *m/z* calcd for $C_{39}H_{61}N_3O_{22}Na$ $[M+Na]^+$ 946.3639; Found, 946.3685.

(2*R*,3*R*,4*S*,5*S*,6*S*)-2-(acetoxymethyl)-6-(((5*S*,9*S*)-5-(tert-butoxycarbonyl)-1-(9*H*-fluoren-9-yl)-3,7,10-trioxo-9-(3-oxo-3-((2-(2-(((2*R*,3*R*,4*R*,5*S*,6*S*)-3,4,5-triacetoxy-6-methyltetrahydro-2*H*-pyran-2-yl)oxy)ethoxy)ethyl)amino)propyl)-2,14-dioxa-4,8,11-triazahexadecan-16-yl)oxy)tetrahydro-2*H*-pyran-3,4,5-triyl triacetate (18)

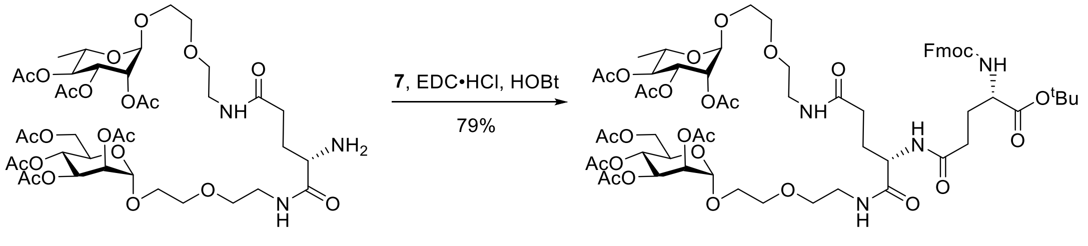

17 18

Compound **17** (7 g, 0.0075 mol), Compound **7** (4.8 g, 0.0112 mol), EDC•HCl (4.8 g,

0.0375 mol), and HOBt (100 mg, 0.00074 mol) were dissolved in DCM (30 mL). The reaction mixture was stirred at room temperature for 3 hours and monitored by TLC staining with 25% sulfuric acid in ethanol (developing solvent: MeOH/DCM = 1:40). Complete conversion was confirmed by TLC. The reaction was diluted with DCM (200 mL) and transferred to a separatory funnel. Washed sequentially with water (100 mL) and saturated NaCl solution (100 mL). The organic layer was collected, dried over anhydrous sodium sulfate, and filtered. The filtrate was distilled under reduced pressure to remove the organic solvent, yielding the crude product. Purification by column chromatography (MeOH/DCM, from 1:200 to 1:80) afforded compound **18** as a white solid (8 g, yield: 79%). **^{1}H NMR (101 MHz, CDCl$_3$)** *δ*7.76 (d, J = 2.5 Hz, 2H), 7.61 – 7.59 (m, 2H), 7.39 (s, 2H), 7.31 (s, 2H), 7.22 (d, J = 6.0 Hz, 1H), 7.06 (d, J = 7.3 Hz, 1H), 6.70 (t, J = 5.6 Hz, 1H), 5.75 (d, J = 8.2 Hz, 1H), 5.33 (d, J = 6.7 Hz, 1H), 5.30 (d, J = 3.5 Hz, 1H), 5.28 (t, J = 1.8 Hz, 1H), 5.26 – 5.24 (m, 1H), 5.23 (dd, J = 3.2, 1.8 Hz, 1H), 5.22 – 5.21 (m, 1H), 5.06 (s, 1H), 4.90 (d, J = 1.8 Hz, 1H), 4.79 (d, J = 1.8 Hz, 1H), 4.44 (d, J = 6.6 Hz, 1H), 4.37 (dd, J = 7.3, 3.1 Hz, 2H), 4.27 (dd, J = 12.2, 5.0 Hz, 2H), 4.21 (t, J = 6.0 Hz, 2H), 4.12 – 4.08 (m, 1H), 4.04 – 4.00 (m, 1H), 3.90 – 3.86 (m, 1H), 3.79 – 3.73 (m, 2H), 3.68 (dd, J = 6.2, 3.5 Hz, 1H), 3.65 (s, 1H), 3.63 – 3.57 (m, 6H), 3.53 (d, J = 5.3 Hz, 4H), 3.40 (d, J = 5.3 Hz, 2H), 2.41 – 2.33 (m, 2H), 2.29 (d, J = 6.8 Hz, 2H), 2.15 (s, 3H), 2.13 (s, 3H), 2.09 (s, 3H), 2.03 (s, 6H), 1.99 (s, 3H), 1.98 (s, 3H), 1.95 – 1.88 (m, 2H), 1.46 (s, 9H), 1.20 (d, J = 6.2 Hz, 3H). **^{13}C{^{1}H} NMR (101 MHz, CDCl$_3$)** *δ* 173.76, 173.50, 172.85, 171.68, 170.83, 170.54, 170.43, 170.41, 170.22, 170.02, 169.81, 156.07, 143.88, 143.75, 141.28, 127.75, 127.12, 125.17, 119.99, 97.60, 97.47, 77.37, 77.26, 77.06, 76.74, 70.95, 69.93, 69.76, 69.65, 69.16, 69.10, 68.45, 67.04, 66.85, 66.47, 66.11, 63.51, 62.50, 53.07, 52.87, 47.12, 39.44, 39.24, 32.07, 31.52, 28.47, 27.94, 20.98, 20.95, 20.83, 20.80, 20.74, 17.41 **HRMS (ESI):** *m/z* calcd for $C_{63}H_{86}N_4O_{27}Na$ [M+Na]$^+$ 1353.5372; Found, 1353.5344.

N^2-(((9*H*-fluoren-9-yl)methoxy)carbonyl)-N^4-((*S*)-7,11-dioxo-1-(((2*S*,3*S*,4*S*,5*R*,6*R*)-3,4,5-triacetoxy-6-(acetoxymethyl)tetrahydro-2*H*-pyran-2-yl)oxy)-17-(((2*R*,3*R*,4*R*,5*S*,6*S*)-3,4,5-triacetoxy-6-methyltetrahydro-2*H*-pyran-2-yl)oxy)-3,15-dioxa-6,12-diazaheptadecan-8-yl)-*L*-asparagine (19)

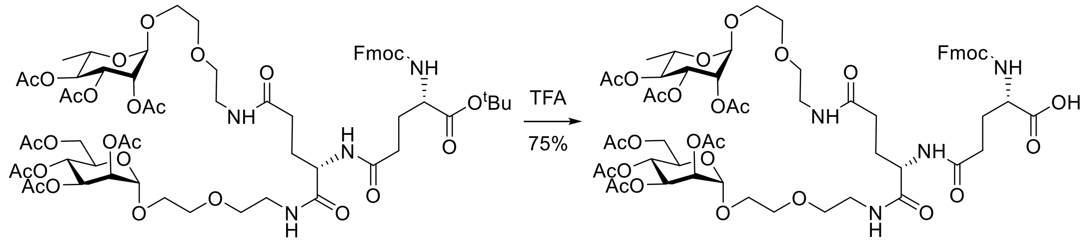

18 **19**

Compound **18** (8.0 g, 0.0060 mol) was dissolved in 50 mL DCM. 12.5 mL TFA was added dropwise over an ice bath. The reaction was stirred for 10 hours, and monitored by TLC staining with 25% sulfuric acid in ethanol (developing solvent: DCM/MeOH = 10:1). Complete conversion was confirmed by TLC analysis. Glacial acetic acid and DCM were removed by vacuum distillation to give a pale-yellow crude product. Purification by silica gel column chromatography (MeOH/DCM, from 1:100 to 1:10) yielded a colorless oily compound **19** (5.6 g, 75% yield). **^{1}H NMR (400 MHz, $CDCl_3$)** δ 7.75 (d, J = 7.5 Hz, 2H), 7.60 (dd, J = 7.7, 3.5 Hz, 2H), 7.49 (d, J = 7.3 Hz, 1H), 7.39 (t, J = 7.4 Hz, 2H), 7.31 (td, J = 7.4, 1.2 Hz, 2H), 6.89 (s, 1H), 6.05 (d, J = 7.5 Hz, 1H), 5.37 – 5.17 (m, 5H), 5.07 (t, J = 9.9 Hz, 1H), 4.91 (d, J = 1.7 Hz, 1H), 4.80 (d, J = 1.6 Hz, 1H), 4.50 – 4.39 (m, 2H), 4.36 (d, J = 7.2 Hz, 2H), 4.28 (dd, J = 12.3, 5.1 Hz, 1H), 4.21 (t, J = 7.2 Hz, 1H), 4.11 (dd, J = 12.2, 2.5 Hz, 1H), 4.05 (dt, J = 9.6, 2.6 Hz, 1H), 3.88 (dd, J = 9.8, 6.2 Hz, 1H), 3.82 – 3.32 (m, 16H), 2.40 (dt, J = 16.2, 7.1 Hz, 2H), 2.35 – 2.19 (m, 2H), 2.16 (s, 3H), 2.14 (s, 3H), 2.10 (s, 3H), 2.05 (s, 6H), 1.99 (s, 6H), 1.21 (d, J = 6.3 Hz, 3H). **$^{13}C\{^1H\}$ NMR (101 MHz, $CDCl_3$)** δ 173.76, 173.50, 172.85, 171.68, 170.83, 170.54, 170.43, 170.41, 170.22, 170.02, 169.81, 156.07, 143.88, 143.75, 141.28, 127.75, 127.12, 125.17, 119.99, 97.60, 97.47, 77.37, 77.26, 77.06, 76.74, 70.95, 69.93, 69.76, 69.65, 69.16, 69.10, 68.45, 67.04, 66.85, 66.47, 66.11, 63.51, 62.50, 53.07, 52.87, 47.12, 39.44, 39.24, 32.07, 31.52, 28.47, 27.94, 20.98, 20.95, 20.83, 20.80, 20.74, 17.41.

(2*S*,3*S*,4*S*,5*R*,6*R*)-2-(((8*S*,13*S*)-13-((((9*H*-fluoren-9-yl)methoxy)carbonyl)amino)-7,10,14-trioxo-8-(3-oxo-3-((2-(2-(((2*R*,3*R*,4*R*,5*S*,6*S*)-3,4,5-triacetoxy-6-methyltetrahydro-2*H*-pyran-2-yl)oxy)ethoxy)ethyl)amino)propyl)-3,18,21-trioxa-6,9,15-triazatetracos-23-yn-1-yl)oxy)-6-(acetoxymethyl)tetrahydro-2*H*-pyran-3,4,5-triyl triacetate (20)

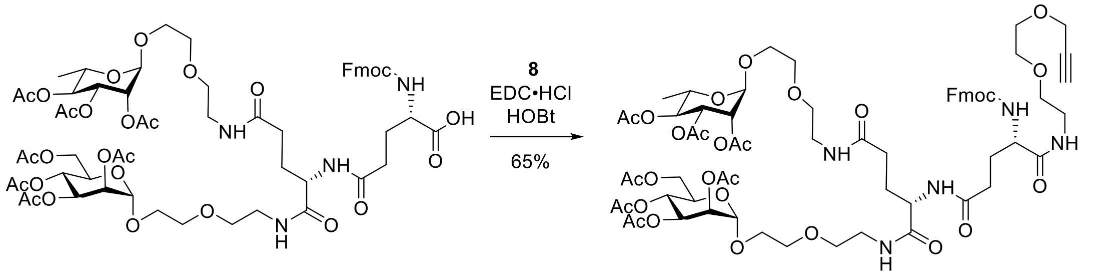

19 **20**

Compound **19** (5.6 g, 0.0043 mol), compound **8** (0.9295 g, 0.0065 mol), EDC•HCl (6.2 g, 0.0325 mol), and HOBt (100 mg, 0.00074 mol) were dissolved in DCM (30 mL). The reaction mixture was stirred at room temperature for 3 hours, and monitored by TLC staining with 25% sulfuric acid in ethanol (developing solvent: MeOH/DCM = 1:40). Complete conversion was confirmed by TLC analysis. The reaction mixture was diluted with DCM (200 mL) and transferred to a separatory funnel. It was washed with water (100 mL) and saturated NaCl solution (100 mL). The organic layer was collected, dried over anhydrous sodium sulfate, and filtered. The filtrate was distilled under reduced pressure to remove the organic solvent, yielding the crude product. Purification by column chromatography (MeOH/DCM, from 1:200 to 1:80) gave compound **20** as a white solid (4 g, 65% yield). **^{1}H NMR (400 MHz, $CDCl_3$)** δ 7.93 (s, 1H), 7.77 – 7.75 (m, 2H), 7.62 – 7.58 (m, 2H), 7.41 (d, J = 7.3 Hz, 2H), 7.32 (td, J = 7.4, 1.2 Hz, 3H), 7.25 (d, J = 6.1 Hz, 1H), 6.86 (s, 1H), 5.85 (d, J = 7.9 Hz, 1H), 5.33 (dd, J = 10.0, 3.3 Hz, 1H), 5.30 (s, 1H), 5.28 (t, J = 1.8 Hz, 1H), 5.25 (td, J = 3.4, 1.3 Hz, 2H), 5.22 (dd, J = 3.5, 1.7 Hz, 1H), 5.08 (d, J = 9.9 Hz, 1H), 4.91 (s, 1H), 4.79 (d, J = 1.7 Hz, 1H), 4.43 (d, J = 6.7 Hz, 1H), 4.34 (t, J = 7.6 Hz, 2H), 4.27 (dd, J = 12.2, 5.1 Hz, 2H), 4.23 – 4.18 (m, 2H), 4.17 (d, J = 2.4 Hz, 2H), 4.11 (dd, J = 12.2, 2.5 Hz, 1H), 4.03 (dd, J = 7.6, 3.5 Hz, 1H), 3.90 – 3.84 (m, 1H), 3.79 – 3.75 (m, 1H), 3.67 (d, J = 2.6 Hz, 4H), 3.66 – 3.62 (m, 6H), 3.60 – 3.55 (m, 6H), 3.41 (s, 4H), 3.35 – 3.29 (m, 1H), 2.61 (s,

2H), 2.50 (t, J = 2.4 Hz, 1H), 2.43 – 2.26 (m, 6H), 2.16 (s, 3H), 2.14 (s, 3H), 2.09 (s, 3H), 2.05 (s, 3H), 2.04 (s, 3H), 1.99 (s, 6H), 1.21 (d, J = 6.3 Hz, 3H). **^{13}C{^{1}H} NMR (101 MHz, CDCl$_3$)** δ 172.89, 172.60, 172.23, 171.43, 170.75, 170.36, 170.28, 170.23, 170.06, 169.98, 169.78, 155.97, 143.90, 143.83, 141.27, 127.73, 127.11, 125.19, 119.98, 97.58, 97.49, 79.48, 77.37, 77.26, 77.06, 76.74, 75.06, 71.01, 69.92, 69.89, 69.79, 69.66, 69.24, 69.11, 69.06, 68.90, 68.43, 67.11, 67.03, 66.91, 66.43, 66.18, 62.52, 58.33, 53.48, 53.45, 52.79, 47.12, 39.48, 39.41, 39.27, 32.22, 31.97, 29.73, 28.50, 20.94, 20.82, 20.77, 20.73, 17.42. **HRMS (ESI):** *m/z* calcd for $C_{66}H_{89}N_5O_{28}Na$ $[M+Na]^+$ 1422.5586; Found 1422.5551.

(*S*)-2-amino-*N*^{5}-((*S*)-7,11-dioxo-1-(((2*S*,3*S*,4*S*,5*S*,6*R*)-3,4,5-trihydroxy-6-(hydroxymethyl)tetrahydro-2*H*-pyran-2-yl)oxy)-17-(((2*R*,3*R*,4*R*,5*R*,6*S*)-3,4,5-trihydroxy-6-methyltetrahydro-2*H*-pyran-2-yl)oxy)-3,15-dioxa-6,12-diazaheptadecan-8-yl)-*N*^{1}-(2-(2-(prop-2-yn-1-yloxy)ethoxy)ethyl)pentanediamide (3)

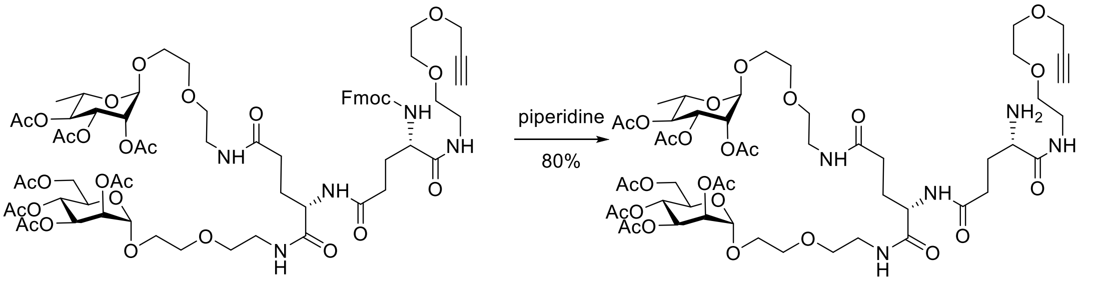

Compound **20** (4 g, 0.0028 mol) was dissolved in DMF (30 mL). Under an ice bath, 0.6 mL of piperidine was added dropwise. The reaction mixture was stirred for 10 hours. The reaction was monitored by TLC staining with 25% sulfuric acid in ethanol (developing solvent: DCM/MeOH = 10:1). Complete conversion was confirmed by TLC analysis. Glacial acetic acid and DCM were removed by vacuum distillation to give a pale-yellow crude product. Purification by column chromatography (MeOH/DCM, from 1:100 to 1:10) afforded a colorless oily compound **3** (2.8 g, 80% yield). **^{1}H NMR (400 MHz, CDCl$_3$)** δ 8.04 (s, 1H), 7.48 – 7.38 (m, 2H), 6.72 (s, 1H), 5.29 – 5.27 (m, 1H), 5.25 (t, J = 1.8 Hz, 1H), 5.21 (dtd, J = 7.7, 3.7, 1.6 Hz, 3H), 5.03

(t, J = 9.9 Hz, 1H), 4.88 (d, J = 1.7 Hz, 1H), 4.78 (d, J = 1.8 Hz, 1H), 4.33 (d, J = 5.9 Hz, 1H), 4.25 (dd, J = 12.2, 5.1 Hz, 1H), 4.15 (d, J = 2.4 Hz, 2H), 4.08 (dd, J = 12.2, 2.5 Hz, 1H), 3.99 (s, 1H), 3.86 (dd, J = 9.7, 6.3 Hz, 1H), 3.76 (ddd, J = 9.8, 4.7, 1.5 Hz, 2H), 3.66 – 3.59 (m, 14H), 3.56 – 3.50 (m, 5H), 3.41 (qd, J = 11.3, 10.1, 6.9 Hz, 5H), 3.31 (d, J = 5.6 Hz, 1H), 3.12 (s, 3H), 2.49 (t, J = 2.4 Hz, 1H), 2.32 (ddt, J = 17.4, 10.0, 5.0 Hz, 5H), 2.13 (s, 3H), 2.12 (s, 3H), 2.07 (s, 3H), 2.02 (s, 3H), 2.02 (s, 3H), 1.96 (s, 6H), 1.91 (s, 1H), 1.18 (d, J = 6.3 Hz, 3H). **$^{13}C\{^1H\}$ NMR (101 MHz, $CDCl_3$)** δ 172.89, 172.60, 172.23, 171.43, 170.75, 170.36, 170.28, 170.23, 170.06, 169.98, 169.78, 155.97, 143.90, 143.83, 141.27, 127.73, 127.11, 125.19, 119.98, 97.58, 97.49, 79.48, 77.37, 77.26, 77.06, 76.74, 75.06, 71.01, 69.92, 69.89, 69.79, 69.66, 69.24, 69.11, 69.06, 68.90, 68.43, 67.11, 67.03, 66.91, 66.43, 66.18, 62.52, 58.33, 53.48, 53.45, 52.79, 47.12, 39.48, 39.41, 39.27, 32.22, 31.97, 29.73, 28.50, 20.94, 20.82, 20.77, 20.73, 17.42. **HRMS (ESI):** *m/z* calcd for $C_{51}H_{79}N_5O_{26}$ $[M+H]^+$ 1178.5086; Found, 1178.5082.

2.2 Synthesis and Characterization of Four-Component Vaccine RMVT

Synthesis and Characterization of Tn Antigen (Compound 4)

(2*R*,3*R*,4*R*,5*R*,6*S*)-6-(((2*R*,3*S*)-3-((((9H-fluoren-9-yl)methoxy)carbonyl)amino)-4-(tert-butoxy)-4-oxobutan-2-yl)oxy)-2-(acetoxymethyl)-5-azidotetrahydro-2H-pyran-3,4-diyl diacetate (S3)

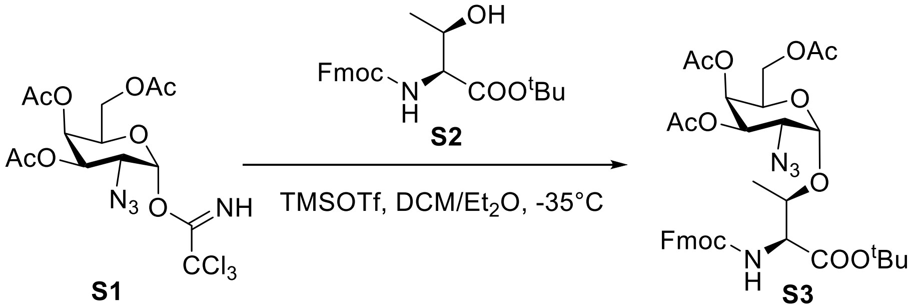

A mixture of **S1**[1] (10.0 g, 21 mmol), **S2** (4.6 g, 21 mmol) and 4 Å molecular sieves (7.0 g) was stirred in anhydrous DCM (20 mL) and Et_2O (40 mL) at rt under N_2 atmosphere for 3 h. Then, the reaction mixture was cooled to −35 °C, and TMSOTf (0.42 mL, 4.2 mmol) was added. After the complete consumption of compound 15 monitored by TLC, the reaction mixture was filtered through a pad of Celite and the residue was washed with DCM (60 mL). The filtrate was washed with saturated

aqueous $NaHCO_3$ solution, dried over anhydrous Na_2SO_4, filtered, concentrated, and used for next step without further purification.

(2*R*,3*R*,4*R*,5*R*,6*S*)-6-(((2*R*,3*S*)-3-((((9H-fluoren-9-yl)methoxy)carbonyl)amino)-4-(tert-butoxy)-4-oxobutan-2-yl)oxy)-5-acetamido-2-(acetoxymethyl)tetrahydro-2H-pyran-3,4-diyl diacetate (S4)

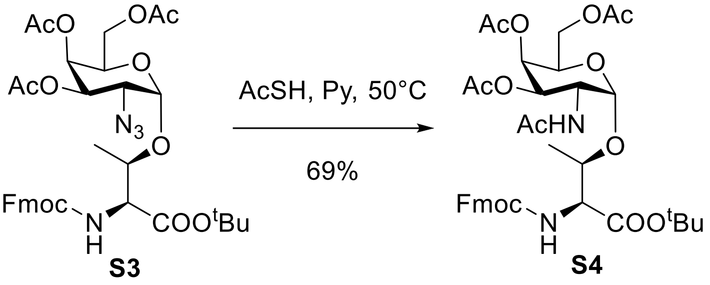

A solution of **S3** (6.0 g, 12 mmol), mercaptoacetic acid (10.0 mL) and pyridine (5.0 mL) was stirred at 50 °C for 8 h. After the complete consumption of compound **e** monitored by TLC, the reaction was quenched with saturated aqueous $NaHCO_3$ solution at 0 °C. Then, the mixture was diluted with DCM (50.0 mL), washed with saturated aqueous $NaHCO_3$ solution and brine. The organic layer was dried over anhydrous Na_2SO_4, filtered and concentrated. The residue was purified by silica gel column chromatography using DCM/MeOH (100:1) as eluent to give **S4** (6.0 g, 69%).**^{1}H NMR (600 MHz, $CDCl_3$)** δ 7.78 (d, *J* = 7.6 Hz, 2H), 7.64 (dd, *J* = 7.8, 3.1 Hz, 2H), 7.41 (td, *J* = 7.4, 4.3 Hz, 2H), 7.34 (td, *J* = 7.5, 3.6 Hz, 2H), 6.06 (d, *J* = 9.9 Hz, 1H), 5.67 (dd, *J* = 9.6, 3.3 Hz, 1H), 5.39 (d, *J* = 3.2 Hz, 1H), 5.10 (dd, *J* = 11.3, 3.2 Hz, 1H), 4.88 (d, *J* = 3.8 Hz, 1H), 4.61 (td, *J* = 10.6, 3.6 Hz, 1H), 4.51 – 4.43 (m, 2H), 4.30 – 4.19 (m, 4H), 4.12 – 4.07 (m, 2H), 2.17 (s, 3H), 2.04 (s, 3H), 2.00 (d, *J* = 8.9 Hz, 6H), 1.46 (s, 9H), 1.32 (d, *J* = 6.4 Hz, 3H). **^{13}C{^{1}H} NMR (151 MHz, $CDCl_3$)** δ 171.03, 170.41, 170.37, 170.04, 156.57, 143.83, 143.78, 141.37, 127.83, 127.80, 127.17, 125.13, 125.07, 120.08, 120.06, 100.03, 83.22, 77.27, 77.06, 76.84, 68.69, 67.47, 67.38, 67.22, 62.16, 58.97, 47.37, 47.24, 28.12, 23.25, 20.81, 20.77, 20.67, 18.65. **HRMS (ESI):** *m/z* calcd for $C_{37}H_{46}N_2O_{13}Na$ $[M+Na]^+$ 749.2892; Found, 749.2901.

(2*R*,3*R*,4*R*,5*R*,6*S*)-5-acetamido-2-(acetoxymethyl)-6-(((2*R*,3*S*)-3-amino-4-(tert-butoxy)-4-oxobutan-2-yl)oxy)tetrahydro-2H-pyran-3,4-diyl diacetate (S5)

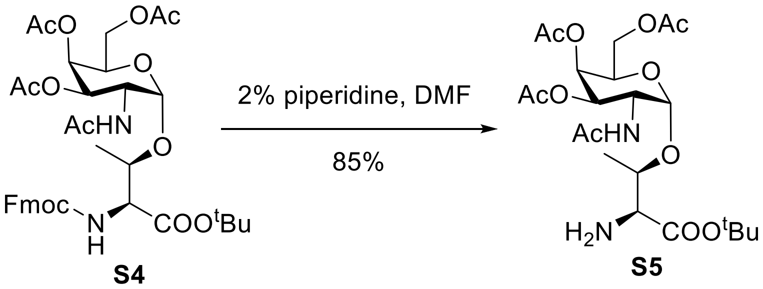

To a solution of **S4** (100.0 mg, 0.13 mmol) in anhydrous DMF (5.0 mL) was added piperidine (100 µL). After stirring at rt for 4 h, the reaction mixture was concentrated, and the residue was purified by silica gel column chromatography using DCM/MeOH (80:1) as eluent to give **S5** (59.0 mg, 85%).**^{1}H NMR (600 MHz, CDCl$_3$)** δ 6.39 (d, *J* = 9.7 Hz, 1H), 5.35 (dd, *J* = 3.2, 1.3 Hz, 1H), 5.08 (dd, *J* = 11.3, 3.2 Hz, 1H), 4.85 (d, *J* = 3.7 Hz, 1H), 4.56 (ddd, *J* = 11.3, 9.6, 3.6 Hz, 1H), 4.25 (td, *J* = 6.4, 1.4 Hz, 1H), 4.10 – 4.04 (m, 2H), 3.97 (qd, *J* = 6.4, 3.3 Hz, 1H), 3.25 (d, *J* = 3.3 Hz, 1H), 2.14 (s, 3H), 2.02 (s, 3H), 1.97 (d, *J* = 3.2 Hz, 6H), 1.44 (s, 9H), 1.35 (d, *J* = 6.4 Hz, 3H). **^{13}C{^{1}H} NMR (151 MHz, CDCl$_3$)** δ 173.61, 170.88, 170.41, 170.39, 100.13, 82.15, 78.32, 77.27, 77.06, 76.85, 69.01, 67.53, 67.31, 62.18, 59.93, 47.63, 28.10, 23.20, 20.77, 20.66, 18.64. **HRMS (ESI):** *m/z* calcd for $C_{22}H_{37}N_2O_{11}$ $[M+H]^+$ 505.2392; Found, 505.2405.

(2*R*,3*R*,4*R*,5*R*,6*S*)-5-acetamido-6-(((2*R*,3*S*)-3-acetamido-4-(tert-butoxy)-4-oxobutan-2-yl)oxy)-2-(acetoxymethyl)tetrahydro-2*H*-pyran-3,4-diyl diacetate (S6)

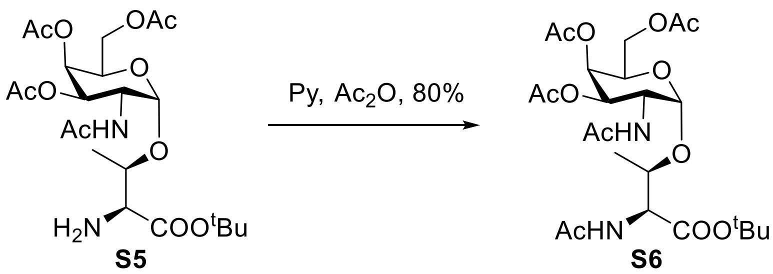

A mixture of **S5** (60.0 mg, 0.12 mmol), acetic anhydride (1.0 mL) and pyridine (2.0 mL) was stirred at rt for 2 h. After the complete consumption of compound **S5** monitored by TLC, the excessive acetic anhydride and pyridine were removed in vacuo. The residue was purified by silica gel column chromatography using DCM/MeOH (80:1) as eluent

to give **S6** (51 mg, 80%).**^{1}H NMR (600 MHz, CDCl$_3$)** δ 6.38 (dt, *J* = 9.9, 4.7 Hz, 1H), 6.04 (dt, *J* = 9.9, 2.5 Hz, 1H), 5.34 (dt, *J* = 3.2, 1.5 Hz, 1H), 5.09 (ddd, *J* = 11.3, 3.3, 1.4 Hz, 1H), 4.88 – 4.84 (m, 1H), 4.58 (ddt, *J* = 16.8, 9.2, 1.9 Hz, 2H), 4.21 – 4.15 (m, 2H), 4.10 – 4.02 (m, 2H), 2.14 (d, *J* = 1.7 Hz, 3H), 2.10 (d, *J* = 2.0 Hz, 3H), 2.01 (d, *J* = 1.6 Hz, 3H), 1.97 (t, *J* = 1.9 Hz, 6H), 1.43 (d, *J* = 1.8 Hz, 9H), 1.28 (dd, *J* = 6.4, 1.7 Hz, 3H). **^{13}C{^{1}H} NMR (151 MHz, CDCl$_3$)** δ 171.11, 170.55, 170.42, 170.34, 170.30, 170.19, 100.15, 83.20, 77.44, 77.29, 77.08, 76.86, 68.76, 67.45, 67.36, 62.19, 56.97, 47.35, 28.09, 23.22, 20.77, 20.73, 20.64, 18.68. **HRMS (ESI):** *m/z* calcd for $C_{24}H_{38}N_2O_{12}Na$ [M+Na]$^+$ 569.2317; Found, 569.2344.

O-((2*S*,3*R*,4*R*,5*R*,6*R*)-3-acetamido-4,5-diacetoxy-6-(acetoxymethyl)tetrahydro-2*H*-pyran-2-yl)-*N*-acetyl-L-threonine (4)

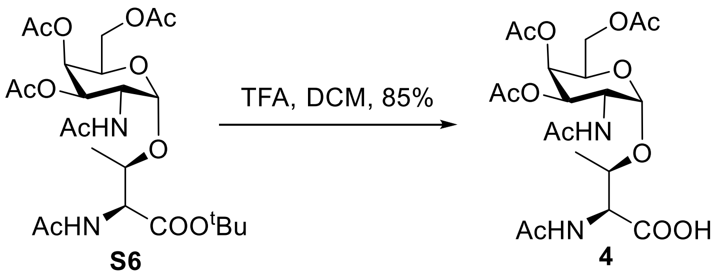

Compound **S6** (51.9 mg, 95 μmol) was dissolved in 0.75 mL DCM. Under an ice bath, 0.25 mL TFA was added dropwise. The reaction was stirred for 4 h and monitored by TLC staining with 25% sulfuric acid in ethanol (developing solvent: DCM/MeOH = 20:1). Complete conversion was confirmed by TLC analysis. Glacial acetic acid and DCM were removed by vacuum distillation to obtain a pale-yellow crude product. Further purification by silica gel column chromatography using DCM/MeOH (60:1) afforded a colorless oily compound **4** (39.6 g, 85%). **^{1}H NMR (600 MHz, CD$_3$OD)** δ 5.39 (dd, *J* = 3.3, 1.3 Hz, 1H), 5.14 (dd, *J* = 11.5, 3.3 Hz, 1H), 5.06 (d, *J* = 3.8 Hz, 1H), 4.43 (d, *J* = 5.8 Hz, 2H), 4.39 (dd, *J* = 11.5, 3.7 Hz, 1H), 4.35 (td, *J* = 6.4, 1.3 Hz, 1H), 4.14 – 4.07 (m, 2H), 2.14 (s, 3H), 2.09 (s, 3H), 2.01 (d, *J* = 6.8 Hz, 6H), 1.95 (s, 3H), 1.27 (d, *J* = 6.5 Hz, 3H). **^{13}C{^{1}H} NMR (151 MHz, CD$_3$OD)** δ 172.78, 172.08, 170.77, 170.73, 170.57, 98.85, 76.28, 68.57, 67.44, 66.74, 61.88, 57.82, 48.03, 47.89, 47.75, 47.61, 47.47, 47.33, 47.18, 21.63, 21.24, 19.24, 19.16, 19.13, 17.74. **HRMS (ESI):** *m/z* calcd for $C_{20}H_{29}N_2O_{12}$ [M-H]$^-$ 489.1726; Found, 489.1726.

(2*S*,3*S*,4*S*,5*R*,6*R*)-2-(((8*S*,13*S*)-13-(2-acetamido-3-(((2*R*,3*S*,4*S*,5*S*,6*S*)-3-acetamido-4,5-diacetoxy-6-(acetoxymethyl)tetrahydro-2*H*-pyran-2-yl)oxy)butanamido)-7,10,14-trioxo-8-(3-oxo-3-((2-(2-(((2*R*,3*R*,4*R*,5*S*,6*S*)-3,4,5-triacetoxy-6-methyltetrahydro-2*H*-pyran-2-yl)oxy)ethoxy)ethyl)amino)propyl)-3,18,21-trioxa-6,9,15-triazatetracos-23-yn-1-yl)oxy)-6-(acetoxymethyl)tetrahydro-2*H*-pyran-3,4,5-triyl triacetate (21)

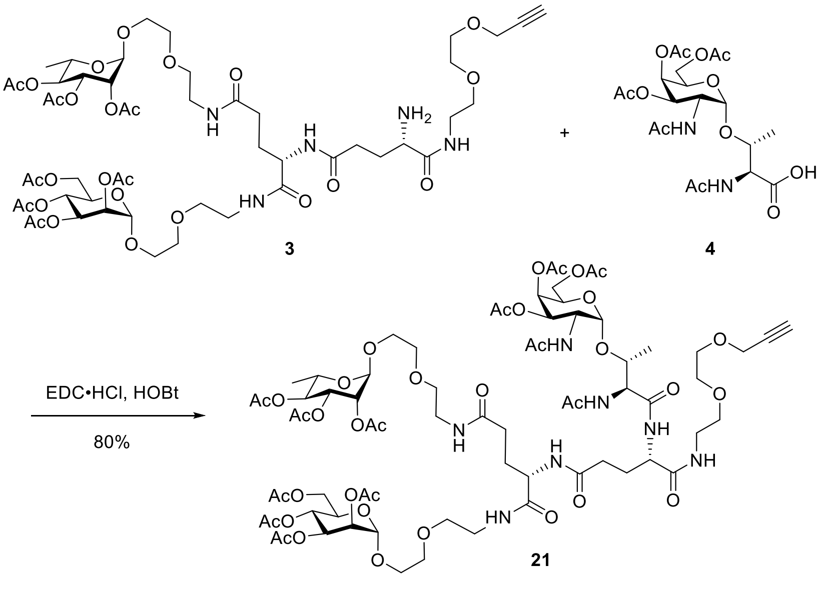

Compound **3** (150 mg, 0.1273 mmol), compound **4** (62 mg, 0.1273 mmol), EDC•HCl (121 mg, 0.6365 mmol), and HOBt (30 mg, 0.00127 mmol) were dissolved in DCM (10 mL). The reaction mixture was stirred at room temperature for 3 hours and monitored by TLC staining with 25% sulfuric acid in ethanol (developing solvent: MeOH/DCM = 1:40). Complete conversion was confirmed by TLC analysis. Then the reaction was diluted with DCM (100 mL) and transferred to a separatory funnel. Washed sequentially with water (50 mL) and saturated NaCl solution (50 mL). The organic layer was collected, dried over anhydrous sodium sulfate, and filtered. The filtrate was distilled under reduced pressure to remove the organic solvent, yielding the crude product. Purification by column chromatography (DCM/MeOH, from 200:1 to 80:1) afforded compound **21** as a white solid (100 mg, 80% yield). **^{1}H NMR (400 MHz, $CDCl_3$)** δ 8.14 (s, 1H), 7.67 (d, J = 7.2 Hz, 1H), 7.37 (d, J = 7.5 Hz, 1H), 7.23 (s, 1H), 6.86 (s, 2H), 5.41 (d, J = 3.9 Hz, 1H), 5.36 – 5.20 (m, 5H), 5.20 – 5.12 (m, 2H), 5.07 (t, J = 9.9 Hz, 1H), 4.92 (d, J = 1.8 Hz, 1H), 4.82 (d, J = 1.7 Hz, 1H), 4.65 – 4.55 (m, 1H),

4.51 (dd, J = 7.5, 3.0 Hz, 1H), 4.46 – 4.24 (m, 5H), 4.19 (t, J = 2.7 Hz, 2H), 4.16 – 4.00 (m, 4H), 3.89 (dd, J = 9.8, 6.3 Hz, 1H), 3.84 – 3.75 (m, 2H), 3.74 – 3.25 (m, 24H), 2.54 (t, J = 2.4 Hz, 1H), 2.37 (dt, J = 14.5, 7.7 Hz, 4H), 2.24 (d, J = 8.1 Hz, 2H), 2.17 (s, 3H), 2.16 (d, J = 1.9 Hz, 3H), 2.15 (s, 3H), 2.11 (s, 3H), 2.09 (s, 3H), 2.06 (s, 3H), 2.05 (s, 3H), 2.04 (s, 3H), 2.00 (s, 3H), 2.00 (s, 3H), 1.98 (s, 3H), 1.97 (s, 1H), 1.96 (s, 3H), 1.22 (dd, J = 6.5, 2.3 Hz, 6H). **$^{13}C\{^{1}H\}$ N MR (101 MHz, $CDCl_3$)** δ 172.76, 172.66, 172.18, 171.08, 170.71, 170.53, 170.48, 170.45, 170.28, 170.20, 170.05, 169.92, 169.72, 99.15, 97.56, 97.42, 79.41, 77.47, 77.37, 77.25, 77.05, 76.73, 76.66, 75.19, 70.95, 69.96, 69.94, 69.88, 69.78, 69.65, 69.08, 69.03, 68.94, 68.87, 68.44, 68.23, 67.37, 67.10, 67.08, 66.79, 66.47, 66.16, 62.51, 62.23, 58.33, 56.06, 52.88, 52.59, 47.68, 39.51, 39.43, 39.39, 32.34, 31.86, 28.73, 28.51, 23.12, 22.78, 20.96, 20.93, 20.81, 20.78, 20.75, 20.72, 20.69, 17.43, 16.60. **HRMS (ESI):** *m/z* calcd for $C_{71}H_{107}N_7O_{37}Na$ $[M+Na]^+$ 1650.6779; Found, 1650.6795.

(2*S*)-2-(2-acetamido-3-(((2*R*,3*S*,4*S*,5*S*,6*S*)-3-acetamido-4,5-dihydroxy-6-(hydroxymethyl)tetrahydro-2*H*-pyran-2-yl)oxy)butanamido)-*N*⁵-((*S*)-7,11-dioxo-1-(((2*S*,3*S*,4*S*,5*S*,6*R*)-3,4,5-trihydroxy-6-(hydroxymethyl)tetrahydro-2*H*-pyran-2-yl)oxy)-17-(((2*R*,3*R*,4*R*,5*R*,6*S*)-3,4,5-trihydroxy-6-methyltetrahydro-2*H*-pyran-2-yl)oxy)-3,15-dioxa-6,12-diazaheptadecan-8-yl)-*N*¹-(2-(2-(prop-2-yn-1-yloxy)ethoxy)ethyl)pentanediamide (1)

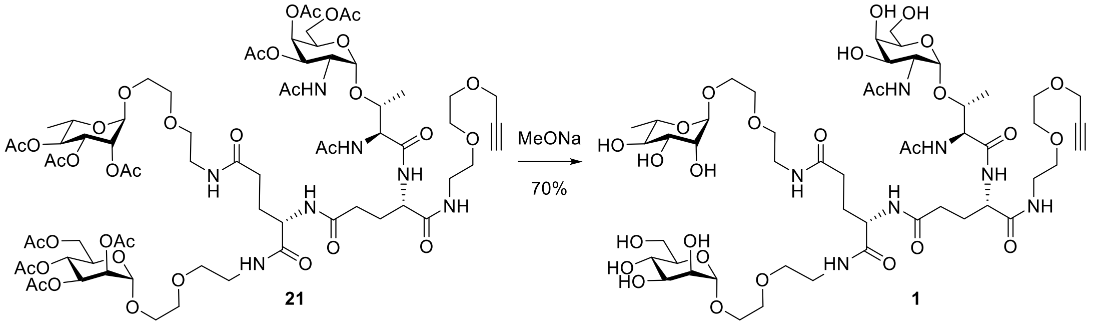

Compound **21** (100 mg, 0.0606 mmol) and MeONa (32.7 mg, 0.0606 mol) were dissolved in methanol (10 mL). The reaction was stirred at room temperature for 24 hours and monitored by TLC staining with 25% sulfuric acid in ethanol (developing solvent: DCM/MeOH/H_2O = 2:1:0.1). Complete conversion was confirmed by TLC

analysis. Acidic ion exchange resin was added into the reaction mixture and was stir until it becomes clear, pH = 7. The reaction was filtered, and the filtrate was distilled under reduced pressure to remove the organic solvent, yielding a pale-yellow crude product. The crude product was washed with DCM, and dissolved in 200 μL methanol. 8 mL dichloromethane was added to precipitate compound **1** as a white solid (52 mg, 70% yield). **^{1}H NMR (400 MHz, CD_3OD)** δ 4.71 (d, J = 1.8 Hz, 1H), 4.63 (d, J = 1.7 Hz, 1H), 4.45 (d, J = 2.6 Hz, 1H), 4.26 – 4.13 (m, 4H), 4.10 (d, J = 2.4 Hz, 2H), 3.86 – 3.78 (m, 2H), 3.77 – 3.66 (m, 7H), 3.65 – 3.42 (m, 28H), 3.41 – 3.23 (m, 9H), 2.78 (t, J = 2.4 Hz, 1H), 2.23 (dt, J = 15.2, 7.4 Hz, 4H), 2.01 (s, 3H), 1.98 (s, 1H), 1.95 (s, 3H), 1.91 – 1.78 (m, 3H), 1.18 (dt, J = 5.4, 2.7 Hz, 6H). **^{13}C{^{1}H} NMR (101 MHz, CD_3OD)** δ 173.63, 173.39, 172.94, 172.67, 172.42, 172.25, 170.73, 100.33, 100.23, 99.26, 79.20, 75.93, 74.78, 73.26, 72.60, 71.63, 71.12, 70.95, 70.81, 70.71, 69.81, 69.65, 69.18, 69.08, 68.97, 68.72, 68.41, 67.30, 66.31, 61.58, 61.37, 57.68, 57.19, 52.93, 50.14, 48.31, 48.24, 48.10, 48.03, 47.88, 47.81, 47.67, 47.60, 47.46, 47.39, 47.17, 46.96, 39.05, 31.86, 31.37, 27.89, 27.62, 21.89, 21.24, 17.62, 16.68. **HRMS (ESI):** *m/z* calcd for $C_{51}H_{87}N_7O_{27}Na$ $[M+Na]^+$ 1252.5542; Found, 1252.5556.

((2*R*,3*R*,4*S*,5*R*,6*R*)-6-(((2*R*,3*R*,4*S*,5*R*,6*R*)-5-(2-(2-(4-((10*S*,15*S*)-10-(2-acetamido-3-(((2*R*,3*S*,4*S*,5*S*,6*S*)-3-acetamido-4,5-dihydroxy-6-(hydroxymethyl)tetrahydro-2H-pyran-2-yl)oxy)butanamido)-9,13,18-trioxo-15-((2-(2-(((2*S*,3*S*,4*S*,5*S*,6*R*)-3,4,5-trihydroxy-6-(hydroxymethyl)tetrahydro-2*H*-pyran-2-yl)oxy)ethoxy)ethyl)carbamoyl)-24-(((2*R*,3*R*,4*R*,5*R*,6*S*)-3,4,5-trihydroxy-6-methyltetrahydro-2*H*-pyran-2-yl)oxy)-2,5,22-trioxa-8,14,19-triazatetracosyl)-1*H*-1,2,3-triazol-1-yl)ethoxy)ethoxy)-3,4-bis(benzyloxy)-6-(((3-nonyldodecanoyl)oxy)methyl)tetrahydro-2H-pyran-2-yl)oxy)-3,4,5-tris(benzyloxy)tetrahydro-2*H*-pyran-2-yl)methyl 3-nonyldodecanoate (22)

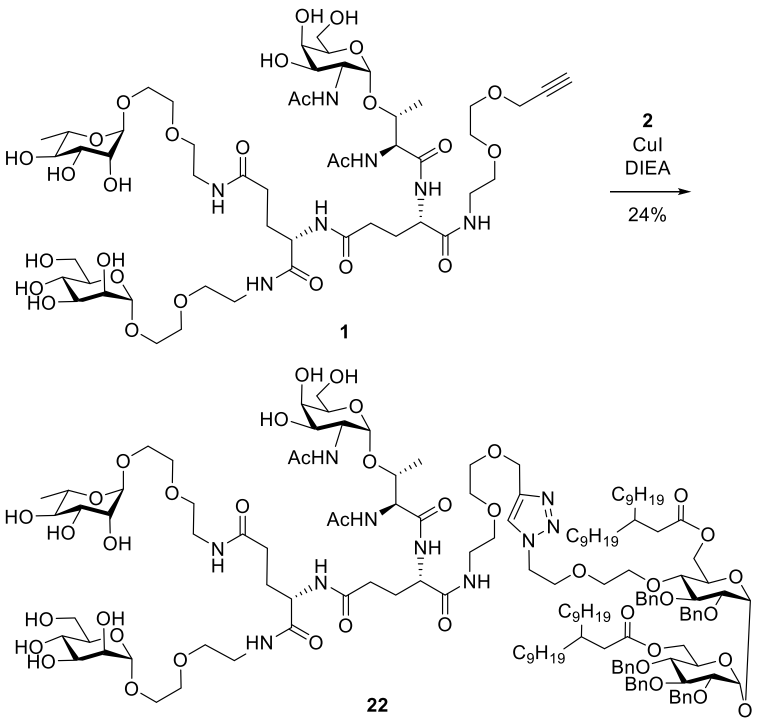

Compound **1** (42.0 mg, 0.0422 mmol) was dissolve in a 4 mL mixture of tetrahydrofuran and methanol. Then, compound **2** (64.0 mg, 0.0422 mmol), copper(I) iodide (80.0 mg, 0.844 mmol), and N,N-diisopropylethylamine (137 μL, 0.844 mmol) were added. The mixture was stirred at room temperature for 12 hours and monitored by TLC staining with 25% sulfuric acid in ethanol (developing solvent: MeOH/DCM/H_2O = 1:4:0.1). Complete conversion was confirmed by TLC analysis. Diatomaceous earth was used to remove insoluble, and then the solvent was removed from the filtrate by vacuum distillation to afford the crude product. Purification by column chromatography (DCM/MeOH, from 10:1 to 4:1) afforded compound **22** as a white solid (23 mg, 24% yield). **^{1}H NMR (400 MHz, CD_3OD)** δ 7.81 (s, 1H), 7.35 – 7.03 (m, 25H), 5.15 (dd, J = 6.8, 3.5 Hz, 2H), 4.87 (d, J = 11.1 Hz, 1H), 4.71 – 4.67 (m, 3H), 4.62 (d, J = 4.4 Hz, 4H), 4.52 – 4.38 (m, 7H), 4.27 – 4.12 (m, 6H), 4.01 (dt, J = 20.7, 10.5 Hz, 7H), 3.92 – 3.70 (m, 12H), 3.70 – 3.35 (m, 33H), 3.33 – 3.24 (m, 7H), 2.20 (s, 2H), 2.12 (d, J = 6.8 Hz, 2H), 2.08 (d, J = 6.8 Hz, 2H), 1.98 (s, 3H), 1.94 (d, J = 3.3 Hz, 2H), 1.93 (d, J = 2.3 Hz, 3H), 1.82 (dd, J = 14.2, 7.5 Hz, 2H), 1.69 (s, 2H), 1.55 – 0.88 (m, 70H), 0.84 – 0.72 (m, 12H). **HRMS (ESI):** *m/z* calcd for $C_{144}H_{226}N_{10}O_{41}Na$ $[M+Na]^+$ 2774.5977; Found, 2774.5924.

((2*R*,3*S*,4*S*,5*R*,6*R*)-6-(((2*R*,3*R*,4*R*,5*S*,6*R*)-5-(2-(2-(4-((10*S*,15*S*)-10-(2-acetamido-3-(((2*R*,3*S*,4*S*,5*S*,6*S*)-3-acetamido-4,5-dihydroxy-6-(hydroxymethyl)tetrahydro-2*H*-pyran-2-yl)oxy)butanamido)-9,13,18-trioxo-15-((2-(2-(((2*S*,3*S*,4*S*,5*S*,6*R*)-3,4,5-trihydroxy-6-(hydroxymethyl)tetrahydro-2*H*-pyran-2-yl)oxy)ethoxy)ethyl)carbamoyl)-24-(((2*R*,3*R*,4*R*,5*R*,6*S*)-3,4,5-trihydroxy-6-methyltetrahydro-2*H*-pyran-2-yl)oxy)-2,5,22-trioxa-8,14,19-triazatetracosyl)-1*H*-1,2,3-triazol-1-yl)ethoxy)ethoxy)-3,4-dihydroxy-6-(((3-nonyldodecanoyl)oxy)methyl)tetrahydro-2*H*-pyran-2-yl)oxy)-3,4,5-trihydroxytetrahydro-2*H*-pyran-2-yl)methyl 3-nonyldodecanoate (RMVT)

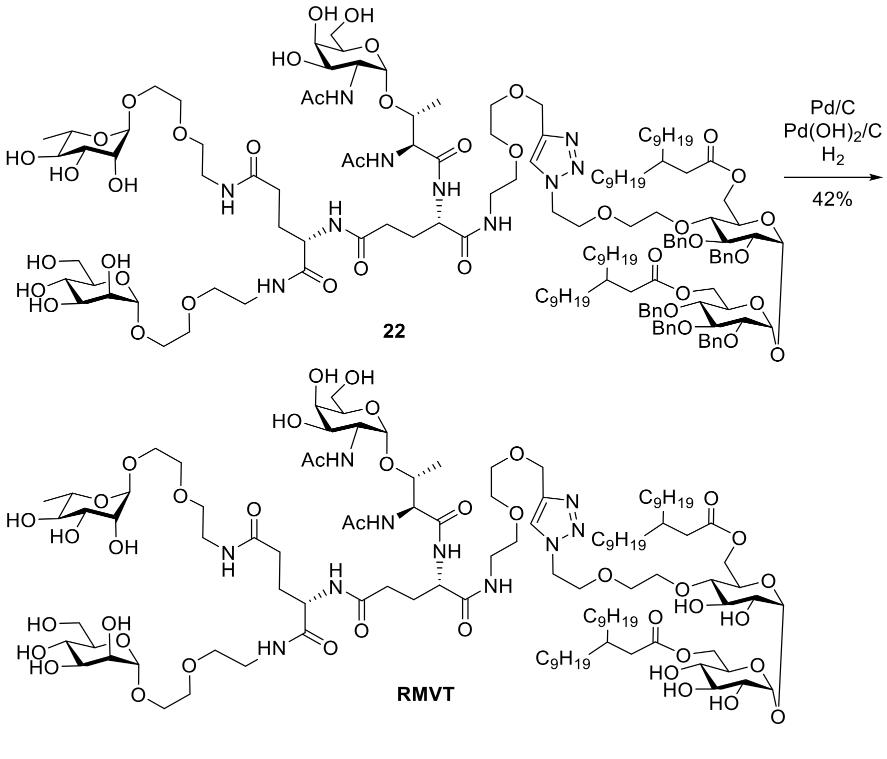

Compound **22** (8.0 mg, 2.0 μmol) was dissolved in 10 mL of a mixed solvent of DCM/MeOH/H_2O (4:2:1). Pd/C (5.0 mg) and $Pd(OH)_2/C$ (5.0 mg) were added. Hydrogen balloon was used for the hydrogenation, and the reaction was stirred for 24 hours. Diatomaceous earth was used to remove the insoluble, and washed with 30 mL of DCM/MeOH/H_2O (5:5:1) and the filtrate was distilled under reduced pressure to remove the solvent, the residue was purified by gel filtration chromatography on Sephadex LH-20 (MeOH) to afford compound **RMVT** as a white solid (3.0 mg, 42%

yield). **^{1}H NMR (400 MHz, CD_3OD)** δ 7.94 (s, 1H), 4.95 (d, J = 3.9 Hz, 2H), 4.63 (s, 3H), 4.57 (s, 4H), 4.49 (d, J = 7.5 Hz, 9H), 4.24 (d, J = 12.7 Hz, 9H), 4.09 (s, 6H), 3.92 (s, 12H), 3.76 (d, J = 25.8 Hz, 18H), 3.64 – 3.41 (m, 32H), 3.38 (p, J = 1.7 Hz, 8H), 3.28 (s, 6H), 2.18 (d, J = 6.3 Hz, 4H), 2.00 (s, 3H), 1.94 (s, 3H), 1.75 (s, 2H), 1.20 (s, 70H), 0.80 (t, J = 6.7 Hz, 12H). **HRMS (ESI):** *m/z* calcd for $C_{109}H_{196}N_{10}O_{41}Na$ $[M+Na]^+$ 2324.3452; Found, 2324.3466.

2.3 Synthesis and Characterization of Three-Component Vaccine RVT

(2*R*,3*R*,4*R*,5*S*,6*S*)-2-(((*S*)-10-((((9*H*-fluoren-9-yl)methoxy)carbonyl)amino)-7,11-dioxo-3,15,18-trioxa-6,12-diazahenicos-20-yn-1-yl)oxy)-6-methyltetrahydro-2*H*-pyran-3,4,5-triyl triacetate (23)

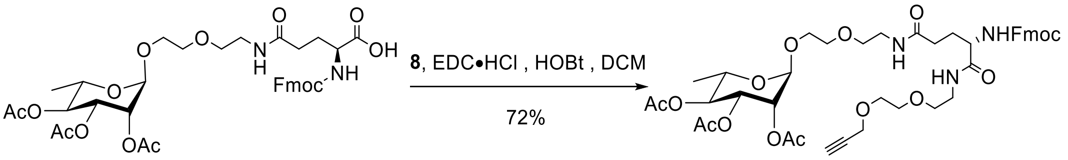

15 **23**

Compound **15** (500 mg, 0.6868 mmol), compound **8** (102 mg, 0.8204 mmol), EDC•HCl (400 mg, 3.434 mmol), and HOBt (83 mg, 0.0687 mmol) were dissolved in DCM (10 mL). The reaction mixture was stirred at room temperature for 3 hours and monitored by TLC staining with 25% sulfuric acid in ethanol (developing solvent: MeOH/DCM = 1:40). Complete conversion was confirmed by TLC analysis. Then the reaction was diluted with DCM (100 mL) and transferred to a separatory funnel. Washed sequentially with water (50 mL) and saturated NaCl solution (50 mL). The organic layer was collected, dried over anhydrous sodium sulfate, and filtered. The filtrate was distilled under reduced pressure to remove the organic solvent, yielding the crude product. Purification by column chromatography (DCM/MeOH, from 200:1 to 80:1) afforded compound **23** as a white viscous solid (410 mg, 72% yield). **^{1}H NMR (400 MHz, $CDCl_3$)** δ 7.77 – 7.74 (m, 2H), 7.61 (d, J = 7.5 Hz, 2H), 7.40 (d, J = 7.4 Hz, 2H), 7.33 – 7.29 (m, 2H), 7.17 (s, 1H), 6.64 (s, 1H), 6.22 (d, J = 7.4 Hz, 1H), 5.29 (dd, J = 10.1, 3.5 Hz, 1H), 5.23 (dd, J = 3.5, 1.7 Hz, 1H), 5.07 (t, J = 9.9 Hz, 1H), 4.82 (d, J = 1.8 Hz, 1H), 4.35 (dd, J = 7.4, 3.0 Hz, 2H), 4.21 (t, J = 7.2 Hz, 2H), 4.16 (d, J = 2.4 Hz, 2H),

3.92 – 3.86 (m, 1H), 3.80 – 3.74 (m, 1H), 3.66 – 3.62 (m, 8H), 3.59 – 3.56 (m, 3H), 3.55 (s, 1H), 3.46 (d, J = 5.2 Hz, 3H), 2.46 (t, J = 2.4 Hz, 1H), 2.39 – 2.29 (m, 3H), 2.14 (s, 3H), 2.07 (s, 1H), 2.03 (s, 3H), 1.99 (s, 3H), 1.20 (d, J = 6.2 Hz, 3H). **^{13}C{^{1}H} NMR (101 MHz, CDCl3)** *δ* 172.91, 171.52, 170.40, 170.29, 169.98, 156.31, 156.29, 143.91, 143.82, 141.28, 127.72, 127.09, 125.20, 119.97, 97.52, 79.48, 77.42, 77.09, 76.77, 74.92, 71.00, 70.03, 69.98, 69.94, 69.85, 69.54, 69.11, 68.99, 66.98, 66.45, 58.34, 47.15, 39.38, 39.31, 20.95, 20.80, 20.76, 17.42. **HRMS (ESI):** *m/z* calcd for $C_{43}H_{55}N_3O_{15}Na$ $[M+Na]^+$ 876.3525; Found, 876.3563.

(2*R*,3*R*,4*R*,5*S*,6*S*)-2-(((*S*)-10-amino-7,11-dioxo-3,15,18-trioxa-6,12-diazahenicos-20-yn-1-yl)oxy)-6-methyltetrahydro-2*H*-pyran-3,4,5-triyl triacetate (24)

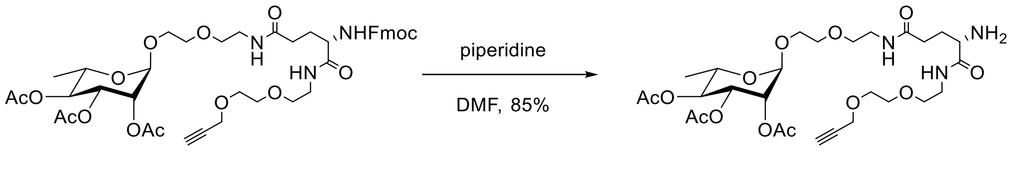

Compound **23** (400 mg, 0.4689 mmol) was dissolved in 10 mL of DMF. Under an ice bath, 0.2 mL of piperidine was added dropwise, and the reaction mixture reacted for 10 hours. The reaction was monitored by TLC staining with 25% sulfuric acid in ethanol (developing solvent: DCM/MeOH = 10:1). Complete conversion was confirmed by TLC analysis. DMF and piperidine were removed by vacuum distillation to give a pale-yellow crude product. Purification by column chromatography (DCM/MeOH, from 90:1 to 10:1) afforded a colorless, transparent, viscous liquid compound **24** (250 mg, 85% yield). **^{1}H NMR (400 MHz, CDCl3)** *δ* 7.61 (t, *J* = 5.8 Hz, 1H), 6.70 (t, *J* = 5.7 Hz, 1H), 5.33 – 5.17 (m, 2H), 5.07 (t, *J* = 9.9 Hz, 1H), 4.82 (d, *J* = 1.8 Hz, 1H), 4.20 (d, *J* = 2.4 Hz, 2H), 3.99 – 3.87 (m, 1H), 3.79 (ddt, *J* = 8.6, 6.4, 2.9 Hz, 1H), 3.73 – 3.54 (m, 11H), 3.50 – 3.35 (m, 5H), 2.48 (t, *J* = 2.4 Hz, 1H), 2.33 (p, *J* = 7.2 Hz, 2H), 2.16 (s, 3H), 2.06 (s, 3H), 2.05 – 2.01 (m, 1H), 2.00 (s, 3H), 1.96 – 1.88 (m, 1H), 1.23 (d, *J* = 6.3 Hz, 3H). **^{13}C{^{1}H} NMR (101 MHz, CDCl3)** *δ* 174.92, 173.02, 170.32, 170.16, 169.99, 97.51, 79.49, 77.46, 77.39, 77.07, 76.75, 76.66, 74.80, 71.01, 70.06, 70.02, 69.94, 69.91, 69.84, 69.73, 69.06, 68.98, 68.95, 66.98, 66.45, 58.38, 54.52, 39.23, 38.90,

32.73, 31.12, 20.95, 20.82, 20.75, 17.42. **HRMS (ESI):** *m/z* calcd for $C_{28}H_{46}N_3O_{13}$ $[M+H]^+$ 632.3025; found, 632.3039.

(2*R*,3*R*,4*R*,5*S*,6*S*)-2-(((10*R*)-10-(2-acetamido-3-(((2*R*,3*S*,4*S*,5*S*,6*S*)-3-acetamido-4,5-diacetoxy-6-(acetoxymethyl)tetrahydro-2*H*-pyran-2-yl)oxy)butanamido)-7,11-dioxo-3,15,18-trioxa-6,12-diazahenicos-20-yn-1-yl)oxy)-6-methyltetrahydro-2*H*-pyran-3,4,5-triyl triacetate (25)

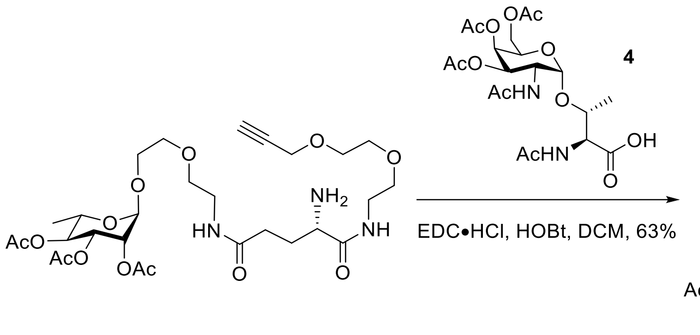

Compound **24** (100.0 mg, 0.1580 mmol), compound **4** (77.6 mg, 0.1580 mmol), EDC•HCl (120.0 mg, 0.6320 mmol), and HOBt (22.4 mg, 0.01580 mmol) were dissolved in DCM (10 mL). The reaction mixture was stirred at room temperature for 3 hours and monitored TLC (developing solvent: MeOH/DCM = 1:40). Complete conversion was confirmed by TLC analysis. Then the reaction was diluted with DCM (100 mL) and transferred to a separatory funnel. Washed sequentially with water (50 mL) and saturated NaCl solution (50 mL). The organic layer was collected, dried over anhydrous sodium sulfate, and filtered. Then the filtrate was distilled under reduced pressure to remove the organic solvent, yielding the crude product. Purification by column chromatography (DCM/MeOH, from 200:1 to 80:1) afforded compound **25** as a white viscous solid (110 mg, 63% yield). **^{1}H NMR (400 MHz, $CDCl_3$)** δ 8.09 (d, J = 6.7 Hz, 1H), 7.36 (t, J = 5.5 Hz, 1H), 7.06 (d, J = 9.0 Hz, 1H), 6.84 – 6.72 (m, 2H), 5.40 (dd, J = 3.2, 1.3 Hz, 1H), 5.28 (dd, J = 10.1, 3.5 Hz, 1H), 5.22 (dd, J = 3.5, 1.7 Hz, 1H), 5.17 (dd, J = 11.6, 3.2 Hz, 1H), 5.12 – 5.03 (m, 2H), 4.83 (d, J = 1.8 Hz, 1H), 4.56 (ddd, J = 15.3, 8.3, 3.3 Hz, 2H), 4.37 (q, J = 6.3 Hz, 1H), 4.32 – 4.23 (m, 2H), 4.20 (d, J = 2.4 Hz, 2H), 4.08 (qd, J = 11.4, 6.5 Hz, 2H), 3.95 – 3.85 (m, 1H), 3.79 (ddd, J = 9.9, 5.2, 3.2 Hz, 1H), 3.72 – 3.52 (m, 11H), 3.44 (hept, J = 5.5, 4.8 Hz, 4H), 2.51 (t, J = 2.3

Hz, 1H), 2.48 – 2.32 (m, 2H), 2.16 (s, 3H), 2.15 (s, 3H), 2.11 (s, 3H), 2.09 – 2.06 (m, 2H), 2.06 (s, 3H), 2.03 (s, 3H), 2.00 (s, 3H), 1.98 (s, 3H), 1.97 (d, *J* = 1.6 Hz, 3H), 1.24 (d, *J* = 4.5 Hz, 3H), 1.22 (d, *J* = 4.3 Hz, 3H). **$^{13}C\{^1H\}$ NMR (101 MHz, $CDCl_3$)** *δ* 173.32, 171.11, 171.05, 170.72, 170.66, 170.50, 170.45, 170.43, 170.29, 169.95, 169.47, 99.34, 97.48, 79.45, 77.39, 77.27, 77.07, 76.75, 76.23, 74.99, 70.98, 70.05, 70.01, 69.97, 69.72, 69.36, 69.07, 68.32, 67.37, 67.10, 66.94, 66.47, 62.25, 58.37, 56.62, 53.20, 47.63, 39.52, 39.36, 31.92, 28.15, 23.18, 22.91, 20.97, 20.81, 20.78, 20.76, 20.74, 20.68, 17.42, 17.03. **HRMS (ESI):** *m/z* calcd for $C_{48}H_{73}N_5O_{25}Na$ $[M+Na]^+$ 1126.4538; Found, 1126.4580.

(2*R*)-2-(2-acetamido-3-(((2*R*,3*S*,4*S*,5*S*,6*S*)-3-acetamido-4,5-dihydroxy-6-(hydroxymethyl)tetrahydro-2*H*-pyran-2-yl)oxy)butanamido)-*N*^{1}-(2-(2-(prop-2-yn-1-yloxy)ethoxy)ethyl)-*N*^{5}-(2-(2-(((2*R*,3*R*,4*R*,5*R*,6*S*)-3,4,5-trihydroxy-6-methyltetrahydro-2*H*-pyran-2-yl)oxy)ethoxy)ethyl)pentanediamide (26)

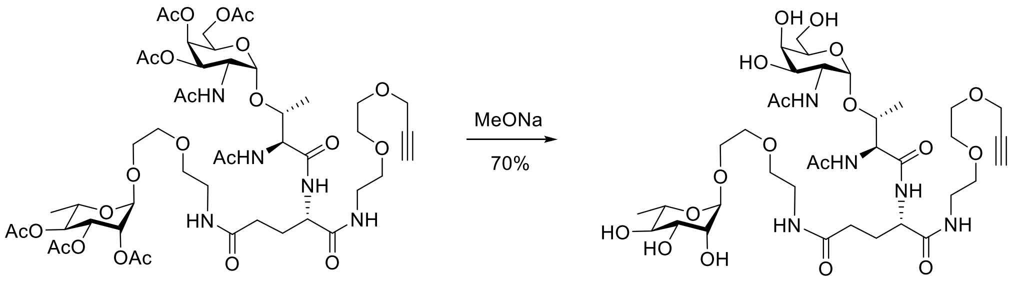

Compound **25** (100 mg, 0.0906 mmol) and MeONa (48.9 mg, 0.09060 mol) were dissolved in methanol (10 mL). The reaction was stirred at room temperature for 24 hours and monitored by TLC staining with 25% sulfuric acid in ethanol (developing solvent: DCM/MeOH/H_2O = 2:1:0.1). Complete conversation was confirmed by TLC staining with 25% sulfuric acid in ethanol. Acidic ion exchange resin was added into the reaction mixture and was stir until it becomes clear, pH = 7. The reaction was filtered, and the filtrate was distilled under reduced pressure to remove the organic solvent, yielding a pale-yellow crude product. The crude product was washed with DCM, and dissolved in 200 μL methanol. 8 mL dichloromethane was added to precipitate crystals, yielding compound **26** as a white solid (54.0 mg, 70% yield). **^1H NMR (400 MHz, CD_3OD)** *δ* 4.72 (d, *J* = 1.7 Hz, 1H), 4.53 (d, *J* = 2.6 Hz, 1H), 4.32 –

4.21 (m, 3H), 4.19 (d, *J* = 2.4 Hz, 2H), 3.90 (q, *J* = 5.4, 4.2 Hz, 2H), 3.85 – 3.74 (m, 3H), 3.72 (dd, *J* = 5.9, 1.8 Hz, 2H), 3.69 – 3.58 (m, 10H), 3.57 – 3.52 (m, 4H), 3.43 (dd, *J* = 13.7, 8.3 Hz, 1H), 3.39 – 3.34 (m, 3H), 2.87 (t, *J* = 2.4 Hz, 1H), 2.29 (t, *J* = 7.5 Hz, 2H), 2.11 (s, 3H), 2.04 (s, 3H), 2.02 – 1.86 (m, 2H), 1.28 (d, *J* = 6.1 Hz, 3H), 1.25 (s, 3H). **$^{13}C\{^{1}H\}$ NMR (101 MHz, CD_3OD)** δ 174.92, 174.39, 173.88, 173.77, 172.25, 101.77, 100.61, 80.60, 77.21, 76.18, 74.04, 73.04, 72.37, 72.24, 71.26, 71.10, 70.62, 70.47, 70.42, 70.18, 70.00, 69.84, 67.75, 62.81, 59.12, 58.63, 54.70, 51.54, 49.68, 49.54, 49.47, 49.33, 49.26, 49.11, 49.04, 48.90, 48.83, 48.62, 48.40, 40.54, 40.48, 33.11, 29.11, 23.28, 22.67, 19.02, 18.11. **HRMS (ESI):** *m/z* calcd for $C_{48}H_{73}N_5O_{25}Na$ $[M+Na]^+$ 874.3904; Found, 874.3921.

((2*R*,3*R*,4*S*,5*R*,6*R*)-6-(((2*R*,3*R*,4*S*,5*R*,6*R*)-5-(2-(2-(4-((10*R*)-10-(2-acetamido-3-(((2*R*,3*S*,4*S*,5*S*,6*S*)-3-acetamido-4,5-dihydroxy-6-(hydroxymethyl)tetrahydro-2*H*-pyran-2-yl)oxy)butanamido)-9,13-dioxo-19-(((2*R*,3*R*,4*R*,5*R*,6*S*)-3,4,5-trihydroxy-6-methyltetrahydro-2*H*-pyran-2-yl)oxy)-2,5,17-trioxa-8,14-diazanonadecyl)-1*H*-1,2,3-triazol-1-yl)ethoxy)ethoxy)-3,4-bis(benzyloxy)-6-(((3-nonyldodecanoyl)oxy)methyl)tetrahydro-2*H*-pyran-2-yl)oxy)-3,4,5-tris(benzyloxy)tetrahydro-2*H*-pyran-2-yl)methyl 3-nonyldodecanoate (27)

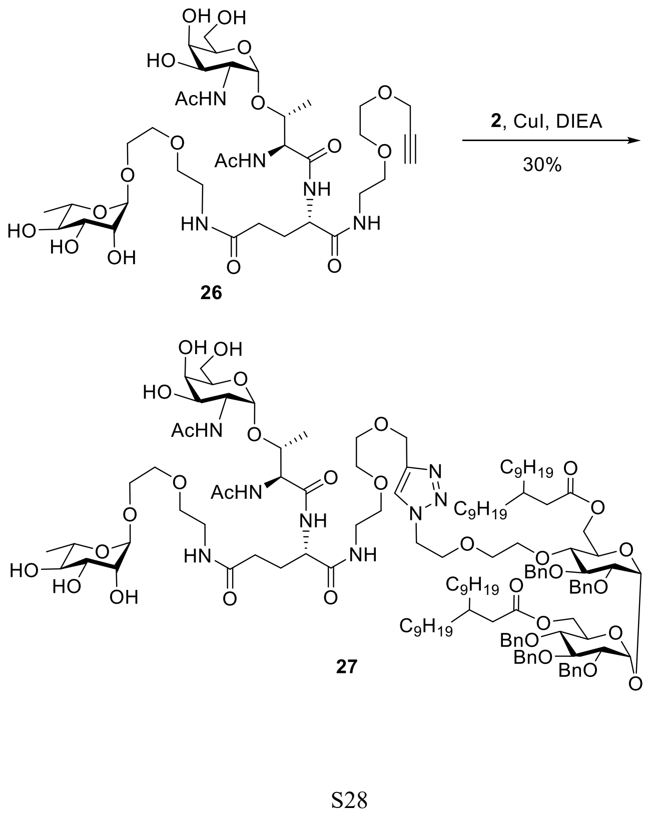

A 4 mL mixture of THF and MeOH (1:1) was used to dissolve compound **26** (30.0 mg, 0.03525 mmol), compound **2** (53.6 mg, 0.03525 mmol) and copper(I) iodide (133 mg, 0.7050 mmol), followed by addition of N,N-diisopropylethylamine (118 μL, 0.7050 mmol). The mixture was stirred at room temperature for 12 hours and monitored by TLC staining with 25% sulfuric acid in ethanol (developing solvent: MeOH/DCM/H_2O = 1:4:0.1). Complete conversion was confirmed by TLC analysis. Diatomaceous earth was used to remove the insoluble, and then the solvent was removed from the filtrate by vacuum distillation to afford the crude product. Purification by column chromatography (DCM/MeOH, from 10:1 to 4:1) afforded compound **27** as a white solid (25 mg, 30% yield). **^{1}H NMR (400 MHz, $CD_3OD/CDCl_3$)** δ 7.90 (d, J = 1.5 Hz, 1H), 7.38 – 7.21 (m, 25H), 5.24 (dd, J = 6.8, 3.5 Hz, 2H), 4.97 (d, J = 10.8 Hz, 1H), 4.81 – 4.75 (m, 2H), 4.72 (d, J = 5.0 Hz, 4H), 4.59 – 4.46 (m, 6H), 4.25 (ddt, J = 14.7, 11.0, 3.7 Hz, 4H), 4.19 – 4.04 (m, 6H), 4.03 – 3.84 (m, 6H), 3.83 – 3.43 (m, 28H), 3.41 – 3.34 (m, 3H), 2.26 (d, J = 4.2 Hz, 2H), 2.22 (d, J = 6.8 Hz, 2H), 2.17 (d, J = 6.8 Hz, 2H), 2.08 (s, 3H), 2.03 (s, 1H), 2.01 (s, 3H), 1.92 (dd, J = 14.4, 7.5 Hz, 1H), 1.78 (s, 2H), 1.32 – 1.20 (m, 70H), 0.88 (td, J = 6.1, 5.7, 3.1 Hz, 12H). **^{13}C{^{1}H} NMR (101 MHz, $CD_3OD/CDCl_3$)** δ 173.51 173.46, 173.41, 172.96, 172.36, 172.27, 170.78, 138.65, 138.13, 137.97, 137.91, 128.21, 128.18, 128.06, 128.04, 127.72, 127.69, 127.60, 127.49, 127.31, 127.29, 124.50, 100.31, 99.18, 92.75, 80.94, 79.64, 78.17, 77.84, 77.52, 74.59, 73.08, 72.88, 72.63, 71.61, 70.99, 70.79, 69.84, 69.39, 69.21, 69.03, 68.40, 66.30, 63.59, 61.39, 57.16, 50.03, 48.35, 48.21, 48.14, 48.00, 47.93, 47.78, 47.71, 47.57, 47.50, 47.29, 47.07, 39.12, 38.83, 35.01, 34.97, 33.53, 31.73, 29.61, 29.43, 29.41, 29.39, 29.15, 26.30, 26.25, 22.41, 21.90, 21.37, 17.59, 16.79, 13.24, 13.23. **HRMS (ESI):** *m/z* calcd for $C_{129}H_{200}N_8O_{32}Na$ $[M+Na]^+$ 2396.4161; Found, 2396.4179.

((2*R*,3*S*,4*S*,5*R*,6*R*)-6-(((2*R*,3*R*,4*R*,5*S*,6*R*)-5-(2-(2-(4-((10*R*)-10-(2-acetamido-3-(((2*R*,3*S*,4*S*,5*S*,6*S*)-3-acetamido-4,5-dihydroxy-6-(hydroxymethyl)tetrahydro-2*H*-pyran-2-yl)oxy)butanamido)-9,13-dioxo-19-(((2*R*,3*R*,4*R*,5*R*,6*S*)-3,4,5-trihydroxy-6-methyltetrahydro-2*H*-pyran-2-yl)oxy)-2,5,17-trioxa-8,14-diazanonadecyl)-1*H*-1,2,3-triazol-1-yl)ethoxy)ethoxy)-3,4-dihydroxy-6-(((3-nonyldodecanoyl)oxy)methyl)tetrahydro-2*H*-pyran-2-yl)oxy)-3,4,5-trihydroxytetrahydro-2*H*-pyran-2-yl)methyl 3-nonyldodecanoate (RVT)

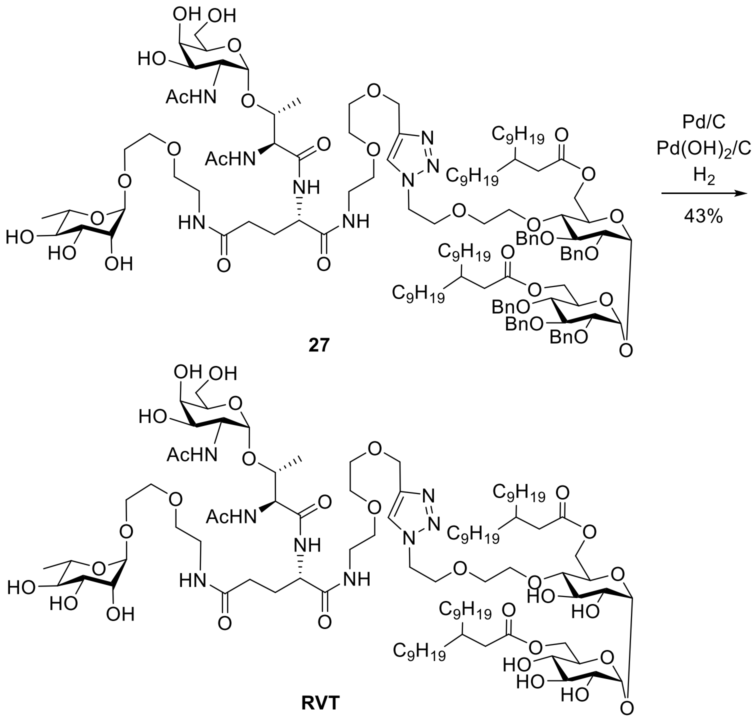

A 10 mL mixed solvent of DCM/MeOH/H_2O (4:2:1) was used to dissolve compound **27** (8.0 mg, 2.0 μmol). Pd/C (5.0 mg) and Pd(OH)$_2$/C (5.0 mg) were added. Hydrogen balloon was used for the hydrogenation, then the reaction was stirred for 24 hours. Diatomaceous earth was used to remove the insoluble and washed with 30 mL of DCM/MeOH/H_2O (5:5:1). The filtrate was distilled under reduced pressure to remove the solvent, the residue was purified by gel filtration chromatography on Sephadex LH-20 (MeOH) to afford compound **RVT** as a white solid (4.0 mg, 43% yield). **^{1}H NMR (400 MHz, CD_3OD)** δ 7.94 (s, 1H), 4.95 (d, J = 3.8 Hz, 2H), 4.62 (s, 3H), 4.57 (s, 3H), 4.52 – 4.43 (m, 5H), 4.28 – 4.05 (m, 11H), 3.96 – 3.75 (m, 11H), 3.74 – 3.41 (m, 25H), 3.40 – 3.24 (m, 11H), 3.10 (t, J = 9.5 Hz, 3H), 2.18 (d, J = 6.7 Hz, 4H), 2.00 (s, 3H),

1.96 (s, 1H), 1.93 (d, J = 4.5 Hz, 3H), 1.84 (d, J = 6.8 Hz, 1H), 1.75 (s, 2H), 1.42 – 0.97 (m, 70H), 0.80 (t, J = 6.8 Hz, 12H). **HRMS (ESI):** *m/z* calcd for $C_{94}H_{170}N_8O_{32}Na$ $[M+Na]^+$ 1946.1813; Found, 1946.1873.

2.4 Synthesis and Characterization of Three-Component Vaccine MVT

(2*R*,3*R*,4*S*,5*S*,6*S*)-2-(acetoxymethyl)-6-(((*S*)-5-(tert-butoxycarbonyl)-1-(9*H*-fluoren-9-yl)-3,8-dioxo-2,12-dioxa-4,9-diazatetradecan-14-yl)oxy)tetrahydro-2*H*-pyran-3,4,5-triyl triacetate (28)

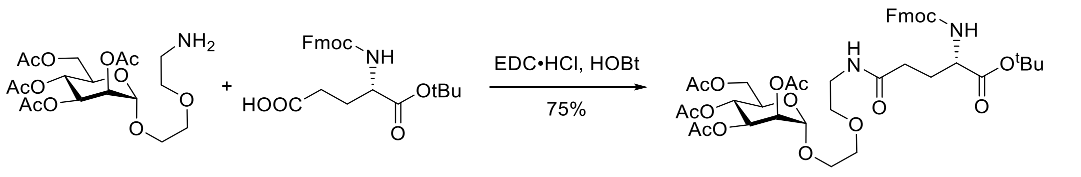

6 7 28

Compound **6** (5 g, 0.01149 mol), compound **7** (4.88 g, 0.01149 mol), EDC•HCl (10.9 g, 0.05745 mol), and HOBt (140 mg, 0.001149 mol) were dissolved in 50 mL DCM. The reaction mixture was stirred at room temperature for 3 hours and monitored by TLC staining with 25% sulfuric acid in ethanol (developing solvent: MeOH/DCM = 1:40). Complete conversion was confirmed by TLC analysis. The reaction was diluted with DCM (200 mL) and transferred to a separatory funnel. Washed sequentially with water (100 mL) and saturated NaCl solution (100 mL). The organic layer was collected, dried over anhydrous sodium sulfate, and filtered. Then the filtrate was distilled under reduced pressure to remove the organic solvent, yielding the crude product. Purification by column chromatography (DCM/MeOH, from 200:1 to 80:1) afforded compound **28** as a white viscous solid (7 g, 75% yield). **^{1}H NMR (400 MHz, $CDCl_3$)** δ 7.76 (dd, J = 7.4, 1.1 Hz, 2H), 7.63 – 7.59 (m, 2H), 7.41 – 7.38 (m, 2H), 7.33 – 7.29 (m, 2H), 6.43 (s, 1H), 5.85 (d, J = 8.1 Hz, 1H), 5.36 (dd, J = 10.0, 3.4 Hz, 1H), 5.31 – 5.27 (m, 1H), 5.25 (dd, J = 3.7, 2.0 Hz, 1H), 4.91 (d, J = 1.8 Hz, 1H), 4.44 – 4.39 (m, 1H), 4.37 – 4.32 (m, 1H), 4.28 – 4.20 (m, 4H), 4.13 (dd, J = 12.2, 2.5 Hz, 1H), 4.05 (ddd, J = 9.9, 5.3, 2.5 Hz, 1H), 3.80 – 3.75 (m, 1H), 3.69 – 3.65 (m, 1H), 3.63 – 3.58 (m, 2H), 3.55 – 3.51 (m, 2H), 3.45 (dt, J = 10.8, 3.8 Hz, 2H), 2.31 (q, J = 6.6, 6.1 Hz, 2H), 2.27 – 2.16 (m, 2H), 2.14 (s, 3H), 2.09 (s, 3H), 2.04 (s, 3H), 1.99 (s, 3H), 1.47 (s, 9H). **$^{13}C\{^1H\}$ NMR**

(101 MHz, $CDCl_3$) *δ* 172.28, 171.22, 170.71, 170.29, 170.12, 169.75, 156.29, 143.98, 143.75, 141.29, 141.27, 127.70, 127.07, 125.20, 125.14, 119.97, 97.52, 82.19, 77.42, 77.30, 77.10, 76.78, 70.06, 69.94, 69.74, 68.96, 68.44, 67.04, 66.94, 66.26, 62.55, 54.21, 47.18, 39.35, 32.36, 28.47, 27.99, 20.91, 20.75, 20.69. **HRMS (ESI):** *m/z* calcd for $C_{42}H_{54}N_2O_{16}Na$ $[M+Na]^+$ 865.3366; Found, 865.3397.

***N*²-(((9*H*-fluoren-9-yl)methoxy)carbonyl)-*N*⁵-(2-(2-(((2*R*,3*R*,4*R*,5*S*,6*S*)-3,4,5-triacetoxy-6-(acetoxymethyl)tetrahydro-2*H*-pyran-2-yl)oxy)ethoxy)ethyl)-D-glutamine (29)**

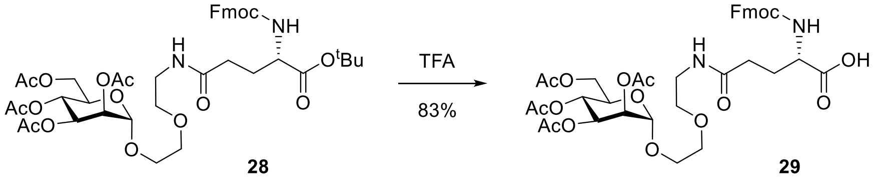

Compound **28** (2 g, 0.0023 mol) was dissolved in 20 mL DCM. 5 mL TFA was added dropwise over ice bath. The reaction was stirred for 10 hours and monitored by TLC staining with 25% sulfuric acid in ethanol (developing solvent: DCM/MeOH = 20:1). Complete conversion was confirmed by TLC analysis. Remove glacial acetic acid and DCM by vacuum distillation to give a pale-yellow crude product. Purification by column chromatography (DCM/MeOH, from 100:1 to 20:1) afforded a colorless, transparent, viscous liquid compound **29** (1.5 g, 83% yield). **^{1}H NMR (400 MHz, $CDCl_3$)** *δ* 7.75 (d, J = 7.5 Hz, 2H), 7.59 (t, J = 6.9 Hz, 2H), 7.40 – 7.37 (m, 2H), 7.31 (dd, J = 7.4, 1.2 Hz, 2H), 6.88 (s, 1H), 6.10 (d, J = 7.2 Hz, 1H), 5.34 (dd, J = 10.1, 3.3 Hz, 1H), 5.31 – 5.25 (m, 2H), 5.24 (dd, J = 3.4, 1.7 Hz, 1H), 4.91 (d, J = 1.7 Hz, 1H), 4.41 – 4.36 (m, 2H), 4.31 (d, J = 10.5 Hz, 1H), 4.21 (q, J = 7.1, 6.2 Hz, 2H), 4.13 (dd, J = 12.2, 2.6 Hz, 1H), 4.07 – 4.02 (m, 1H), 3.80 – 3.75 (m, 1H), 3.68 (dd, J = 6.6, 2.8 Hz, 1H), 3.63 – 3.57 (m, 2H), 3.53 (t, J = 4.1 Hz, 2H), 3.49 (d, J = 6.6 Hz, 1H), 3.43 (d, J = 5.0 Hz, 1H), 2.54 – 2.38 (m, 2H), 2.20 (dd, J = 8.5, 5.1 Hz, 1H), 2.14 (s, 3H), 2.12 (d, J = 6.7 Hz, 1H), 2.09 (s, 3H), 2.04 (s, 3H), 2.00 (s, 3H). **$^{13}C\{^1H\}$ NMR (101 MHz, $CDCl_3$)** *δ* 173.88, 173.45, 170.88, 170.53, 170.45, 169.82, 156.27, 143.91, 143.69, 141.28, 141.26, 127.73, 127.10, 125.20, 125.14, 119.98, 97.52, 77.38, 77.26, 77.06,

77.00, 76.74, 69.97, 69.74, 69.09, 68.46, 67.10, 67.04, 66.19, 62.59, 53.41, 47.11, 39.64, 32.29, 28.72, 20.93, 20.77, 20.73, 20.71. **HRMS (ESI):** *m/z* calcd for $C_{28}H_{26}N_2O_{16}Na$ $[M+Na]^+$ 809.2740 ; Found, 809.2754.

(2*R*,3*R*,4*R*,5*S*,6*S*)-2-(((*R*)-10-((((9*H*-fluoren-9-yl)methoxy)carbonyl)amino)-7,11-dioxo-3,15,18-trioxa-6,12-diazahenicos-20-yn-1-yl)oxy)-6-(acetoxymethyl)tetrahydro-2*H*-pyran-3,4,5-triyl triacetate (30)

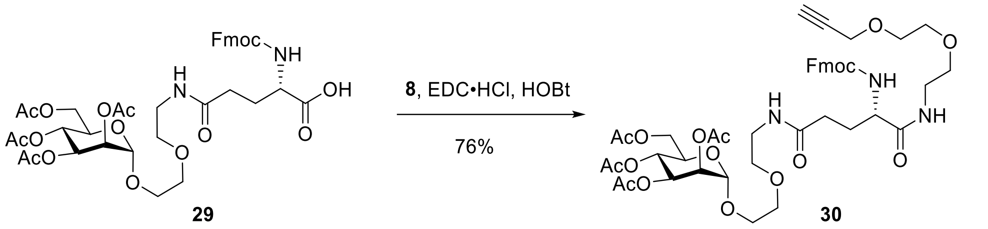

Compound **29** (1.5 g, 1.908 mmol), compound **8** (236 mg, 1.908 mmol), EDC•HCl (1.82 g, 9.54 mmol), and HOBt (122 mg, 0.1908 mol) were dissolved in DCM (50 mL). The reaction mixture was stirred at room temperature for 3 hours and monitored by TLC staining with 25% sulfuric acid in ethanol (developing solvent: MeOH/DCM = 1:40). Complete conversion was confirmed by TLC analysis. The reaction was diluted with DCM (200 mL) and transferred to a separatory funnel. Washed sequentially with water (100 mL) and saturated NaCl solution (100 mL). The organic layer was collected, dried over anhydrous sodium sulfate, and filtered. Then the filtrate was distilled under reduced pressure to remove the organic solvent, yielding the crude product. Purification by column chromatography (DCM/MeOH, from 200:1 to 80:1) gave compound **30** as a white solid (1.3 g, 76% yield). 7.75 – 7.73 (m, 2H), 7.59 (d, *J* = 7.5 Hz, 2H), 7.39 (dd, *J* = 7.6, 1.2 Hz, 2H), 7.30 (dd, *J* = 7.5, 1.2 Hz, 2H), 7.17 (s, 1H), 6.63 (s, 1H), 6.26 – 6.21 (m, 1H), 5.33 (dd, *J* = 9.9, 3.4 Hz, 1H), 5.27 (d, *J* = 9.8 Hz, 1H), 5.22 (dd, *J* = 3.4, 1.8 Hz, 1H), 4.89 (d, *J* = 1.8 Hz, 1H), 4.34 (d, *J* = 7.3 Hz, 2H), 4.23 (d, *J* = 6.9 Hz, 1H), 4.18 (d, *J* = 2.4 Hz, 1H), 4.15 (d, *J* = 2.4 Hz, 2H), 4.11 (d, *J* = 9.7 Hz, 1H), 4.04 – 4.00 (m, 1H), 3.79 – 3.75 (m, 1H), 3.63 (tdd, *J* = 5.7, 3.7, 1.9 Hz, 8H), 3.56 – 3.53 (m, 4H), 3.48 (d, *J* = 4.8 Hz, 2H), 3.44 (d, *J* = 4.8 Hz, 1H), 3.40 (t, *J* = 5.0 Hz, 1H), 2.45 (d, *J* = 2.4 Hz, 1H), 2.38 – 2.30 (m, 2H), 2.13 (s, 3H), 2.07 (s, 3H), 2.03 (s, 1H), 2.02 (s, 3H), 1.99 (d, *J* = 2.7 Hz, 1H), 1.98 (s, 3H). **$^{13}C\{^1H\}$ NMR (101 MHz, $CDCl_3$)** δ 172.92, 171.47, 170.73, 170.31, 170.18, 169.75, 156.31, 143.91, 143.81, 141.28, 141.26,

127.72, 127.09, 125.20, 125.18, 119.98, 97.48, 79.50, 77.37, 77.26, 77.06, 76.74, 74.87, 70.04, 69.92, 69.86, 69.70, 69.57, 69.00, 68.46, 67.02, 66.20, 62.54, 58.36, 54.43, 47.15, 39.40, 39.31, 32.40, 29.16, 20.92, 20.76, 20.72, 20.71. **HRMS (ESI):** *m/z* calcd for $C_{45}H_{58}N_3O_{17}$ $[M+H]^+$ 912.3762; Found, 912.3735.

(2*S*,3*S*,4*R*,5*R*,6*R*)-2-(acetoxymethyl)-6-(((*R*)-10-amino-7,11-dioxo-3,15,18-trioxa-6,12-diazahenicos-20-yn-1-yl)oxy)tetrahydro-2*H*-pyran-3,4,5-triyl triacetate (31)

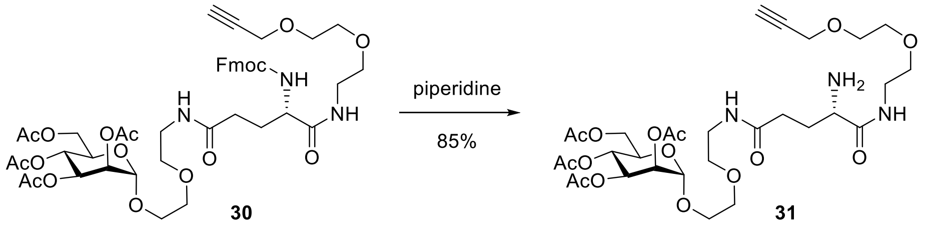

Compound **30** (400 mg, 0.4689 mmol) was dissolved in 10 mL DMF. Under an ice bath, 0.2 mL of piperidine was added dropwise. The reaction mixture was stirred for 10 hours and monitored by TLC staining with 25% sulfuric acid in ethanol (developing solvent: DCM/MeOH = 10:1). Complete conversion was confirmed by TLC analysis. DMF and piperidine were removed by vacuum distillation to give a pale-yellow crude product. Purification by column chromatography (DCM/MeOH, from 90:1 to 10:1) afforded compound **40** as a colorless, transparent, viscous liquid (250 mg, yield 85%). **^{1}H NMR (400 MHz, $CDCl_3$)** δ 7.62 (t, J = 5.8 Hz, 1H), 6.71 (t, J = 5.6 Hz, 1H), 5.35 (dd, J = 10.0, 3.3 Hz, 1H), 5.28 (t, J = 9.9 Hz, 1H), 5.24 (dd, J = 3.3, 1.8 Hz, 1H), 4.92 (d, J = 1.7 Hz, 1H), 4.27 (dd, J = 12.2, 5.3 Hz, 1H), 4.20 (d, J = 2.4 Hz, 2H), 4.13 (dd, J = 12.2, 2.5 Hz, 1H), 4.05 (ddd, J = 9.7, 5.2, 2.5 Hz, 1H), 3.84 – 3.75 (m, 1H), 3.73 – 3.54 (m, 11H), 3.53 – 3.36 (m, 5H), 2.48 (t, J = 2.4 Hz, 1H), 2.34 (td, J = 7.0, 3.6 Hz, 2H), 2.17 (s, 3H), 2.11 (s, 3H), 2.08 (d, J = 6.1 Hz, 1H), 2.06 (s, 3H), 2.01 (s, 3H), 1.93 (dt, J = 14.1, 7.1 Hz, 1H). **^{13}C{^{1}H} NMR (101 MHz, $CDCl_3$)** δ 174.84, 173.01, 170.70, 170.25, 170.04, 169.74, 97.55, 79.51, 77.40, 77.28, 77.08, 76.76, 74.80, 70.01, 69.99, 69.86, 69.74, 69.66, 68.99, 68.98, 68.45, 67.11, 66.19, 62.52, 58.38, 54.52, 39.25, 38.89, 32.71, 31.07, 20.92, 20.77, 20.71. **HRMS (ESI):** *m/z* calcd for $C_{30}H_{48}N_3O_{15}$ $[M+H]^+$ 690.3080; Found, 690.3081.

(2*R*,3*R*,4*R*,5*S*,6*S*)-2-(((10*R*)-10-(2-acetamido-3-(((2*R*,3*S*,4*S*,5*S*,6*S*)-3-acetamido-4,5-diacetoxy-6-(acetoxymethyl)tetrahydro-2*H*-pyran-2-yl)oxy)butanamido)-7,11-dioxo-3,15,18-trioxa-6,12-diazahenicos-20-yn-1-yl)oxy)-6-(acetoxymethyl)tetrahydro-2*H*-pyran-3,4,5-triyl triacetate (32)

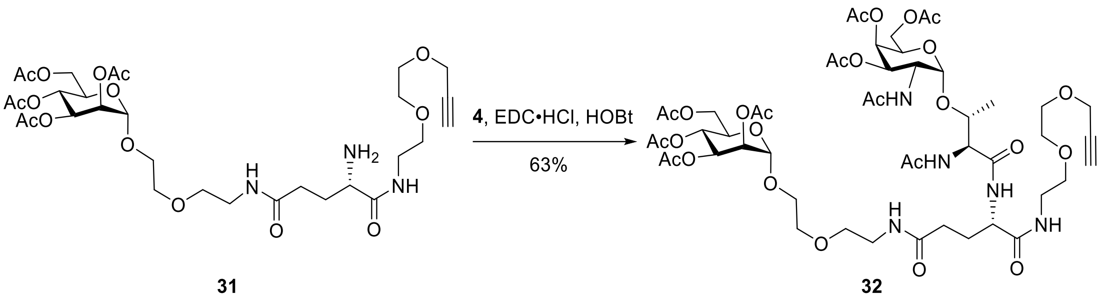

Compound **31** (100.0 mg, 0.1580 mmol), compound **4** (77.6 mg, 0.1580 mmol), EDC•HCl (120.0 mg, 0.6320 mmol), and HOBt (22.4 mg, 0.01580 mmol) were dissolved in DCM (10 mL). The mixture was stirred at room temperature for 3 hours and monitored by TLC staining with 25% sulfuric acid in ethanol (developing solvent: MeOH/DCM = 1:40). Complete conversion was confirmed by TLC analysis. The reaction was diluted with DCM (100 mL) and transferred to a separatory funnel. Washed sequentially with water (50 mL) and saturated NaCl solution (50 mL). The organic layer was collected, dried over anhydrous sodium sulfate, and filtered. Then the filtrate was distilled under reduced pressure to remove the organic solvent, yielding the crude product. Purification by column chromatography (DCM/MeOH, from 200:1 to 80:1) afforded compound **32** as a white viscous solid (115 mg, 63% yield). **^{1}H NMR (400 MHz, $CDCl_3$)** δ 8.05 (d, J = 6.6 Hz, 1H), 7.42 (s, 1H), 7.02 (d, J = 9.1 Hz, 1H), 6.89 – 6.69 (m, 2H), 5.39 (dd, J = 3.2, 1.3 Hz, 1H), 5.34 (dd, J = 10.0, 3.2 Hz, 1H), 5.32 – 5.26 (m, 1H), 5.24 (dd, J = 3.2, 1.7 Hz, 1H), 5.17 (dd, J = 11.6, 3.2 Hz, 1H), 5.06 (d, J = 3.6 Hz, 1H), 4.92 (d, J = 1.8 Hz, 1H), 4.56 (ddd, J = 11.5, 8.7, 3.1 Hz, 2H), 4.38 (d, J = 6.3 Hz, 1H), 4.33 – 4.22 (m, 3H), 4.20 (d, J = 2.3 Hz, 2H), 4.17 – 4.11 (m, 1H), 4.10 – 4.01 (m, 3H), 3.85 – 3.78 (m, 1H), 3.77 – 3.31 (m, 16H), 2.51 (t, J = 2.4 Hz, 1H), 2.48 – 2.36 (m, 2H), 2.17 (s, 3H), 2.15 (s, 3H), 2.12 (s, 3H), 2.11 (s, 3H), 2.10 – 2.07 (m, 1H), 2.06 (s, 3H), 2.04 (s, 3H), 2.01 (s, 3H), 2.00 (d, J = 7.1 Hz, 1H), 1.98 (s, 3H),

1.96 (s, 3H), 1.24 (d, *J* = 6.2 Hz, 3H). **$^{13}C\{^1H\}$ NMR (101 MHz, $CDCl_3$)** δ 173.31, 171.23, 171.07, 170.80, 170.77, 170.54, 170.48, 170.36, 170.22, 169.75, 169.56, 99.33, 97.54, 79.45, 77.36, 77.25, 77.04, 76.73, 76.19, 75.00, 70.01, 69.94, 69.82, 69.68, 69.41, 69.09, 69.03, 68.47, 68.35, 67.40, 67.14, 67.10, 66.19, 62.55, 62.29, 58.39, 56.68, 53.14, 47.68, 39.53, 39.38, 31.95, 28.29, 23.20, 22.91, 20.95, 20.79, 20.75, 20.73, 20.70, 17.08. **HRMS (ESI):** *m/z* calcd for $C_{50}H_{76}N_5O_{26}$ $[M+H]^+$ 1162.4773; found, 1162.4793.

(2*R*)-2-(2-acetamido-3-(((2*R*,3*S*,4*S*,5*S*,6*S*)-3-acetamido-4,5-dihydroxy-6-(hydroxymethyl)tetrahydro-2*H*-pyran-2-yl)oxy)butanamido)-*N*^{1}-(2-(2-(prop-2-yn-1-yloxy)ethoxy)ethyl)-*N*^{5}-(2-(2-(((2*R*,3*R*,4*R*,5*R*,6*S*)-3,4,5-trihydroxy-6-(hydroxymethyl)tetrahydro-2*H*-pyran-2-yl)oxy)ethoxy)ethyl)pentanediamide (33)

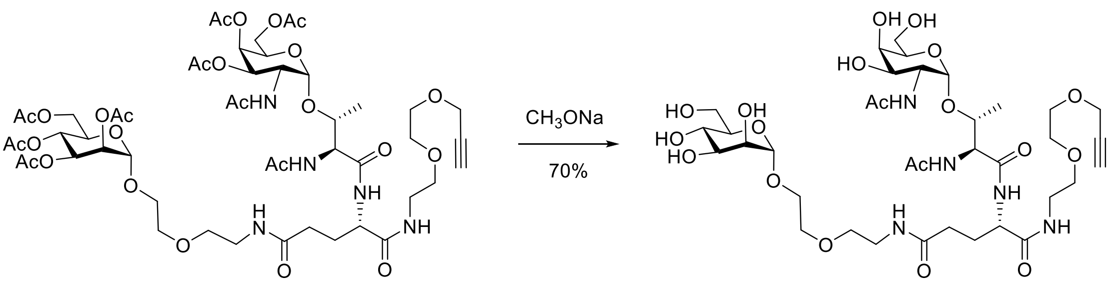

32 **33**

Compound **32** (100 mg, 0.0906 mmol) and CH_3ONa (48.9 mg, 0.09060 mol) were dissolved in methanol (10 mL). The reaction was stirred at room temperature for 24 hours and monitored by TLC staining with 25% sulfuric acid in ethanol (developing solvent: DCM/MeOH/H_2O = 2:1:0.1). Complete conversion was confirmed by TLC analysis. Acidic ion exchange resin was added and stirred until the reaction mixture becomes clear, pH = 7. Then the reaction was filtered, and the filtrate was distilled under reduced pressure to remove the organic solvent, yielding a pale-yellow crude product. The crude product was washed with DCM, then was dissolved in 200 μL MeOH. 8 mL DCM was added dropwise to precipitate crystals, yielding compound **33** as a white solid (53.0 mg, 70% yield). **^1H NMR (400 MHz, CD_3OD)** δ 4.81 (d, *J* = 1.7 Hz, 1H), 4.53 (d, *J* = 2.4 Hz, 1H), 4.32 – 4.17 (m, 5H), 3.91 (d, *J* = 4.2 Hz, 2H), 3.85 – 3.80 (m, 3H), 3.79 – 3.70 (m, 4H), 3.70 – 3.50 (m, 13H), 3.44 (dt, *J* = 13.9, 5.3 Hz, 1H), 3.36 (dt, *J* = 7.7, 4.7 Hz, 3H), 2.87 (t, *J* = 2.4 Hz, 1H), 2.30 (t, *J* = 7.5 Hz, 2H), 2.11 (s,

3H), 2.04 (s, 3H), 2.03 – 1.85 (m, 2H), 1.28 (d, J = 6.3 Hz, 3H). **$^{13}C\{^{1}H\}$ NMR (101 MHz, CD_3OD)** δ 173.50, 172.97, 172.41, 172.33, 170.78, 100.25, 99.17, 79.18, 75.86, 74.73, 73.25, 71.66, 71.17, 70.72, 69.79, 69.66, 69.16, 69.01, 68.93, 68.76, 68.63, 67.30, 66.33, 61.62, 61.37, 57.69, 57.15, 53.25, 50.15, 48.25, 48.10, 48.03, 47.89, 47.82, 47.68, 47.61, 47.47, 47.39, 47.18, 46.97, 39.12, 39.06, 31.71, 27.72, 21.83, 21.23, 17.56. **HRMS (ESI):** *m/z* calcd for $C_{36}H_{62}N_5O_{19}$ $[M+H]^+$ 868.4034; Found, 868.4038.

((2*R*,3*R*,4*S*,5*R*,6*R*)-6-(((2*R*,3*R*,4*S*,5*R*,6*R*)-5-(2-(2-(4-((10*R*)-10-(2-acetamido-3-(((2*R*,3*S*,4*S*,5*S*,6*S*)-3-acetamido-4,5-dihydroxy-6-(hydroxymethyl)tetrahydro-2*H*-pyran-2-yl)oxy)butanamido)-9,13-dioxo-19-(((2*R*,3*R*,4*R*,5*R*,6*S*)-3,4,5-trihydroxy-6-(hydroxymethyl)tetrahydro-2*H*-pyran-2-yl)oxy)-2,5,17-trioxa-8,14-diazanonadecyl)-1H-1,2,3-triazol-1-yl)ethoxy)ethoxy)-3,4-bis(benzyloxy)-6-(((3-nonyldodecanoyl)oxy)methyl)tetrahydro-2H-pyran-2-yl)oxy)-3,4,5-tris(benzyloxy)tetrahydro-2H-pyran-2-yl)methyl 3-nonyldodecanoate (34)

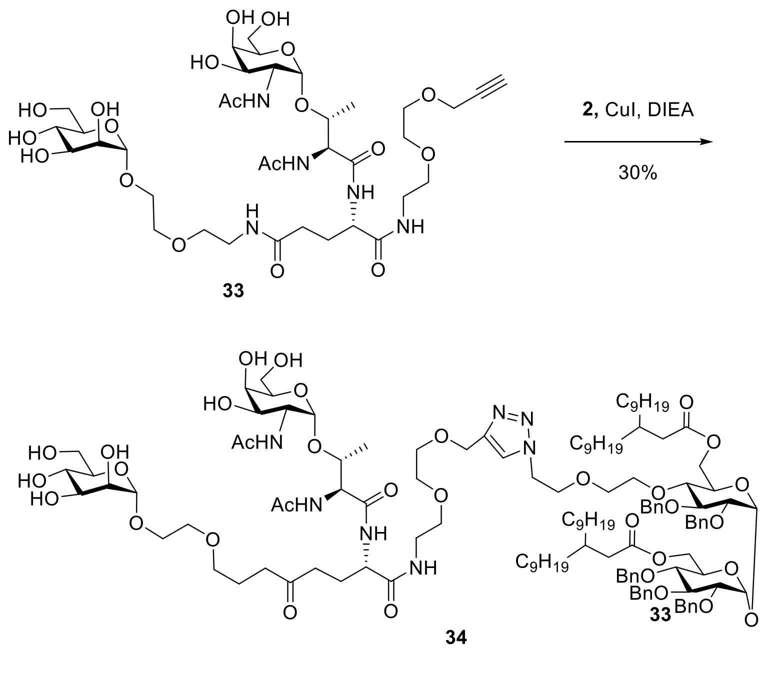

A 4 mL mixture of THF/MeOH (1:1) was used to dissolve compound **33** (30.0 mg, 0.03525 mmol), compound **2** (53.6 mg, 0.03525 mmol), copper(I) iodide (133 mg, 0.7050 mmol), followed by addition of *N*,*N*-diisopropylethylamine (118 μL, 0.7050 mmol). The mixture was stirred at room temperature for 12 hours and monitored by

TLC staining with 25% sulfuric acid in ethanol (developing solvent: MeOH/DCM/H_2O = 1:4:0.1). Complete conversion was confirmed by TLC analysis. Diatomaceous earth was used to remove the insoluble, and then the solvent was removed from the filtrate by vacuum distillation to afford the crude product. Purification by column chromatography (DCM/MeOH, from 10:1 to 4:1) afforded compound **34** as a white solid (24 mg, 30% yield). **^{1}H NMR (400 MHz, $CD_3OD/CDCl_3$)** δ 7.82 – 7.77 (m, 1H), 7.20 (tdt, *J* = 14.1, 8.9, 1.6 Hz, 20H), 5.15 (dd, *J* = 6.7, 3.5 Hz, 2H), 4.87 (d, *J* = 11.1 Hz, 1H), 4.70 – 4.67 (m, 2H), 4.65 – 4.59 (m, 3H), 4.53 – 4.36 (m, 7H), 4.22 – 3.95 (m, 10H), 3.94 – 3.77 (m, 6H), 3.77 – 3.65 (m, 6H), 3.65 – 3.56 (m, 5H), 3.55 – 3.41 (m, 16H), 3.39 – 3.34 (m, 2H), 3.25 (q, *J* = 2.5, 1.4 Hz, 4H), 2.17 (t, *J* = 7.5 Hz, 2H), 2.12 (d, *J* = 6.7 Hz, 2H), 2.08 (d, *J* = 6.7 Hz, 2H), 1.98 (d, *J* = 2.4 Hz, 3H), 1.94 (dd, *J* = 6.0, 2.6 Hz, 1H), 1.92 (d, *J* = 2.3 Hz, 3H), 1.82 (dd, *J* = 14.4, 7.5 Hz, 1H), 1.69 (s, 2H), 1.32 – 1.04 (m, 67H), 0.79 (td, *J* = 5.3, 2.8 Hz, 12H). **^{13}C{^{1}H} NMR (101 MHz, $CD_3OD/CDCl_3$)** δ 173.43, 173.34, 172.95, 172.35, 172.29, 170.79, 144.23, 138.69, 138.16, 137.98, 128.19, 128.16, 128.04, 128.02, 127.71, 127.67, 127.58, 127.49, 127.28, 124.50, 100.23, 99.18, 92.68, 80.94, 78.19, 77.87, 77.54, 75.93, 74.96, 74.55, 73.21, 73.07, 72.87, 71.88, 71.63, 71.18, 70.71, 70.38, 69.79, 69.38, 69.18, 69.02, 68.94, 68.69, 67.30, 66.31, 63.57, 61.63, 61.39, 57.15, 50.18, 50.00, 48.30, 48.16, 48.09, 47.94, 47.87, 47.73, 47.66, 47.52, 47.45, 47.23, 47.02, 39.12, 38.80, 38.70, 35.01, 33.53, 31.73, 29.64, 29.60, 29.39, 29.16, 27.70, 26.30, 26.24, 22.40, 21.88, 21.31, 17.58, 13.19. **HRMS (ESI):** *m/z* calcd for $C_{129}H_{200}N_8O_{33}Na$ $[M+Na]^+$ 2412.4110; Found, 2412.4113.

((2*R*,3*S*,4*S*,5*R*,6*R*)-6-(((2*R*,3*R*,4*R*,5*S*,6*R*)-5-(2-(2-(4-((10*R*)-10-(2-acetamido-3-(((2*R*,3*S*,4*S*,5*S*,6*S*)-3-acetamido-4,5-dihydroxy-6-(hydroxymethyl)tetrahydro-2*H*-pyran-2-yl)oxy)butanamido)-9,13-dioxo-19-(((2*R*,3*R*,4*R*,5*R*,6*S*)-3,4,5-trihydroxy-6-(hydroxymethyl)tetrahydro-2*H*-pyran-2-yl)oxy)-2,5,17-trioxa-8,14-diazanonadecyl)-1*H*-1,2,3-triazol-1-yl)ethoxy)ethoxy)-3,4-dihydroxy-6-(((3-nonyldodecanoyl)oxy)methyl)tetrahydro-2*H*-pyran-2-yl)oxy)-3,4,5-trihydroxytetrahydro-2*H*-pyran-2-yl)methyl 3-nonyldodecanoate (MVT)

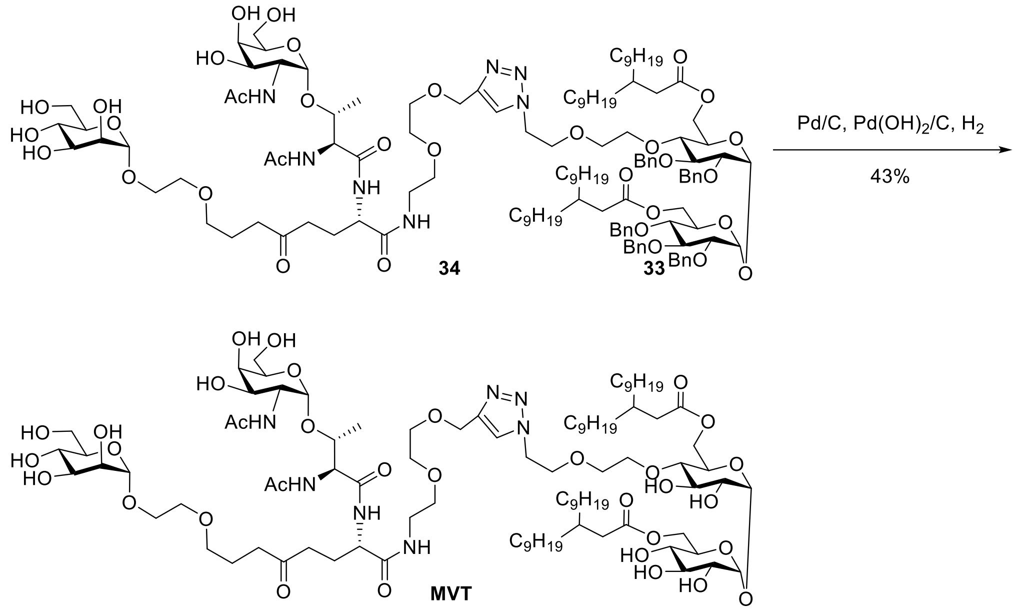

Compound **34** (8.0 mg, 2.0 μmol) was dissolved in 10 mL of a mixed solvent of DCM/MeOH/H_2O (4:2:1). Pd/C (5.0 mg) and $Pd(OH)_2/C$ (5.0 mg) were added. Hydrogen balloon was used for hydrogenation, and the reaction mixture was stirred for 24 hours. Diatomaceous earth was used to filter out insoluble and washed with 30 mL of DCM/MeOH/H_2O (5:5:1). The filtrate was distilled under reduced pressure to remove the solvent, the residue was purified by gel filtration chromatography on Sephadex LH-20 (MeOH) to afford compound **MVT** as a white solid (4.0 mg, 43% yield). **^{1}H NMR (400 MHz, CD_3OD)** δ 4.95 (d, J = 3.7 Hz, 2H), 4.57 (s, 3H), 4.53 – 4.44 (m, 6H), 4.28 – 4.05 (m, 14H), 3.92 (s, 7H), 3.84 – 3.42 (m, 39H), 3.40 – 3.24 (m, 12H), 2.18 (t, J = 5.8 Hz, 6H), 2.00 (s, 3H), 1.96 (s, 1H), 1.93 (s, 3H), 1.75 (s, 2H), 1.20 (s, 67H), 0.80 (t, J = 6.7 Hz, 12H). **HRMS (ESI):** *m/z* calcd for $C_{94}H_{170}N_8O_{33}Na$ $[M+Na]^+$ 1962.1763; Found, 1962.1711.

2.5 Synthesis and Characterization of Two-Component Vaccine VT

(2*R*,3*R*,4*R*,5*R*,6*S*)-5-acetamido-6-((3-acetamido-4-oxo-4-((2-(2-(prop-2-yn-1-yloxy)ethoxy)ethyl)amino)butan-2-yl)oxy)-2-(acetoxymethyl)tetrahydro-2*H*-pyran-3,4-diyl diacetate (35)

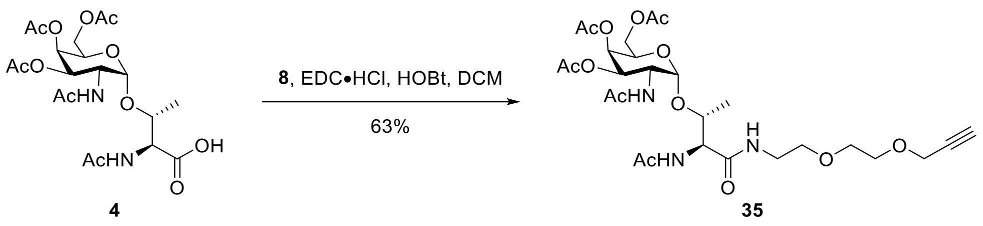

Compound **4** (100.0 mg, 0.2040 mmol), compound **8** (28.9 mg, 0.2040 mmol), EDC•HCl (120.0 mg, 0.8160 mmol), and HOBt (24.4 mg, 0.02040 mmol) were dissolved in DCM (10 mL). The mixture was stirred at room temperature for 3 hours and monitored by TLC staining with 25% sulfuric acid in ethanol (developing solvent: MeOH/DCM = 1:40). Complete conversion was confirmed by TLC analysis. The reaction was diluted with dichloromethane (100 mL) and transferred to a separatory funnel. Washed sequentially with water (50 mL) and saturated NaCl solution (50 mL). The organic layer was collected, dried over anhydrous sodium sulfate, and filtered. Then the filtrate distilled under reduced pressure to remove the organic solvent, yielding the crude product. Purification by column chromatography (DCM/MeOH, from 200:1 to 80:1) afforded compound **35** as a white viscous solid (115 mg, 63% yield).

2-acetamido-3-(((2*S*,3*R*,4*R*,5*R*,6*R*)-3-acetamido-4,5-dihydroxy-6-(hydroxymethyl)tetrahydro-2*H*-pyran-2-yl)oxy)-*N*-(2-(2-(prop-2-yn-1-yloxy)ethoxy)ethyl)butanamide (36)

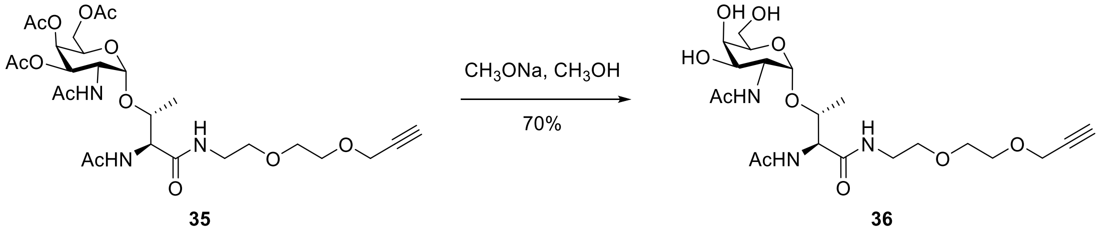

Dissolve compound **35** (100 mg, 0.1626 mmol) and CH_3ONa (35.1 mg, 0.6504 mmol) in methanol solution (10 mL). The reaction was stirred at room temperature for 24 hours and monitored by TLC staining with 25% sulfuric acid in ethanol (developing solvent: DCM/MeOH/H_2O = 4:1:0.1). Complete conversion was confirmed by TLC analysis. Acidic ion exchange resin was added into the reaction mixture and was stir until it becomes clear, pH = 7. The reaction was filtered, and the filtrate was distilled under reduced pressure to remove the organic solvent, yielding a pale-yellow crude product. The crude product was washed with DCM, and dissolved in 200 μL methanol. 8 mL dichloromethane was added to precipitate crystals, yielding compound **36** a white solid (56.0 mg, 70% yield).

((2*R*,3*R*,4*S*,5*R*,6*R*)-6-(((2*R*,3*R*,4*S*,5*R*,6*R*)-5-(2-(2-(4-(10-(1-(((2*S*,3*R*,4*R*,5*R*,6*R*)-3-acetamido-4,5-dihydroxy-6-(hydroxymethyl)tetrahydro-2*H*-pyran-2-yl)oxy)ethyl)-9,12-dioxo-2,5-dioxa-8,11-diazatridecyl)-1H-1,2,3-triazol-1-yl)ethoxy)ethoxy)-3,4-bis(benzyloxy)-6-(((3-nonyldodecanoyl)oxy)methyl)tetrahydro-2*H*-pyran-2-yl)oxy)-3,4,5-tris(benzyloxy)tetrahydro-2*H*-pyran-2-yl)methyl 3-nonyldodecanoate (37)

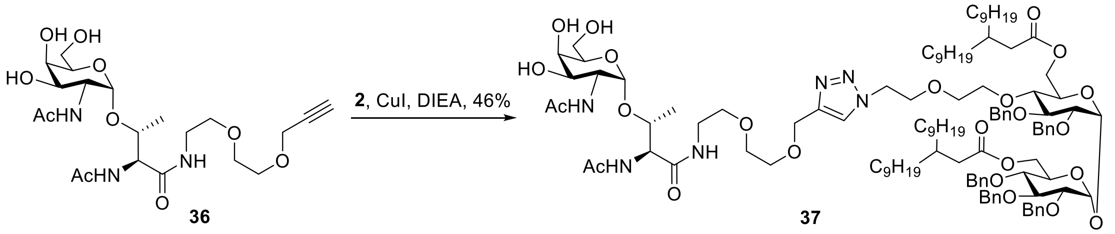

A 4 mL mixture of THF/MeOH (1:1) was used to dissolve compound **36** (30.0 mg, 0.03525 mmol), compound **2** (53.6 mg, 0.03525 mmol), copper(I) iodide (133 mg, 0.7050 mmol), followed by addition of *N*,*N*-diisopropylethylamine (118 μL, 0.7050 mmol). The mixture was stirred at room temperature for 12 hours and monitored by TLC staining with 25% sulfuric acid in ethanol (developing solvent: MeOH/DCM/H_2O = 1:4:0.1). Complete conversion was confirmed by TLC analysis. Diatomaceous earth was used to remove the insoluble, and then the solvent was removed from the filtrate by vacuum distillation to afford the crude product. Gel Purification by column chromatography (DCM/MeOH, from 10:1 to 4:1) afforded white solid compound **37**

(30 mg, 46% yield).[1]**H NMR (400 MHz, CD_3OD)** δ 7.95 (s, 1H), 7.45 – 7.18 (m, 25H), 5.27 (dd, J = 6.7, 3.5 Hz, 2H), 4.99 (d, J = 11.1 Hz, 1H), 4.83 – 4.79 (m, 2H), 4.76 – 4.71 (m, 3H), 4.63 – 4.48 (m, 7H), 4.32 – 4.06 (m, 9H), 4.05 – 3.82 (m, 8H), 3.78 – 3.39 (m, 19H), 2.24 (d, J = 6.7 Hz, 2H), 2.19 (d, J = 6.7 Hz, 2H), 2.08 (d, J = 2.6 Hz, 3H), 2.06 (d, J = 3.6 Hz, 3H), 1.81 (s, 2H), 1.28 (t, J = 4.2 Hz, 67H), 0.91 (dd, J = 7.2, 4.2 Hz, 12H). **HRMS (ESI):** *m/z* calcd for $C_{114}H_{174}N_6O_{24}Na$ $[M+Na]^+$ 2034.2472; Found, 2034.2496.

((2*R*,3*S*,4*S*,5*R*,6*R*)-6-(((2*R*,3*R*,4*R*,5*S*,6*R*)-5-(2-(2-(4-(10-(1-(((2*S*,3*R*,4*R*,5*R*,6*R*)-3-acetamido-4,5-dihydroxy-6-(hydroxymethyl)tetrahydro-2*H*-pyran-2-yl)oxy)ethyl)-9,12-dioxo-2,5-dioxa-8,11-diazatridecyl)-1H-1,2,3-triazol-1-yl)ethoxy)ethoxy)-3,4-dihydroxy-6-(((3-nonyldodecanoyl)oxy)methyl)tetrahydro-2*H*-pyran-2-yl)oxy)-3,4,5-trihydroxytetrahydro-2*H*-pyran-2-yl)methyl 3-nonyldodecanoate (VT)

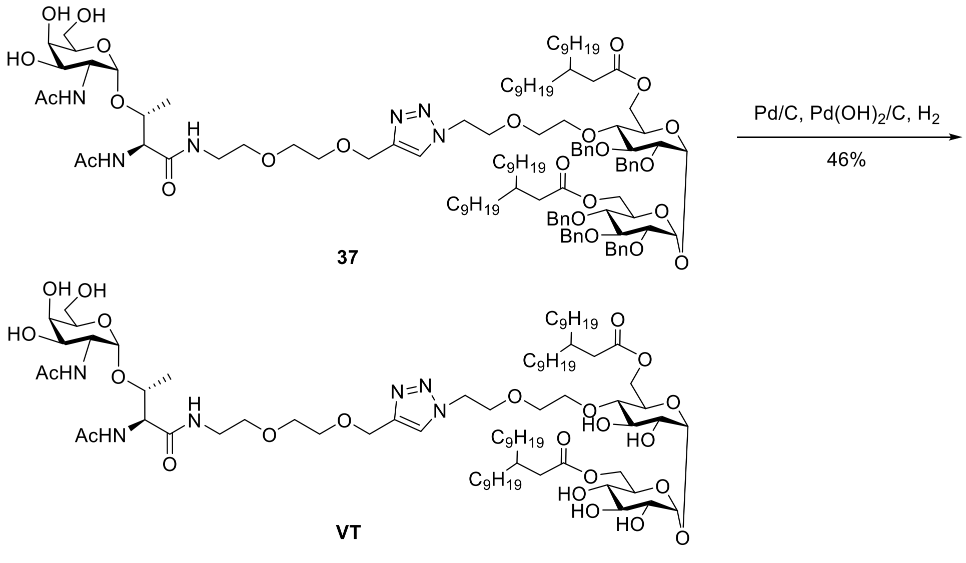

Compound **37** (8.0 mg, 2.0 μmol) was dissolved in 10 mL of a mixed solvent of DCM/MeOH/H_2O (4:2:1). Pd/C (5.0 mg) and $Pd(OH)_2/C$ (5.0 mg) were added. Hydrogen gas was passed through the mixture, which was then sealed and stirred for 24 hours. Diatomaceous earth was used to remove the insoluble and washed with 30 mL of DCM/MeOH/H_2O (5:5:1). The filtrate was distilled under reduced pressure to remove the solvent, the residue was purified by gel filtration chromatography on Sephadex LH-20 (MeOH) to afford compound **VT** as a white solid (4.0 mg, 46% yield).[1]**H NMR (400 MHz, CD_3OD)** δ 7.96 (s, 1H), 4.95 (d, J = 3.8 Hz, 2H), 4.57 (s,

2H), 4.50 (t, J = 5.6 Hz, 3H), 4.43 (d, J = 2.6 Hz, 1H), 4.24 (d, J = 11.8 Hz, 3H), 4.19 – 4.04 (m, 6H), 3.91 (d, J = 11.2 Hz, 5H), 3.79 (t, J = 5.3 Hz, 5H), 3.73 – 3.35 (m, 23H), 2.18 (dd, J = 6.8, 4.8 Hz, 4H), 1.98 (s, 3H), 1.96 (s, 3H), 1.75 (s, 3H), 1.20 (s, 67H), 0.80 (t, J = 6.6 Hz, 12H). **HRMS (ESI):** *m/z* calcd for $C_{79}H_{144}N_6O_{24}$ $[M]^+$ 1561.0227; Found, 1561.0271.

2.6 Preparation of Tn-CRM197

The preparation of Tn-CRM197 was based on our previous research,[1] and MALDI-TOF MS analysis showed that the carbohydrate loading of Tn-CRM197 was 4.8%.

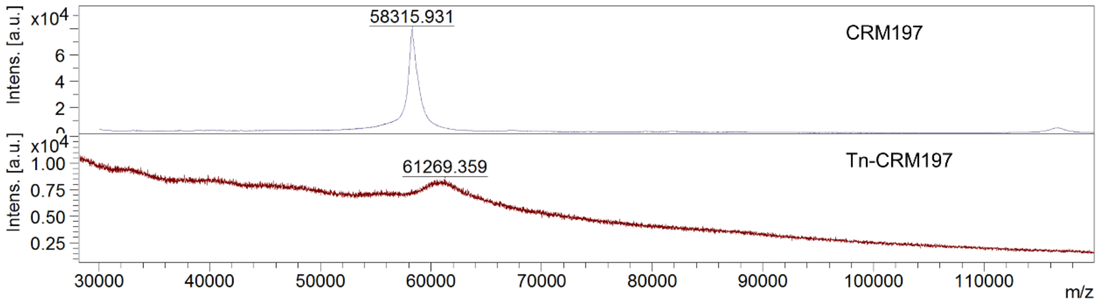

Figure S1. Characterization of glycoprotein Tn-CRM197.

2.7 Cytokine of Murine Bone-Marrow-Derived Macrophage Analysis

Bone marrow-derived macrophages (BMDMs) were prepared from Balb/c mice as previously described. The harvested BMDMs were seeded in 96-well plates at a density of 1 × 10^4 cells per well and incubated overnight at 37°C. Subsequently, the cells were treated with LPS (100 ng per well), vizantin, or its conjugates (1 nmol per well), in the presence or absence of Rhamnose sera (diluted 1:100). After 48 h of incubation, the levels of IL-6 and TNF-α in the culture supernatants were determined using ELISA kits, following the manufacturers' instructions.

Table S1. The capabilities of conjugate vaccines to induce BMDMs to produce inflammatory cytokines IL-6 and TNF-α.

	IL-6	TNF-α
Untreated	10.72 ± 1.49	18.51 ± 1.06
Vizantin	174.13 ± 3.81	180.64 ± 19.09
**VT**	143.47 ± 14.76	131.88 ± 3.73
**VT**/Rha sera	152.92 ± 3.24	159.14 ± 7.07
**MVT**	234.22 ± 8.72	333.17 ± 13.33
**MVT**/Rha sera	225.46 ± 9.95	312.96 ± 19.8
**RVT**	120.61 ± 13.54	211.45 ± 2.92
**RVT**/Rha sera	320.99 ± 28.34	384.57 ± 19.25
**RMVT**	351.88 ± 30.76	302.25 ± 13.39
**RMVT**/Rha sera	514.17 ± 21.99	589.88 ± 14.11
LPS	776.58 ± 8.46	1073.26 ± 30.95

2.8 Human Mincle Binding Assay

DSPC, cholesterol, and conjugates **VT**, **MVT**, **RVT**, **RMVT** were dissolved in the MeOH (50 μg/mL) and added to 96-well plates (100 μL per well). The solvents were evaporated at room temperature. Then, the coated plates were incubated with 100 μL hMincle-Fc (1 μg/mL in binding buffer) for 1.5 h at 37°C. After washing with PBST, HRP-rabbit anti-human IgG Fc was added, and the plates were incubated for another 1 h. TMB solution was added to the plates and incubated in the darkness for 20 min. Finally, 100 μL 0.5 M H_2SO_4 solution was added and the OD value at 450 nm was measured.

Table S2. The binding affinity of DSPC, Chol, and conjugates **VT**, **MVT**, **RVT**, **RMVT** to human Mincle-Fc protein ($\bar{x} \pm s$, n = 3)

	Human Mincle-Fc
Control	0.75 ± 0.07
DSPC/Chol	0.87 ± 0.04
Vizantin	3.44 ± 0.10
**VT**	3.10 ± 0.12
**MVT**	2.82 ± 0.09
**RVT**	2.82 ± 0.19
**RMVT**	2.92 ± 0.13

2.9 Liposome Size Analysis of the Conjugates VT, MVT, RVT, and RMVT

The preparation of the vaccine liposome used the previously reported protocol.[1-2] Briefly, a mixture of DSPC, cholesterol, and the conjugate in a molar ratio of 65:50:10 was dissolved in the mixture of DCM and MeOH (1:2, v/v). The solvents were removed under reduced pressure by rotary evaporation, and a thin lipid film formed on the vial wall. The film was hydrated with HEPES buffer (pH = 7.5) and shaken on a vortex mixer. Finally, the mixture was sonicated at rt for 15 min to yield the liposomal formulations of the conjugates **VT**, **MVT**, **RVT**, and **RMVT**. The average liposome diameter of **VT**, **MVT**, **RVT**, and **RMVT** is 1070.0 ± 62.63, 982.2 ± 62.34, 933.5 ± 35.54 nm, and 950.1 ± 18.75 nm. The polydispersity index (PDI) of **VT**, **MVT**, **RVT**, and **RMVT** is around 0.281, 0.222, 0.251, and 0.215. The emulsion of Tn-CRM197 with an alum adjuvant was prepared according to the protocol reported in the literature. Generally, Tn-CRM197 was dissolved in PBS buffer and thoroughly mixed with an alum adjuvant in accordance with the manufacturer's instructions.

Table S3. DLS analysis results for the liposomal formulation of **VT**.

Test	Size (d.nm)	PDI
1	1015	0.347
2	1056	0.208
3	1138	0.287
**Ave**	**1070**	**0.281**
**SD**	**62.63**	**0.07**

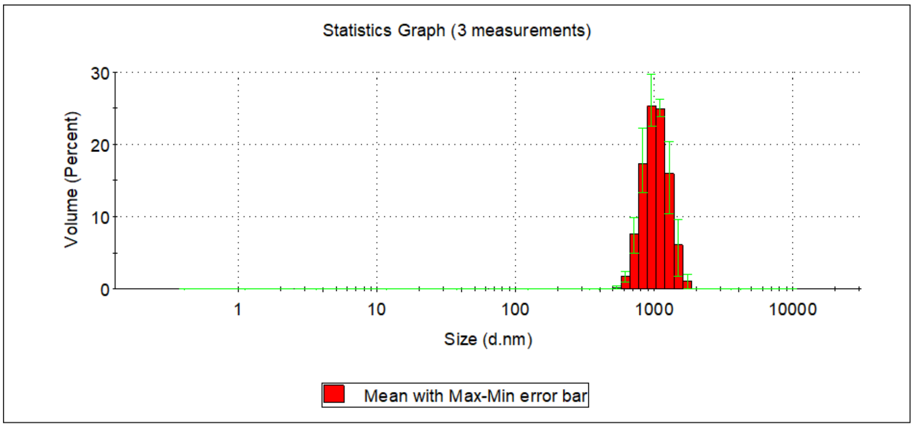

Figure S2. Size distribution of the liposomes of **VT** based on DLS volume analysis.

Table S4. DLS analysis results for the liposomal formulation of **MVT**.

Test	Size (d.nm)	PDI
1	910.7	0.235
2	1011	0.210
3	1025	0.222
**Ave**	**982.2**	**0.222**
**SD**	**62.34**	**0.013**

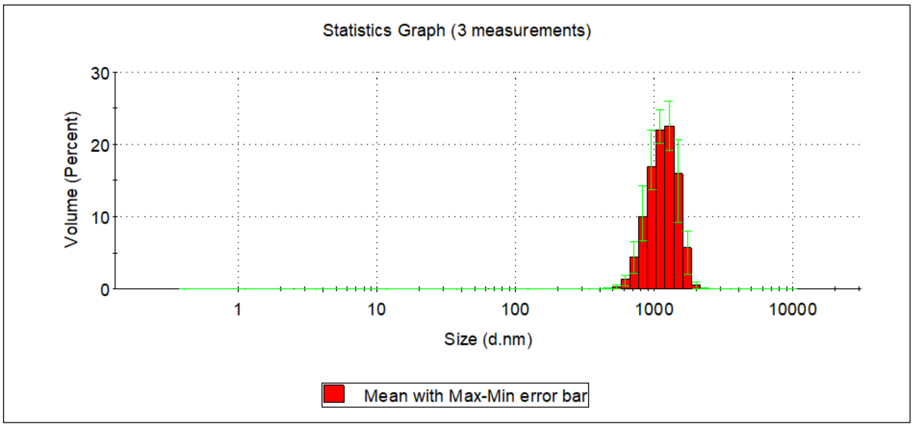

Figure S3. Size distribution of the liposomes of **MVT** based on DLS volume analysis.

Table S5. DLS analysis results for the liposomal formulation of **RVT**.

Test	Size (d.nm)	PDI
1	915.1	0.266
2	974.5	0.155
3	911	0.331
**Ave**	**933.5**	**0.251**
**SD**	**35.54**	**0.089**

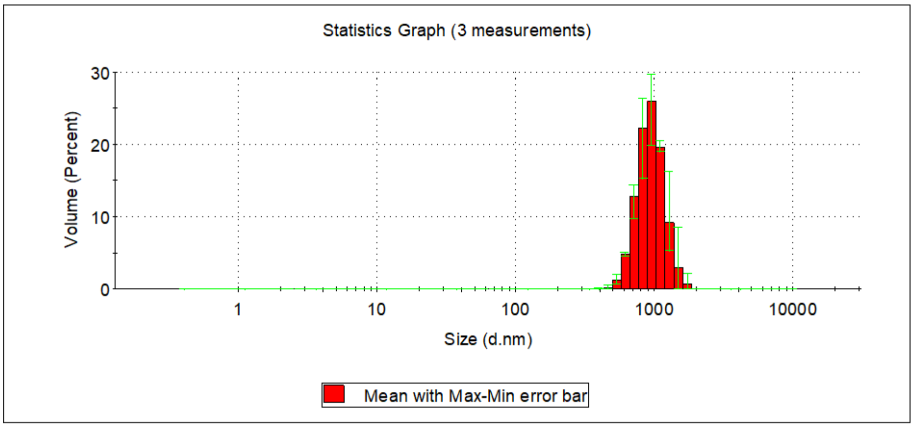

Figure S4. Size distribution of the liposomes of **RVT** based on DLS volume analysis.

Table S6. DLS analysis results for the liposomal formulation of **RMVT**.

Test	Size (d.nm)	PDI
1	935.8	0.149
2	943.1	0.224
3	971.3	0.271
**Ave**	**950.1**	**0.215**
**SD**	**18.75**	**0.062**

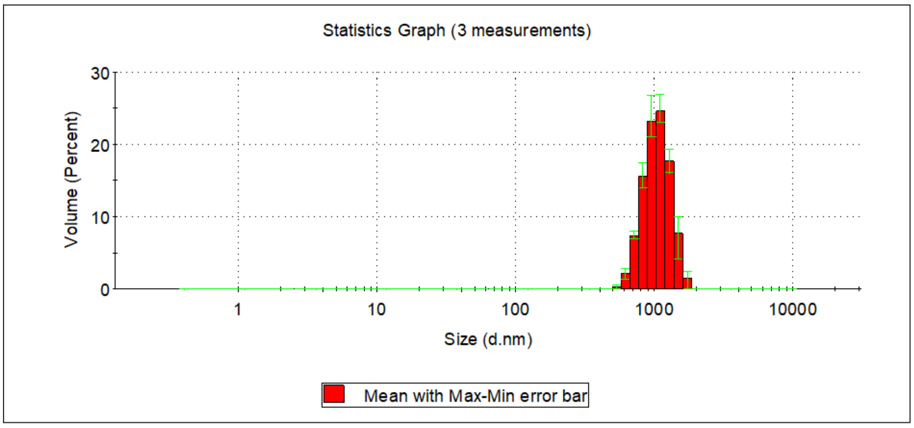

Figure S5. Size distribution of the liposomes of **RMVT** based on DLS volume analysis.

2.10 Mouse Immunization

Two groups of female Balb/c mice (n=6 per group) were primed subcutaneously with 0.1 mL of an emulsion containing 50 μg Rha-OVA and Freund's complete adjuvant, and then boosted three times at weekly intervals with the same dose of vaccine emulsified

in Freund's incomplete adjuvant. Blood samples were collected before each inoculation (D-35, D-28, D-21, D-14) and 1 or 2 weeks after the fourth inoculation (D-7, D-0) to analyze anti-Rha Abs. Thereafter, each group of female Balb/c (six mice per group) mice (6-8 weeks old) was immunized subcutaneously with 0.1 mL liposome solution containing 6 μg of Tn or an emulsion preparation of glycoprotein vaccine (1.7 μg of Tn in 0.1 mL emulsion). Then, the mice were boosted 3 times on days 14, 21, and 28 after the initial immunization by s.c. injection using the same vaccine and using the same immunization protocol. Blood samples were collected from the eye socket of each mouse on day 0 before the initial immunization and on days 21, 27, and 38 after the first injection. Then, the blood samples were clotted to obtain antisera and stored at -80 °C before use.

Table S7. Body weight of mice at days 0, 14, 21, 27, and 38 after vaccination. ($\bar{x} \pm s$, n = 6)

	Day 0	Day 14	Day 21	Day 27	Day 38
Tn-CRM197/Alum	18.8 ± 0.8	19.4 ± 0.9	21.2 ± 1.4	21.6 ± 1.1	22.7 ± 1.1
**VT**	19 ± 0.7	19.2 ± 0.7	20.6 ± 0.7	21.8 ± 1.0	23.2 ± 0.9
**MVT**	18.7 ± 1.0	19.3 ± 0.6	20.2 ± 0.9	22.1 ± 0.8	22.3 ± 1.6
**RVT**	19.1 ± 0.6	19.5 ± 0.5	20.8 ± 1.4	21.3 ± 0.7	22.7 ± 0.9
**RMVT**	18.6 ± 0.7	19.5 ± 1.1	20.9 ± 1.1	21.7 ± 1.0	22.8 ± 1.2
**RVT**/Rha Model	18.9 ± 0.9	18.9 ± 0.3	19.9 ± 1.0	21 ± 0.5	22.0 ± 1.2
**RMVT**/Rha Model	18 ± 0.5	19.2 ± 1.2	20.5 ± 0.8	21.4 ± 0.7	22.5 ± 0.7

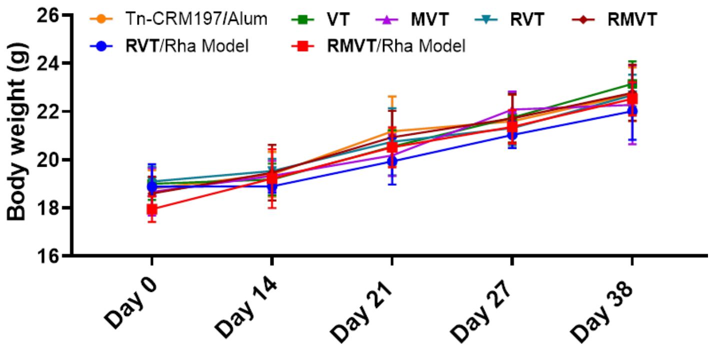

Figure S6. Body weight of mice at days 0, 14, 21, 27, and 38 after vaccination.

2.11 ELISA Protocol and Results

ELISA protocol

The conjugate of Tn-HSA (2 μg/mL, 100 μL) was dissolved in 0.1 M carbonate buffer (pH = 9.6) and then added to each well of a 96-well microtiter plate. After incubation at 4 °C overnight and followed by incubation at 37°C for 1 h, the plate was washed with PBS containing 0.1% Tween-20 (PBST) 3 times, and treated with 250 μL blocking buffer (2% skim milk in PBS) at rt for 1 h. Afterwards, a pooled sample, diluted with serial half-log dilutions from 1:300 to 1:656100 in PBS, was added to the coated plates (100 μL per well, each group set with 3 wells) and incubated at 37°C for 2 h. After washing with PBST, the plates were incubated with a 1:5000 dilution of HRP-linked goat anti-mouse IgG and IgM, and with 1:2000 dilutions of kappa, IgG1, IgG2a, IgG2b, and IgG3 antibodies. The plates were shaken at 400 rpm for 1 h, and subsequently washed with PBST. A 100 μL colorimetric substrate 3,3',5,5'-tetramethylbenzidine (TMB) solution was added to the plates and incubated in the dark for 20 min. Finally, 100 μL of 0.5 M H_2SO_4 solution was added, and the optical density (OD) was measured using a microplate reader at 450 nm with 570 nm as the reference. For titer analysis, the best-fit line was obtained with OD as the ordinate and the natural logarithm of the serum dilutions as the abscissa. The antibody titer was defined as the dilution at which the OD was 0.2.

Results of antibody titers

Table S8. Rha-specific IgG antibody titers of pooled sera collected from mice on days -35, -28, -21, -14, -7 and 0 after immunization with Rha-OVA. ($\bar{x} \pm s$, n = 3)

	Group 6	Group 7
Day -35	7266 ± 1278	3029 ± 603
Day -28	5459 ± 241	5335 ± 280
Day -21	39020 ± 4812	52282 ± 1380
Day -14	112162 ± 703	157509 ± 6658
Day -7	216679 ± 12432	245328 ± 2825
Day 0	189932 ± 10826	205037 ± 5636

Table S9. Rha-specific IgG antibody titers in pooled sera collected on days 21, 27, and 38 from mice immunized with **VT**, **RVT**, and **RMVT**. ($\bar{x} \pm s$, n = 3)

	Day 21	Day 27	Day 38
**VT**	1589 ± 670	1103 ± 635	1491 ± 539
**RVT**	22370 ± 687	33632 ± 1721	49149 ± 2302
**RMVT**	25131 ± 2054	40430 ± 1158	55819 ± 4107

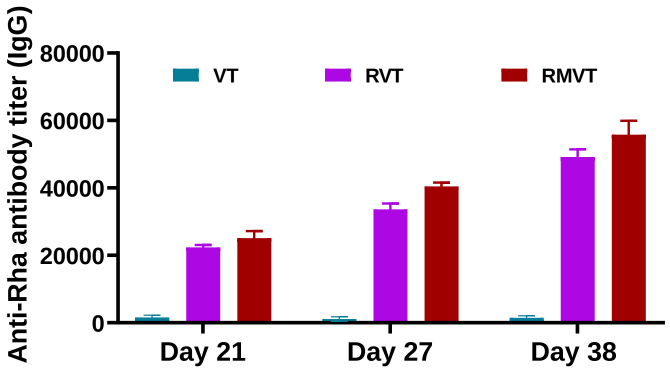

Figure S7. Rha-specific IgG antibody titers in pooled sera collected on days 21, 27, and 38 from mice immunized with **VT**, **RVT**, and **RMVT**.

Table S10. IgM antibody titers of pooled sera collected from mice on days 21, 27, and 38 after immunization with conjugate vaccines. ($\bar{x} \pm s$, n = 3)

	Day 21	Day 27	Day 38
Tn-CRM197/Alum	40554 ± 1006	41554 ± 532	38235 ± 3574
**VT**	65440 ± 3041	23042 ± 936	30134 ± 3087
**MVT**	59770 ± 3125	30752 ± 502	28672 ± 1197
**RVT**	66244 ± 4176	38175 ± 238	35524 ± 1013
**RMVT**	73678 ± 1096	41941 ± 1785	45341 ± 1777
**RVT**/Rha Model	75915 ± 902	33576 ± 2237	50377 ± 1547
**RMVT**/Rha Model	73358 ± 2247	34950 ± 2057	48202 ± 989

Table S11. IgG antibody titers of pooled sera collected from mice on days 21, 27, and 38 after immunization with conjugate vaccines. ($\bar{x} \pm s$, n = 3)

	Day 21	Day 27	Day 38
Tn-CRM197/Alum	14484 ± 1701	36607 ± 568	53851 ± 676
**VT**	14176 ± 2055	46664 ± 2075	58624 ± 3409
**MVT**	25395 ± 2357	55592 ± 3771	78515 ± 271
**RVT**	28188 ± 1287	69593 ± 4022	114639 ± 4960
**RMVT**	29738 ± 2894	86441 ± 835	133173 ± 5059
**RVT**/Rha Model	100214 ± 979	121533 ± 1608	154301 ± 6105
**RMVT**/Rha Model	115656 ± 1258	137187 ± 4122	217097 ± 11415

Table S12. The antibody titers of kappa and IgG isotypes in antiserum collected from mice 38 days after immunization with conjugate Tn-CRM197/Alum.

Kappa	IgG1	IgG2a	IgG2b	IgG3
61108 ± 4827	79616 ± 2390	18119 ± 7583	40135 ± 4774	17471 ± 7407

Table S13. The antibody titers of kappa and IgG isotypes in antiserum collected from mice 38 days after immunization with **VT**.

Kappa	IgG1	IgG2a	IgG2b	IgG3
55500 ± 3579	35714 ± 4089	28275 ± 6260	36273 ± 5847	17351 ± 5766

Table S14. The antibody titers of kappa and IgG isotypes in antiserum collected from mice 38 days after immunization with **MVT**.

Kappa	IgG1	IgG2a	IgG2b	IgG3
64845 ± 1607	84946 ± 3678	76498 ± 406	74596 ± 2291	12160 ± 7490

Table S15. The antibody titers of kappa and IgG isotypes in antiserum collected from mice 38 days after immunization with **RVT**.

Kappa	IgG1	IgG2a	IgG2b	IgG3
77179 ± 2837	72950 ± 3798	55031 ± 1074	67187 ± 1340	34250 ± 6952

Table S16. The antibody titers of kappa and IgG isotypes in antiserum collected from mice 38 days after immunization with **RMVT**.

Kappa	IgG1	IgG2a	IgG2b	IgG3
91960 ± 4682	85935 ± 4197	50041 ± 3022	78481 ± 4212	29709 ± 5230

Table S17. The antibody titers of kappa and IgG isotypes in antiserum collected from Rha-OVA pre-immunized mice 38 days after immunization with **RVT**.

Kappa	IgG1	IgG2a	IgG2b	IgG3
110397 ± 6635	77426 ± 1154	46334 ± 2440	95991 ± 4571	18404 ± 3376

Table S18. The antibody titers of kappa and IgG isotypes in antiserum collected from Rha-OVA pre-immunized mice 38 days after immunization with **RMVT**.

Kappa	IgG1	IgG2a	IgG2b	IgG3
127263 ± 1119	131254 ± 6795	84327 ± 8374	167230 ± 10229	19803 ± 4798

2.12 Results of IFN-γ and IL-4 Levels Provoked by Conjugates

Table S19. IFN-γ and IL-4 in sera collected from normal mice or mice 38 days after immunized with Tn-CRM197/Alum **VT**, **MVT**, **RVT**, and **RMVT**. ($\bar{x} \pm s$, n = 3)

	IFN-γ	IL-4
NS	10.98 ± 1.69	11.36 ± 0.53
Tn-CRM197/Alum	24.13 ± 5.81	56.59 ± 15.5
**VT**	35.93 ± 1.29	30.93 ± 2.42
**MVT**	42.10 ± 2.84	60.96 ± 6.40
**RVT**	42.18 ± 2.44	55.35 ± 7.15
**RMVT**	46.36 ± 3.82	65.96 ± 3.91
**RVT**/Rha Model	57.93 ± 7.61	101.77 ± 6.49
**RMVT**/Rha Model	96.32 ± 7.08	137.07 ± 6.32

2.13 FACS Analyses

The cell samples used for FACS analyses were prepared similarly to the reported protocol. MCF-7 and MDA231 cancer cells were incubated in DMEM containing 10% FBS. TA3Ha cancer cells were incubated in MEM containing 10% FBS. The cells were harvested using trypsin-EDTA. A suspension of 2.0×10^5 target cells was incubated with 50 μL 1:10 dilution of normal mouse serum or antiserum at 4 ºC for 1 h. After washing with FACS buffer 3 times, 50 μL 1:50 dilution of FITC-labeled goat anti-mouse IgG antibody was added to the cell suspension and incubated at 4 ºC for 1 h. The resulting cells were collected and washed with FACS buffer 3 times. Percent positive cells and MFI of stained cells were recorded using a FACS flow cytometer. Data were processed and analyzed with FlowJo software.

Table S20. Mean fluorescence intensities for binding analysis of sera derived from mice immunized with Tn-CRM197/Alum **VT**, **MVT**, **RVT**, and **RMVT**. ($\bar{x} \pm s$, n = 3)

	MCF-7	TA3Ha	MDA231
NS	1936 ± 308	8876 ± 303	13063 ± 302
Tn-CRM197/Alum	3033 ± 130	22678 ± 181	12326 ± 206
**VT**	3843 ± 63	28276 ± 124	13107 ± 299
**MVT**	4065 ± 48	34748 ± 201	14675 ± 25
**RVT**	4792 ± 61	37211 ± 211	12596 ± 196
**RMVT**	5577 ± 85	40719 ± 659	12143 ± 220
**RVT**/Rha Model	6182 ± 128	41835 ± 110	13801 ± 230
**RMVT**/Rha Model	6619 ± 439	50974 ± 588	15363 ± 369

2.14 Complement-Dependent Cytotoxicity (CDC)

CDC was determined using a commercially available LDH cytotoxicity detection kit following the manufacturer's instructions. MCF-7, TA3Ha, or MDA231 cancer cells (1.0×10^4 cells per well) were seeded in a 96-well plate and incubated at 37ºC overnight. After washing, the plates were incubated with 100 μL of 1:10 dilution normal mouse serum or a day 38 antiserum at 37°C for 2 h. The wells were washed twice and then incubated with 100 μL of 1:10 dilution of rabbit complement serum at 37ºC for 1 h. For low control (spontaneous LDH release), no antiserum was added. For high control (maximum LDH release), rabbit complement serum was replaced with 100 μL of 5% Triton X-100. After incubation, 20 μL of supernatant from each well was carefully transferred to another 96-well plate containing 80 μL of DPBS. Then, 100 μL of the

LDH cytotoxicity detection reagent was added to each well, and the plate was incubated in the dark for 30 min. The optical absorption (A) of each well was read at 490 nm wavelength using a microplate reader. The percentage of cell lysis was calculated according to the following equation:

$$Cell\ lysis\ \% = \frac{experimental\ A\ -\ low\ control\ A}{high\ control\ A - low\ control\ A} \times 100\%$$

Where "*experimental A*" is the optical absorption at 490 nm of cells lysed by treatment with antiserum, "*low control A*" is the optical absorption of cells lysed without serum treatment, and "*high control A*" is the optical absorption of cells lysed with 5% Triton X-100 solution.

Table S21. Lysis of MCF-7, TA3Ha, or MDA231 cancer cells through antibody-mediated CDC. ($\bar{x} \pm s$, n = 6)

	MCF-7	TA3Ha	MDA231
NS	14.09 ± 3.84	9.24 ± 2.14	4.47 ± 3.85
Tn-CRM197/Alum	55.49 ± 9.30	27.10 ± 3.57	4.12 ± 2.42
**VT**	49.13 ± 7.89	21.99 ± 3.72	5.28 ± 3.62
**MVT**	57.47 ± 5.37	32.91 ± 4.12	10.63 ± 7.10
**RVT**	56.84 ± 3.97	29.71 ± 2.15	9.87 ± 5.16
**RMVT**	64.96 ± 6.70	33.30 ± 1.84	12.27 ± 3.52
**RVT**/Rha Model	69.77 ± 6.85	33.69 ± 3.18	5.88 ± 4.97
**RMVT**/Rha Model	73.52 ± 4.83	48.19 ± 2.42	10.51 ± 6.47

2.15 Antibody-dependent cell-mediated cytotoxicity assay (ADCC)

NK cells were isolated and enriched by magnetic sorting using an NK cell isolation kit according to the manufacturer's instructions. TA3Ha and B16F10 cells were seeded into 96-well plates at a density of 1×10^4 cells per well (50 μL/well) and incubated overnight. Subsequently, sera from immunized mice were added (50 μL/well), followed by NK cells at 2.5×10^4 cells per well (100 μL/well). For the low control (spontaneous LDH release), an equal volume of medium was added instead of immune serum and NK cells. For the high control (maximum LDH release), an equal volume of 1% Triton X-100 was added to tumor cells without immune serum and NK cells. After 12 h of incubation, the 96-well plates were centrifuged. Then, 20 μL of cell supernatant was collected from each well, transferred to a flat-bottom 96-well assay plate, and mixed

with LDH assay enzyme cocktail. The plate was incubated on a shaker for 30–40 min, and the absorbance (A) of each well was measured at 490 nm using a microplate reader. The percentage of specific cell lysis was calculated according to the following equation:

$$Cell\ lysis\ \% = \frac{experimental\ A\ -\ low\ control\ A}{high\ control\ A - low\ control\ A} \times 100\%$$

Where "*experimental A*" is the optical absorption at 490 nm of cells lysed by treatment with antiserum, "*low control A*" is the optical absorption of cells lysed without serum treatment, and "*high control A*" is the optical absorption of cells lysed with 1% Triton X-100 solution.

Table S22. Lysis of TA3Ha, or B16F10 cancer cells through antibody-mediated ADCC. ($\bar{x} \pm s$, n = 6)

	TA3Ha	B16F10
NS	7.38 ± 1.25	6.07 ± 1.77
Tn-CRM197/Alum	14.87 ± 1.02	7.37 ± 3.18
**VT**	14.66 ± 0.95	6.03 ± 1.54
**MVT**	17.69 ± 2.95	6.57 ± 1.99
**RVT**	17.25 ± 1.94	7.58 ± 2.25
**RMVT**	23.02 ± 2.11	6.54 ± 2.27
**RVT**/Rha Model	30.21 ± 1.03	6.84 ± 2.02
**RMVT**/Rha Model	37.20 ± 2.71	8.48 ± 2.40

2.16 Tumor Challenge Study

Eight groups of 6- to 8-week-old female Balb/c mice (n = 10 per group) were used to evaluate the inhibitory effect against TA3Ha tumors. Fifty Balb/c mice were randomly allocated into five groups: PBS, PBS/CP, Tn-CRM197/Alum/CP, **VT**/CP, and **MVT**/CP. Additionally, thirty Balb/c mice pre-immunized with high-titer anti-Rha antibodies were randomly assigned to three groups: Rha-OVA/CP, **RVT**/Rha Model/CP, and **RMVT**/Rha Model/CP On day 0, all mice were intraperitoneally inoculated with TA3Ha cancer cells (5.0×10^4 cells). One day before vaccine inoculation (day 1), the seven groups designated with "CP" received a low dose of cyclophosphamide (50 mg/kg, i.p.) to deplete regulatory T cells; the PBS group received no CP. The first immunization was administered on day 2. The PBS and PBS/CP groups received PBS, while the other six groups received their respective vaccine formulations (Tn-

CRM197/Alum, **VT**, **MVT**, **RVT**, or **RMVT**) via subcutaneous injection. Booster vaccinations were administered on days 5, 7, and 11 using the same protocol. Mouse survival was monitored for 50 days following tumor challenge. Surviving mice were rechallenged with an additional dose of TA3Ha cells (5.0×10^4 cells) on day 50, and survival was recorded for another 50 days.

Table S23. Survival time after 1st tumor challenge (day).

Mouse	1	2	3	4	5	6	7	8	9	10
PBS	10	13	14	15	15	16	16	16	17	18
PBS/CP	14	15	16	16	17	17	18	18	21	24
Rha-OVA/CP	14	15	15	16	16	17	18	18	18	18
Tn-CRM197/Alum/CP	14	16	18	19	20	24	24	27	>50	>50
**VT**/CP	18	19	20	21	24	24	30	31	32	>50
**MVT**/CP	18	18	20	21	25	32	33	>50	>50	>50
**RVT**/Rha Model/CP	18	18	23	24	24	30	>50	>50	>50	>50
**RMVT/**Rha Model/CP	24	27	30	32	>50	>50	>50	>50	>50	>50

Table S24. Survival time after 2nd tumor challenge (day).

Mouse	1	2	3	4	5	6
Tn-CRM197/Alum/CP (n=2)	>50	>50	/	/	/	/
**VT**/CP (n=1)	>50	/	/	/	/	/
**MVT**/CP (n=3)	17	>50	>50	/	/	/
**RVT**/Rha Model/CP (n=4)	27	>50	>50	>50	/	/
**RMVT/**Rha Model/CP (n=6)	26	30	>50	>50	>50	>50

Table S25. Analysis of IgG antibody in the serum of tumor-bearing mice by ELISA. ($\bar{x} \pm s$, n = 3)

	Mean ± SD
NS	5075 ± 1263
Tn-CRM197/Alum/CP live	75633 ± 8042
Tn-CRM197/Alum/CP dead	17656 ± 543
**VT**/CP live	87494 ± 1251
**VT**/CP dead	18101 ± 1525
**MVT**/CP live	125275 ± 5369
**MVT**/CP dead	44445 ± 2408
**RVT**/Rha Model/CP live	150338 ± 4947
**RVT**/Rha Model/CP dead	61061 ± 2590
**RMVT**/Rha Model/CP live	221565 ± 12587
**RMVT**/Rha Model/CP dead	71468 ± 6334

Table S26. Mean FITC-A for binding analysis of sera from live or dead mice immunized with vaccines to TA3Ha tumor cells. ($\bar{x} \pm s$, n = 3)

	Mean±SD
NS	7148 ± 627
Tn-CRM197/Alum/CP live	13466 ± 77
Tn-CRM197/Alum/CP dead	30493 ± 352
**VT**/CP live	17859 ± 209
**VT**/CP dead	59840 ± 460
**MVT**/CP live	25140 ± 200
**MVT**/CP dead	62043 ± 1110
**RVT**/Rha Model/CP live	23416 ± 656
**RVT**/Rha Model/CP dead	82440 ± 686
**RMVT**/Rha Model/CP live	34638 ± 424
**RMVT**/Rha Model/CP dead	108644 ± 887

3. NMR and MS Spectra of New Compounds

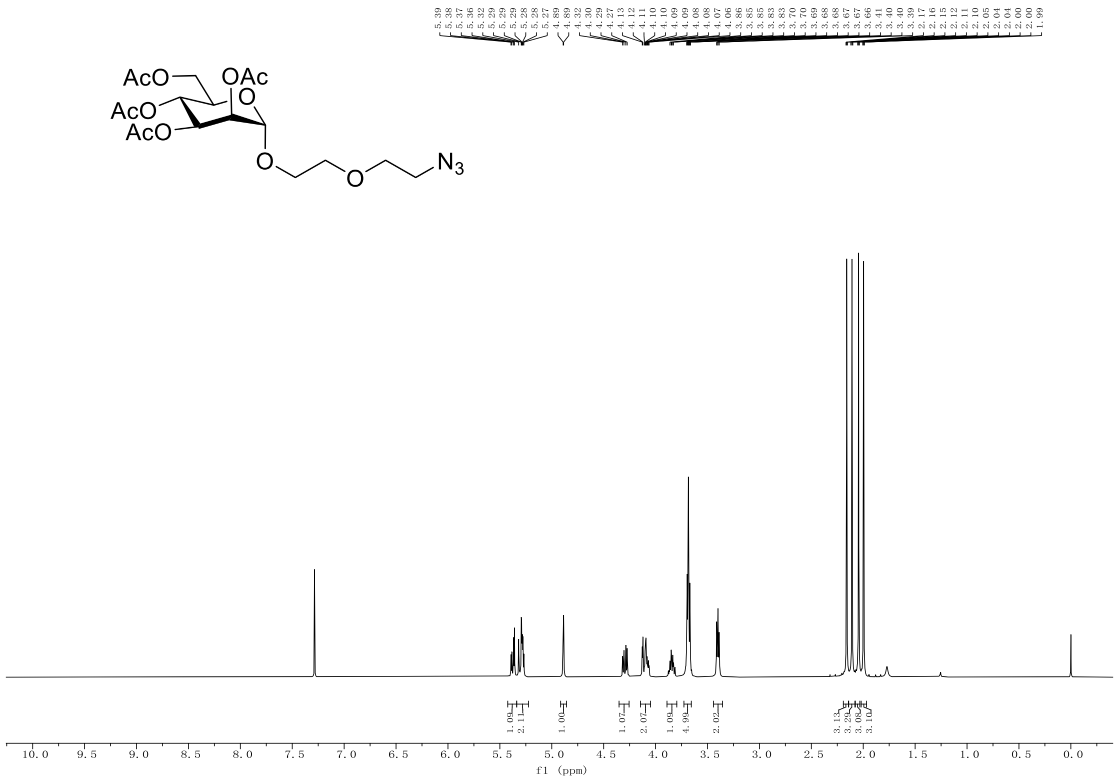

Figure S8. ^{1}H NMR (400 MHz, $CDCl_3$) spectrum of compound **11**.

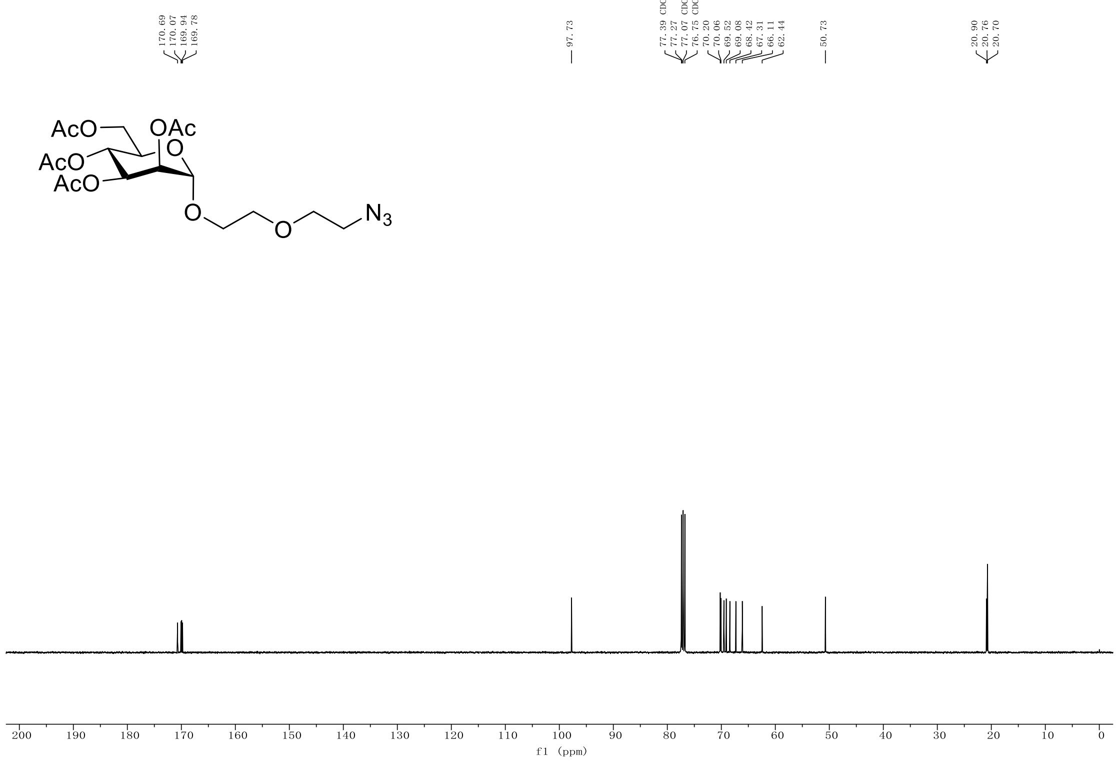

Figure S9. $^{13}C\{^{1}H\}$ NMR (101 MHz, $CDCl_3$) spectrum of compound **11**.

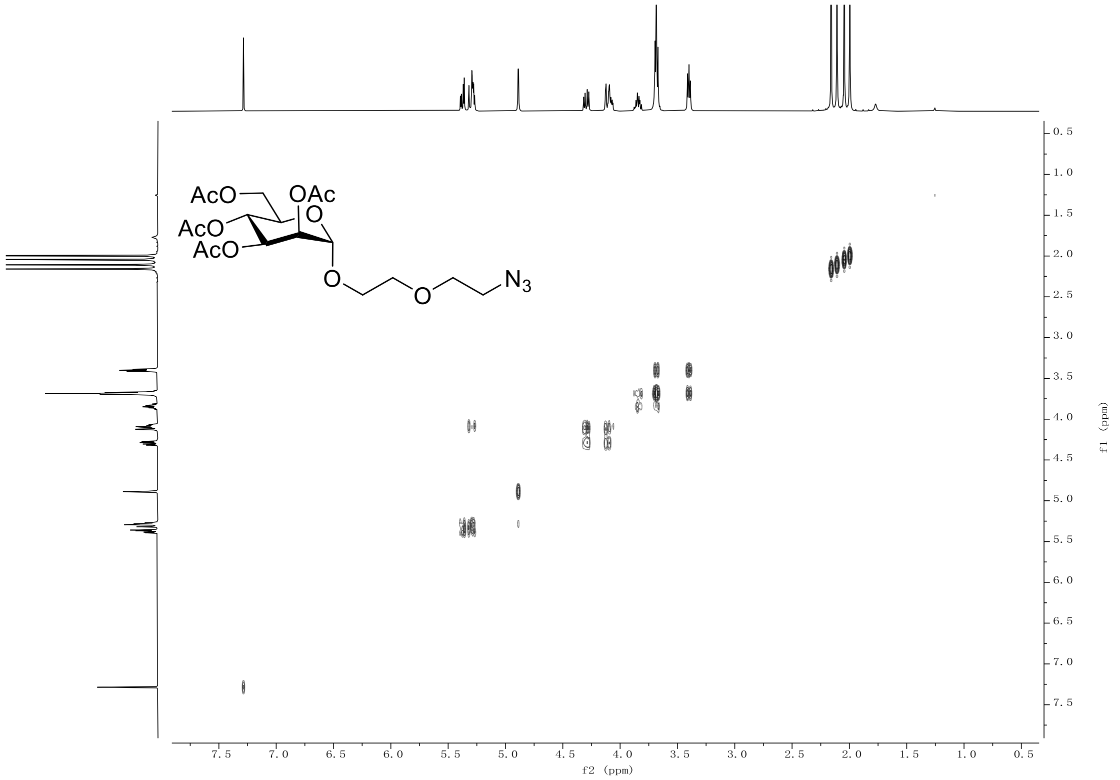

Figure S10. ^{1}H-^{1}H COSY NMR (400 MHz, $CDCl_3$) spectrum of compound **11**.

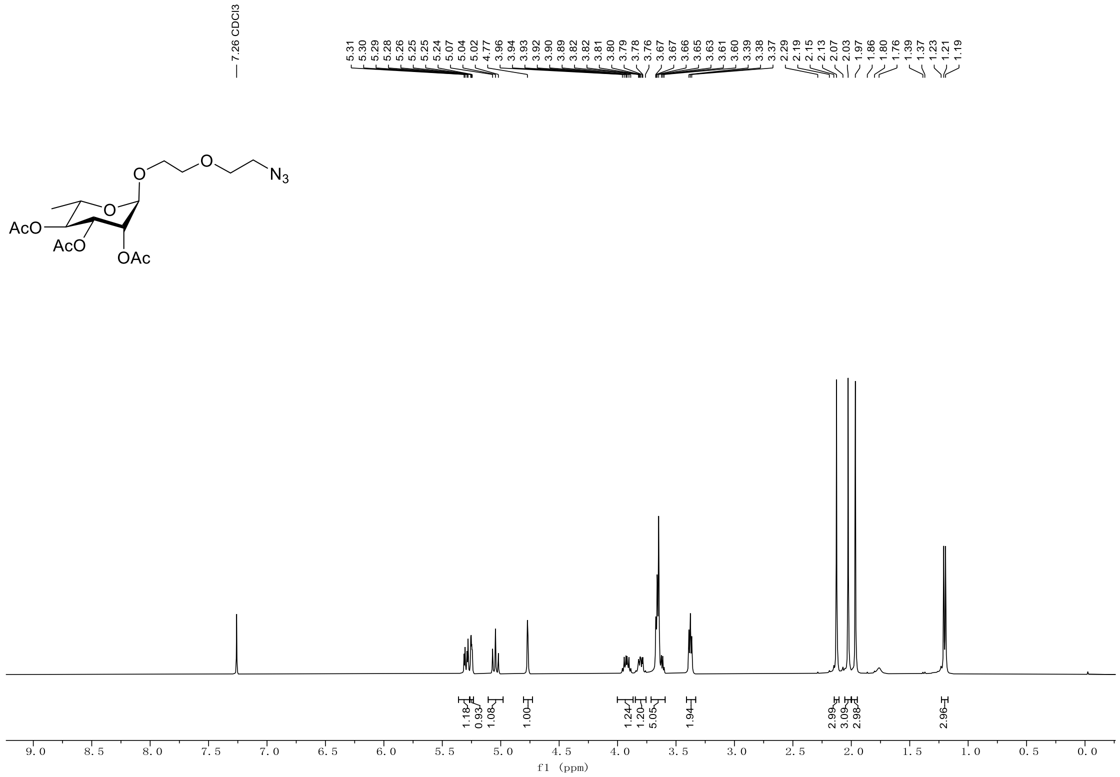

Figure S11. ^{1}H NMR (400 MHz, $CDCl_3$) spectrum of compound **13**.

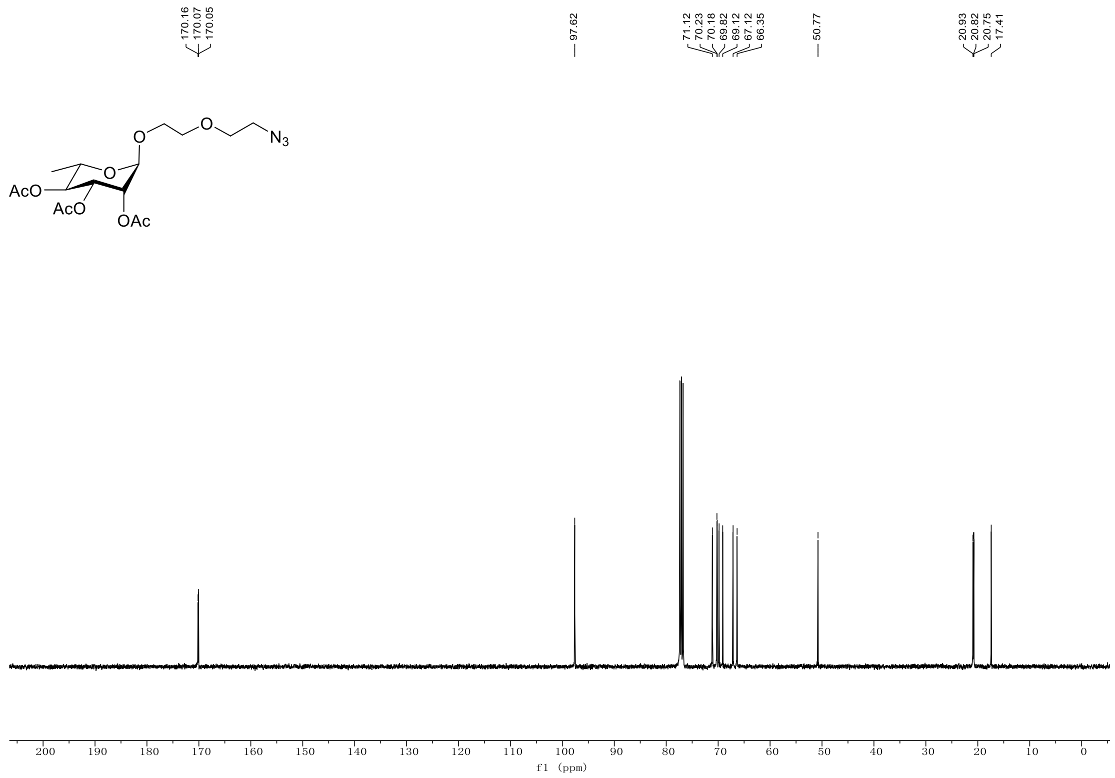

Figure S12. $^{13}C\{^{1}H\}$ NMR (101 MHz, $CDCl_3$) spectrum of compound **13**.

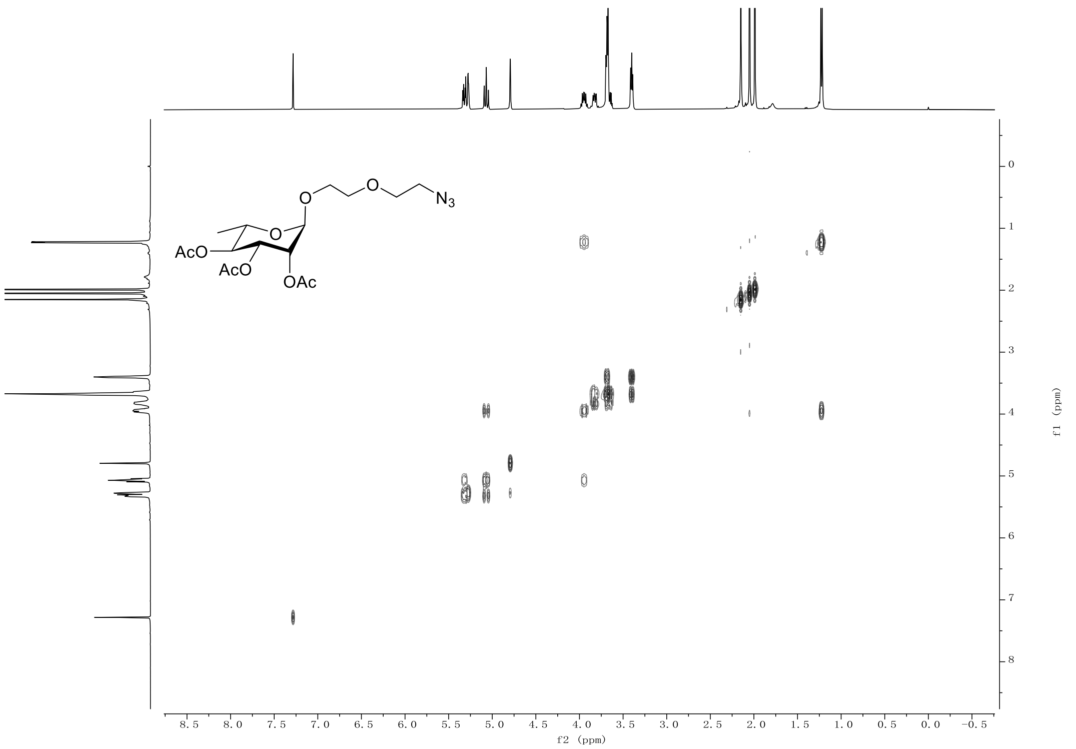

Figure S13. ^{1}H-^{1}H COSY NMR (400 MHz, $CDCl_3$) spectrum of compound **13**.

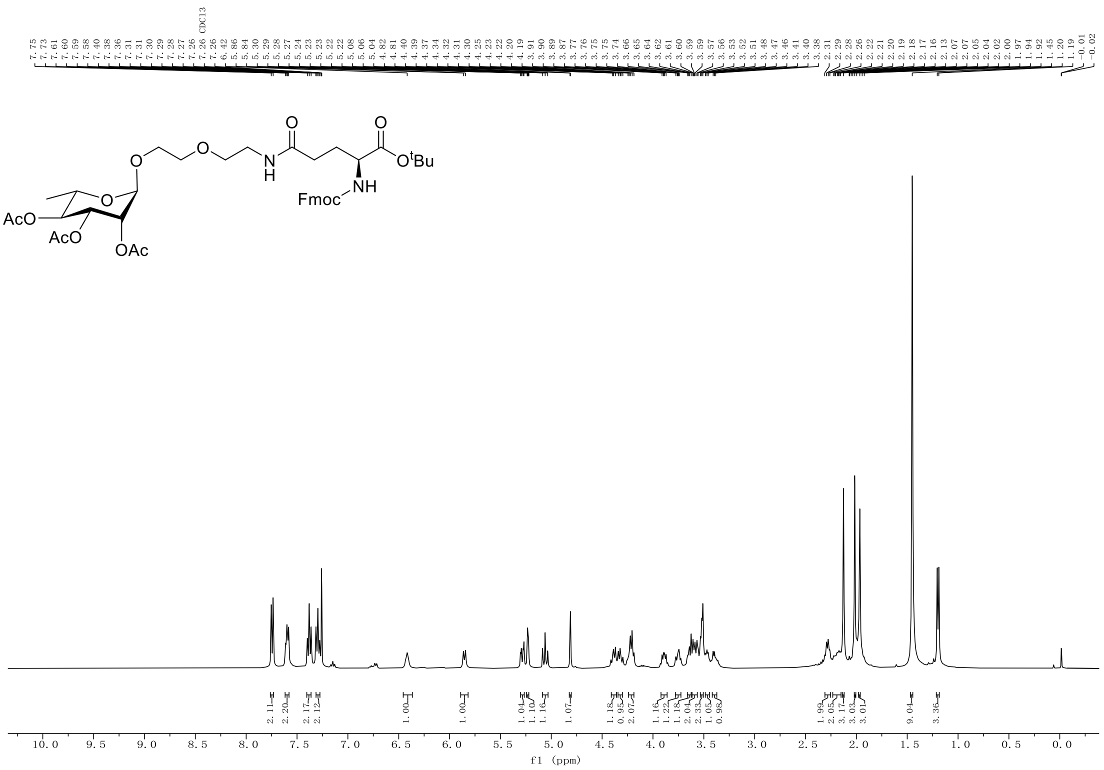

Figure S14. ^{1}H NMR (400 MHz, $CDCl_3$) spectrum of compound **14**.

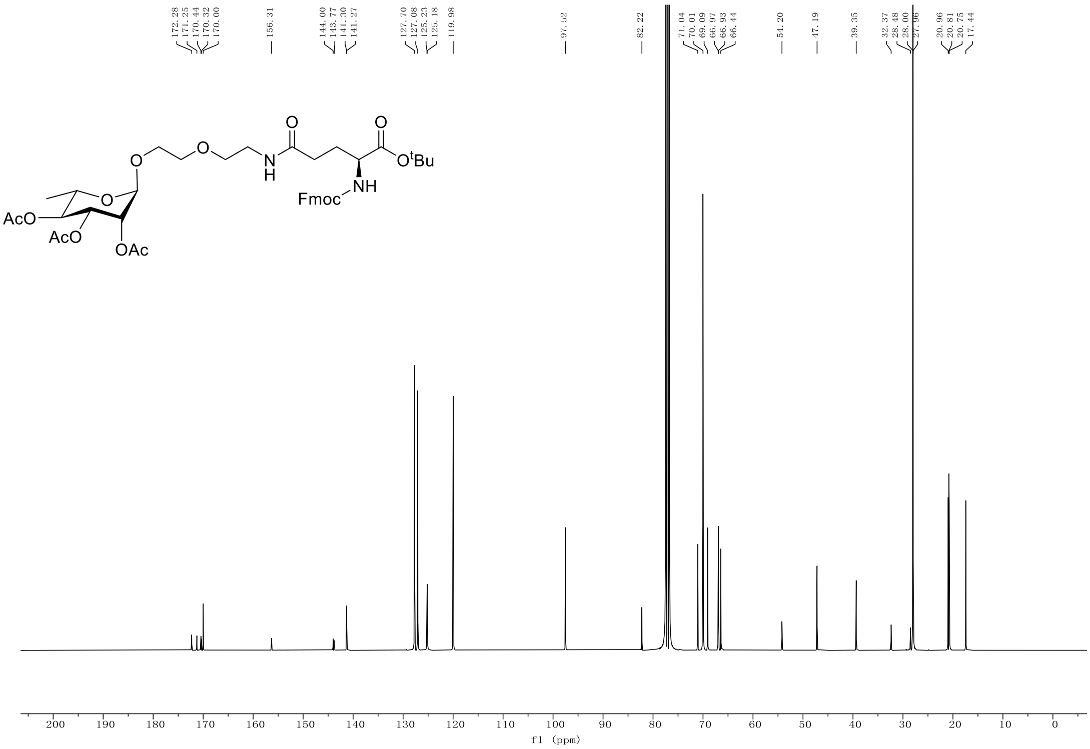

Figure S15. $^{13}C\{^{1}H\}$ NMR (101 MHz, $CDCl_3$) spectrum of compound **14**.

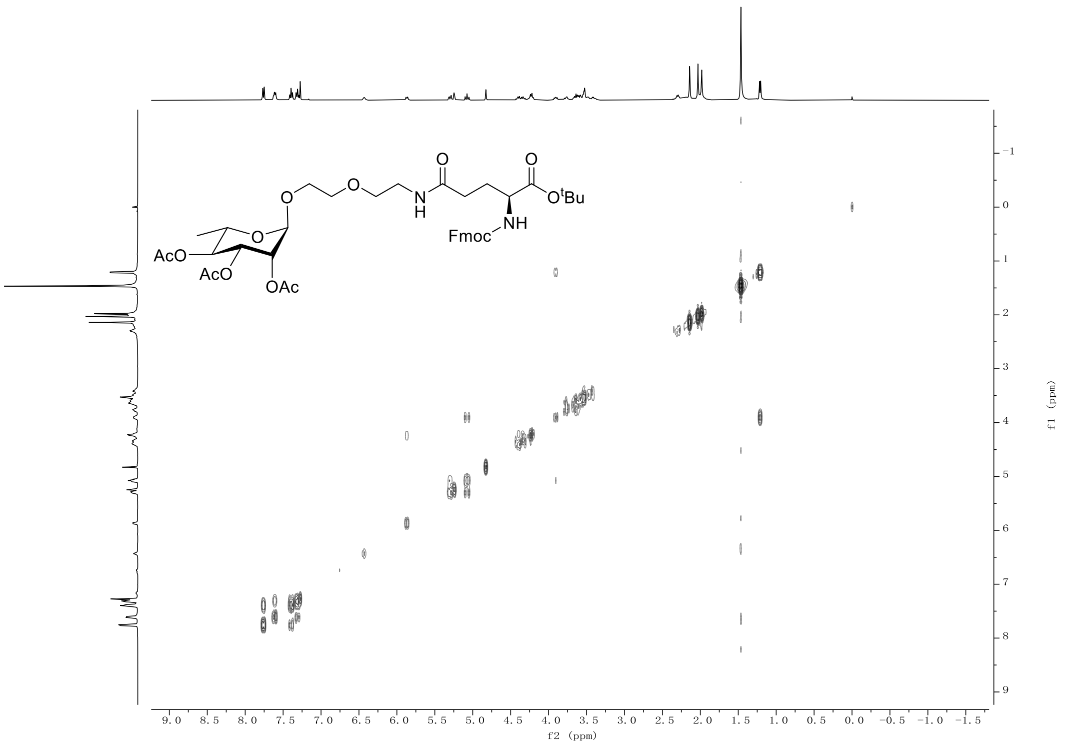

Figure S16. ^{1}H-^{1}H COSY NMR (400 MHz, $CDCl_3$) spectrum of compound **14**.

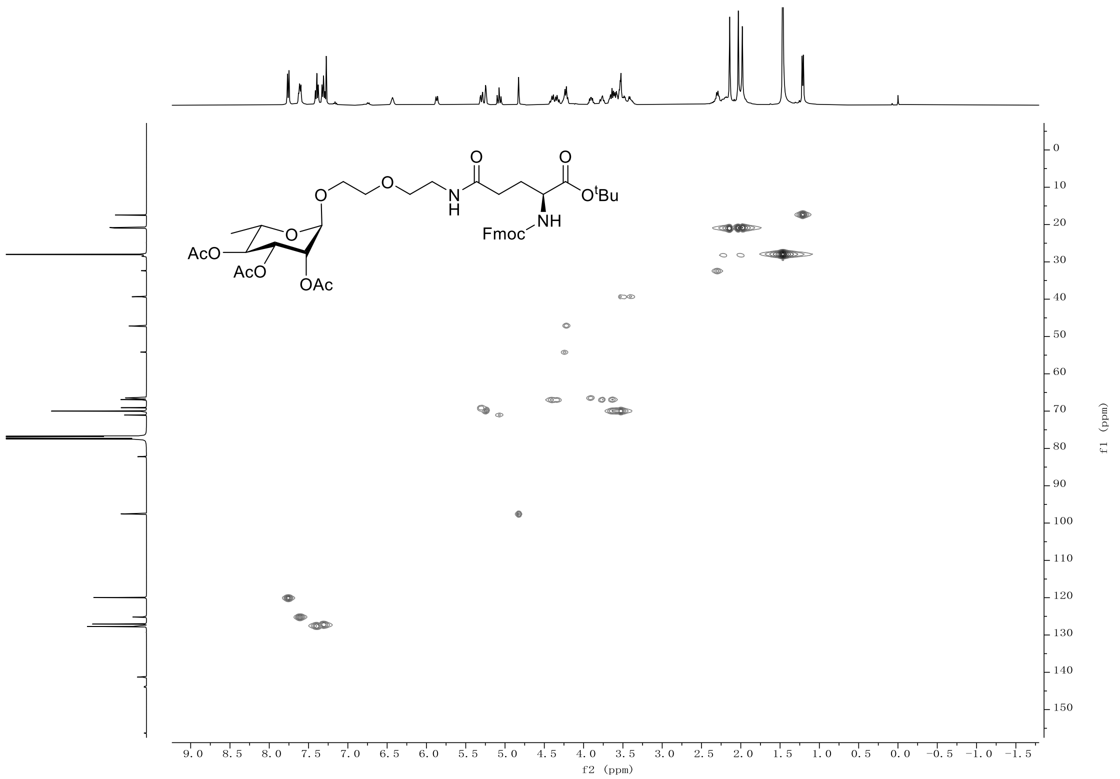

Figure S17. HSQC NMR (400/101 MHz, $CDCl_3$) spectrum of compound **14**.

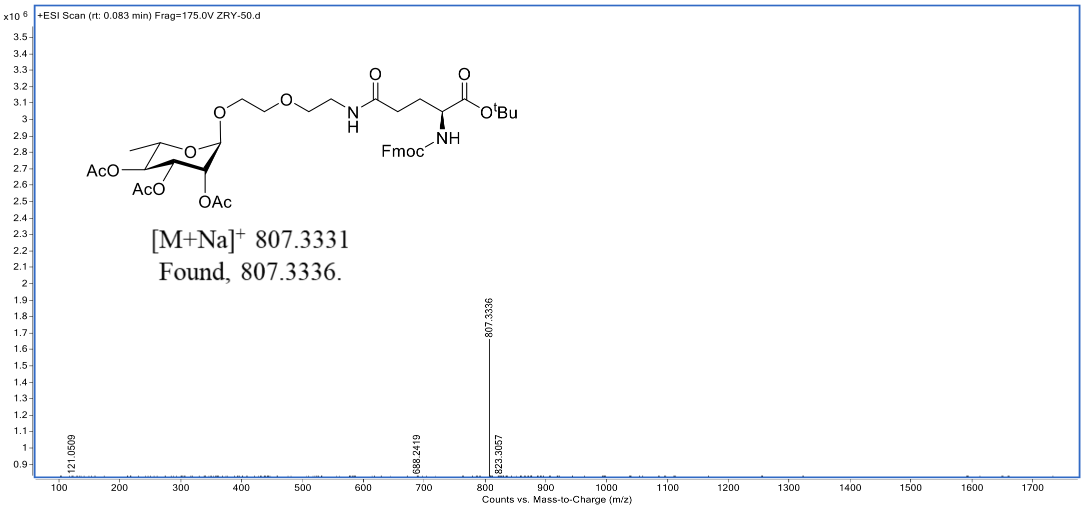

Figure S18. HRMS (ESI) spectrum of compound **14**.

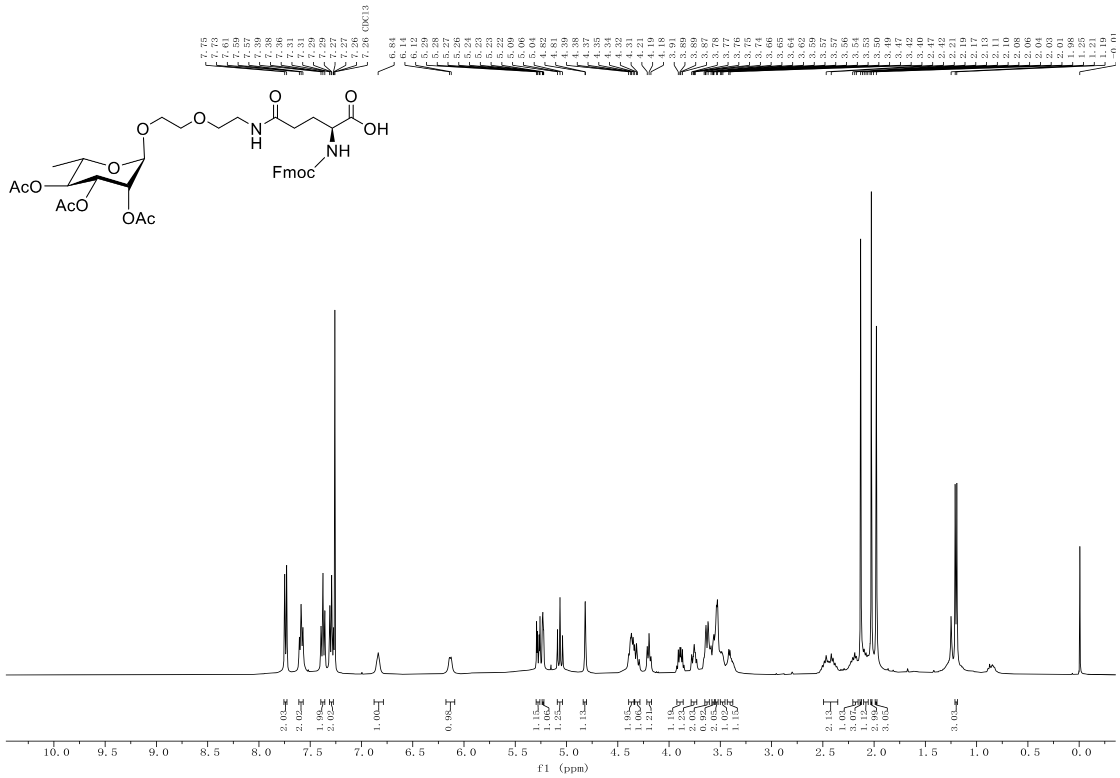

Figure S19. ^{1}H NMR (400 MHz, $CDCl_3$) spectrum of compound **15**.

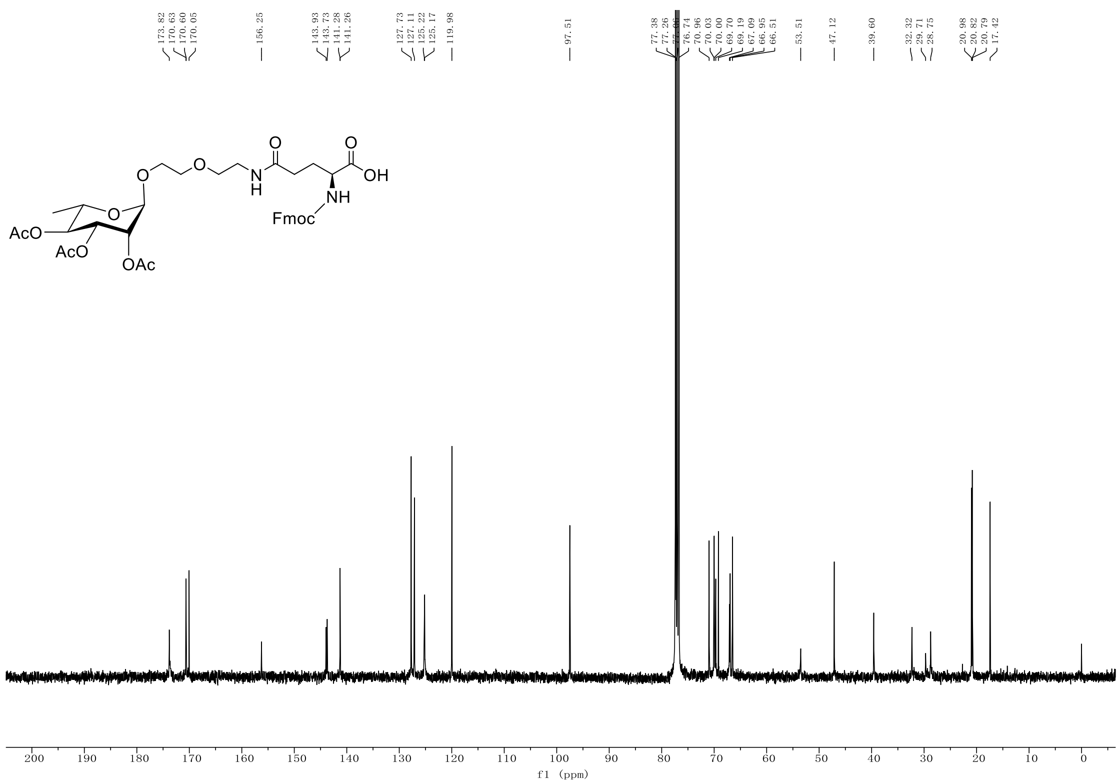

Figure S20. $^{13}C\{^1H\}$ NMR (101 MHz, $CDCl_3$) spectrum of compound **15**.

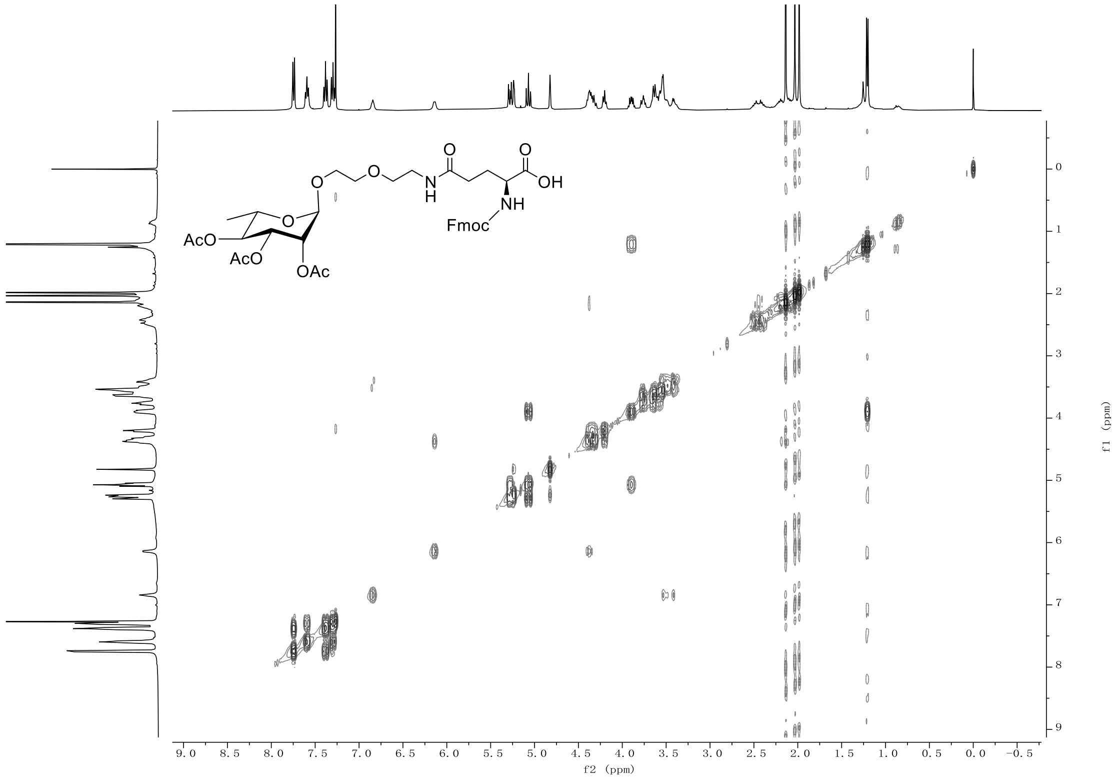

Figure S21. ^1H-^1H COSY NMR (400 MHz, $CDCl_3$) spectrum of compound **15**.

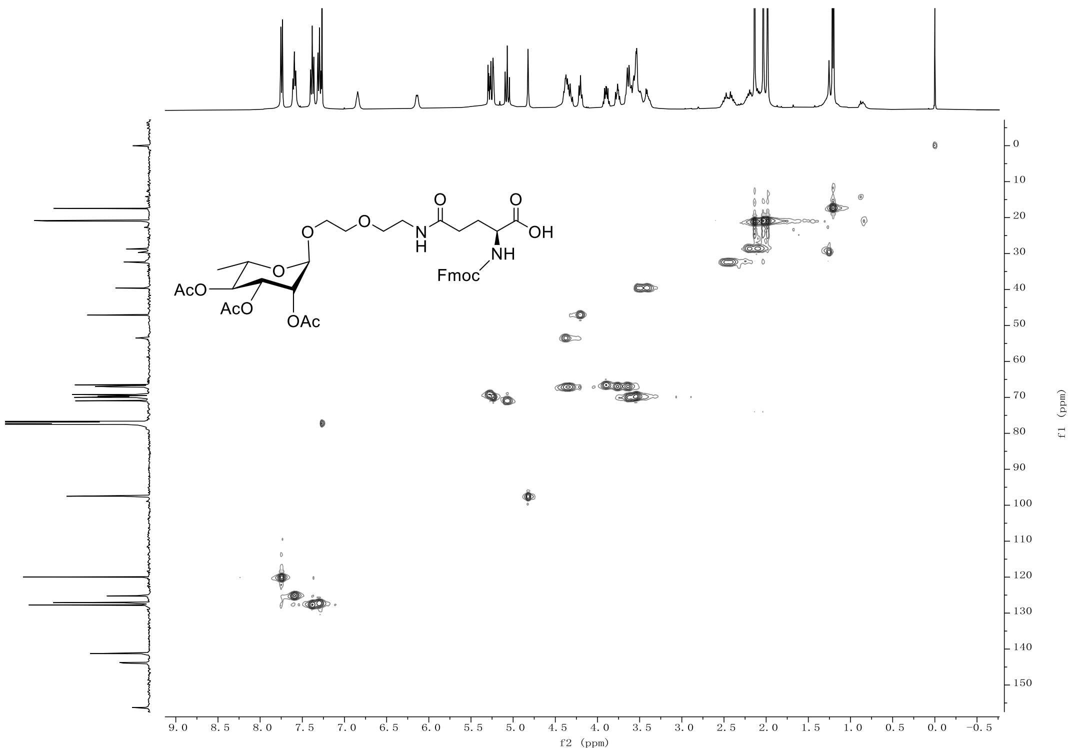

Figure S22. HSQC NMR (400/101 MHz, $CDCl_3$) spectrum of compound **15**.

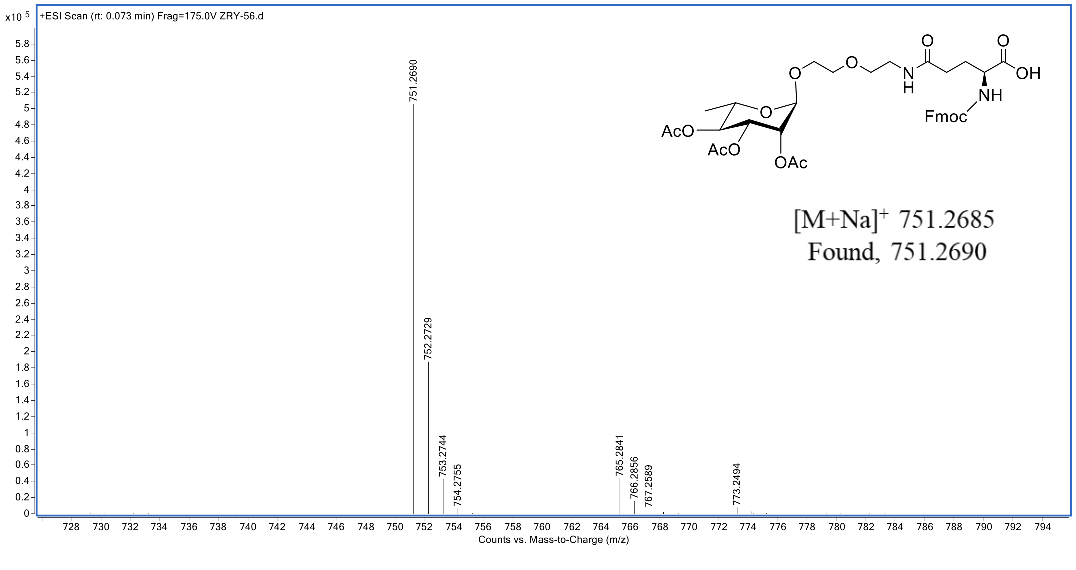

Figure S23. HRMS (ESI) spectrum of compound **15**.

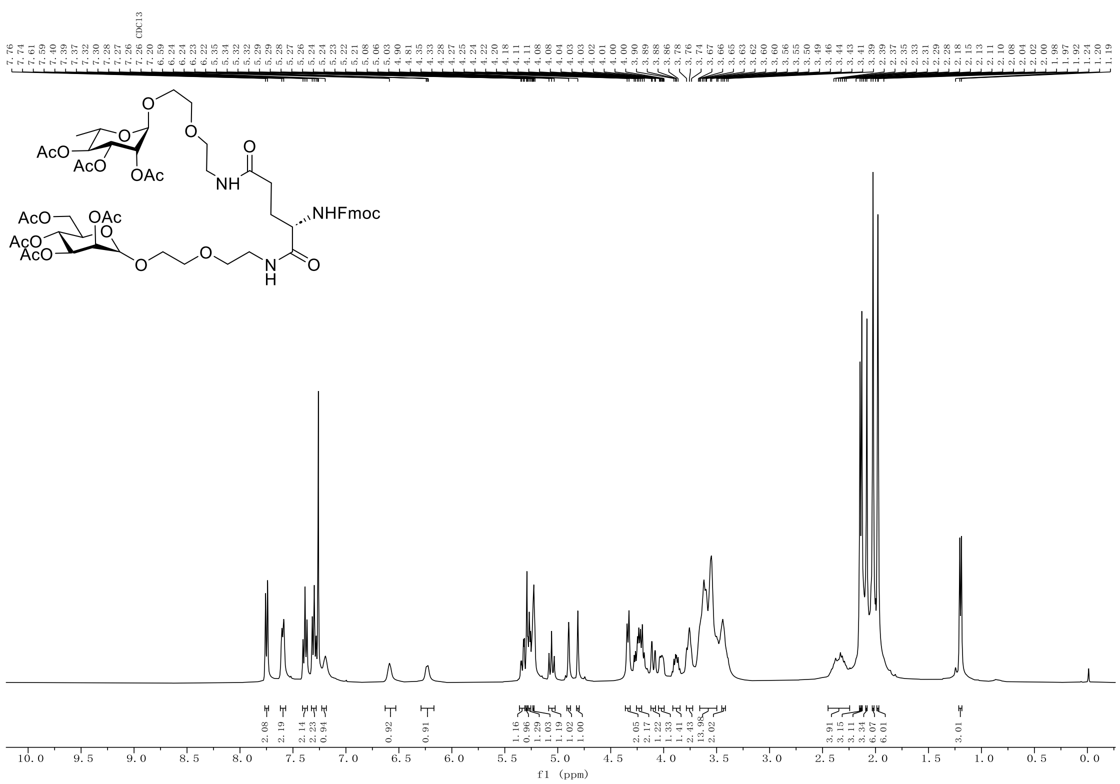

Figure S24. ^{1}H NMR (400 MHz, $CDCl_3$) spectrum of compound **16**.

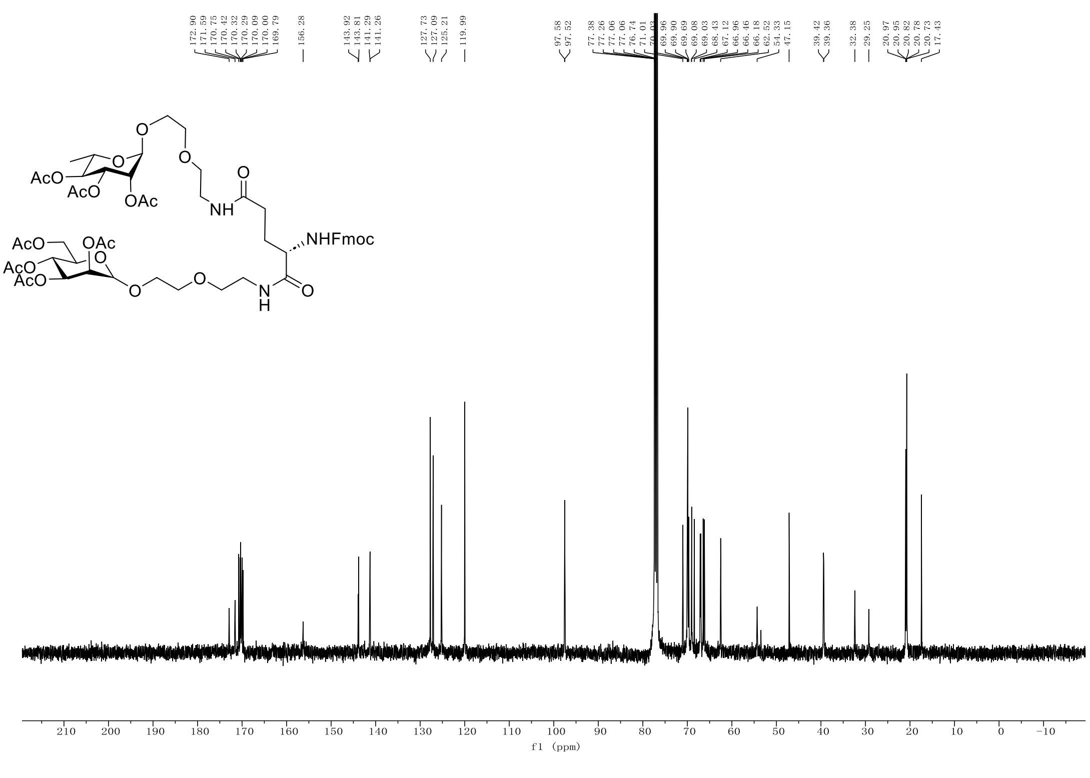

Figure S25. $^{13}C\{^{1}H\}$ NMR (101 MHz, $CDCl_3$) spectrum of compound **16**.

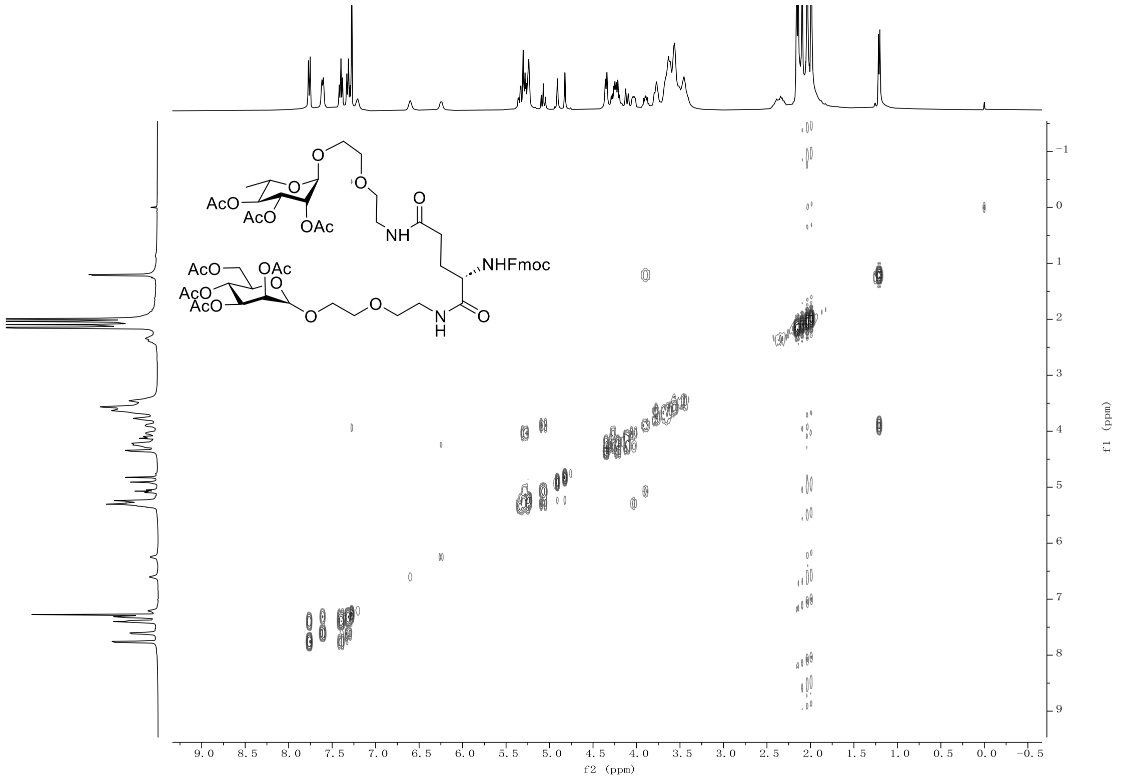

Figure S26. ^{1}H-^{1}H COSY NMR (400 MHz, $CDCl_3$) spectrum of compound **16**.

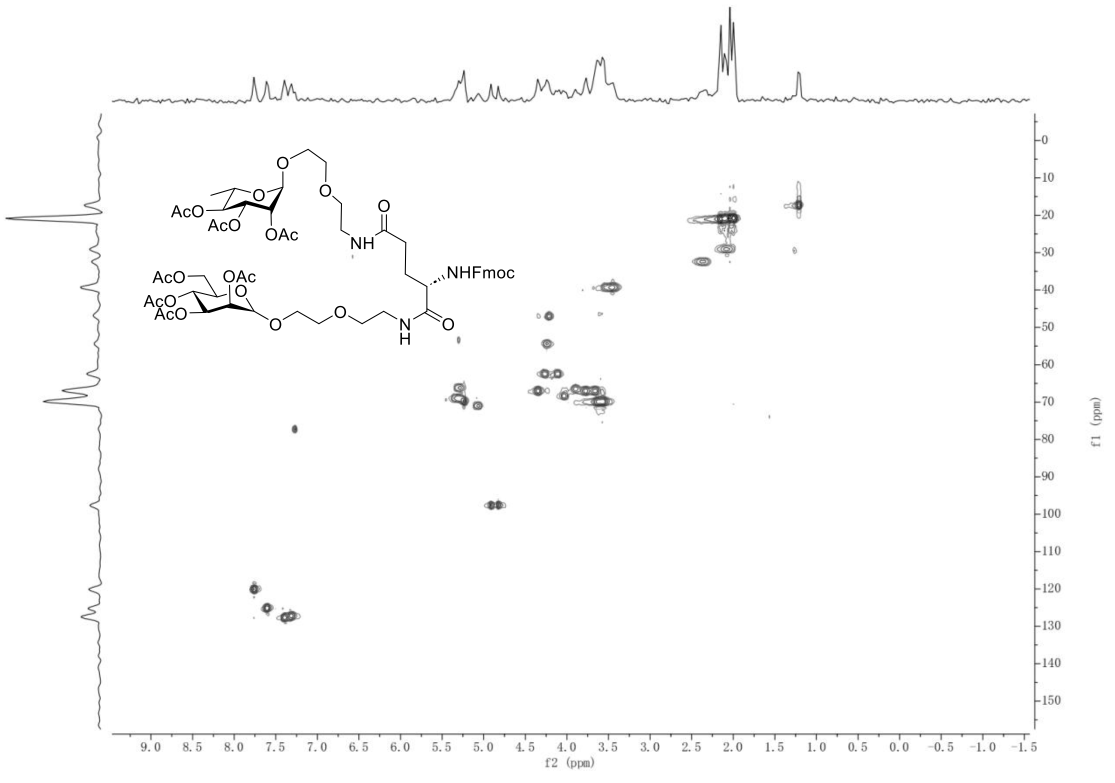

Figure S27. HSQC NMR (400/101 MHz, $CDCl_3$) spectrum of compound **16**.

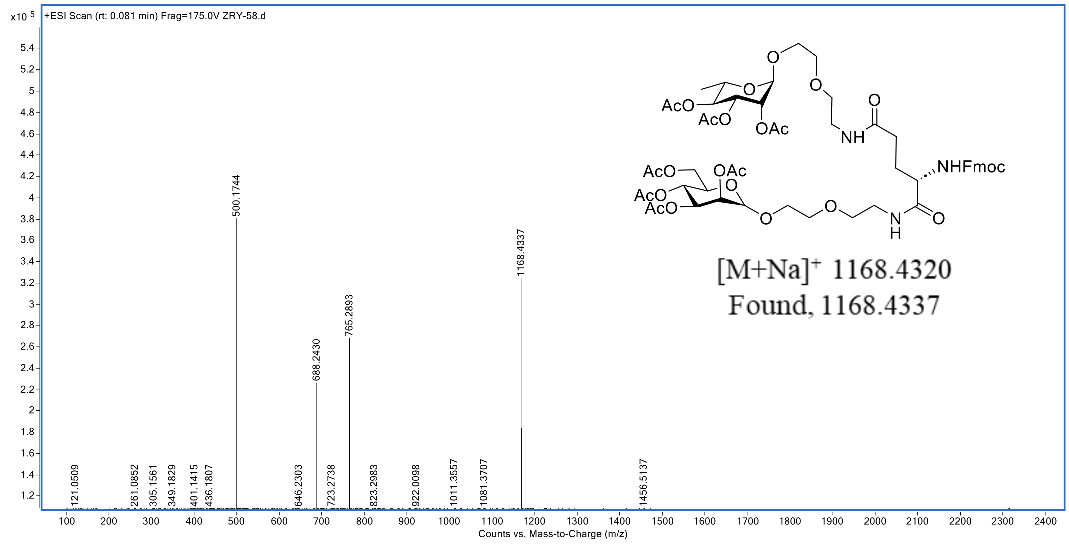

Figure S28. HRMS (ESI) spectrum of compound **16**.

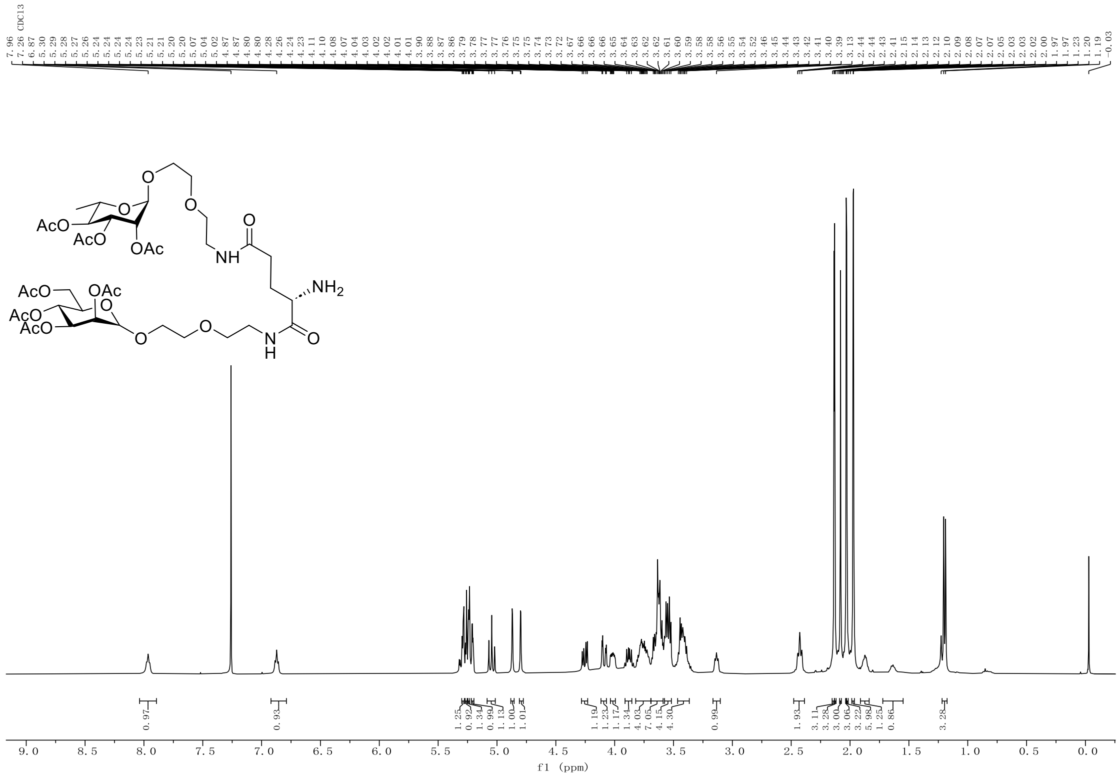

Figure S29. ^{1}H NMR (400 MHz, $CDCl_3$) spectrum of compound **17**.

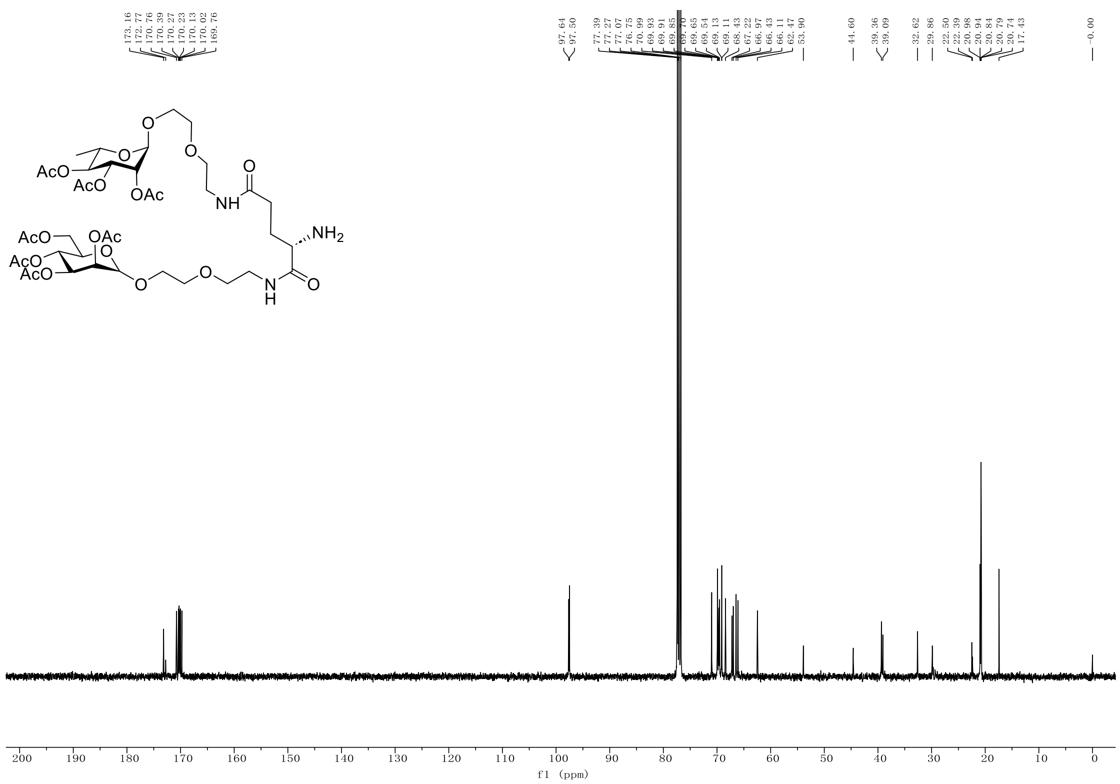

Figure S30. $^{13}C\{^{1}H\}$ NMR (101 MHz, $CDCl_3$) spectrum of compound **17**.

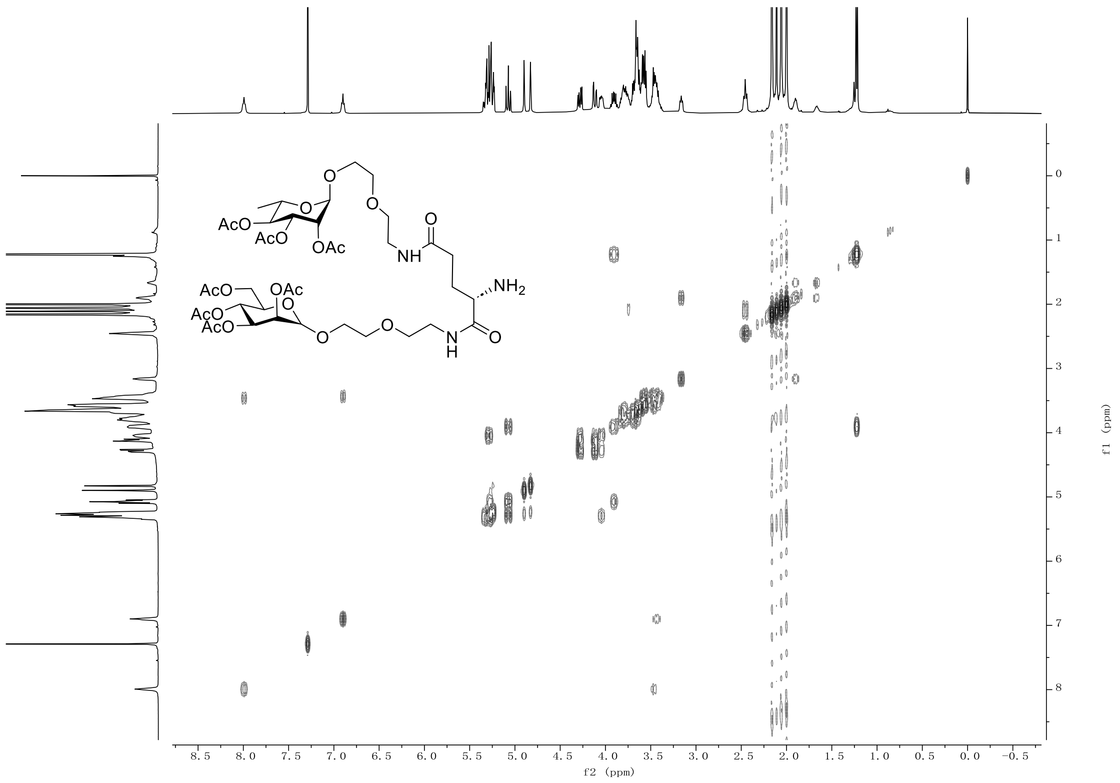

Figure S31. ^{1}H-^{1}H COSY NMR (400 MHz, $CDCl_3$) spectrum of compound **17**.

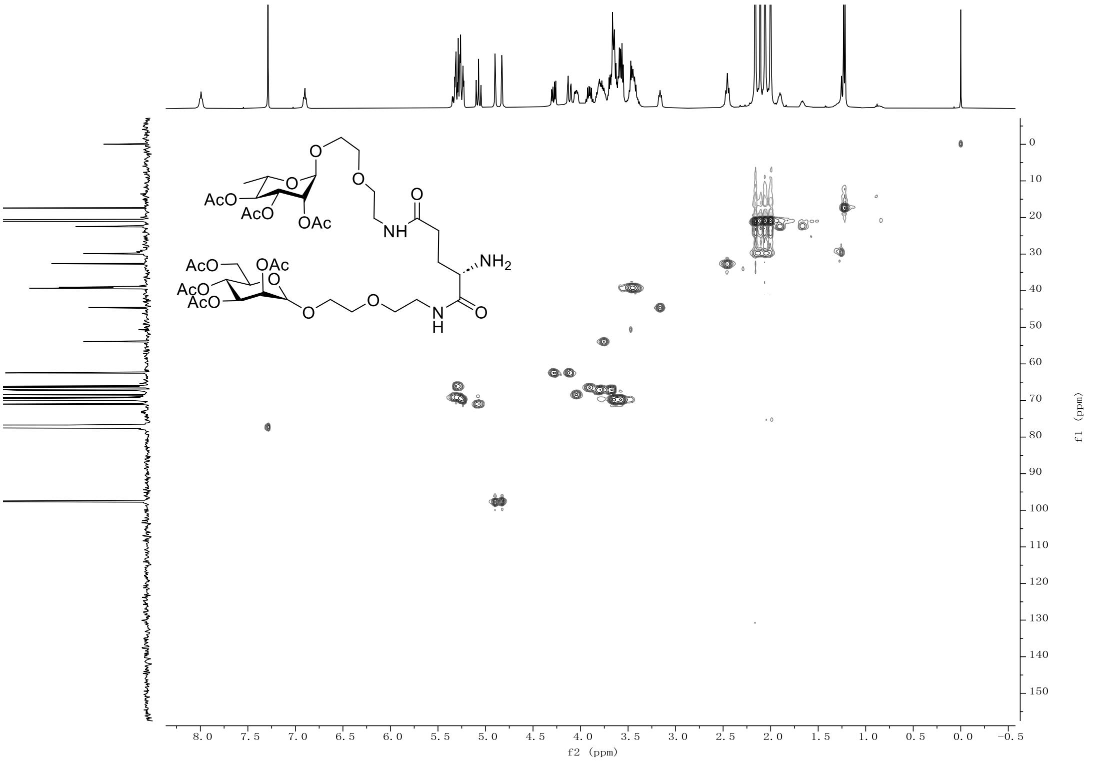

Figure S32. HSQC NMR (400/101 MHz, $CDCl_3$) spectrum of compound **17**.

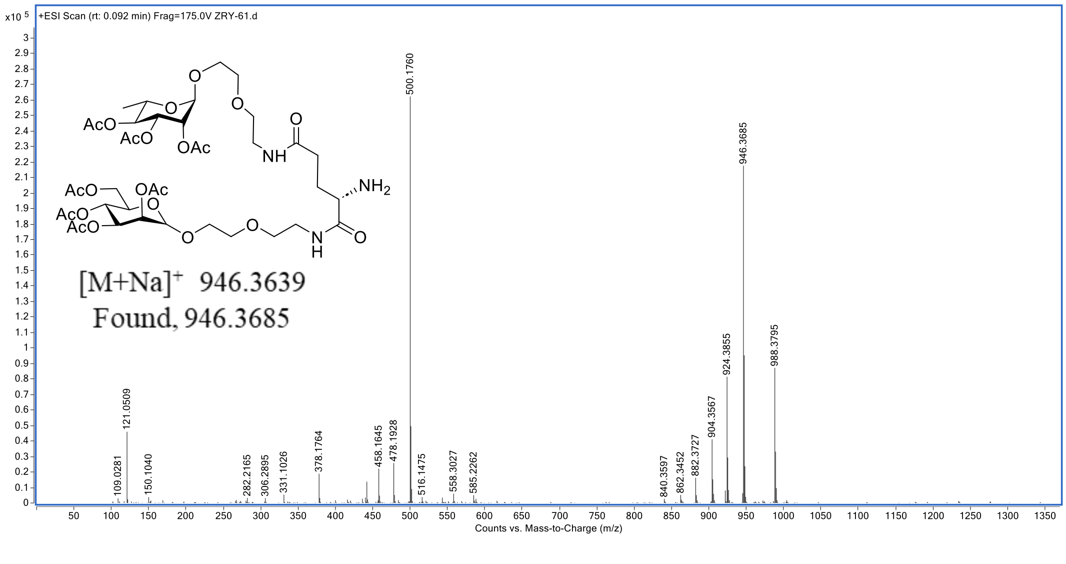

Figure S33. HRMS (ESI) spectrum of compound **17**.

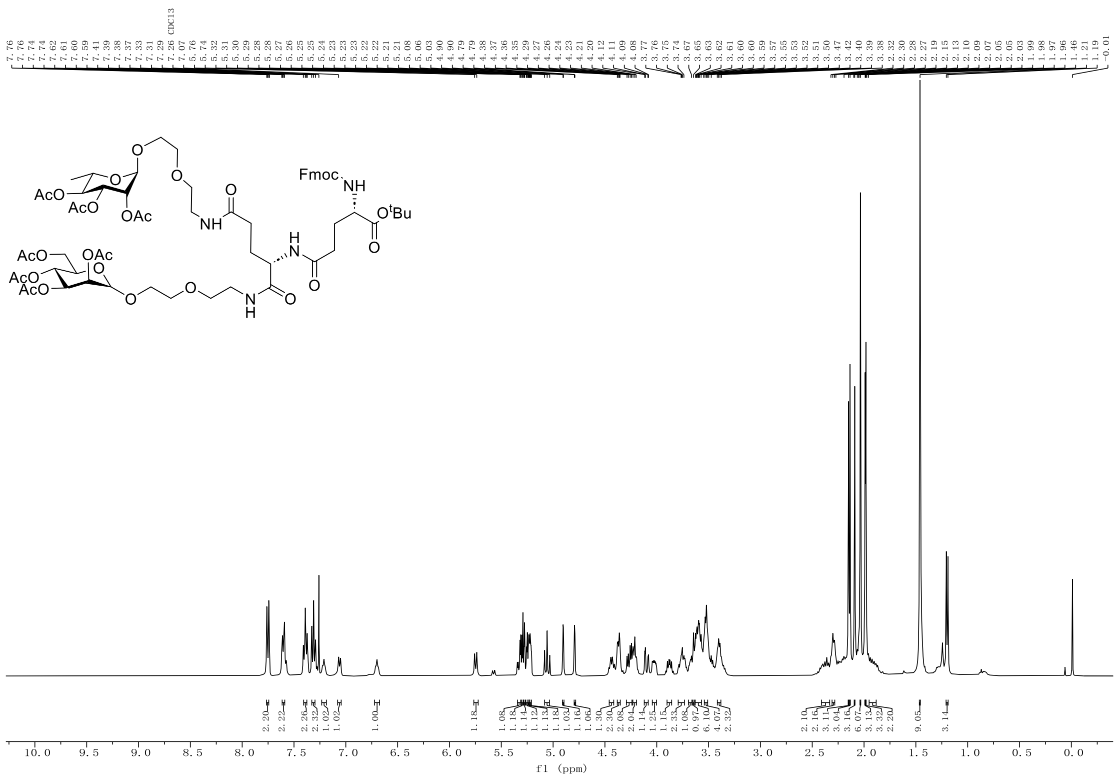

Figure S34. ^{1}H NMR (400 MHz, CDCl$_3$) spectrum of compound **18**.

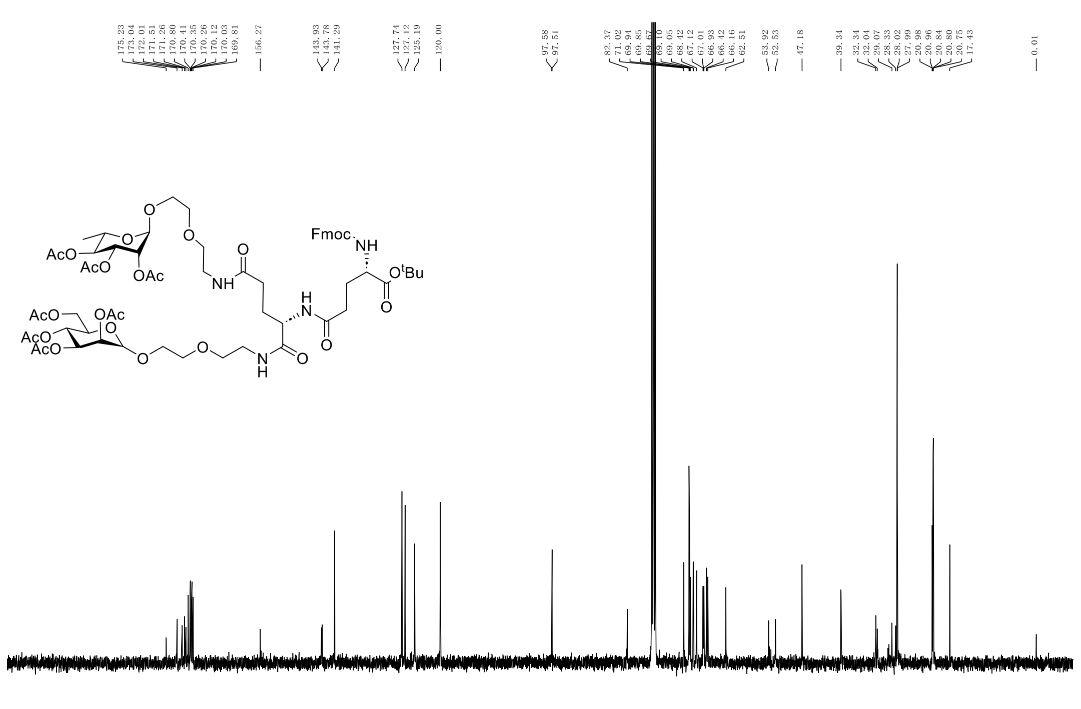

Figure S35. ^{13}C{^{1}H} NMR (100 MHz, CDCl$_3$) spectrum of compound **18**.

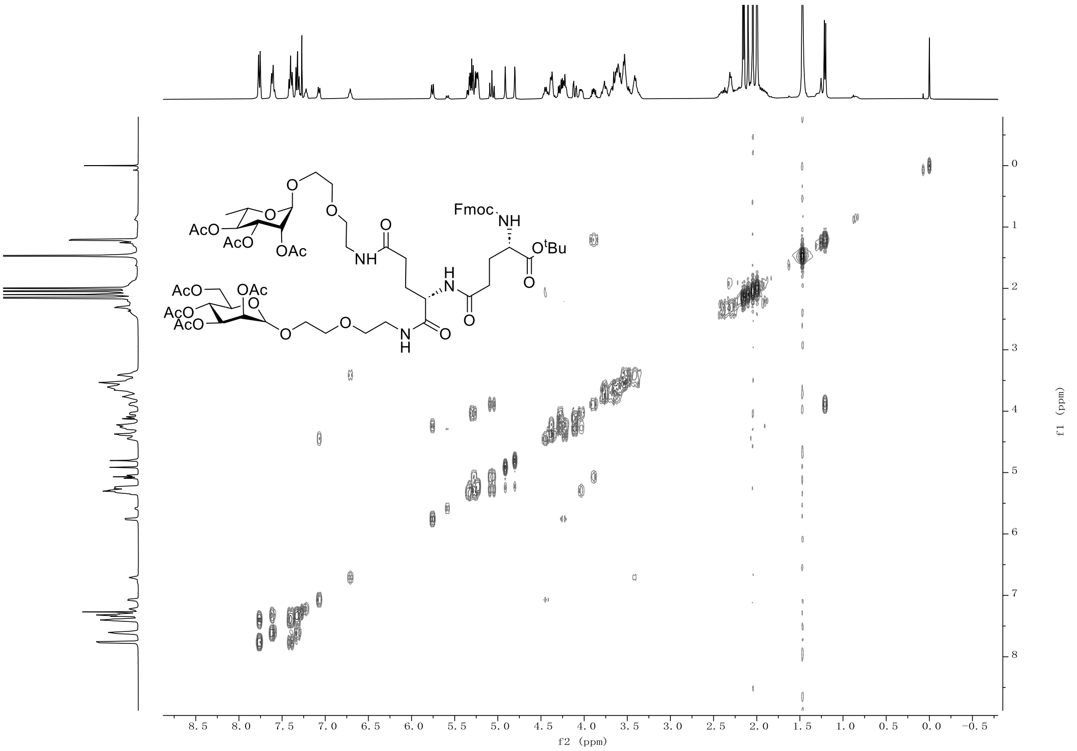

Figure S36. ^{1}H-^{1}H COSY NMR (400 MHz, CDCl$_3$) spectrum of compound **18**.

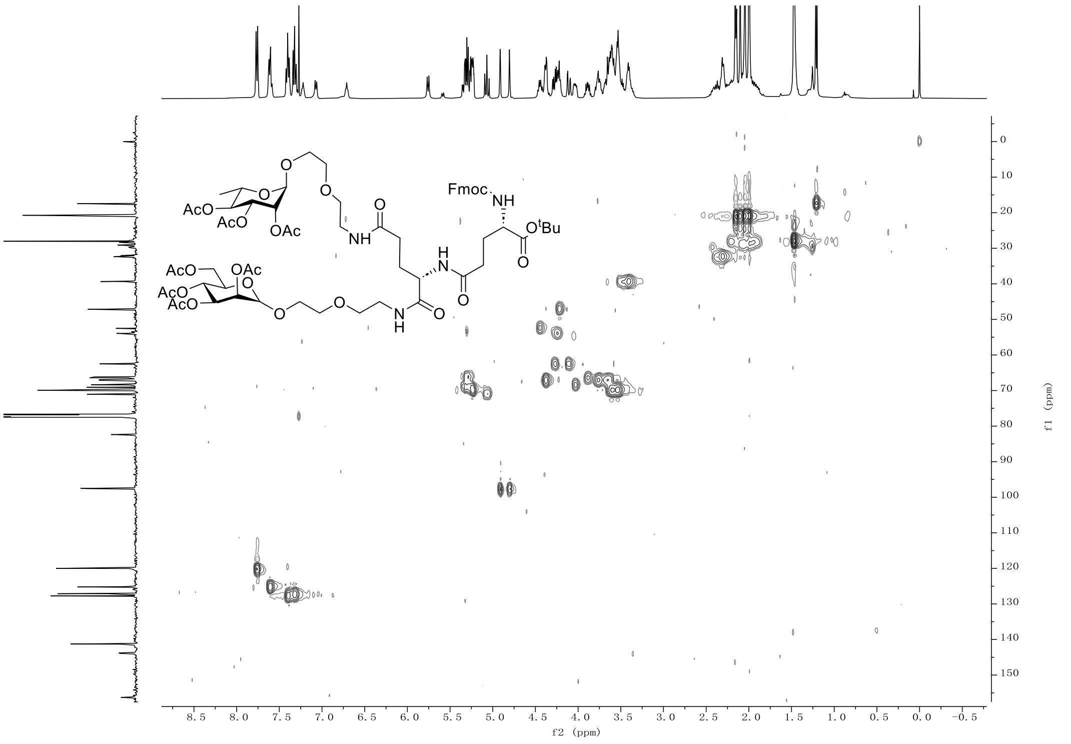

Figure S37. HSQC NMR (400/101 MHz, CDCl$_3$) spectrum of compound **18**.

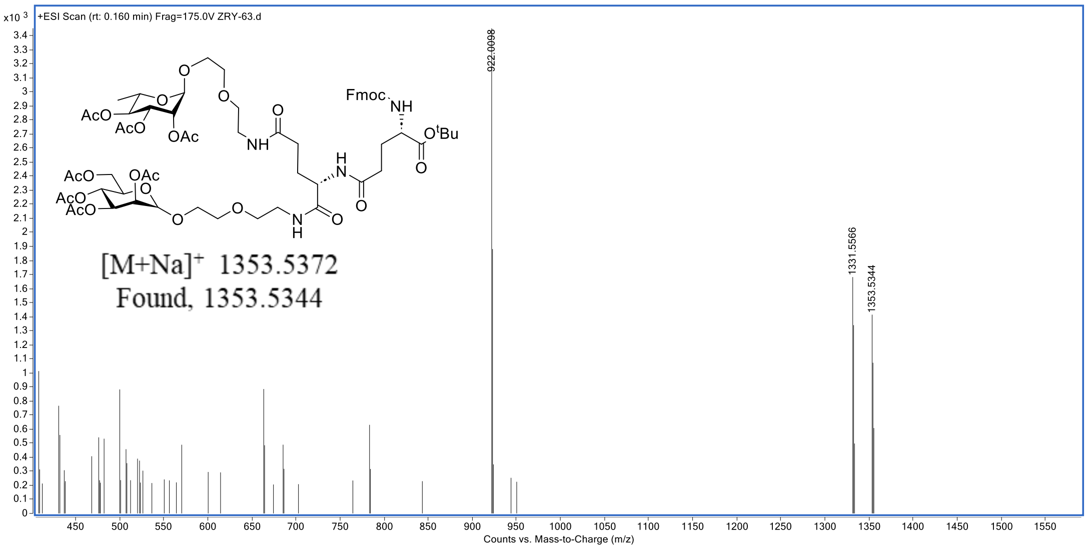

Figure S38. HRMS (ESI) spectrum of compound **18**.

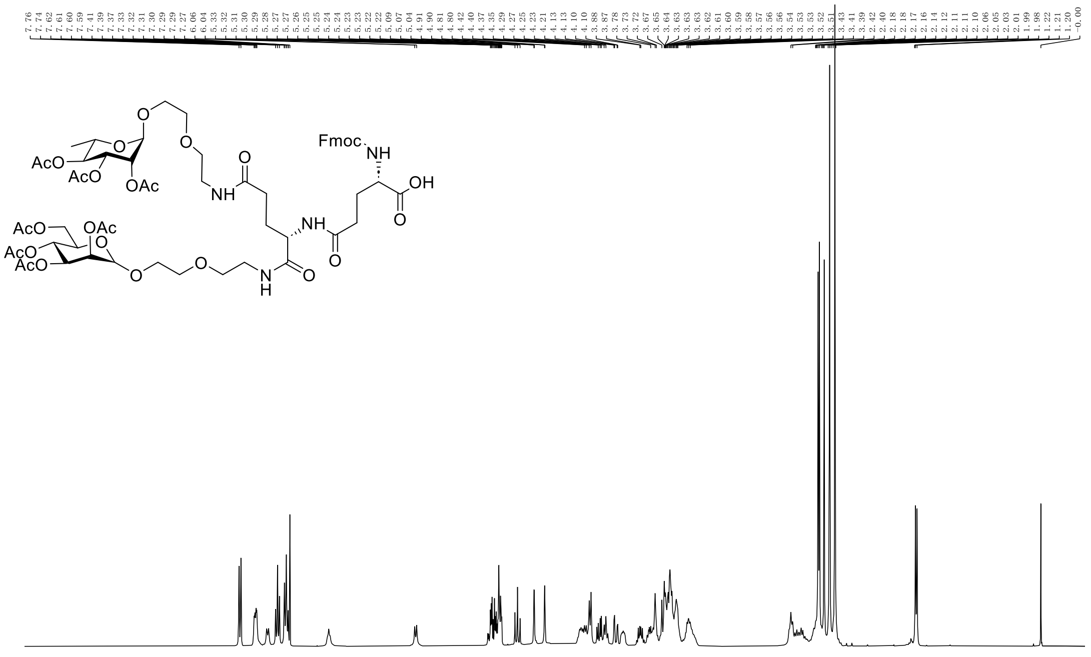

Figure S39. ^{1}H NMR (400 MHz, $CDCl_3$) spectrum of compound **19**.

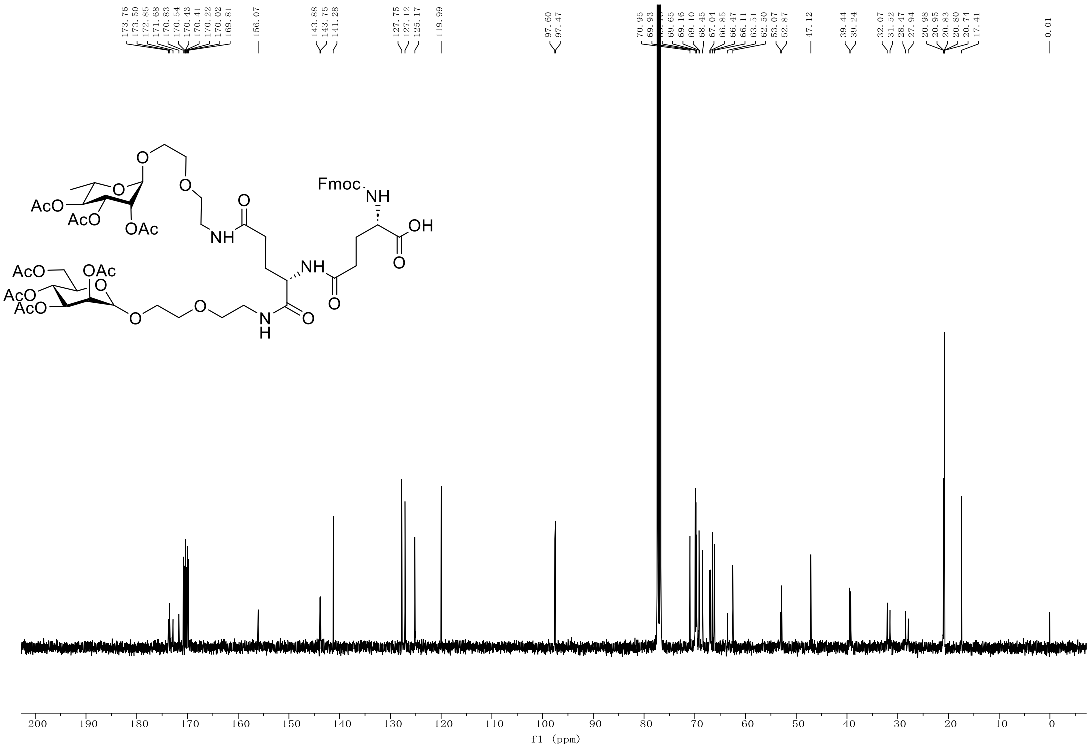

Figure S40. $^{13}C\{^{1}H\}$ NMR (101 MHz, $CDCl_3$) spectrum of compound **19**.

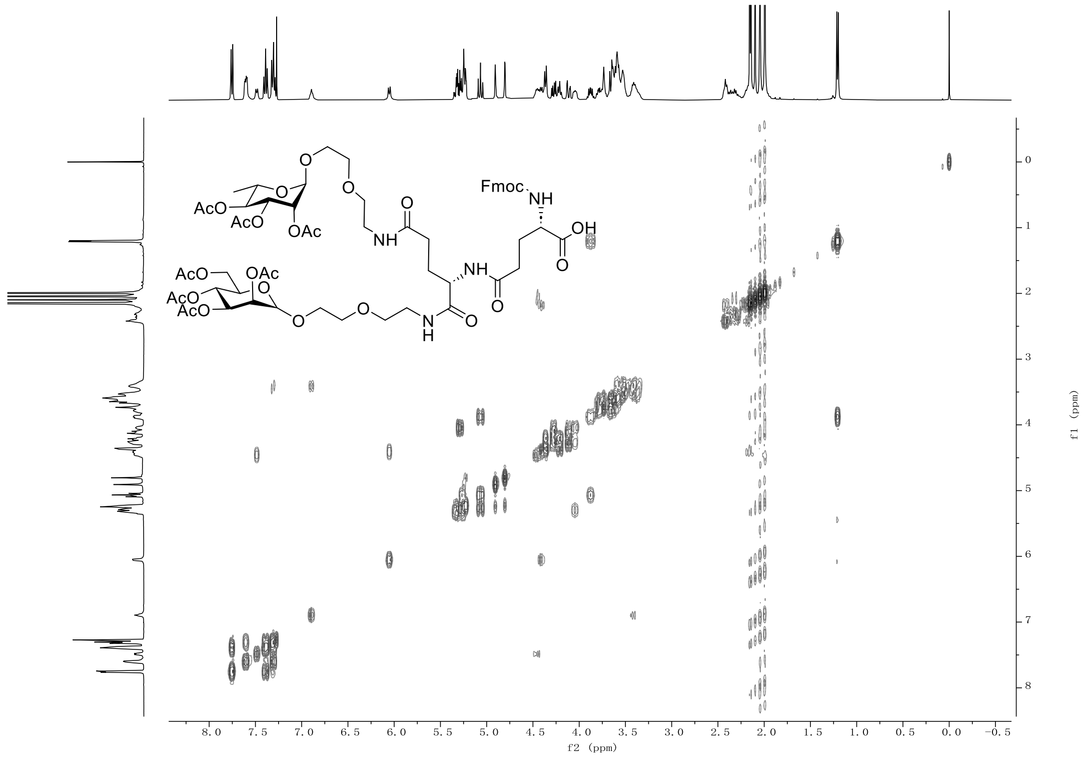

Figure S41. ^{1}H-^{1}H COSY NMR (400 MHz, $CDCl_3$) spectrum of compound **19**.

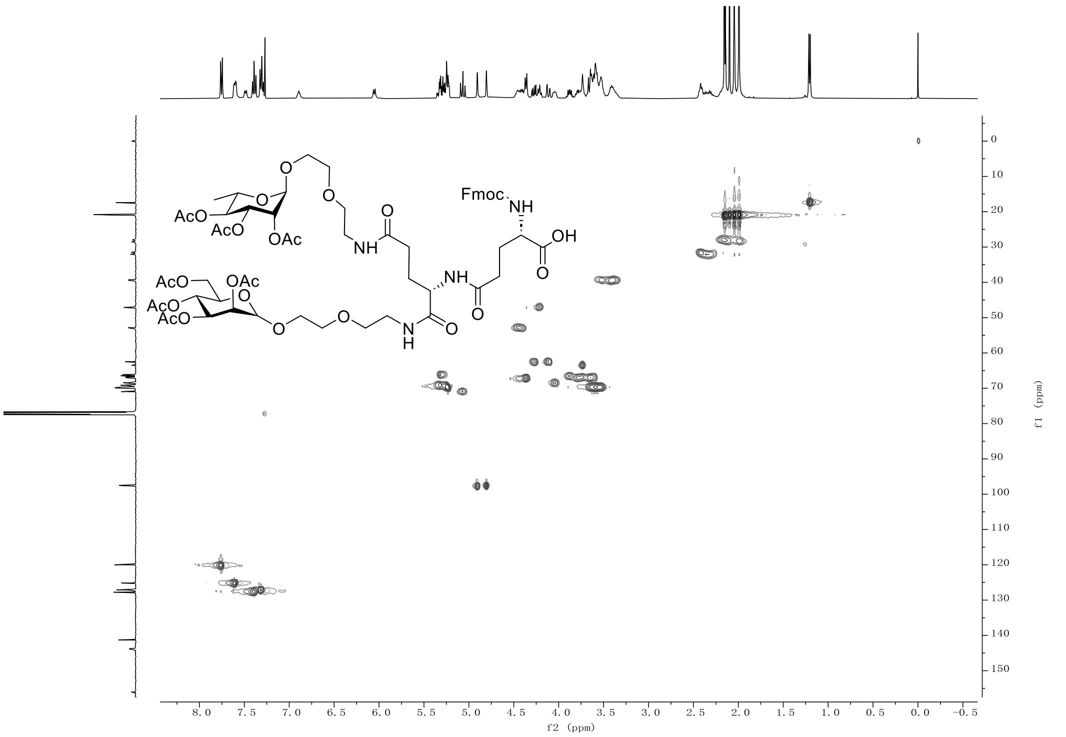

Figure S42. HSQC NMR (400/101 MHz, $CDCl_3$) spectrum of compound **19**.

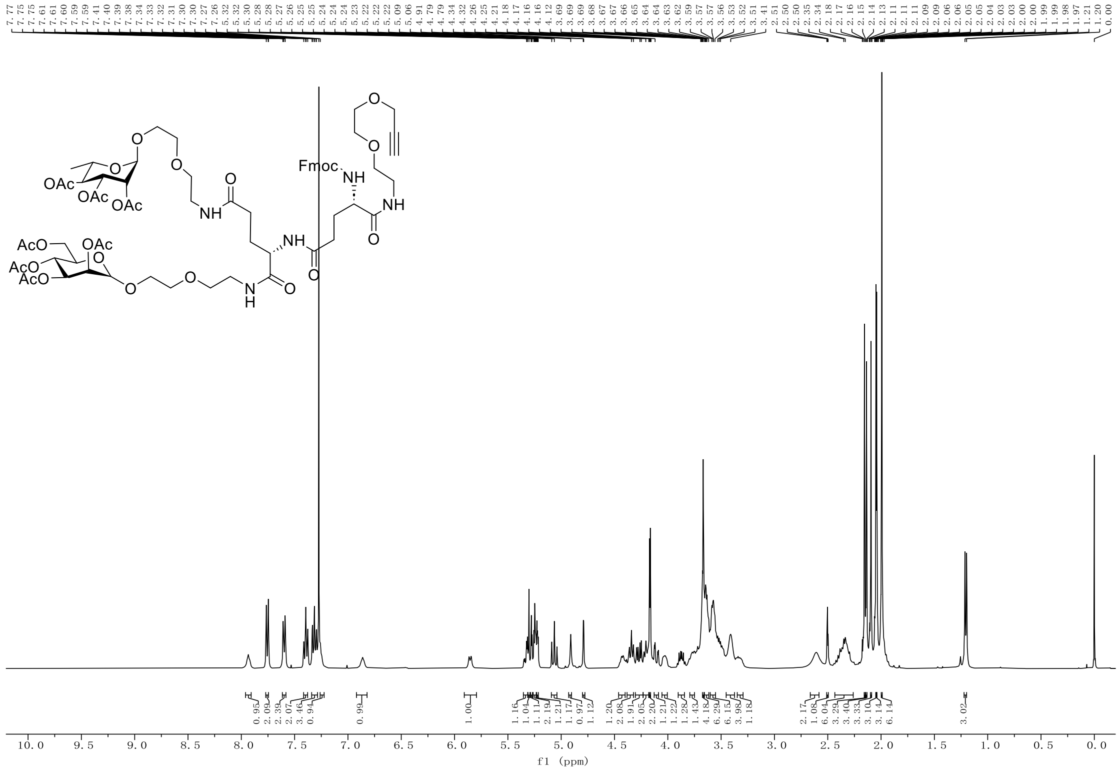

Figure S43. ^1H NMR (400 MHz, $CDCl_3$) spectrum of compound **20**.

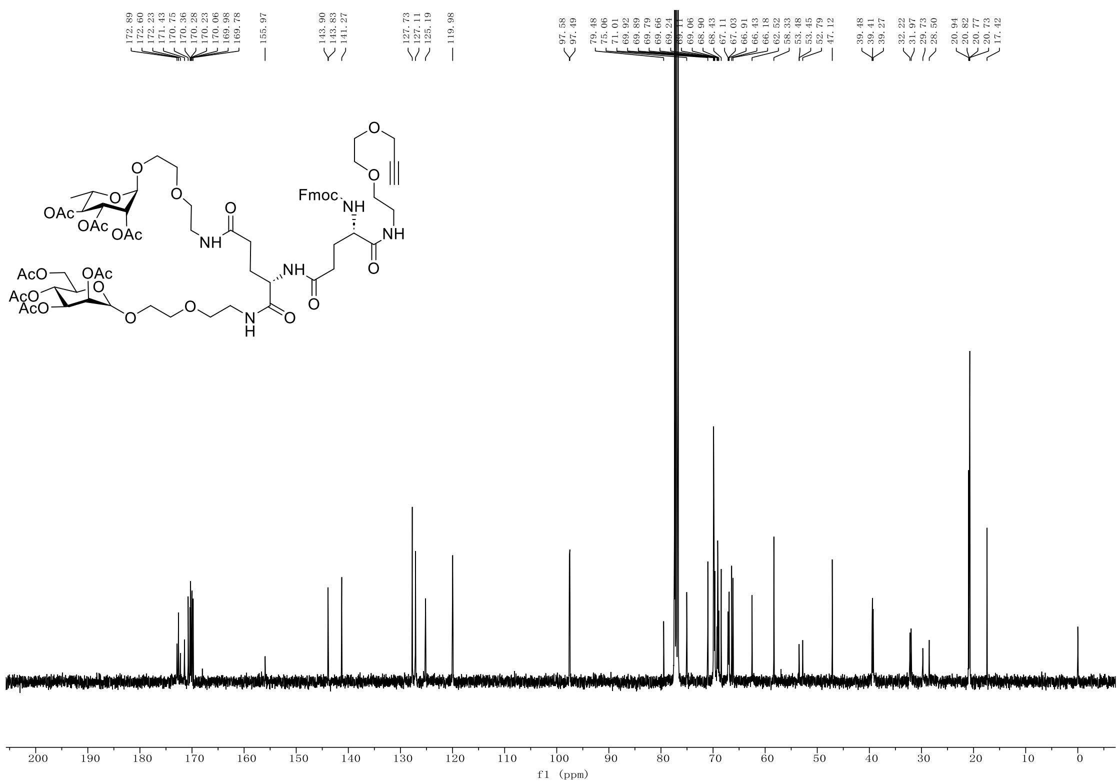

Figure S44. $^{13}C\{^{1}H\}$ NMR (101 MHz, $CDCl_3$) spectrum of compound **20**.

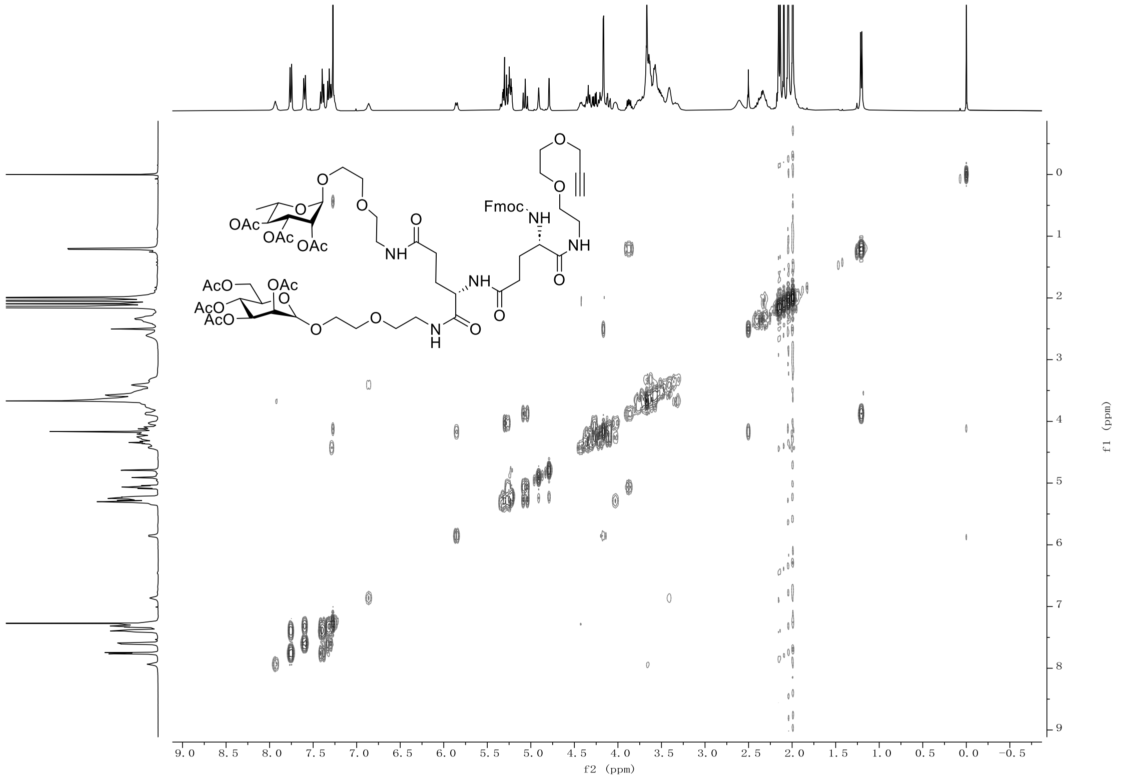

Figure S45. ^{1}H-^{1}H COSY NMR (400 MHz, $CDCl_3$) spectrum of compound **20**.

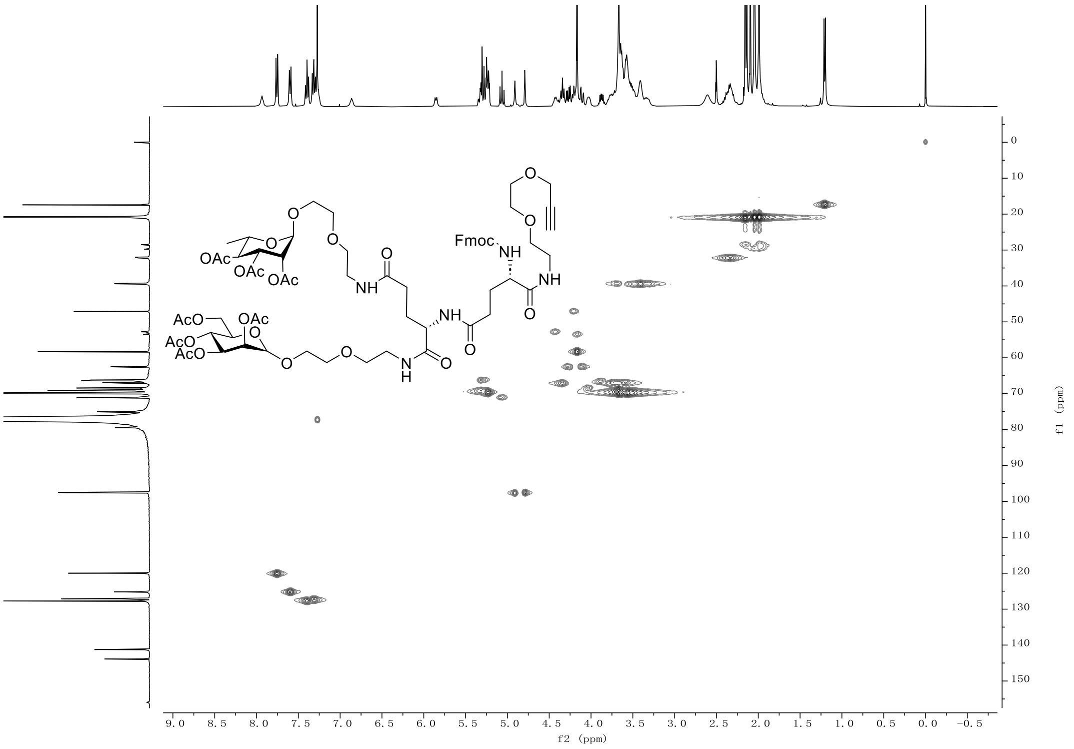

Figure S46. HSQC NMR (400/101 MHz, $CDCl_3$) spectrum of compound **20**.

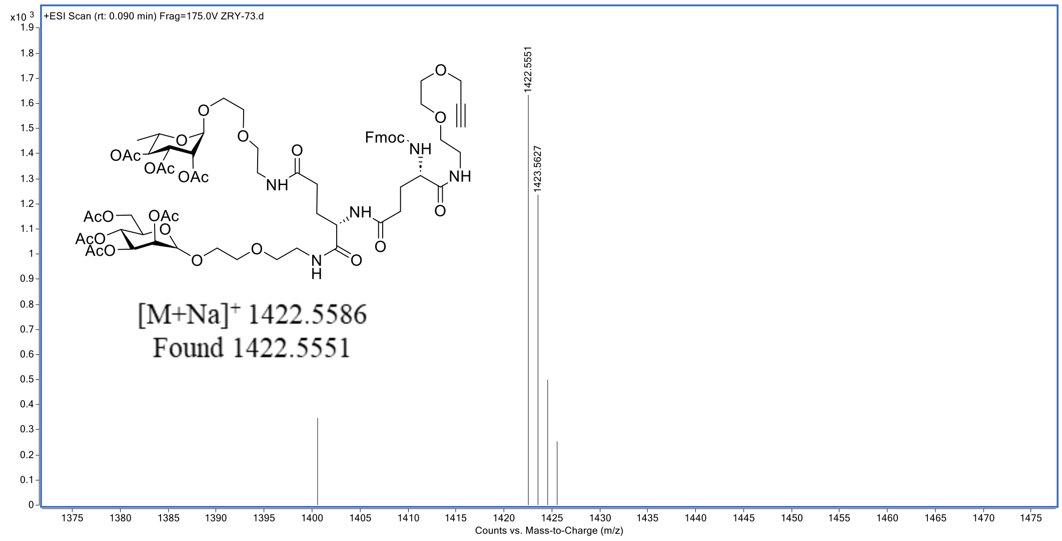

Figure S47. HRMS (ESI) spectrum of compound **20**.

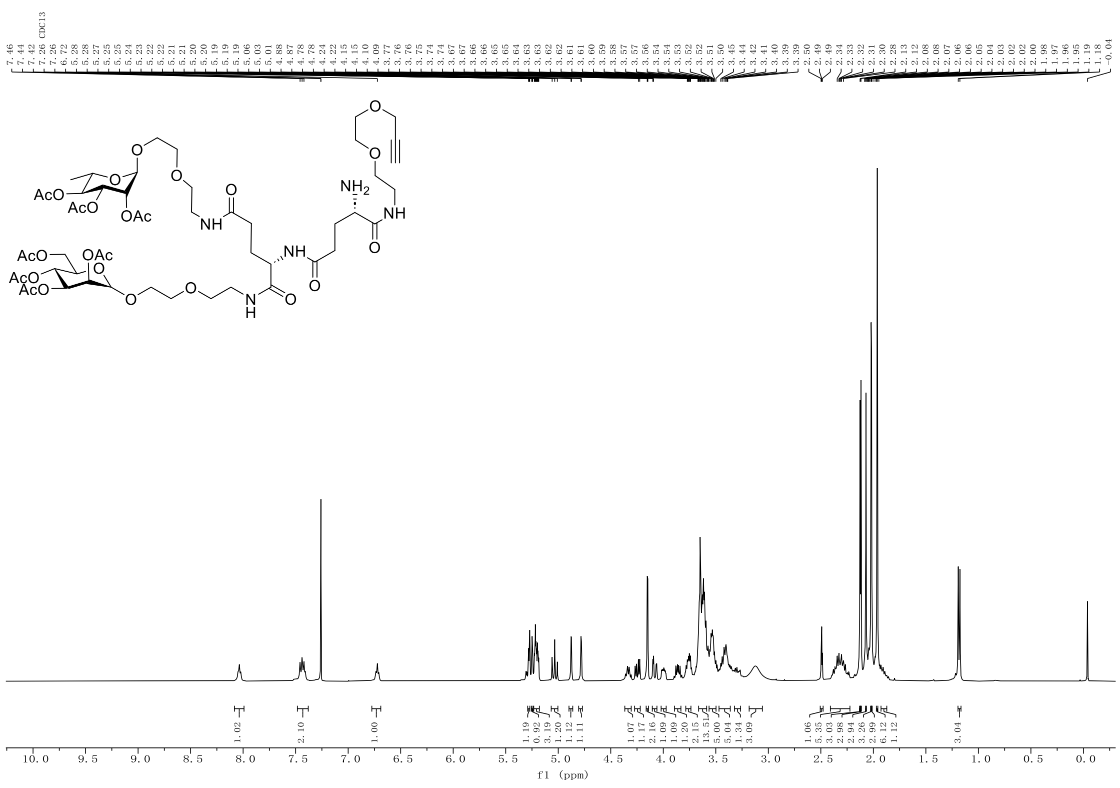

Figure S48. ^{1}H NMR (400 MHz, $CDCl_3$) spectrum of compound **3**.

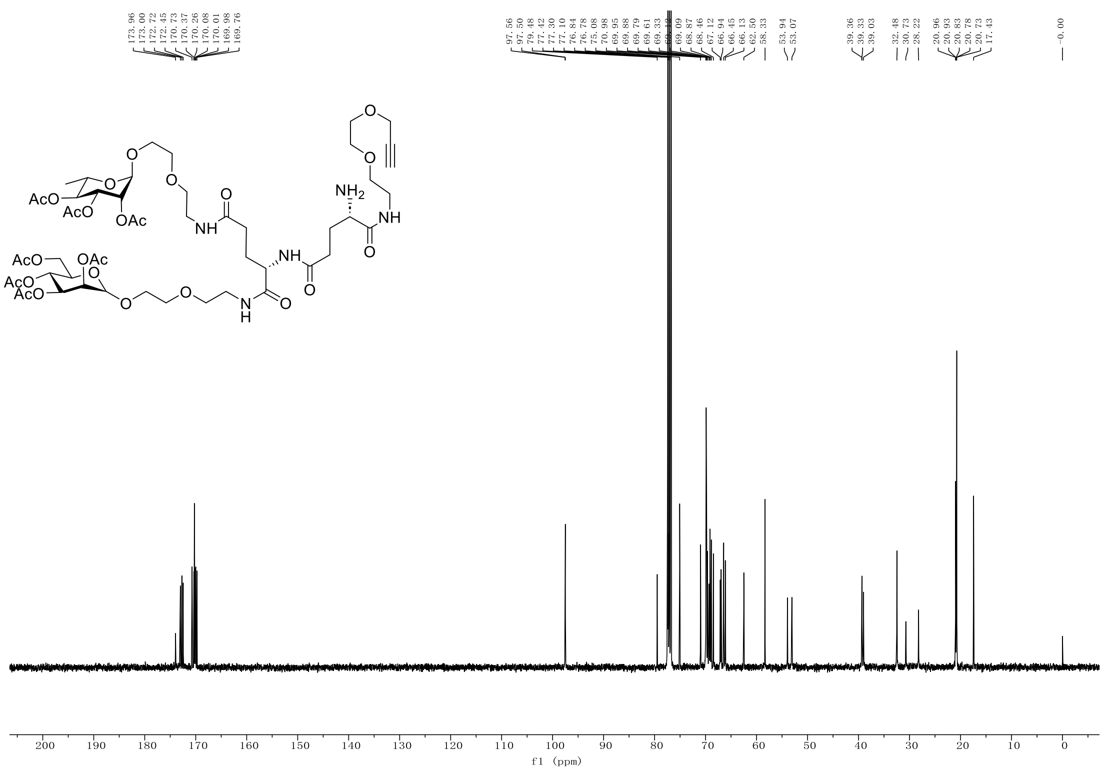

Figure S49. $^{13}C\{^{1}H\}$ NMR (101 MHz, $CDCl_3$) spectrum of compound **3**.

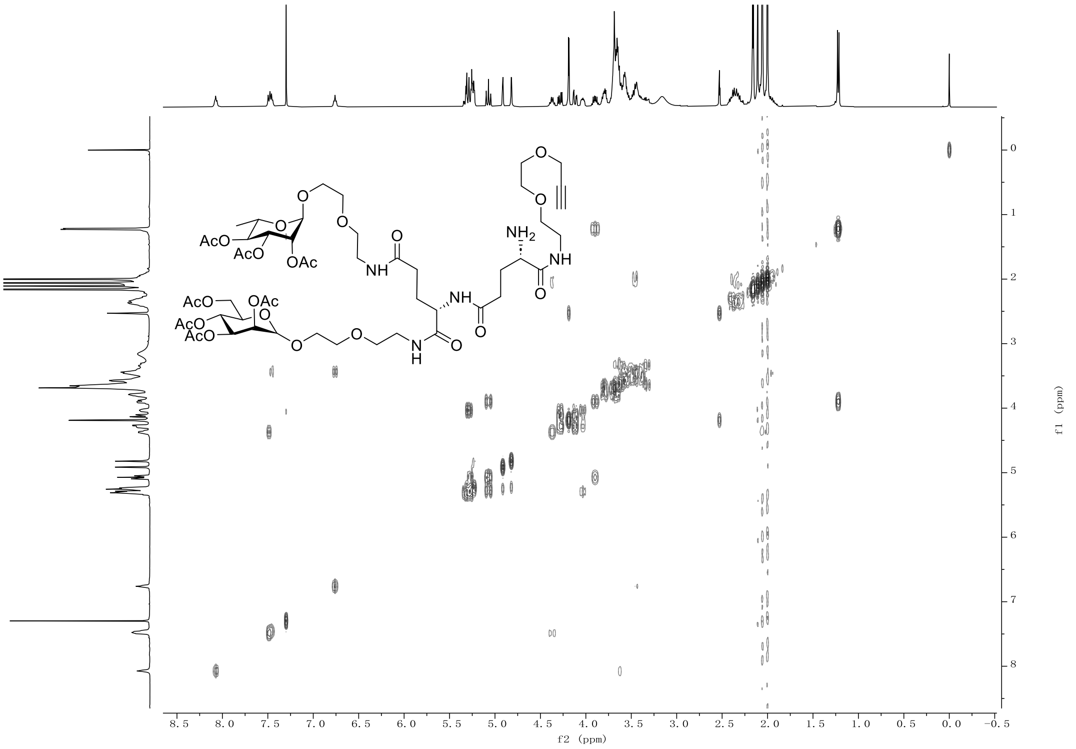

Figure S50. ^1H-^1H COSY NMR (400 MHz, $CDCl_3$) spectrum of compound **3**.

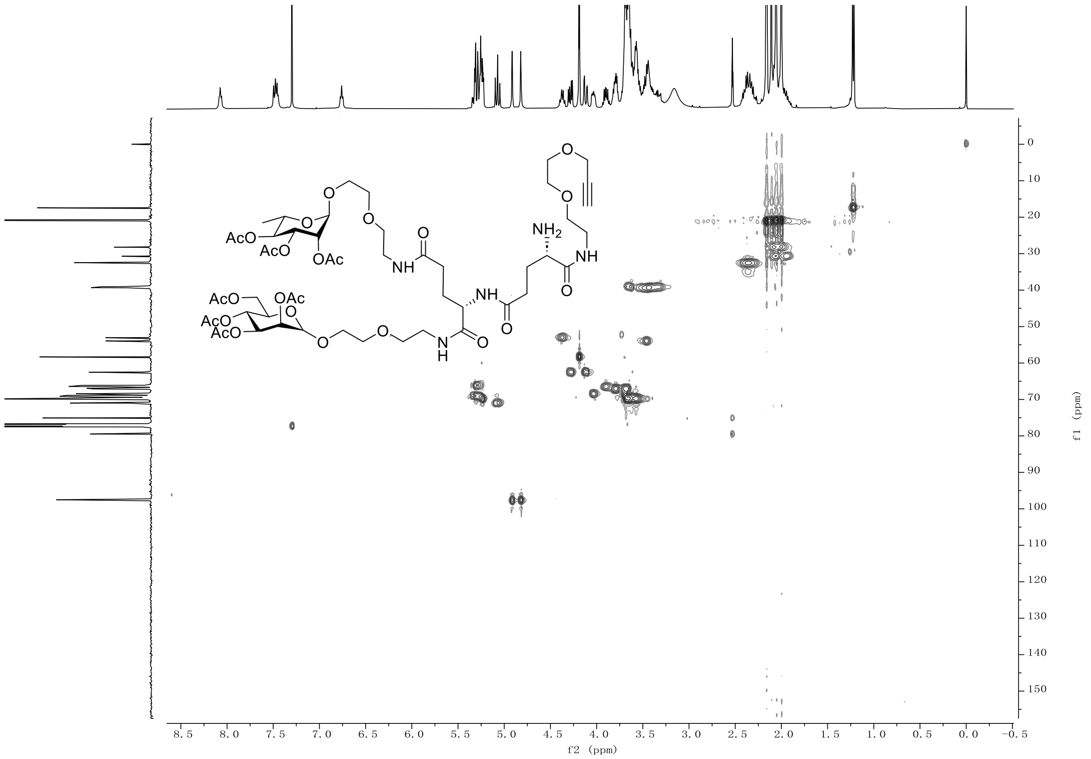

Figure S51. HSQC NMR (400/101 MHz, $CDCl_3$) spectrum of compound **3**.

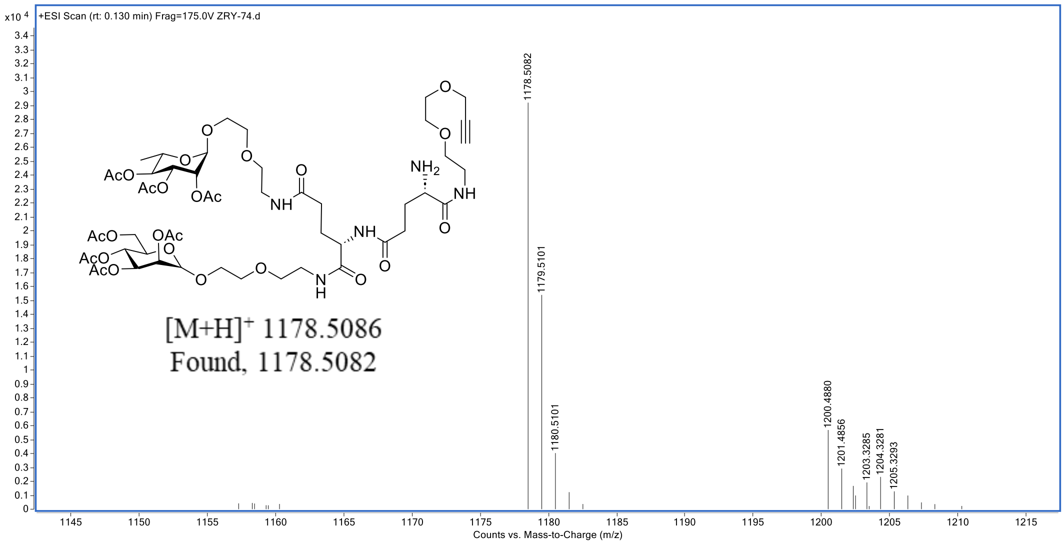

Figure S52. HRMS (ESI) spectrum of compound **3**.

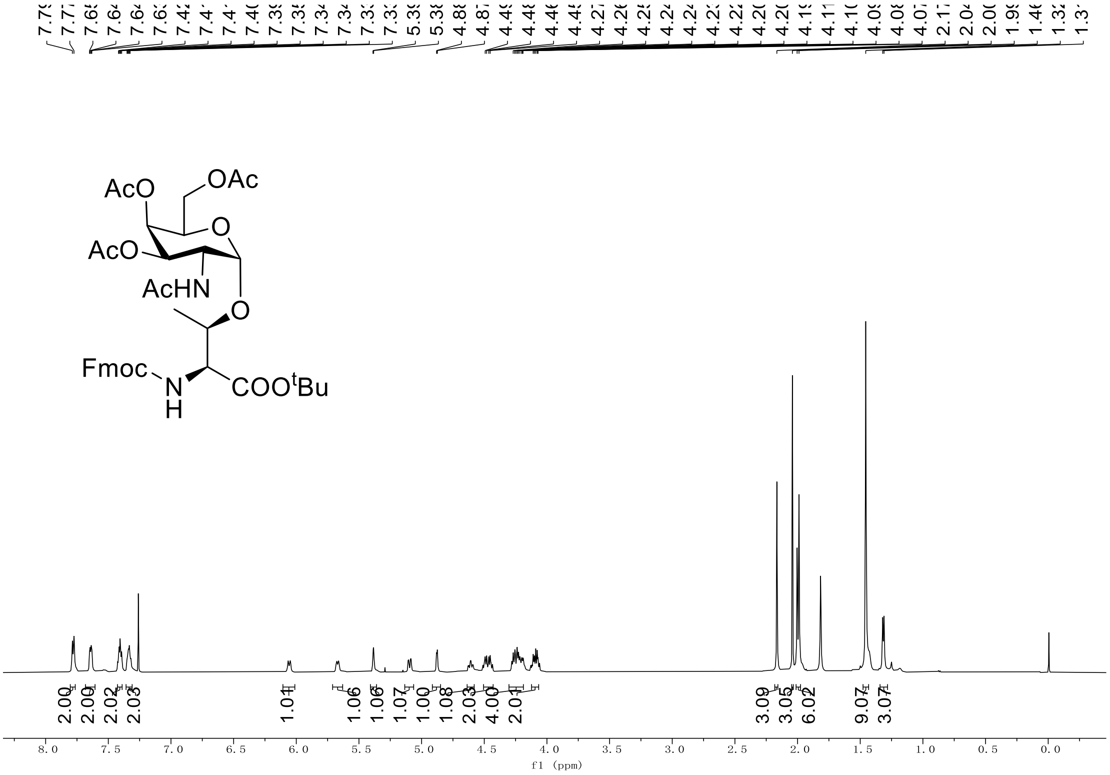

Figure S53. ^{1}H NMR (600 MHz, $CDCl_3$) spectrum of compound **S4**

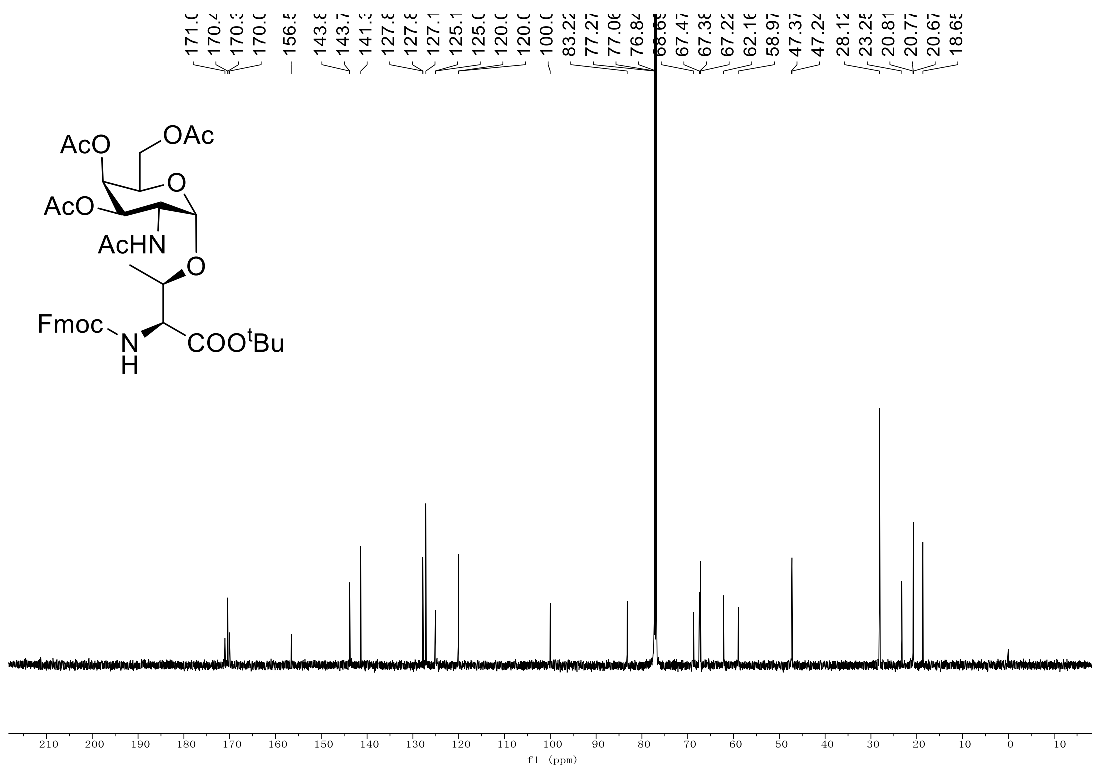

Figure S54. $^{13}C\{^{1}H\}$ NMR (151 MHz, $CDCl_3$) spectrum of compound **S4**

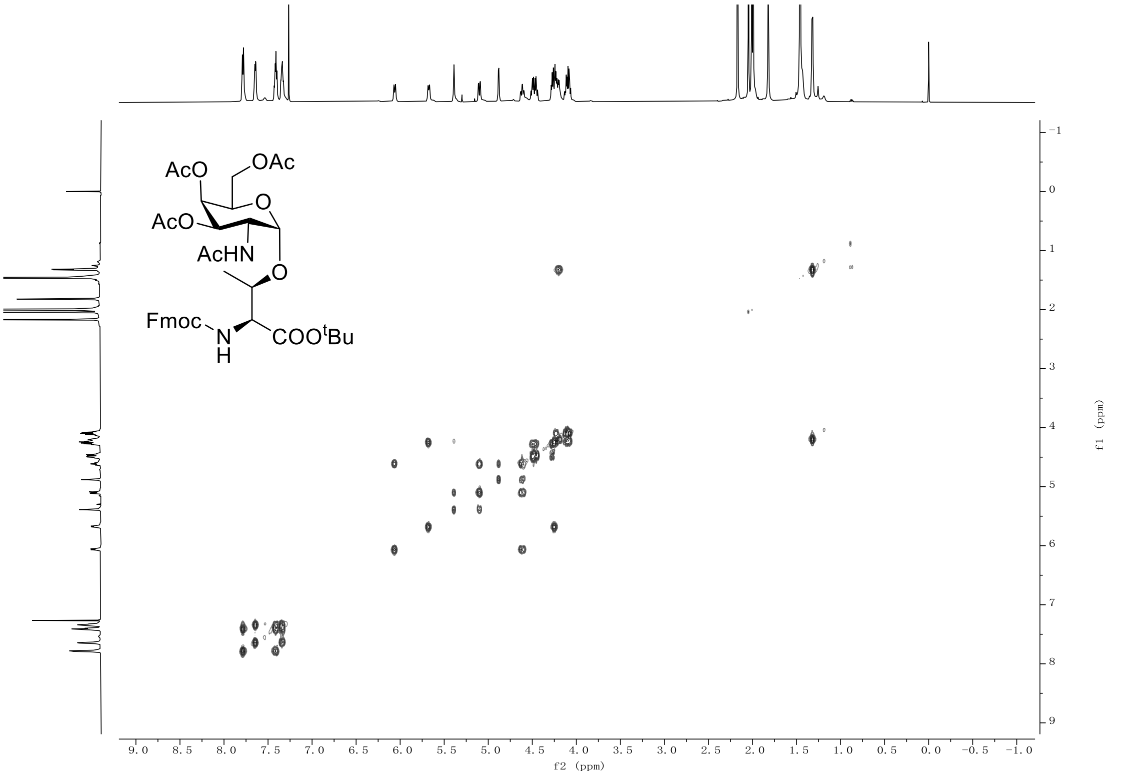

Figure S55. ^{1}H-^{1}H COSY NMR (600 MHz, $CDCl_3$) spectrum of compound **S4**.

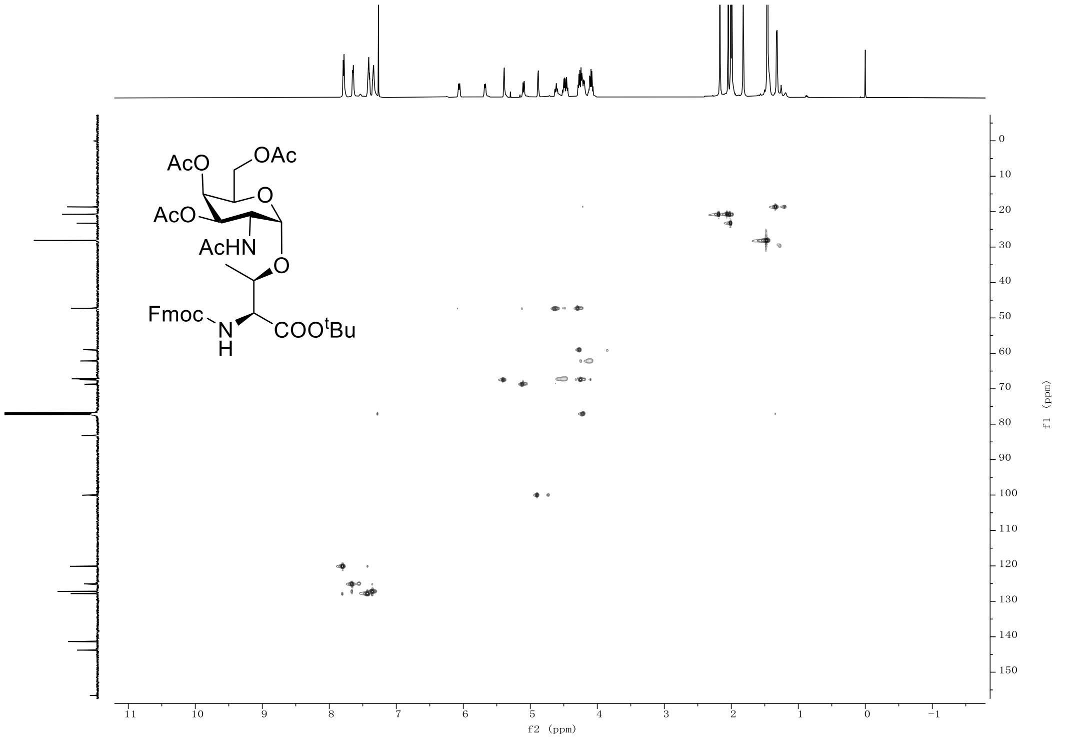

Figure S56. HSQC NMR (600/151 MHz, $CDCl_3$) spectrum of compound **S4**.

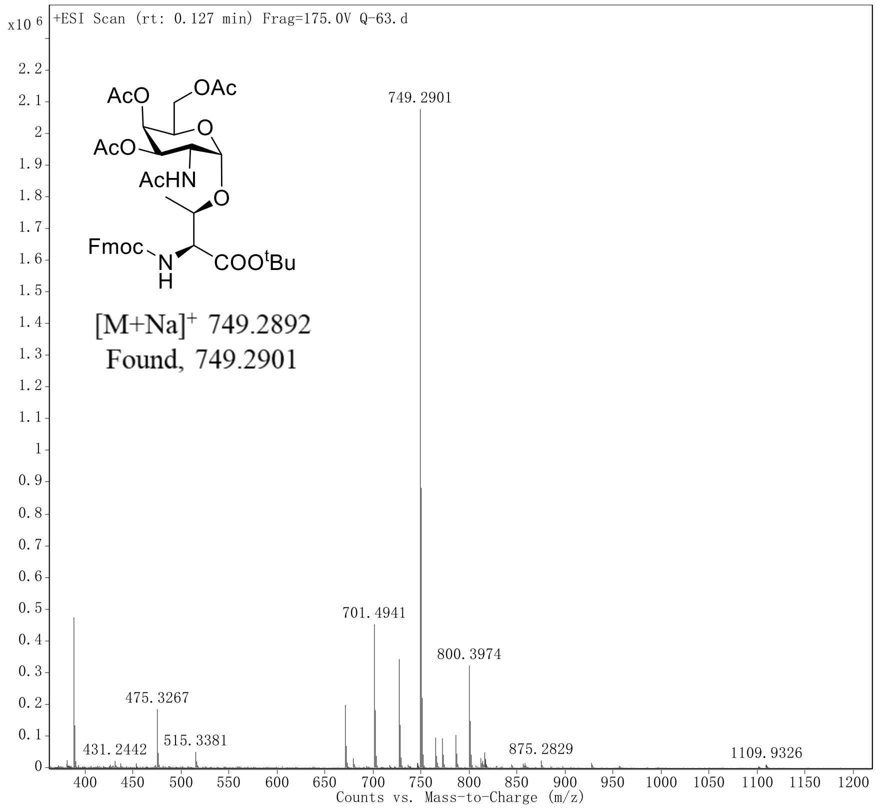

Figure S57. HRMS (ESI) spectrum of compound **S4**.

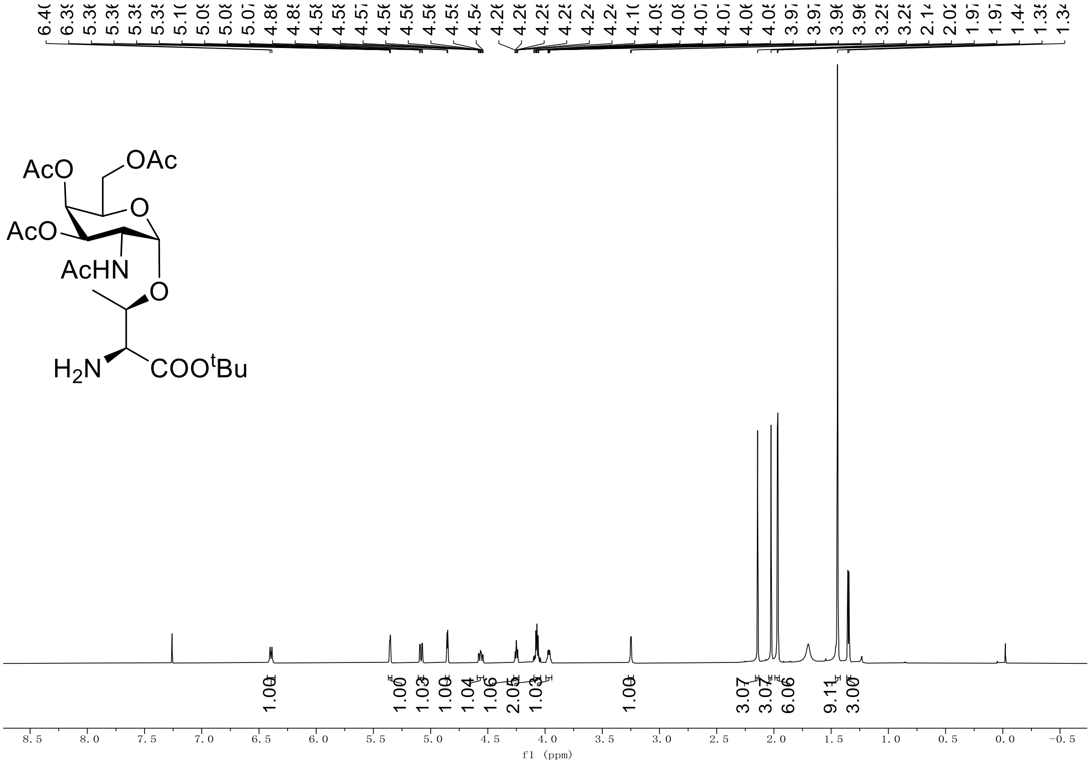

Figure S58. ^{1}H NMR (600 MHz, $CDCl_3$) spectrum of compound **S5**.

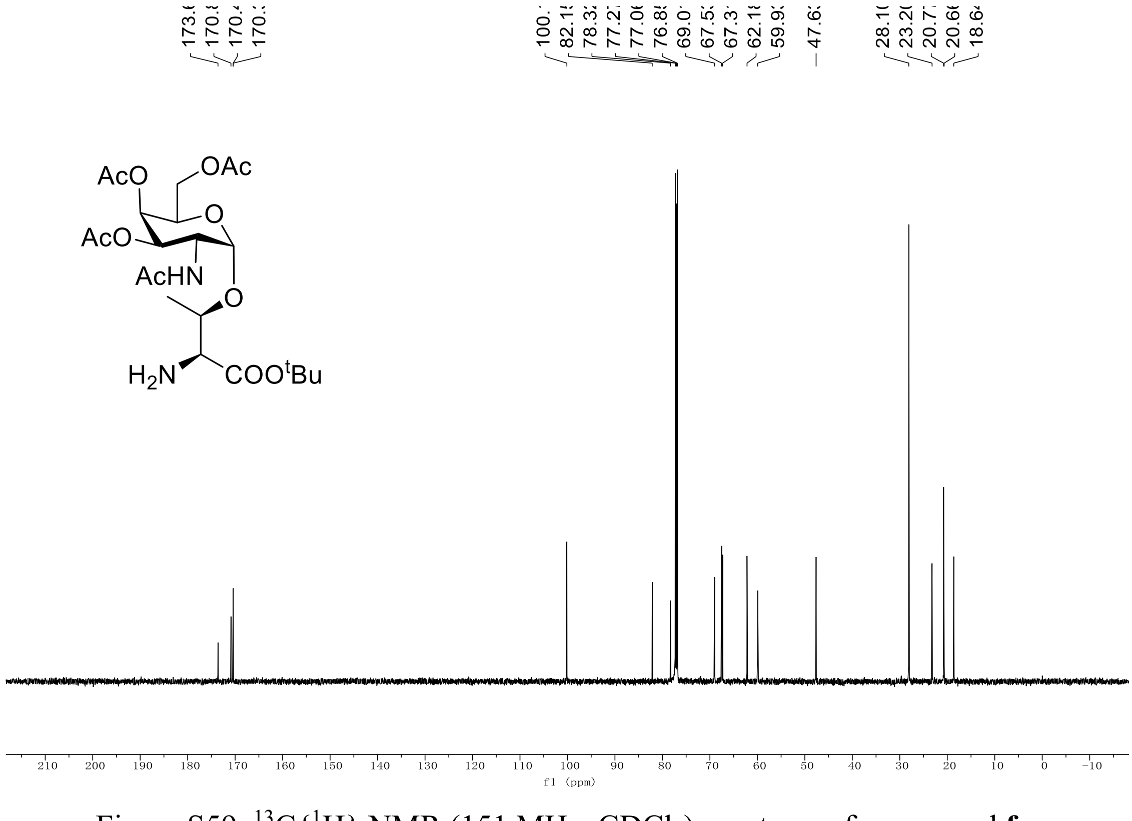

Figure S59. $^{13}C\{^{1}H\}$ NMR (151 MHz, $CDCl_3$) spectrum of compound **f**.

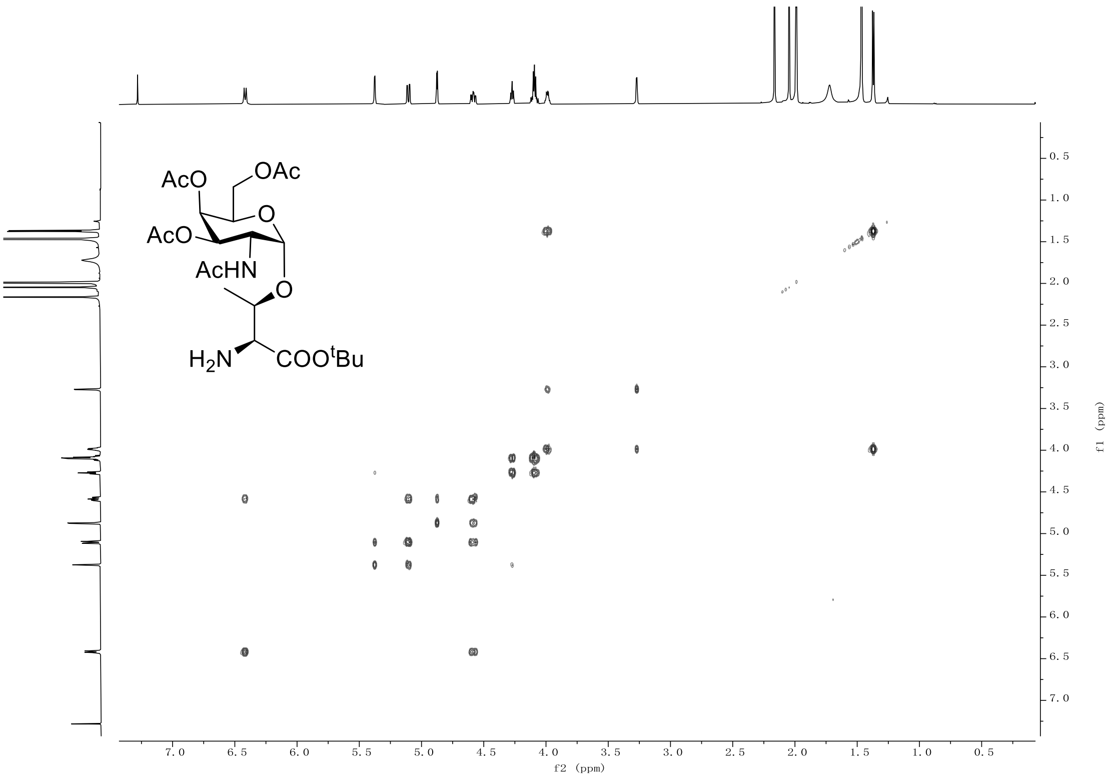

Figure S60. ^{1}H-^{1}H COSY NMR (600 MHz, $CDCl_3$) spectrum of compound **S5**.

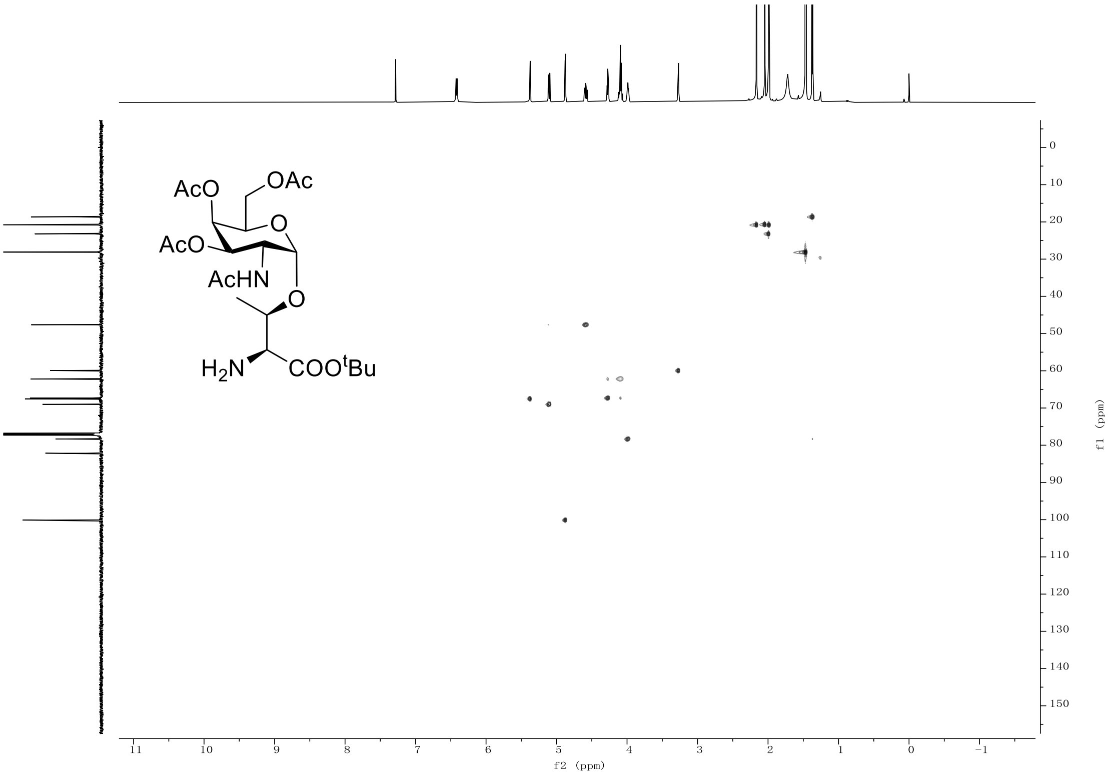

Figure S61. HSQC NMR (600/151 MHz, $CDCl_3$) spectrum of compound **S5**.

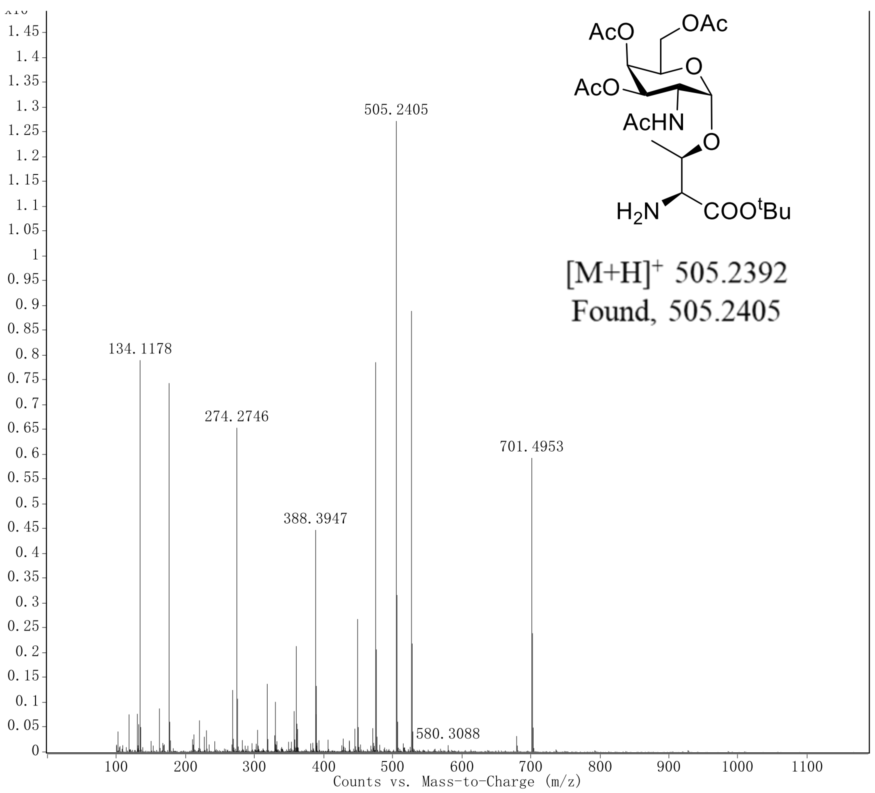

Figure S62. HRMS (ESI) spectrum of compound **S5**.

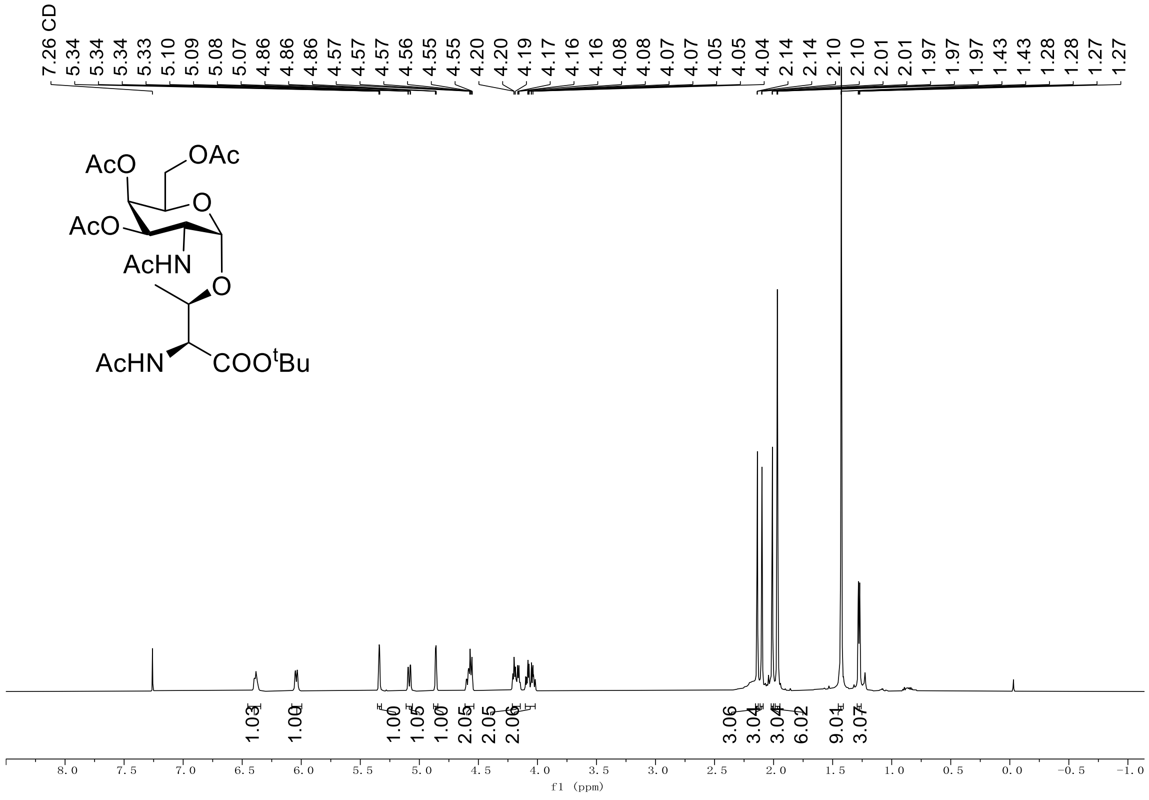

Figure S63. ^{1}H NMR (600 MHz, $CDCl_3$) spectrum of compound **S6**.

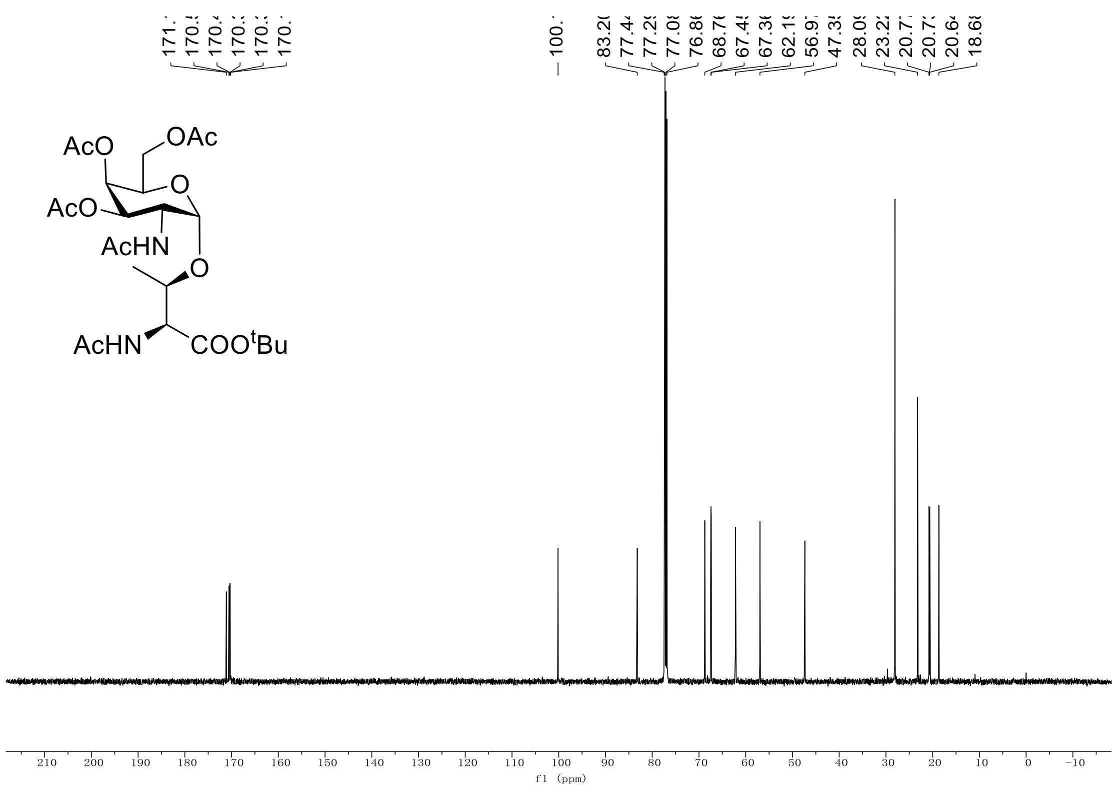

Figure S64. $^{13}C\{^{1}H\}$ NMR (151 MHz, $CDCl_3$) spectrum of compound **S6**.

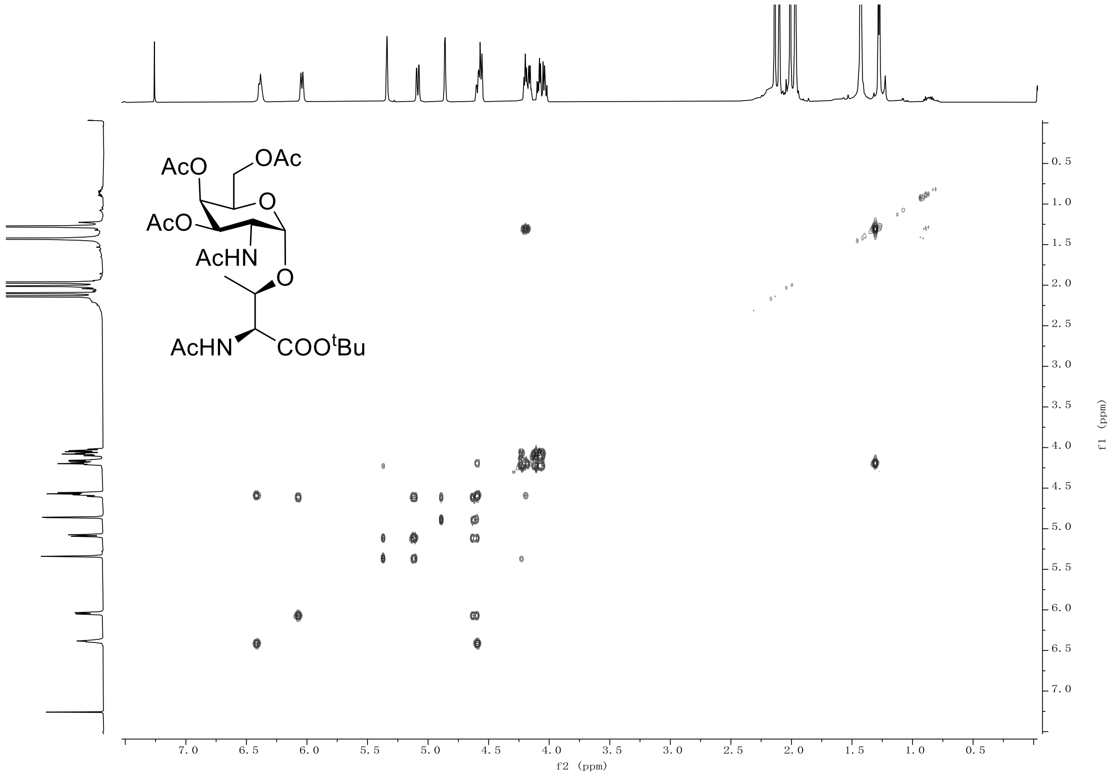

Figure S65. ^{1}H-^{1}H COSY NMR (600 MHz, $CDCl_3$) spectrum of compound **S6**.

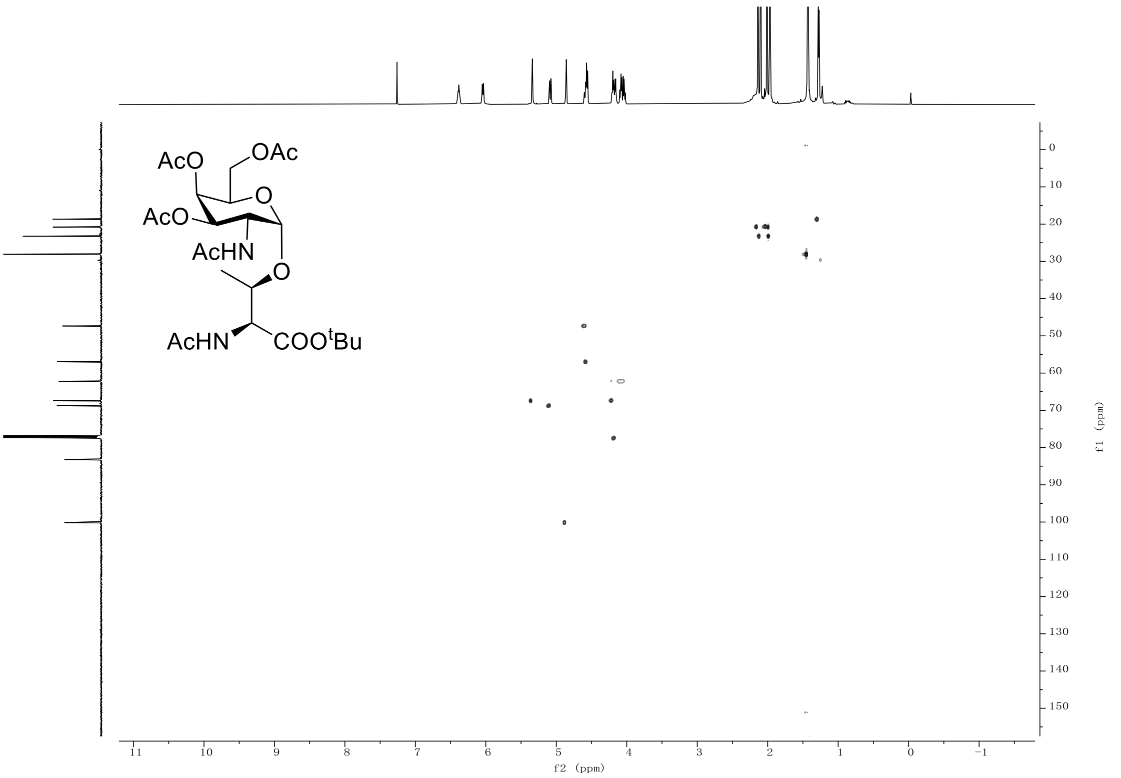

Figure S66. HSQC NMR (600/151 MHz, $CDCl_3$) spectrum of compound **S6**.

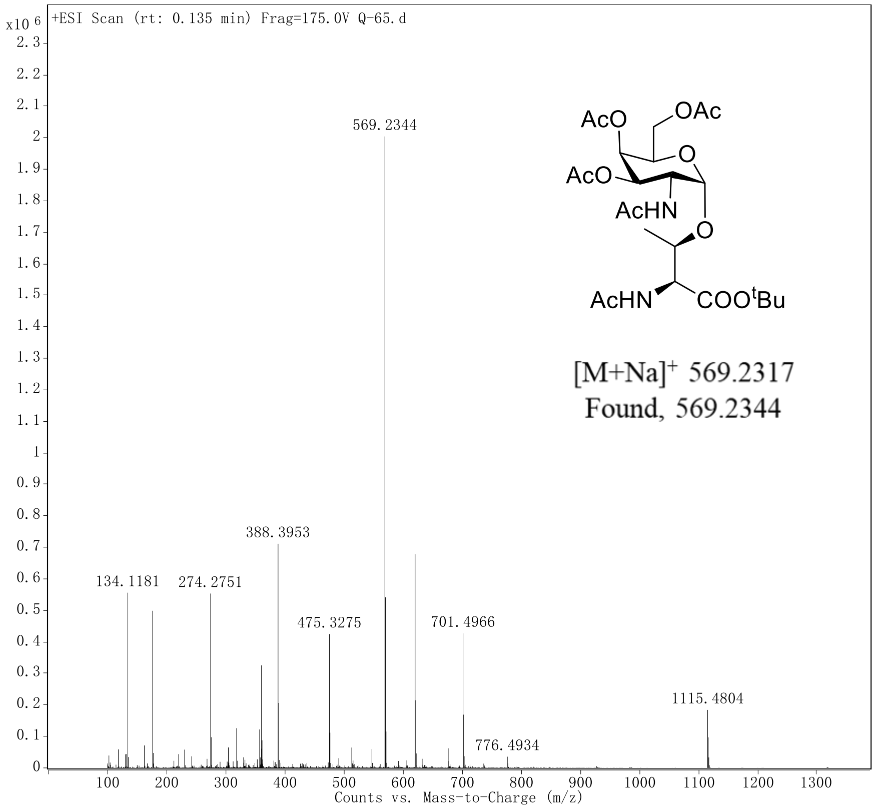

Figure S67. HRMS (ESI) spectrum of compound **S6**.

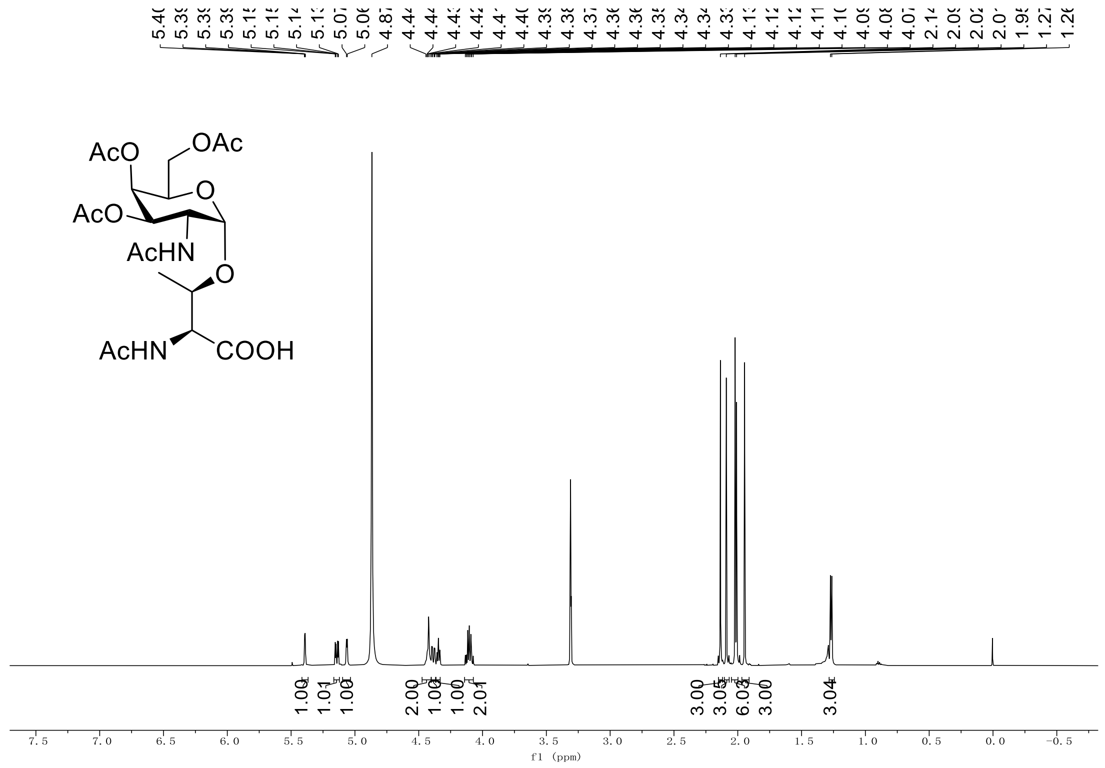

Figure S68. ^{1}H NMR (600 MHz, CD_3OD) spectrum of compound **4**.

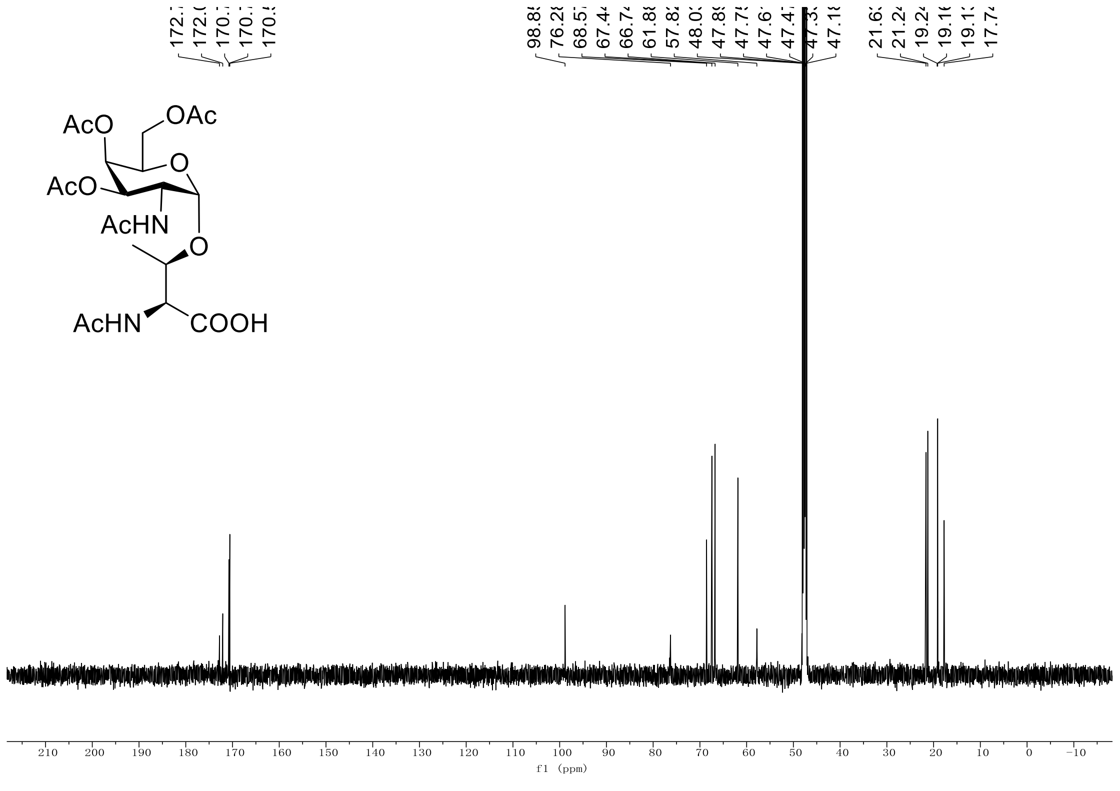

Figure S69. $^{13}C\{^{1}H\}$ NMR (151 MHz, CD_3OD) spectrum of compound **4**.

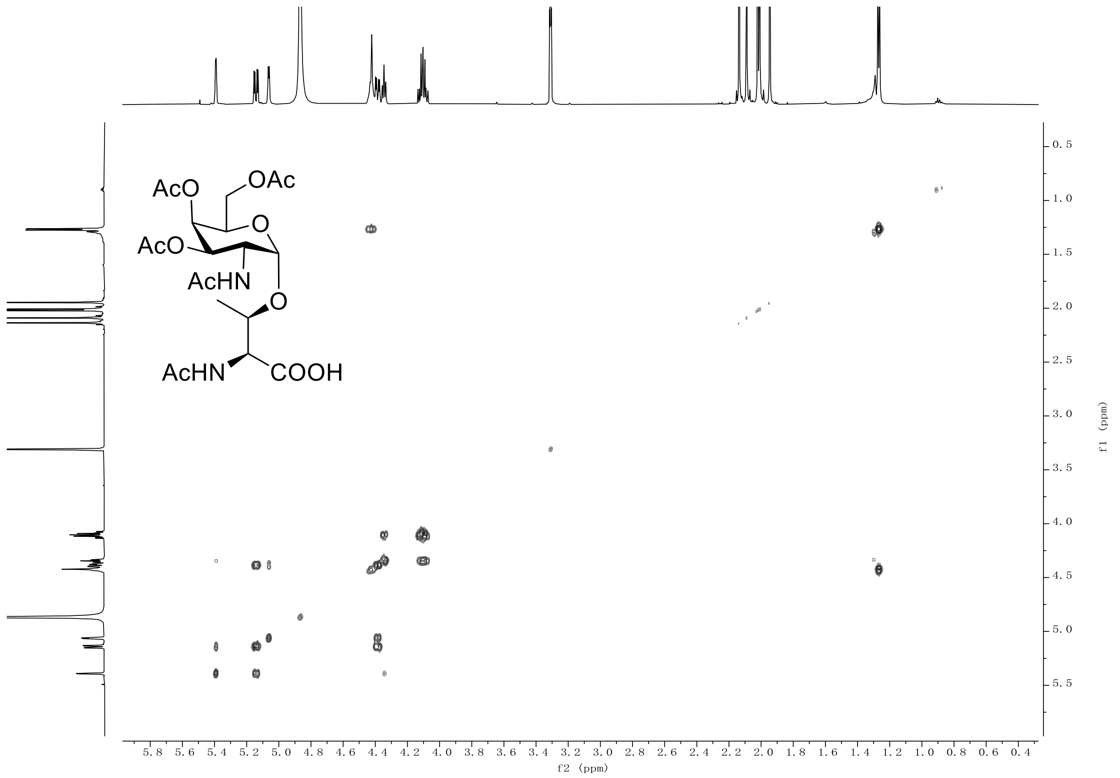

Figure S70. ^{1}H-^{1}H COSY NMR (600 MHz, CD_3OD) spectrum of compound **4**.

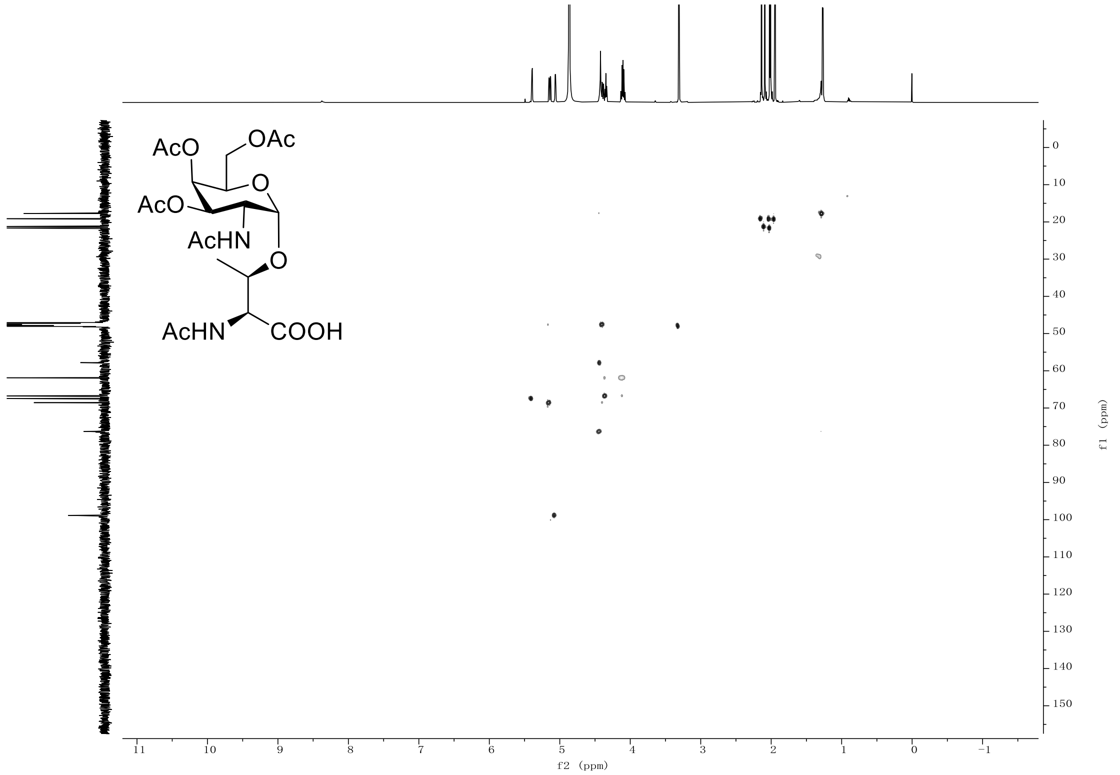

Figure S71. HSQC NMR (600/151 MHz, CD_3OD) spectrum of compound **4**.

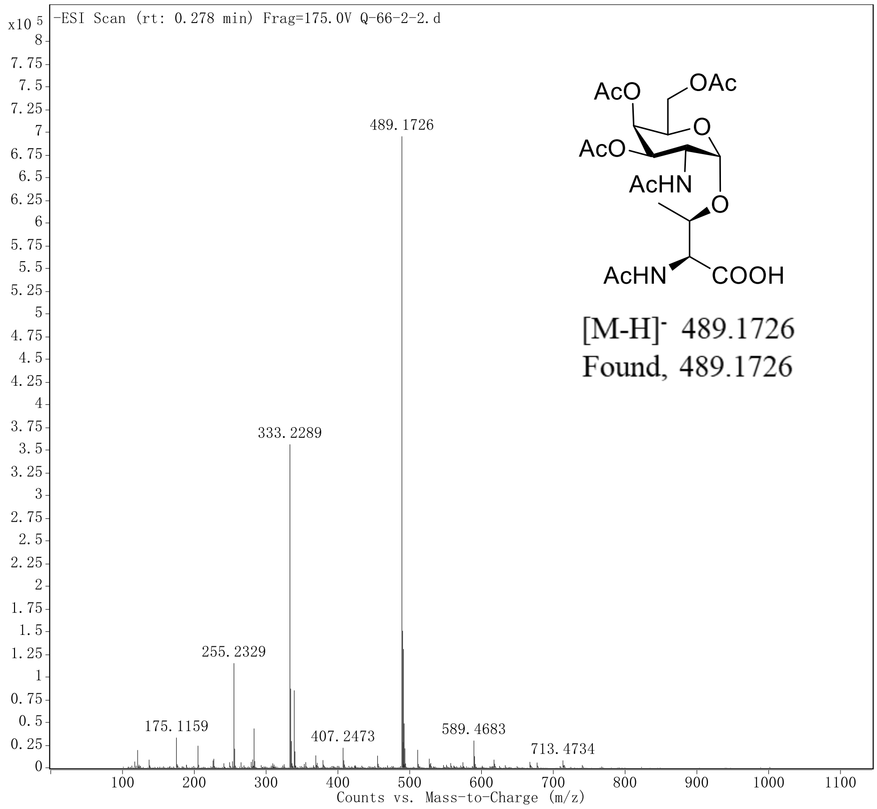

Figure S72. HRMS (ESI) spectrum of compound **4**.

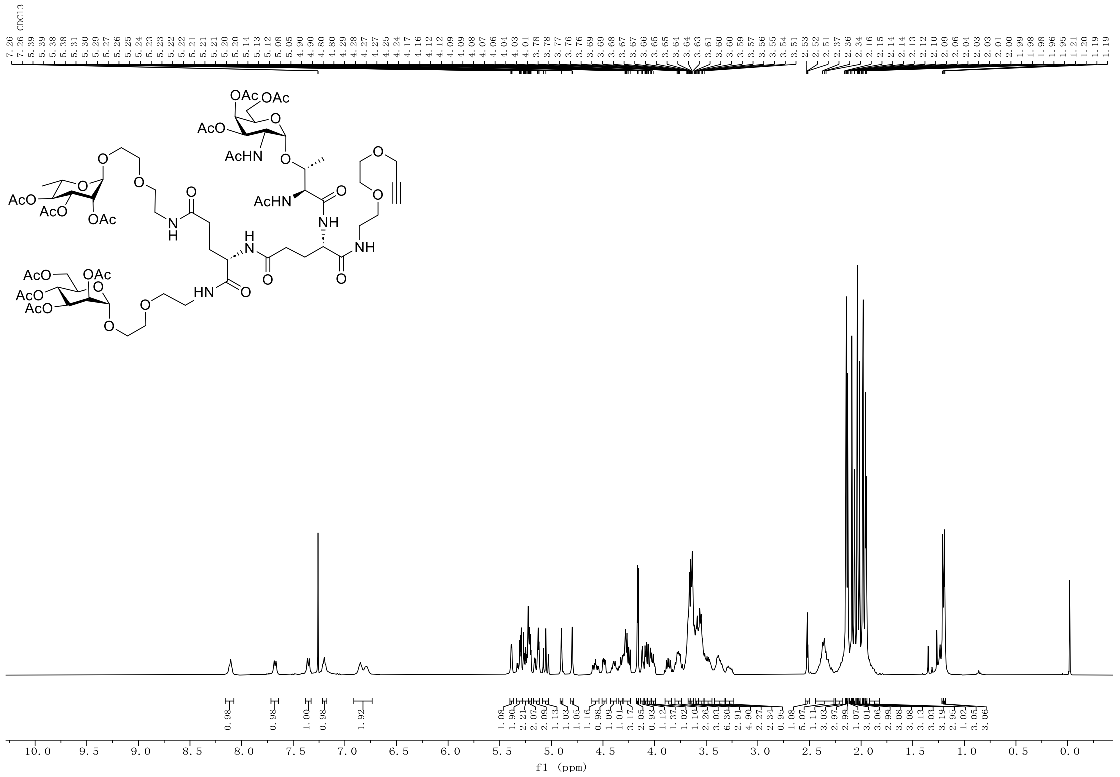

Figure S73. ^{1}H NMR (400 MHz, $CDCl_3$) spectrum of compound **21**.

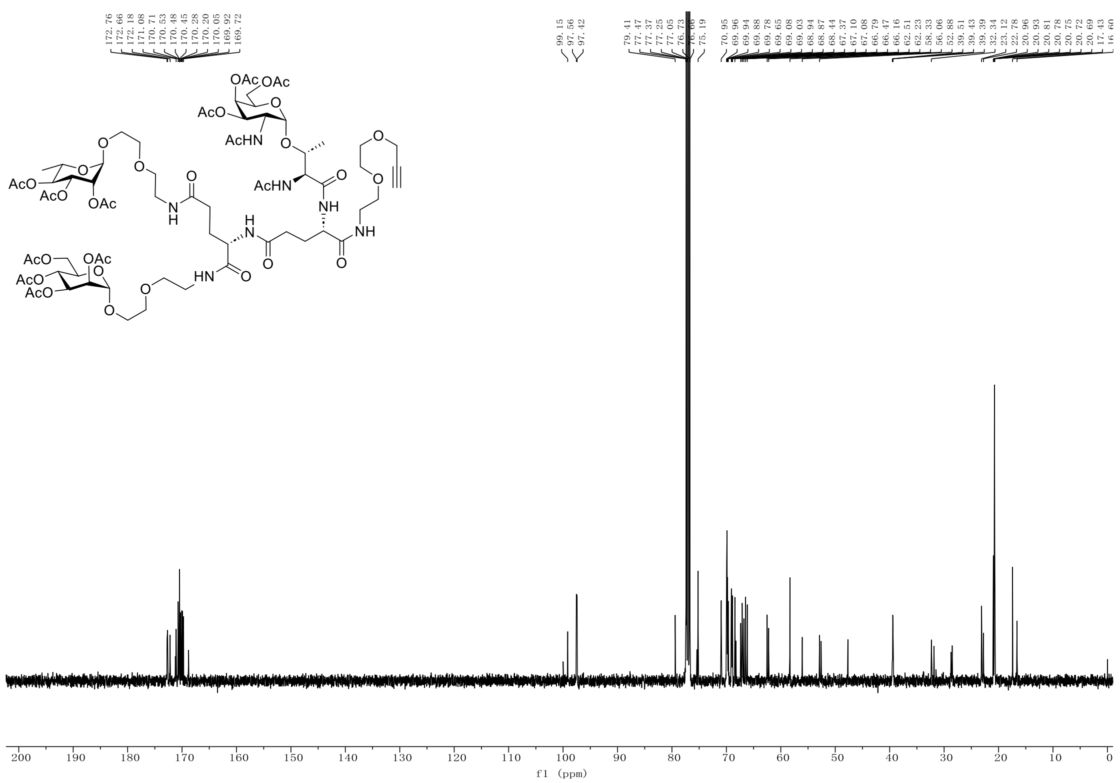

Figure S74. $^{13}C\{^{1}H\}$ NMR (101 MHz, $CDCl_3$) spectrum of compound **21**.

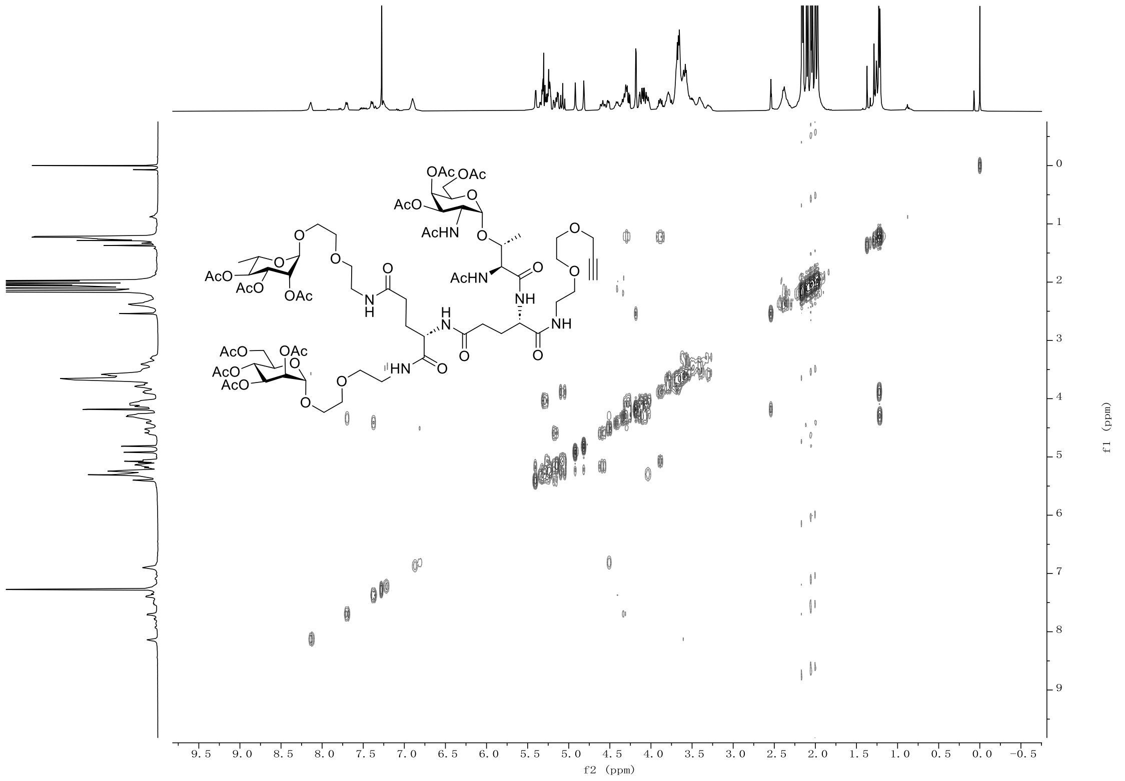

Figure S75. ^{1}H-^{1}H COSY NMR (400 MHz, $CDCl_3$) spectrum of compound **21**.

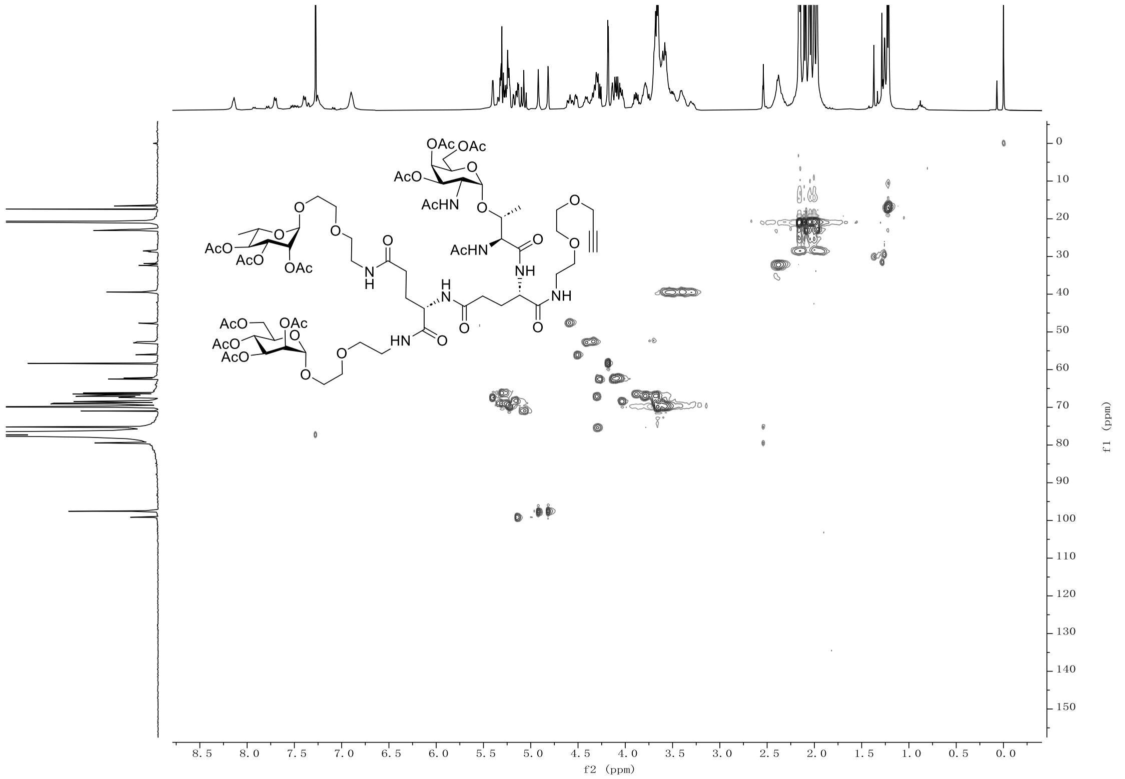

Figure S76. HSQC NMR (400/101 MHz, $CDCl_3$) spectrum of compound **21**.

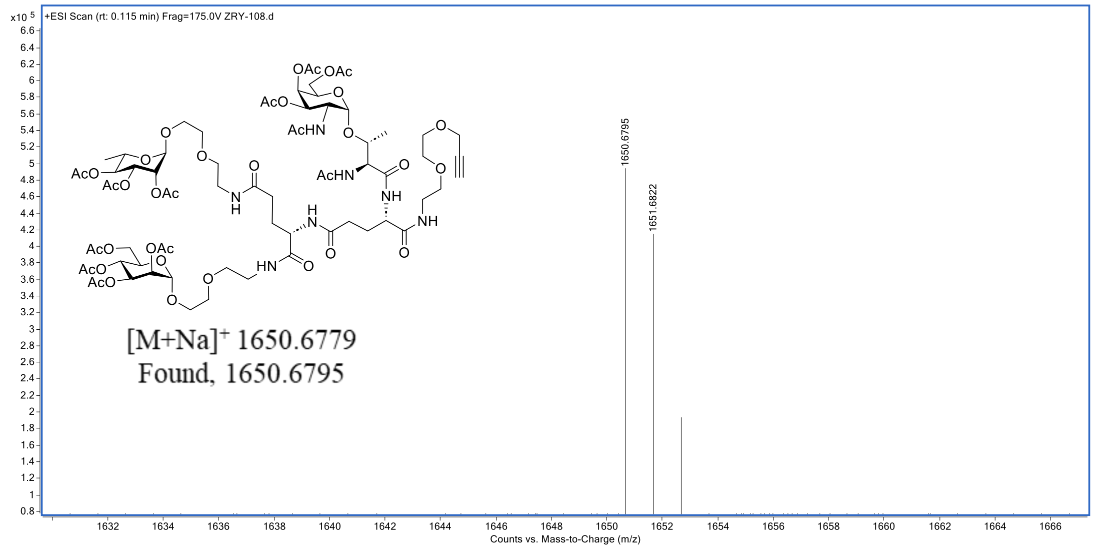

Figure S77. HRMS (ESI) spectrum of compound **21**.

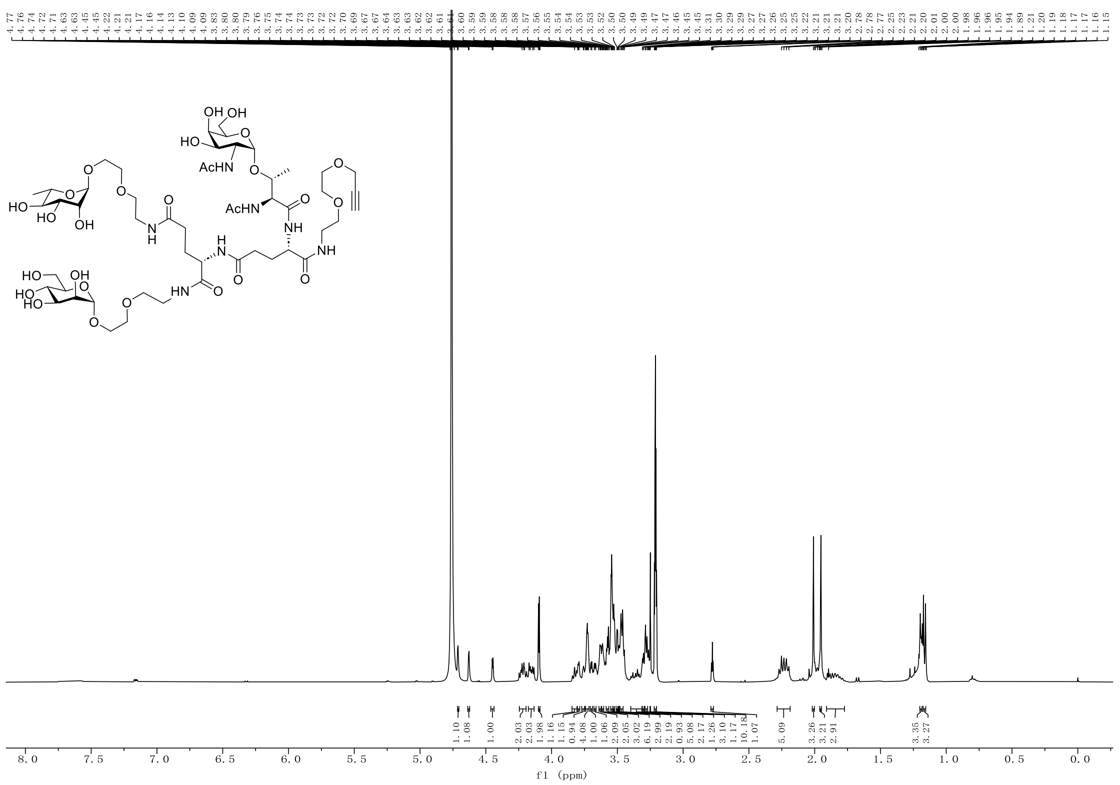

Figure S78. ^{1}H NMR (400 MHz, CD_3OD) spectrum of compound **1**.

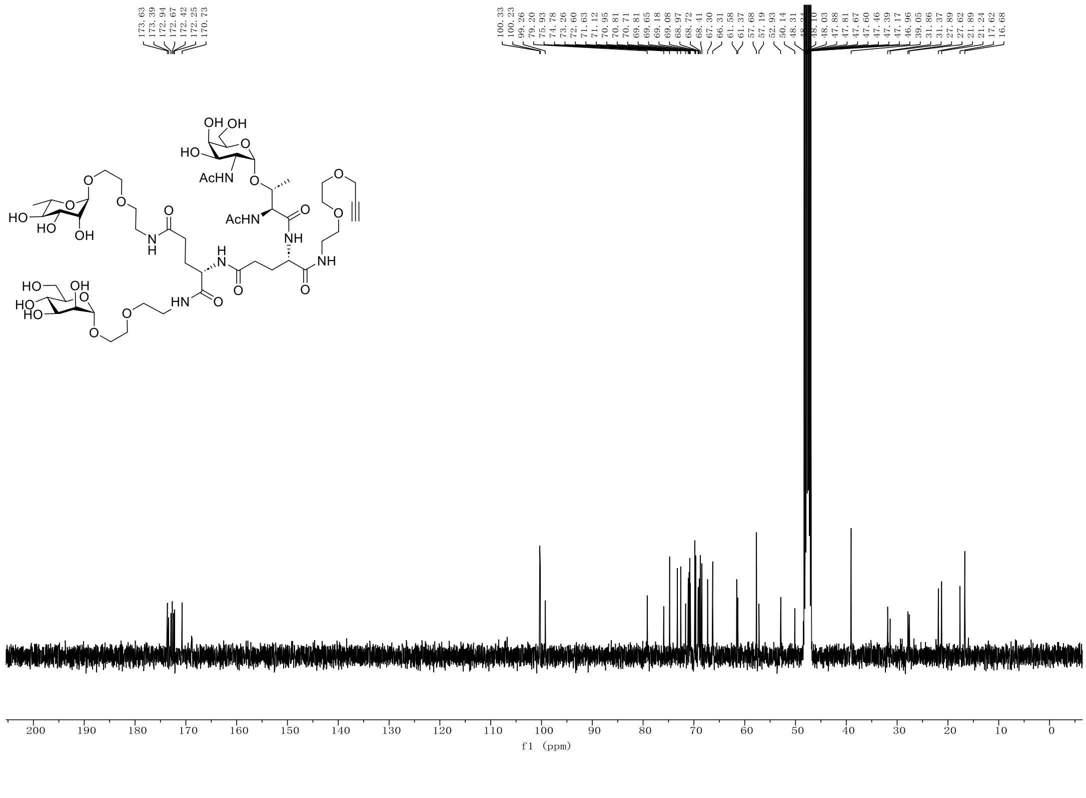

Figure S79. $^{13}C\{^{1}H\}$ NMR (101 MHz, CD_3OD) spectrum of compound **1**.

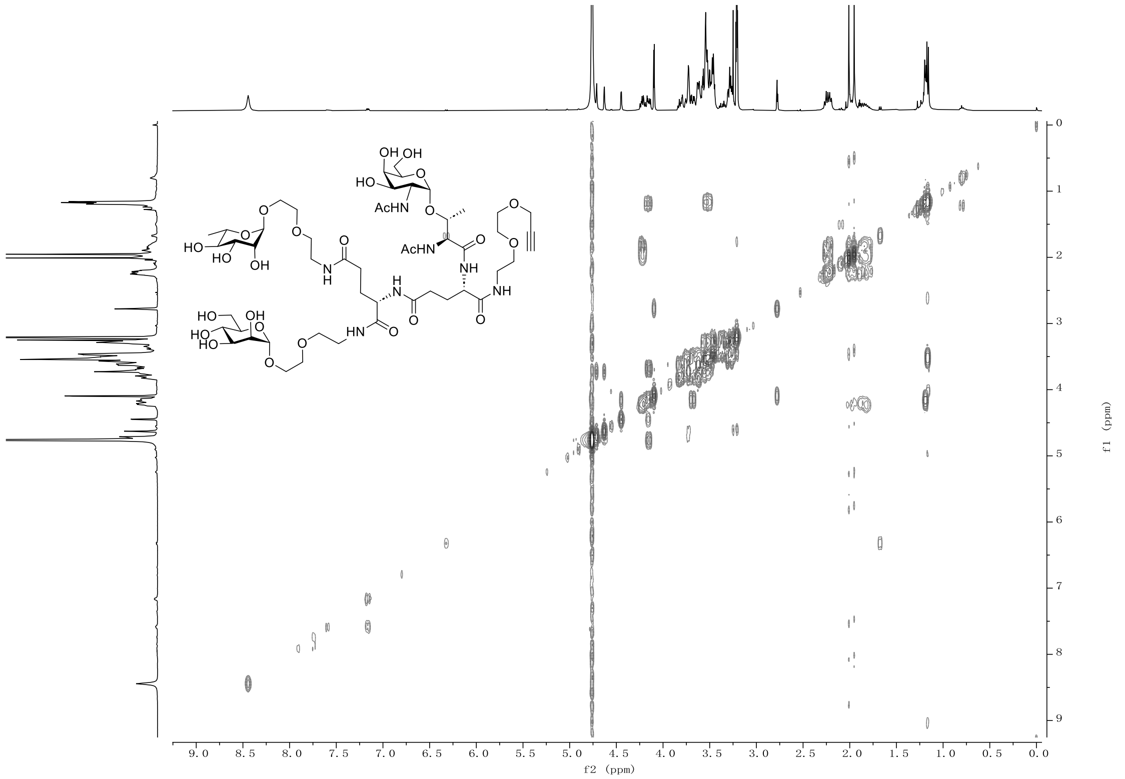

Figure S80. ^1H-^1H COSY NMR (400 MHz, CD_3OD) spectrum of compound **1**.

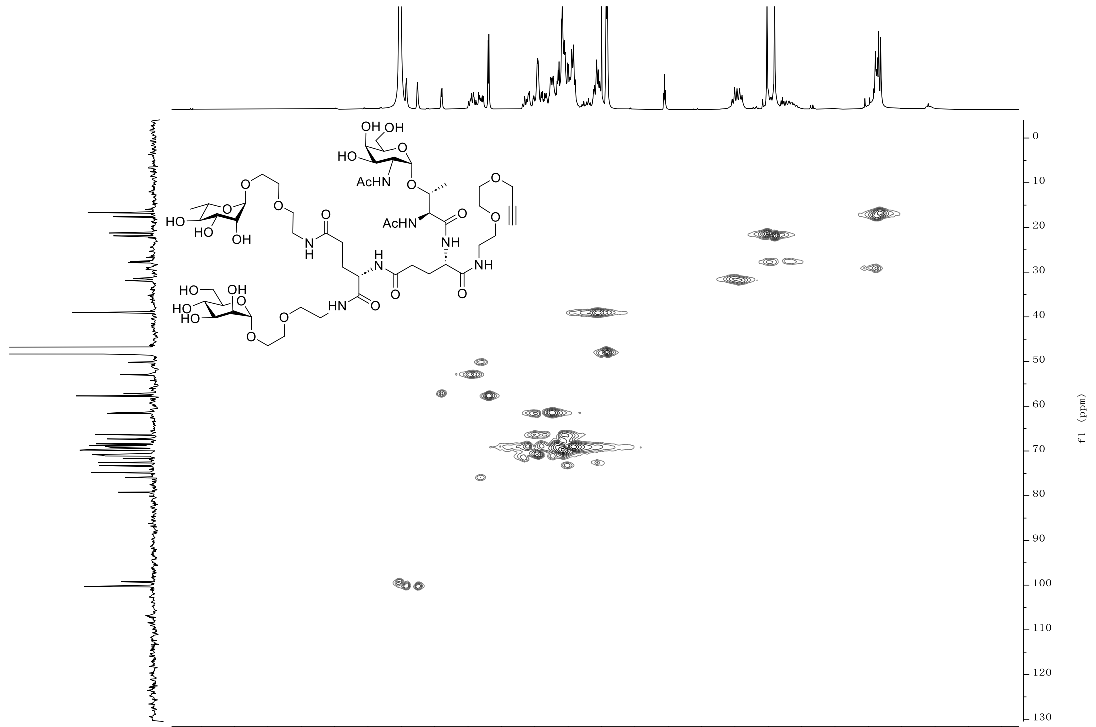

Figure S81. HSQC NMR (400/101 MHz, CD_3OD) spectrum of compound **1**.

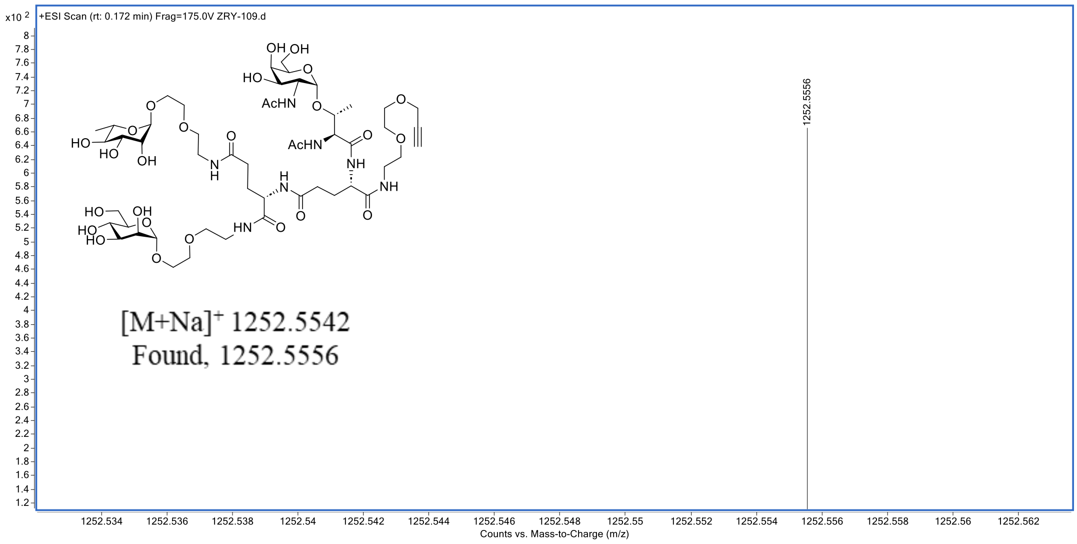

Figure S82. HRMS (ESI) spectrum of compound **1**.

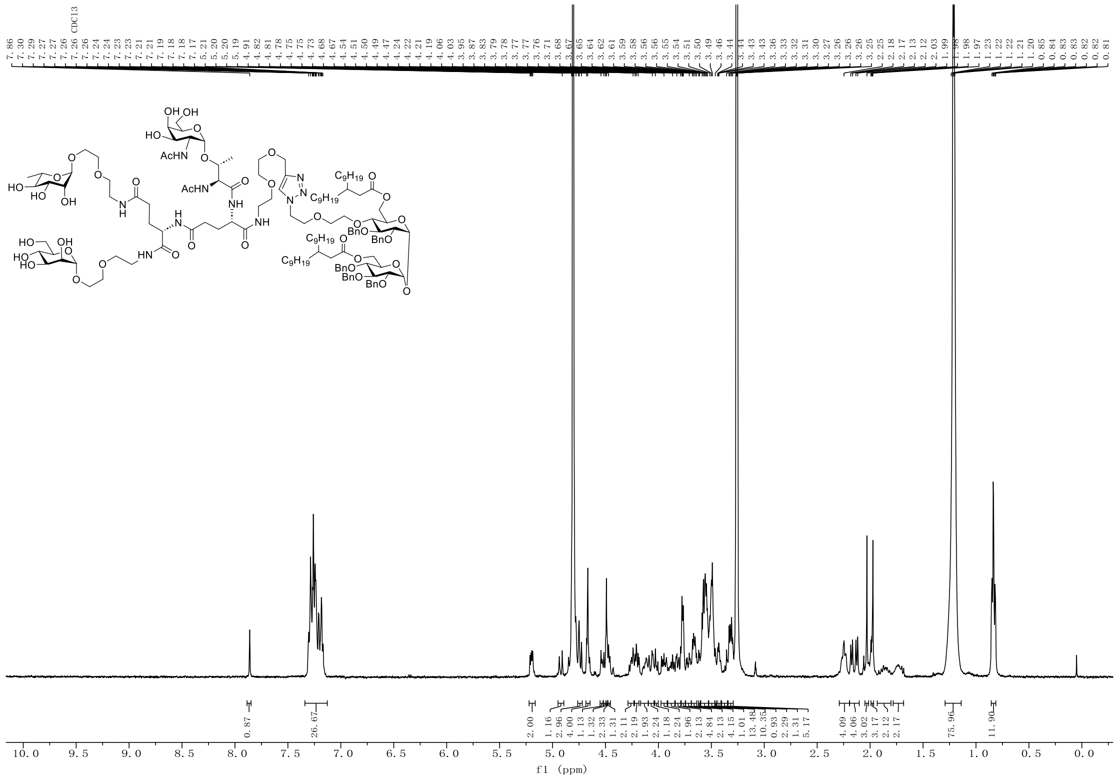

Figure S83. ^{1}H NMR (400 MHz, CD_3OD) spectrum of compound **22**.

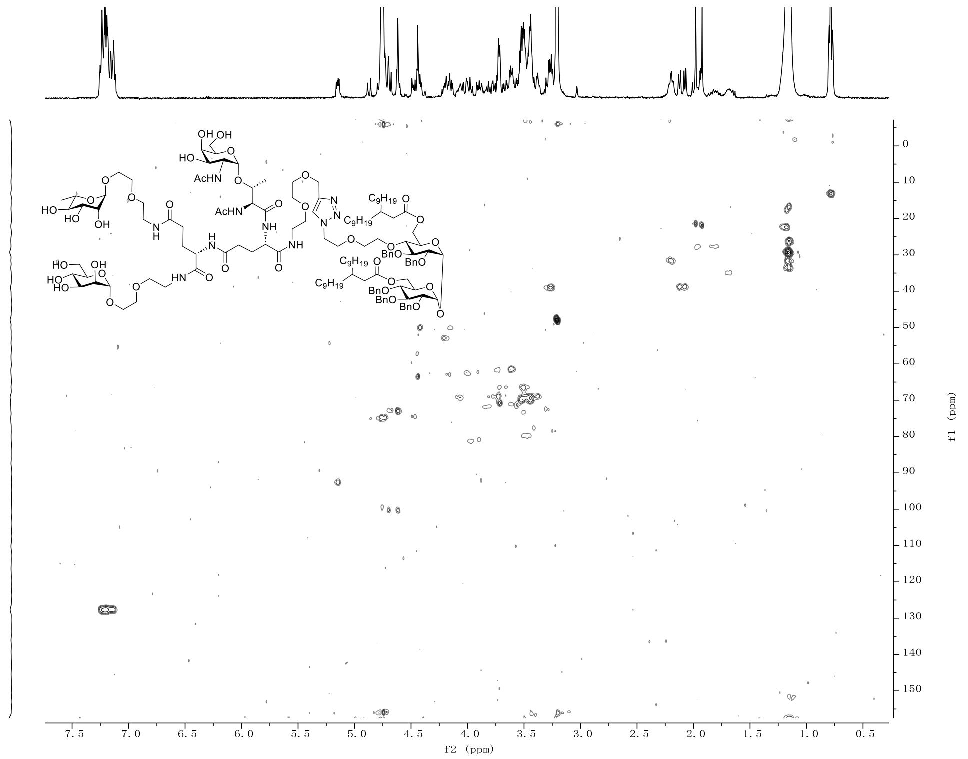

Figure S84. HSQC NMR (400/101 MHz, CD_3OD) spectrum of compound **22**.

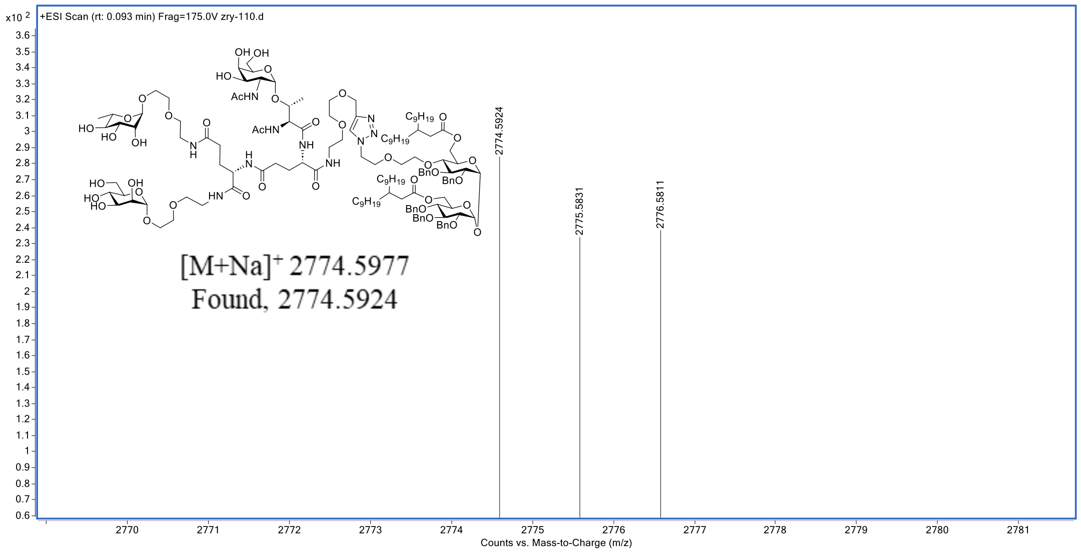

Figure S85. HRMS (ESI) spectrum of compound **22**.

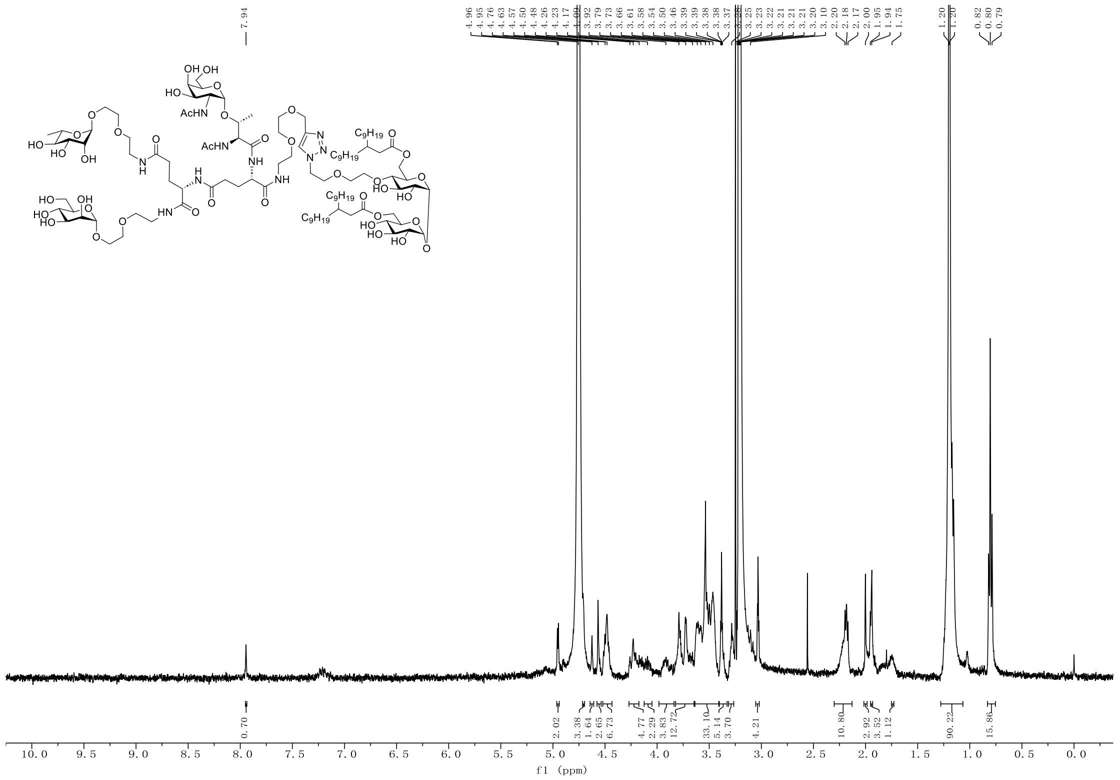

Figure S86. ^{1}H NMR (400 MHz, CD_3OD) spectrum of compound **RMVT**.

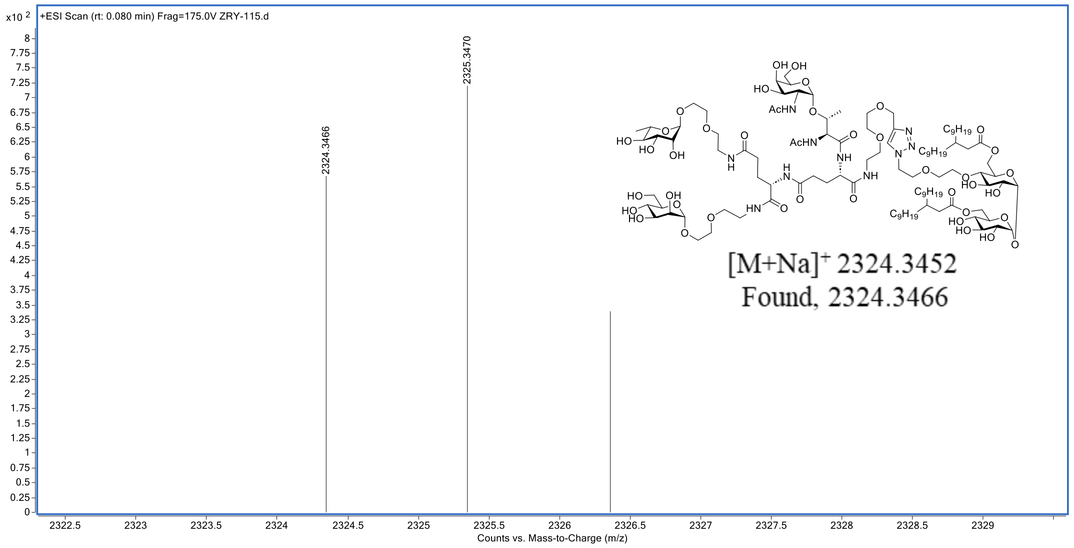

Figure S87. HRMS (ESI) spectrum of compound **RMVT**.

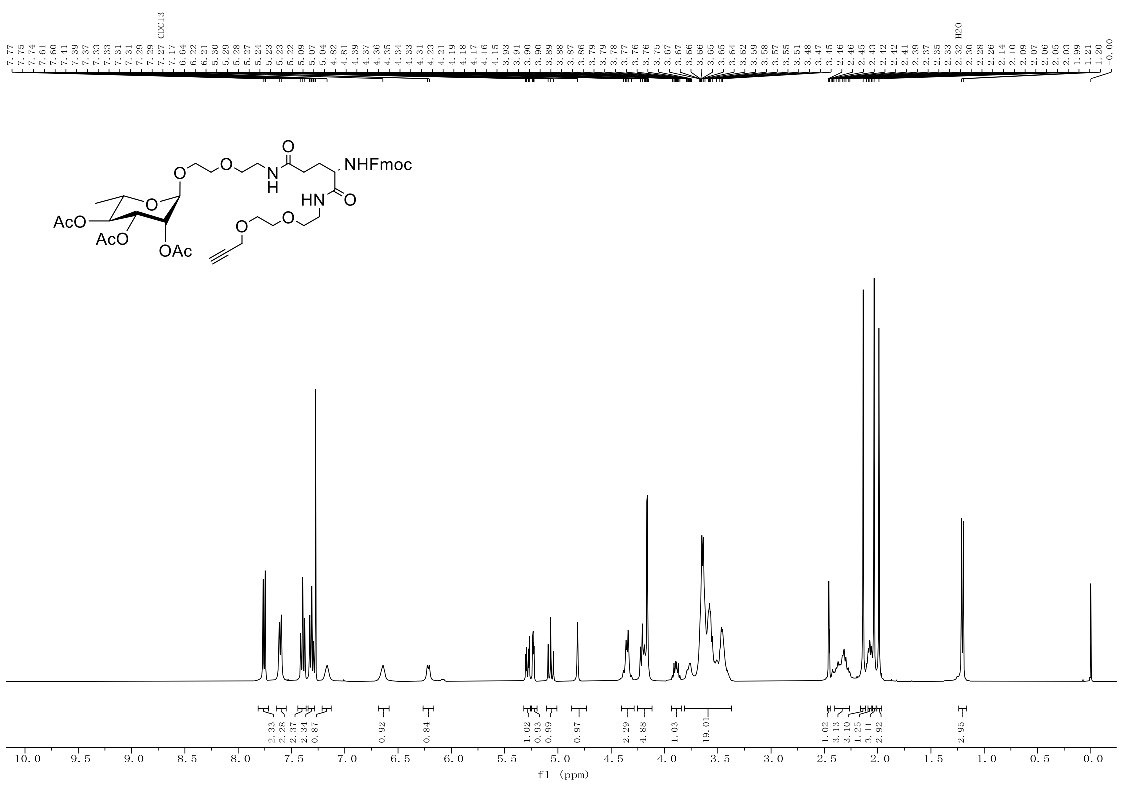

Figure S88. ^{1}H NMR (400 MHz, $CDCl_3$) spectrum of compound **23**.

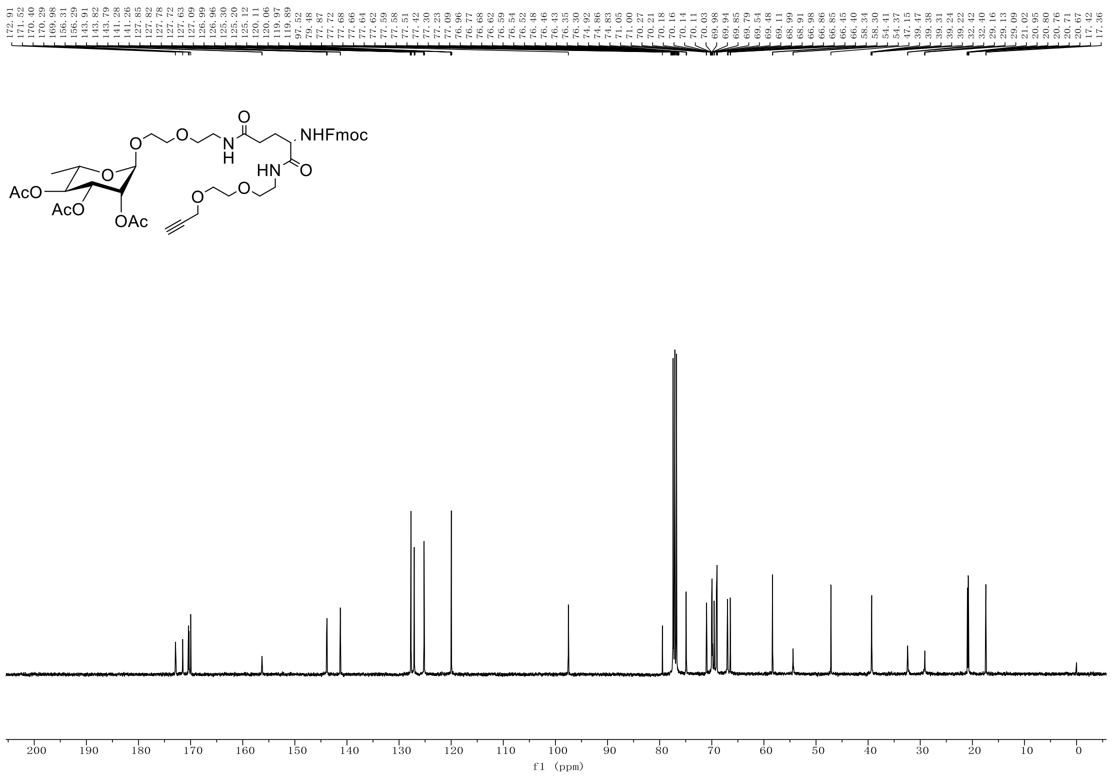

Figure S89. $^{13}C\{^{1}H\}$ NMR (101 MHz, $CDCl_3$) spectrum of compound **23**.

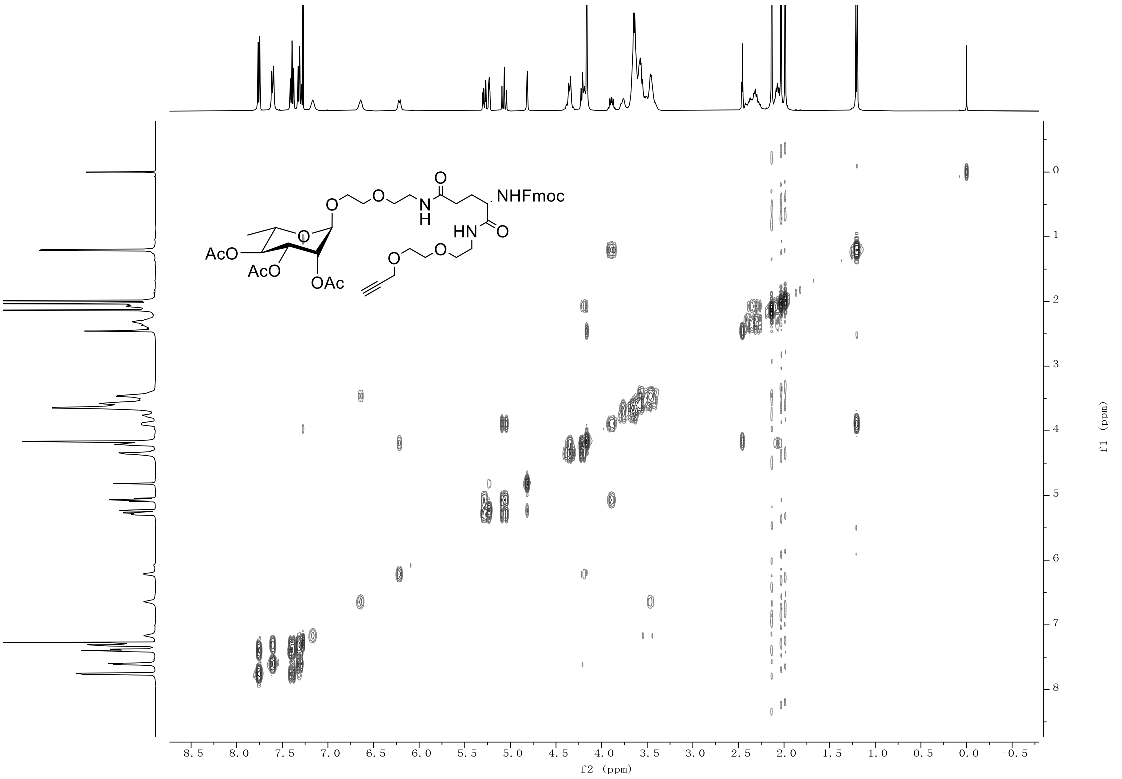

Figure S90. ^{1}H-^{1}H COSY NMR (400 MHz, $CDCl_3$) spectrum of compound **23**.

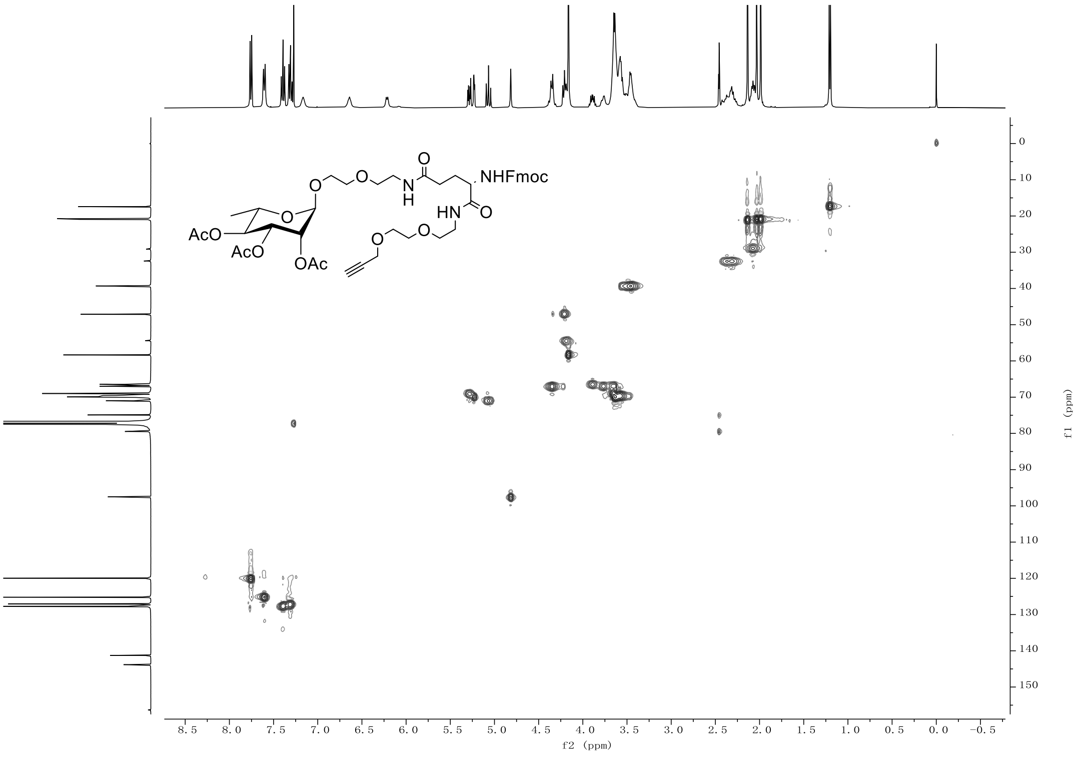

Figure S91. HSQC NMR (400/101 MHz, $CDCl_3$) spectrum of compound **23**.

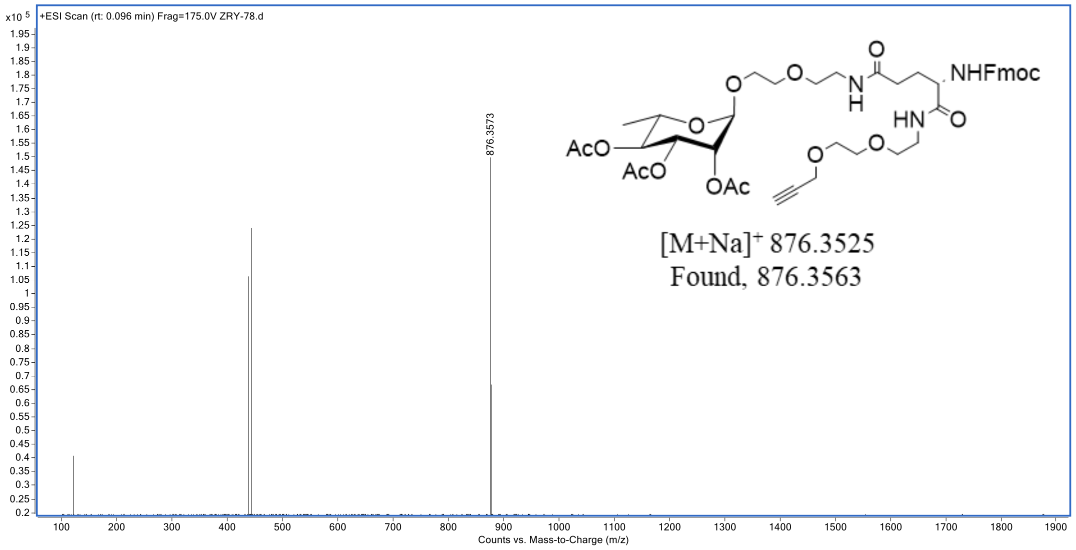

Figure S92. HRMS (ESI) spectrum of compound **23**.

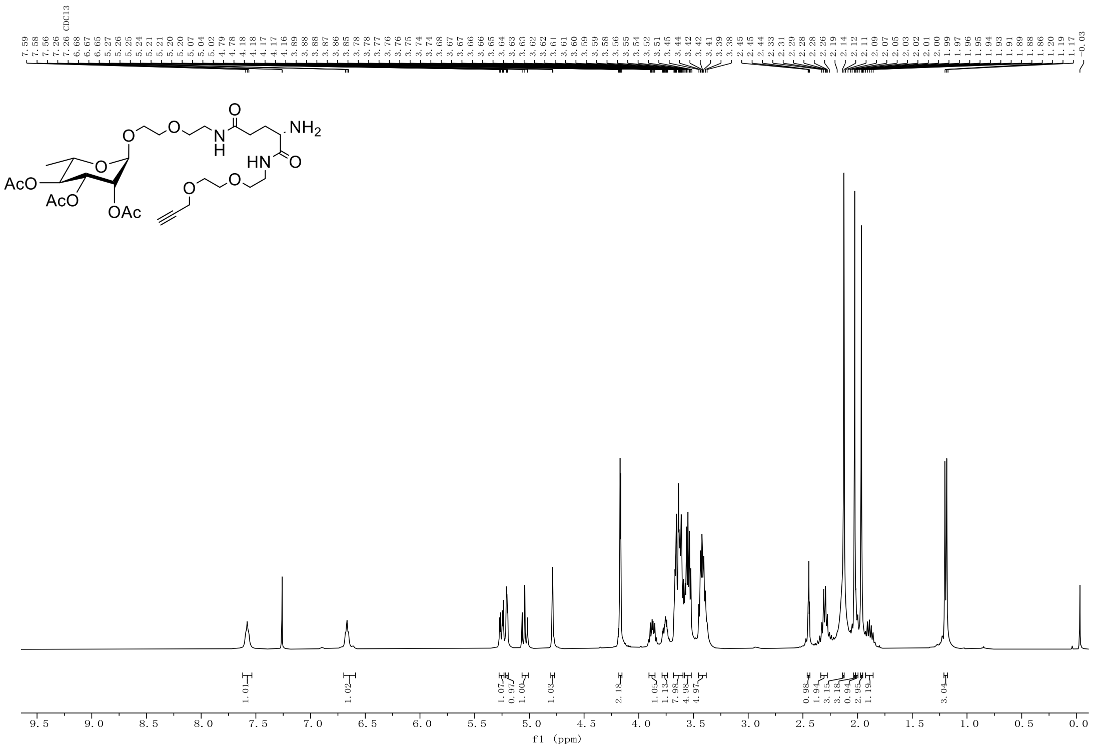

Figure S93. ^{1}H NMR (400 MHz, $CDCl_3$) spectrum of compound **24**.

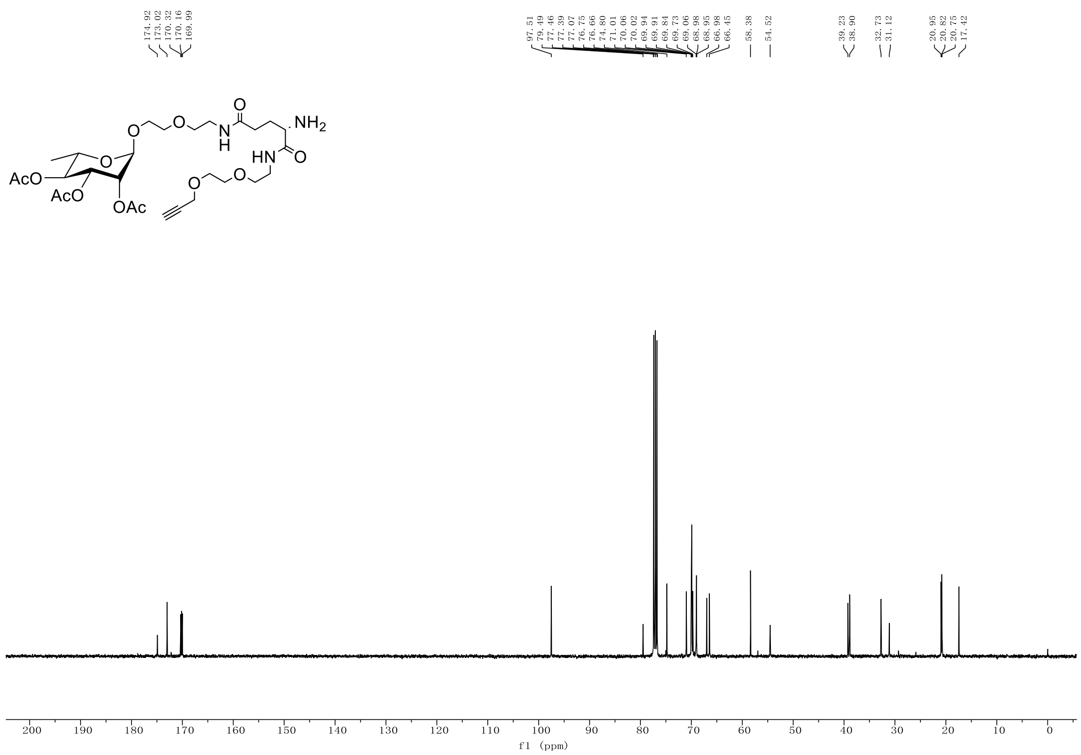

Figure S94. $^{13}C\{^1H\}$ NMR (101 MHz, $CDCl_3$) spectrum of compound **24**.

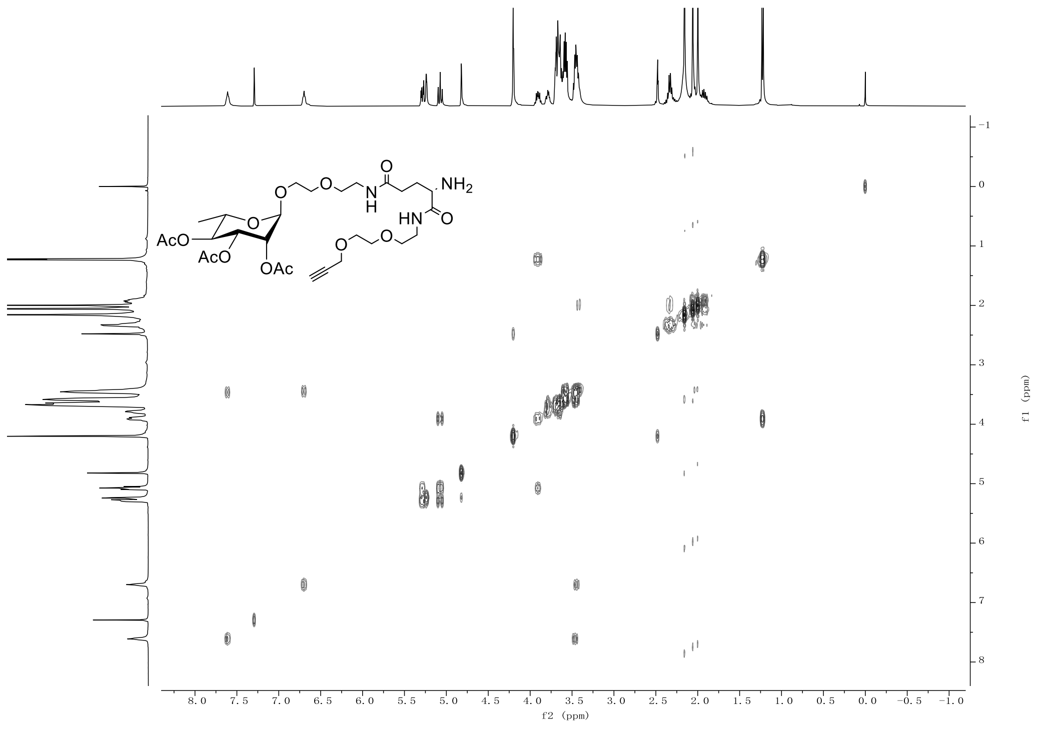

Figure S95. ^1H-^1H COSY NMR (400 MHz, $CDCl_3$) spectrum of compound **24**.

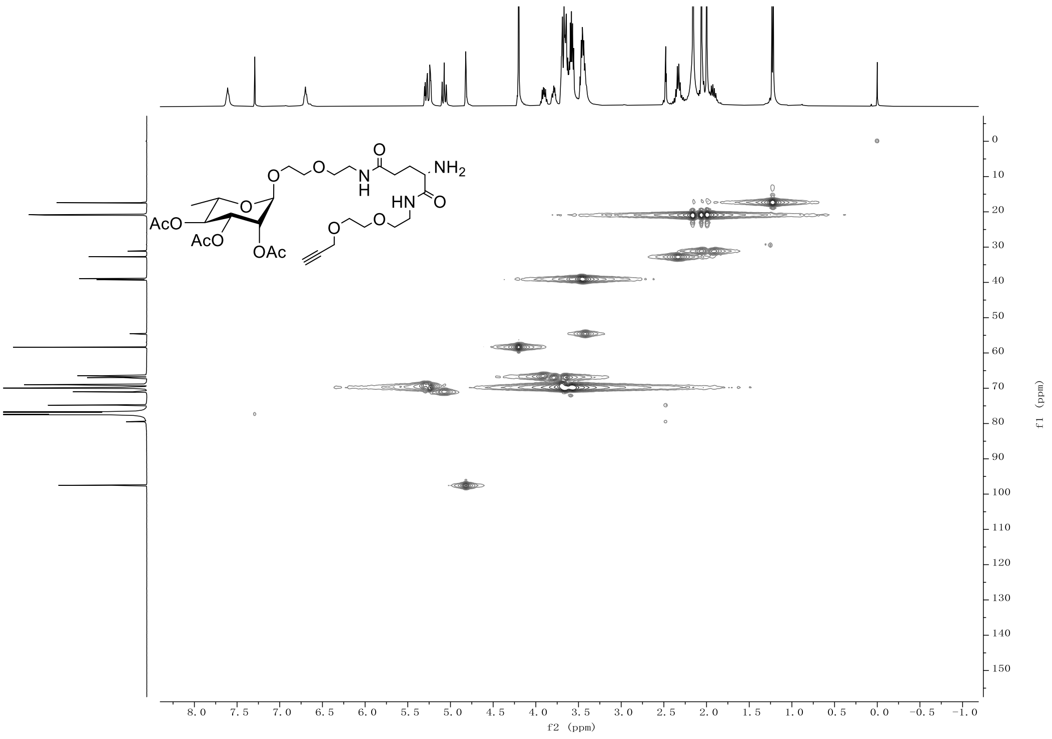

Figure S96. HSQC NMR (400/101 MHz, $CDCl_3$) spectrum of compound **24**.

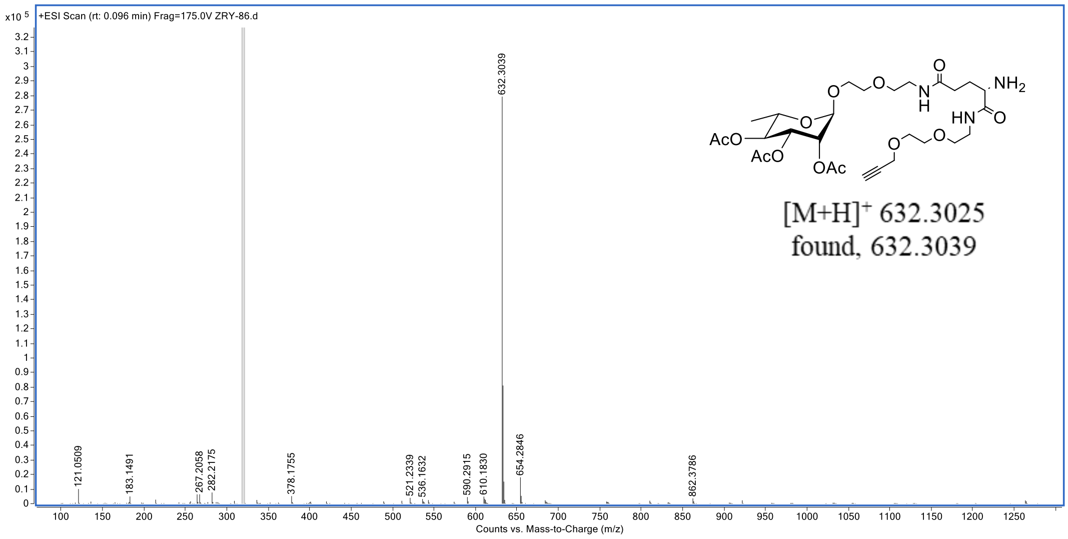

Figure S97. HRMS (ESI) spectrum of compound **24**.

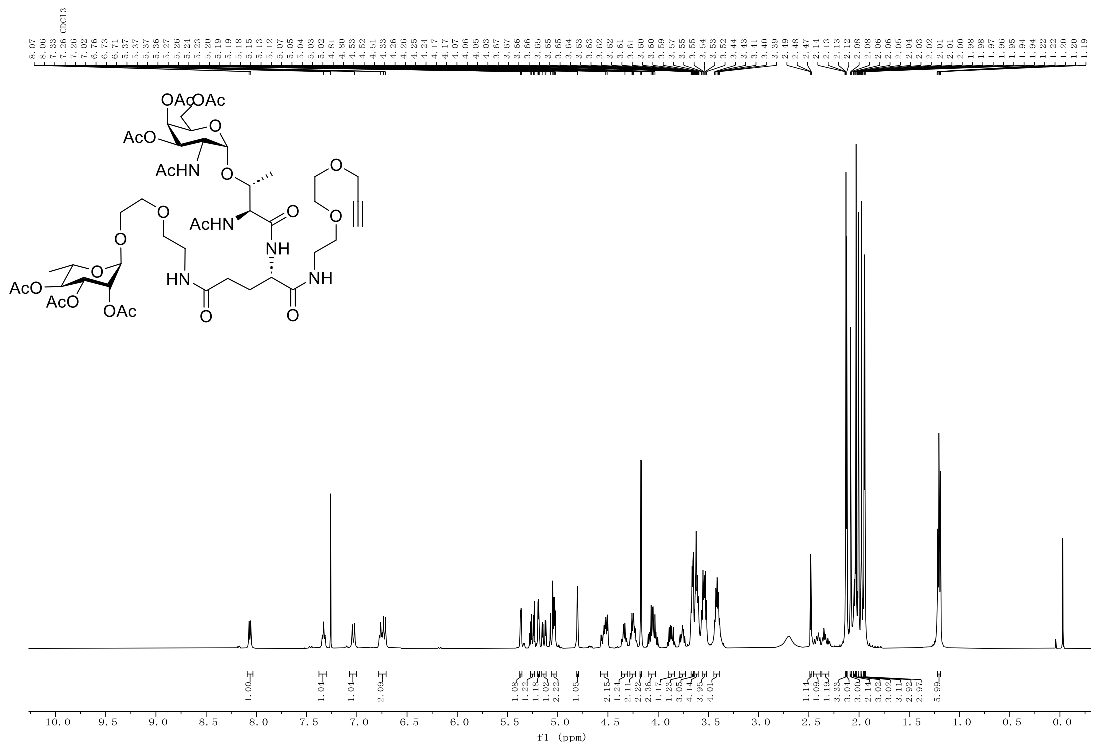

Figure S98. ^{1}H NMR (400 MHz, $CDCl_3$) spectrum of compound **25**.

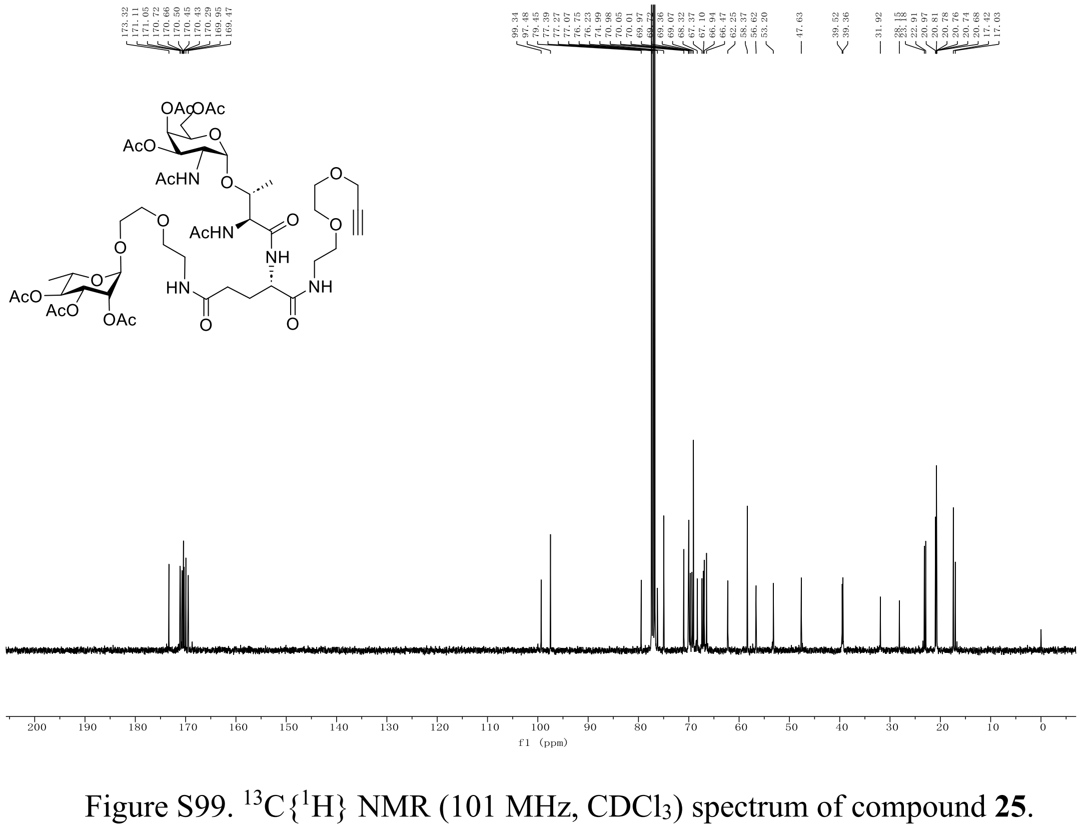

Figure S99. $^{13}C\{^{1}H\}$ NMR (101 MHz, $CDCl_3$) spectrum of compound **25**.

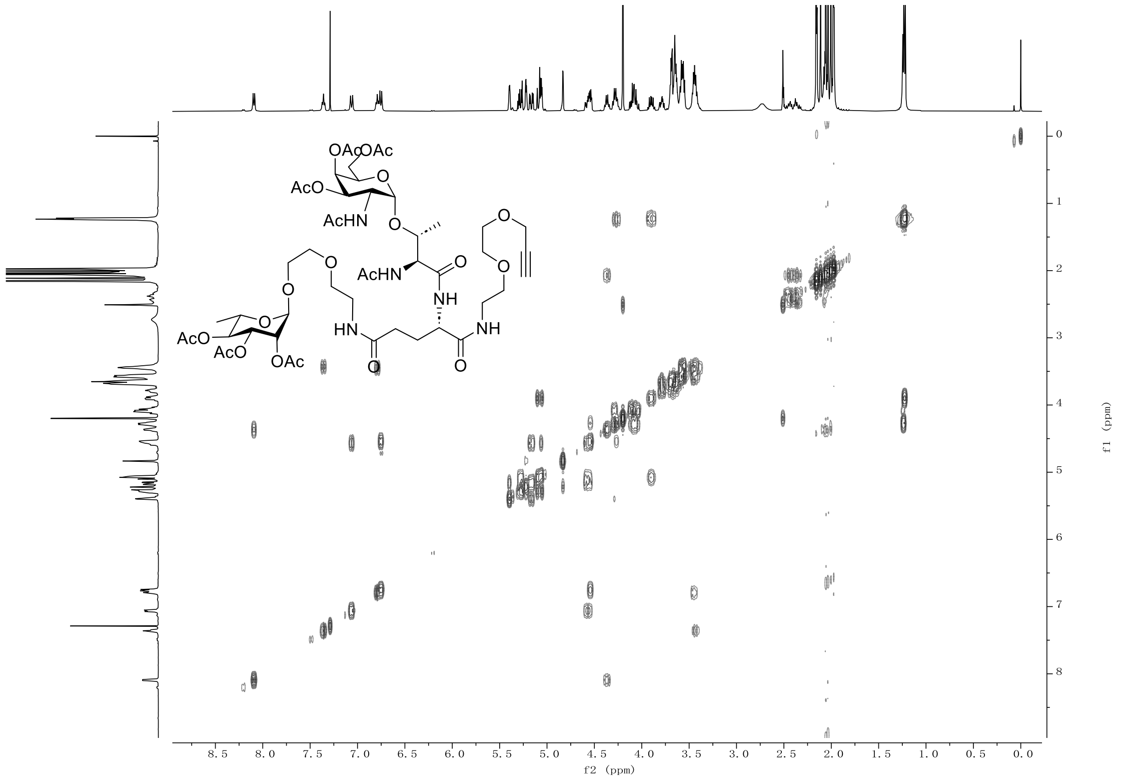

Figure S100. ^{1}H-^{1}H COSY NMR (400 MHz, $CDCl_3$) spectrum of compound **25**.

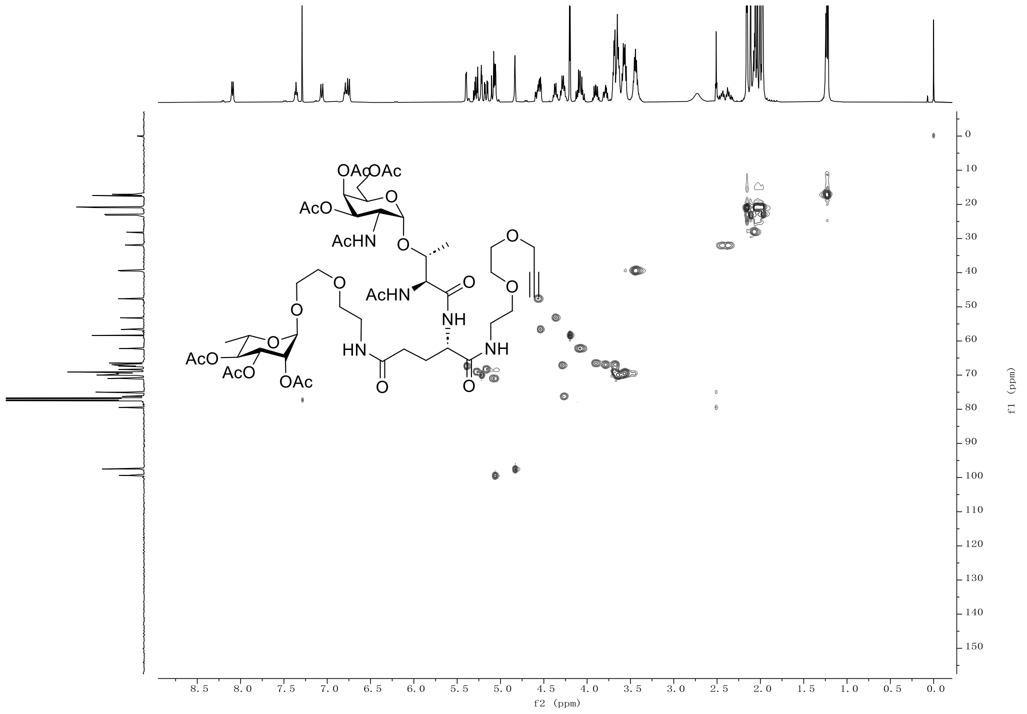

Figure S101. HSQC NMR (400/101 MHz, $CDCl_3$) spectrum of compound **25**.

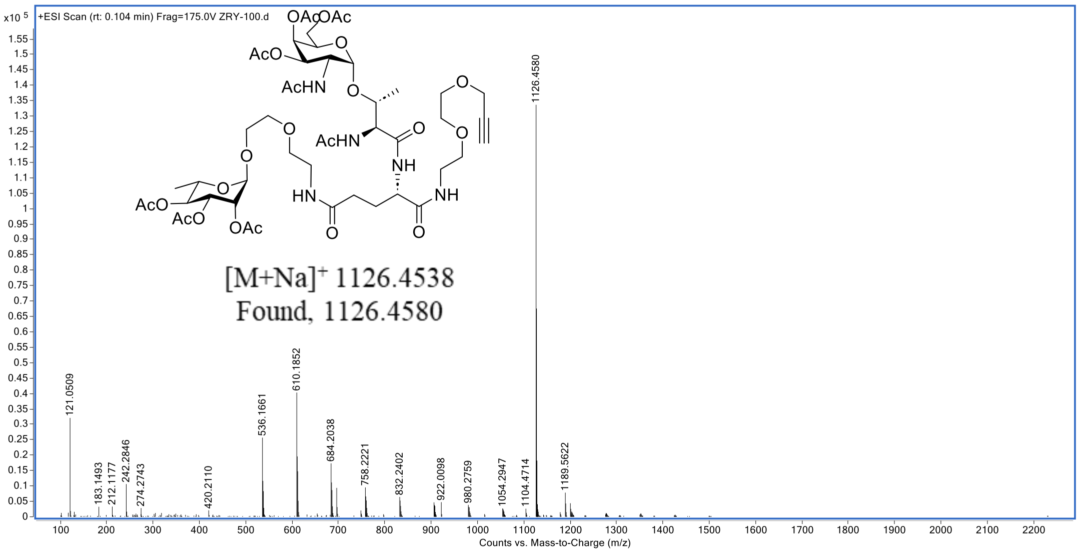

Figure S102. HRMS (ESI) spectrum of compound **25**.

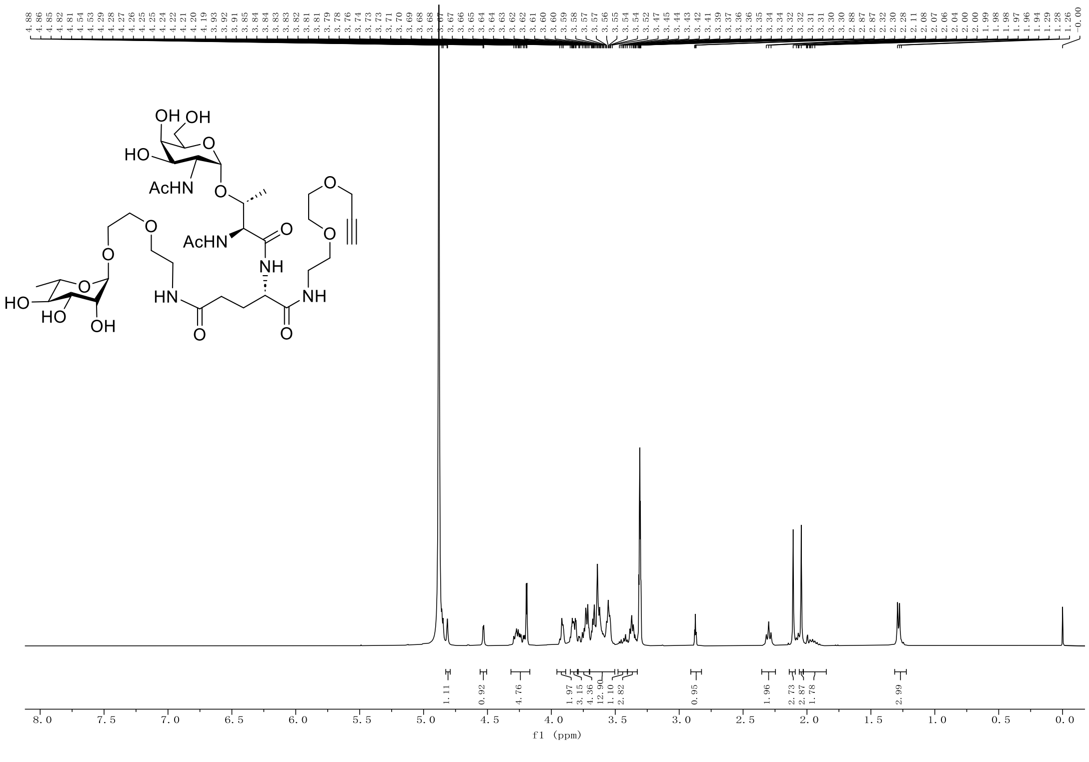

Figure S103. ^{1}H NMR (400 MHz, CD_3OD) spectrum of compound **26**.

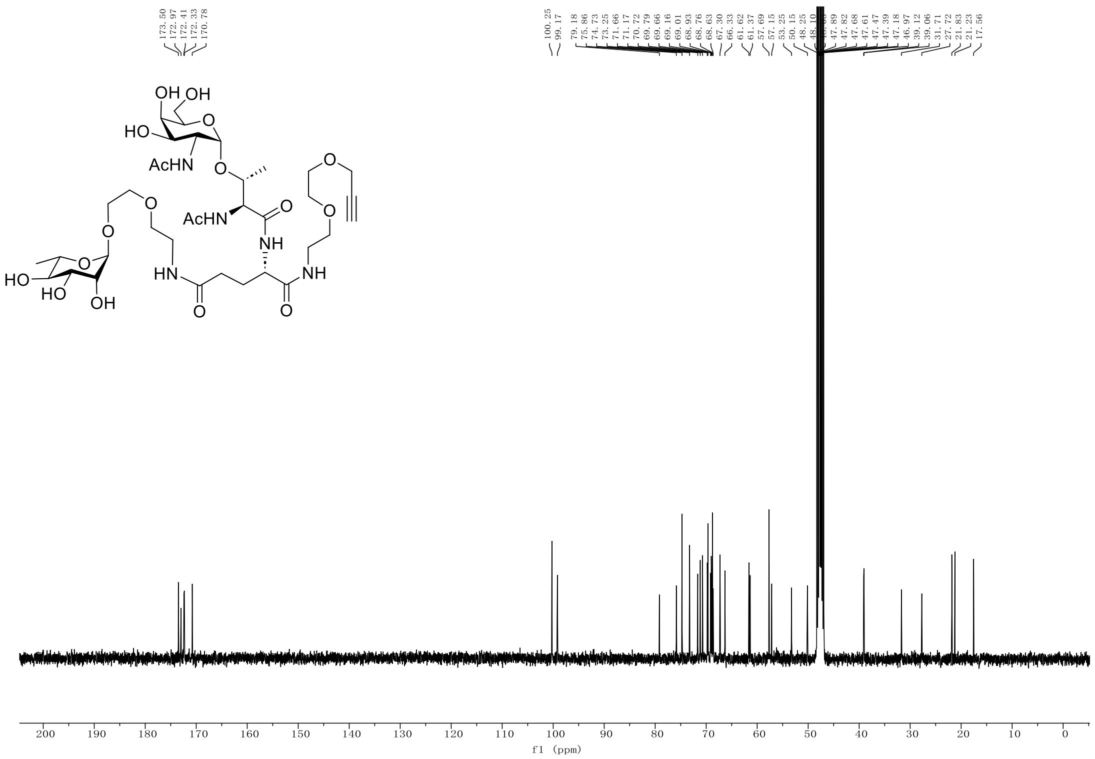

Figure S104. $^{13}C\{^{1}H\}$ NMR (101 MHz, CD_3OD) spectrum of compound **26**.

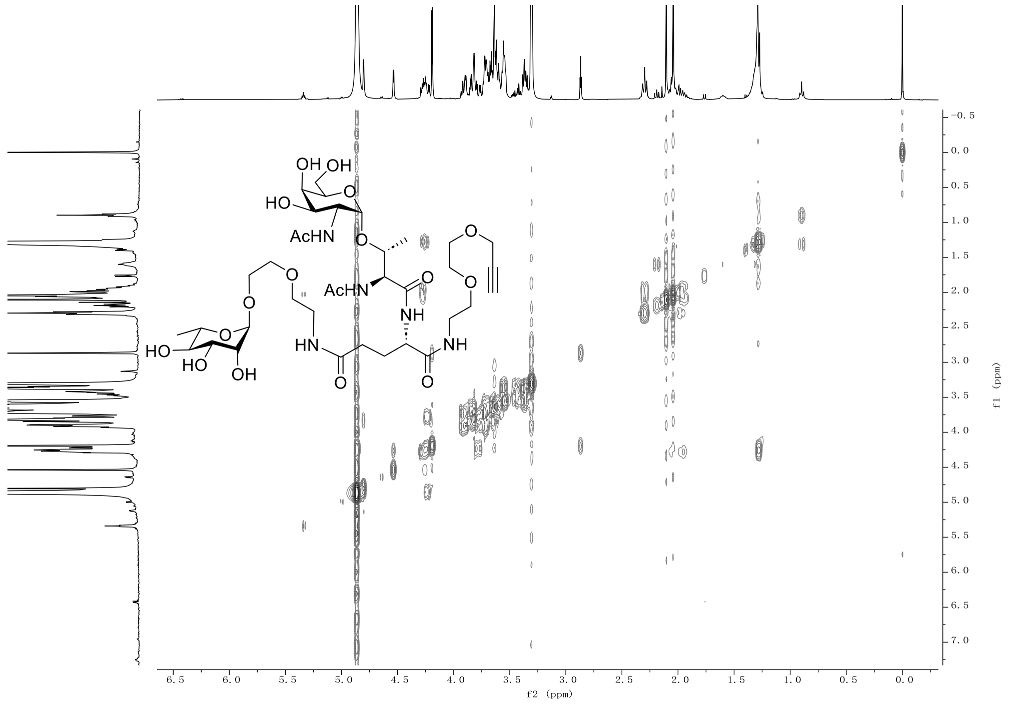

Figure S105. ^{1}H-^{1}H COSY NMR (400 MHz, CD_3OD) spectrum of compound **26**.

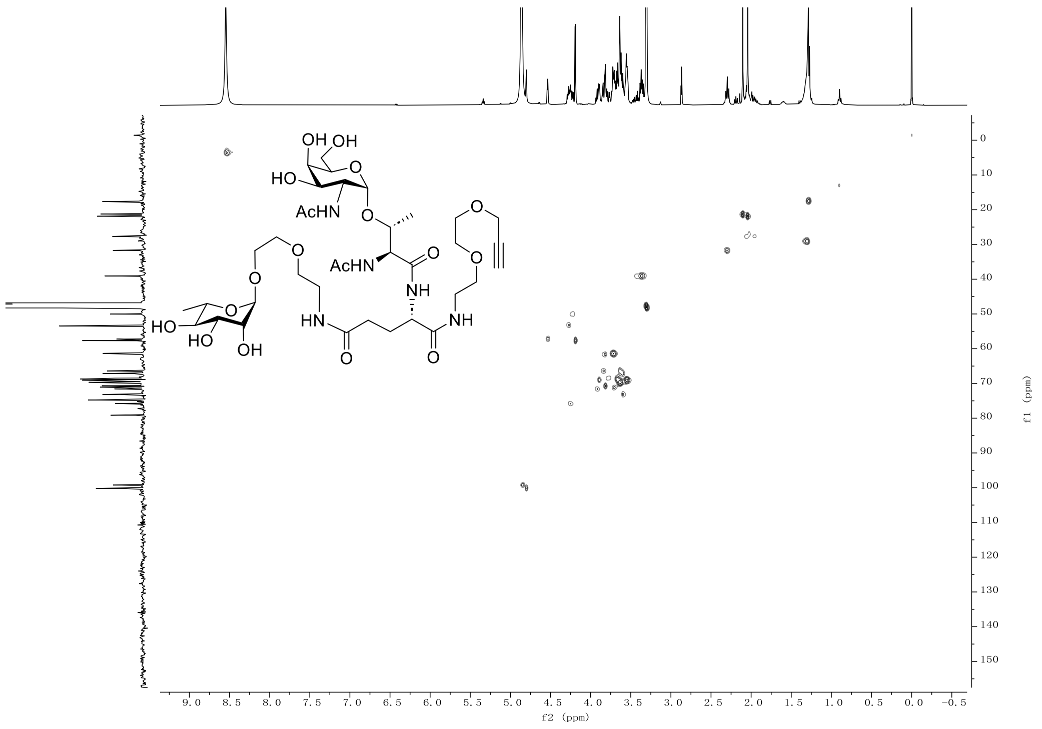

Figure S106. HSQC NMR (400/101 MHz, CD_3OD) spectrum of compound **26**.

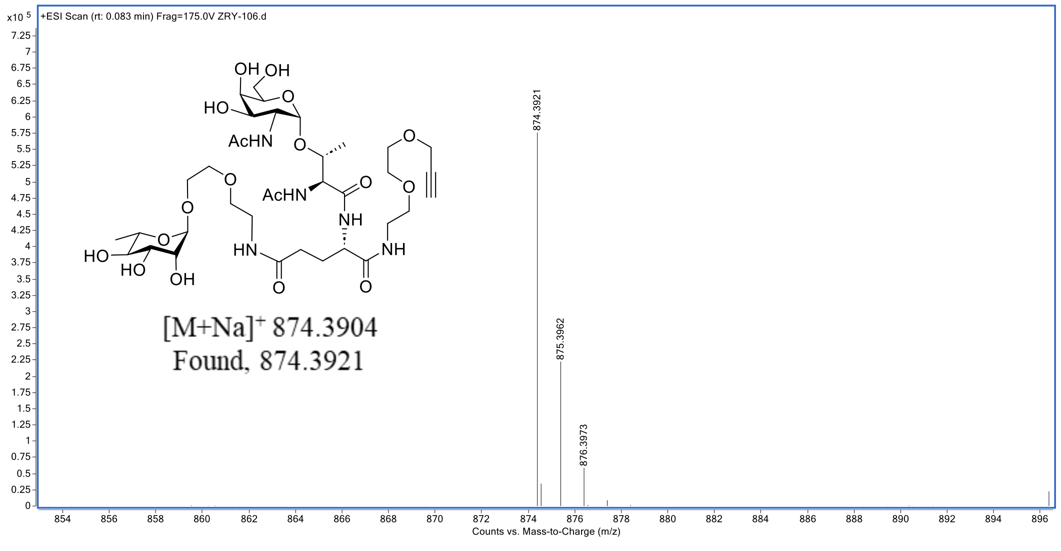

Figure S107. HRMS (ESI) spectrum of compound **26**.

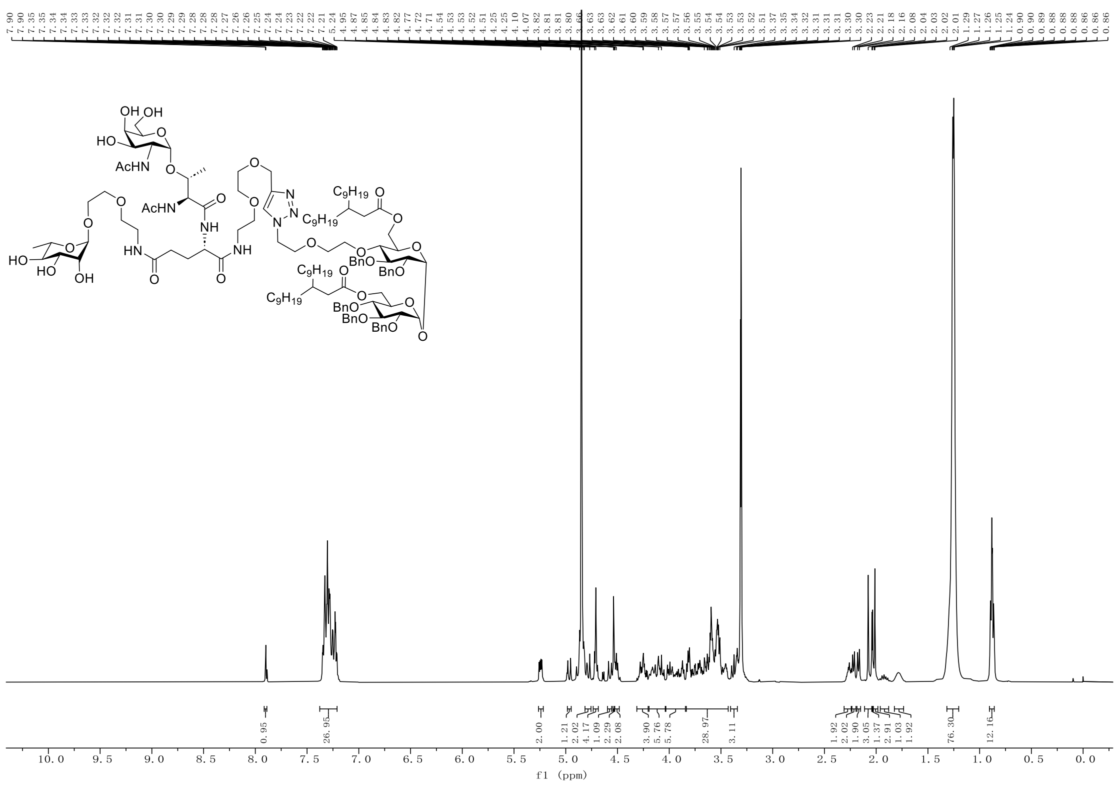

Figure S108. ^{1}H NMR (400 MHz, $CD_3OD/CDCl_3$) spectrum of compound **27**.

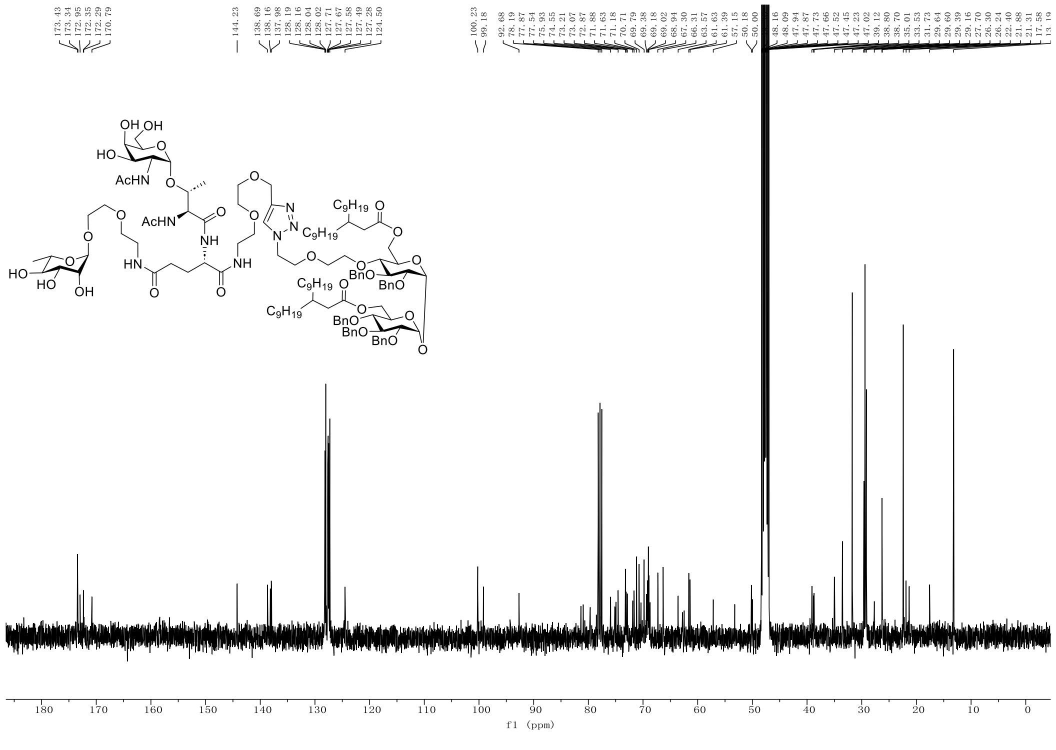

Figure S109. $^{13}C\{^{1}H\}$ NMR (101 MHz, $CD_3OD/CDCl_3$) spectrum of compound **27**.

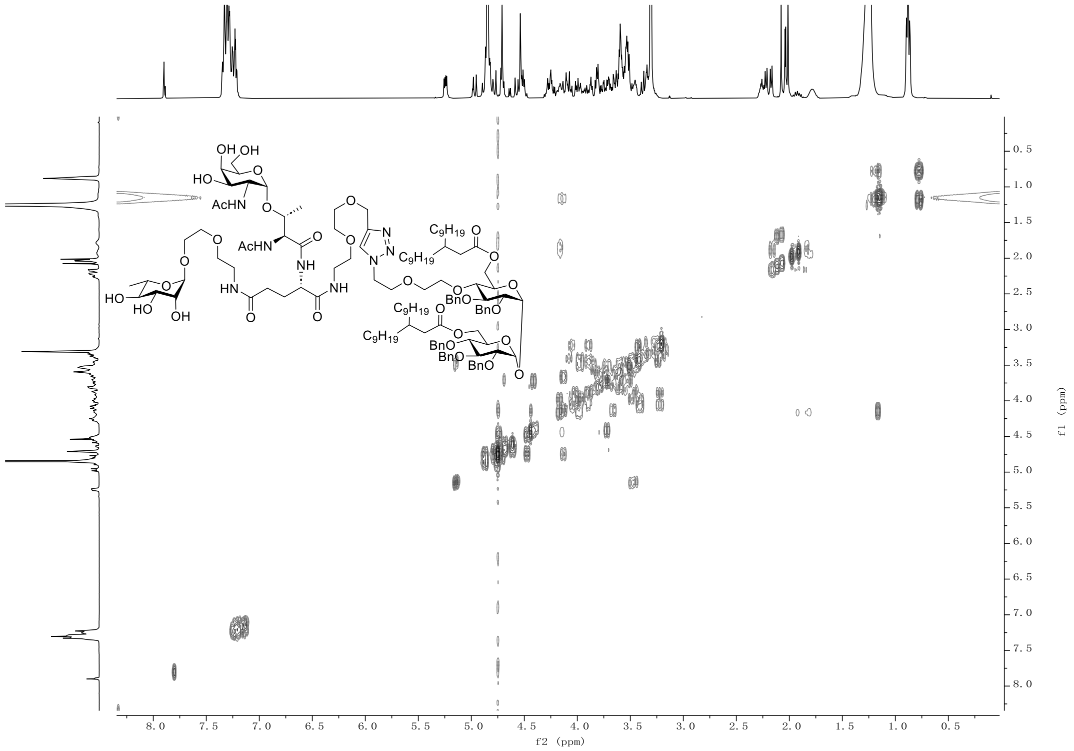

Figure S110. ^{1}H-^{1}H COSY NMR (400 MHz, $CD_3OD/CDCl_3$) spectrum of compound **27**.

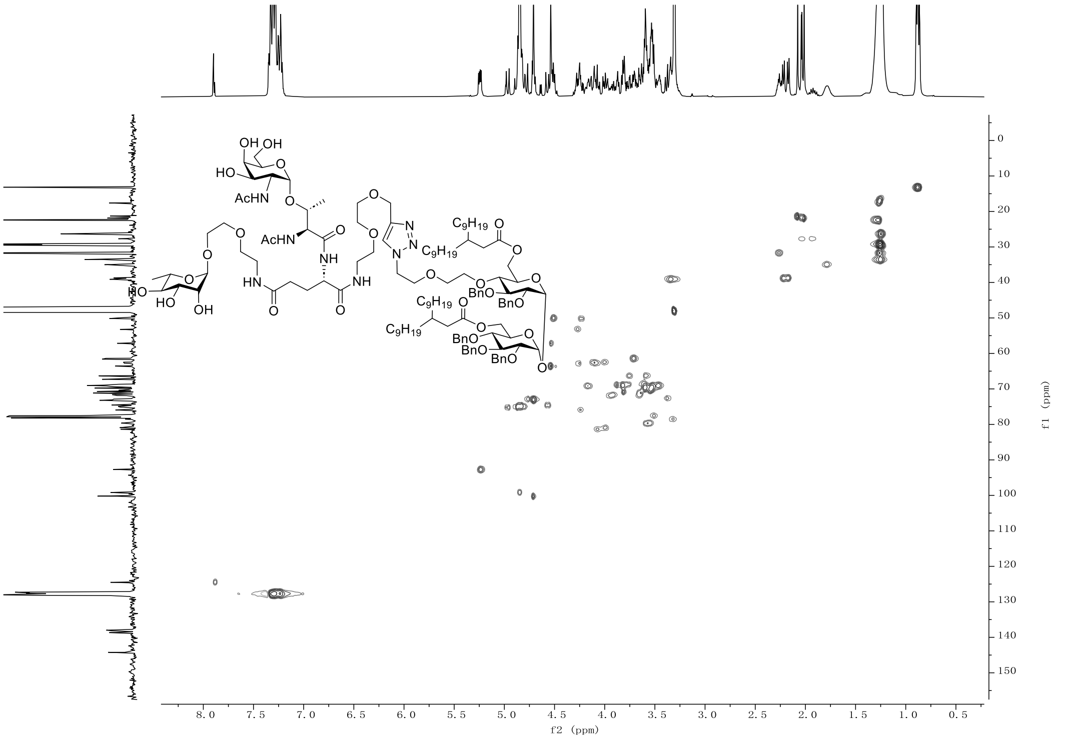

Figure S111. HSQC NMR (400/101 MHz, $CD_3OD/CDCl_3$) spectrum of compound **27**.

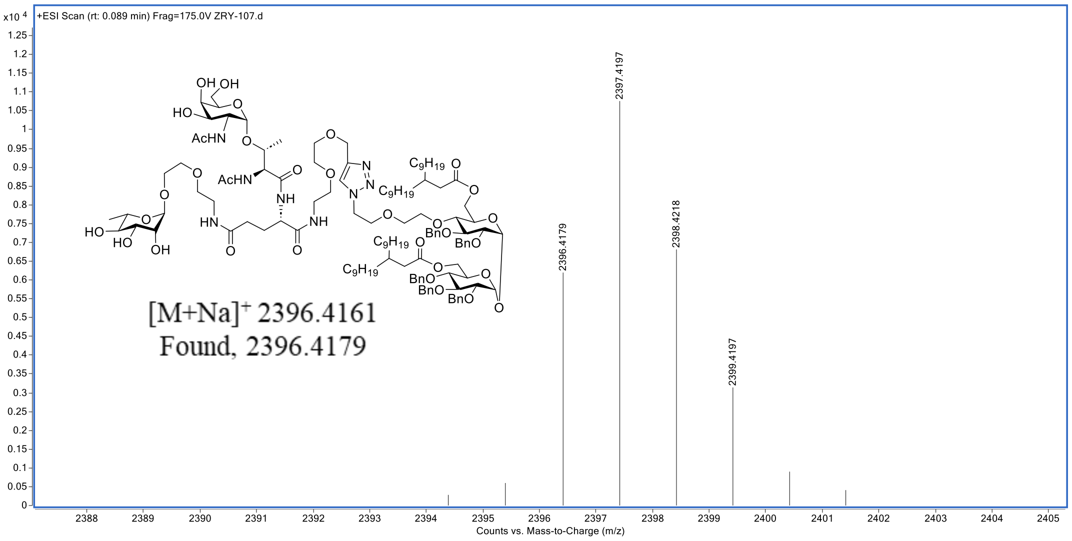

Figure S112. HRMS (ESI) spectrum of compound **27**.

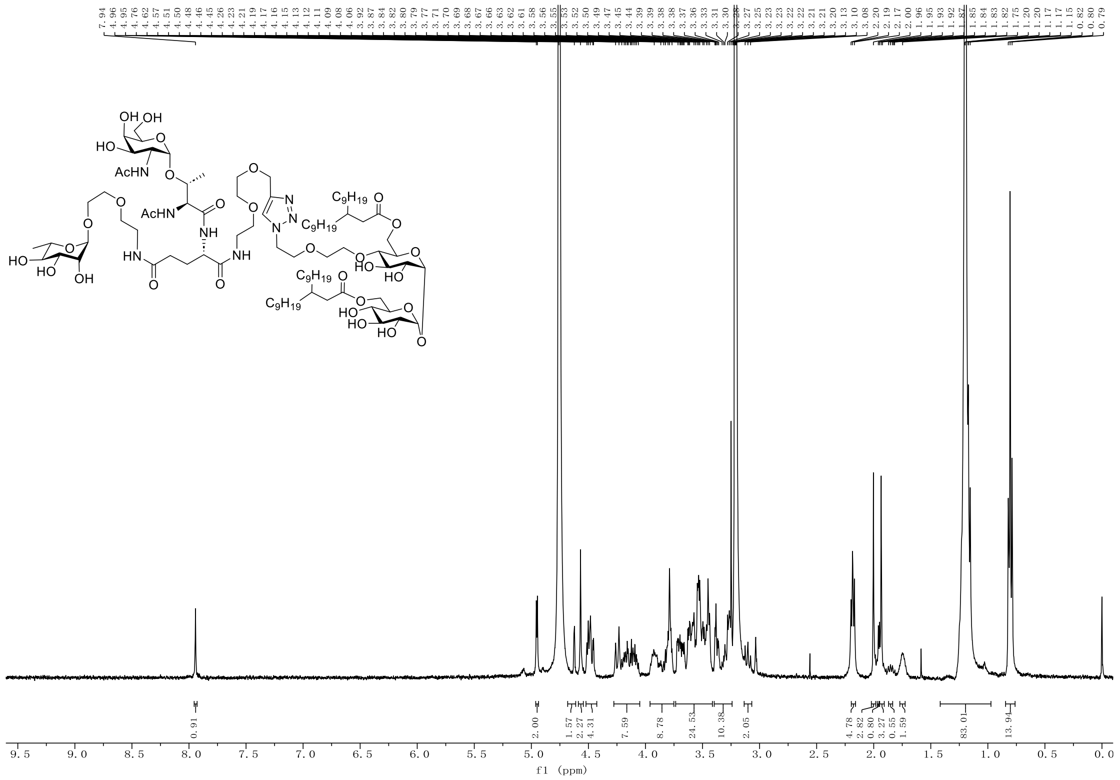

Figure S113. ^{1}H NMR (400 MHz, CD_3OD) spectrum of compound **RVT**.

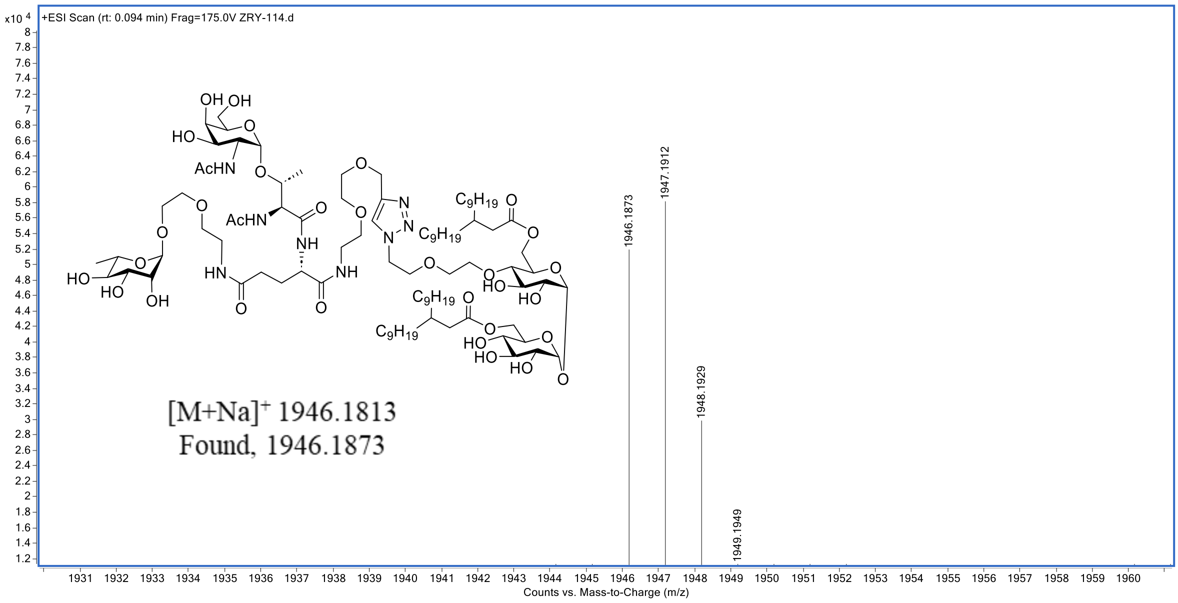

Figure S114. HRMS (ESI) spectrum of compound **RVT**.

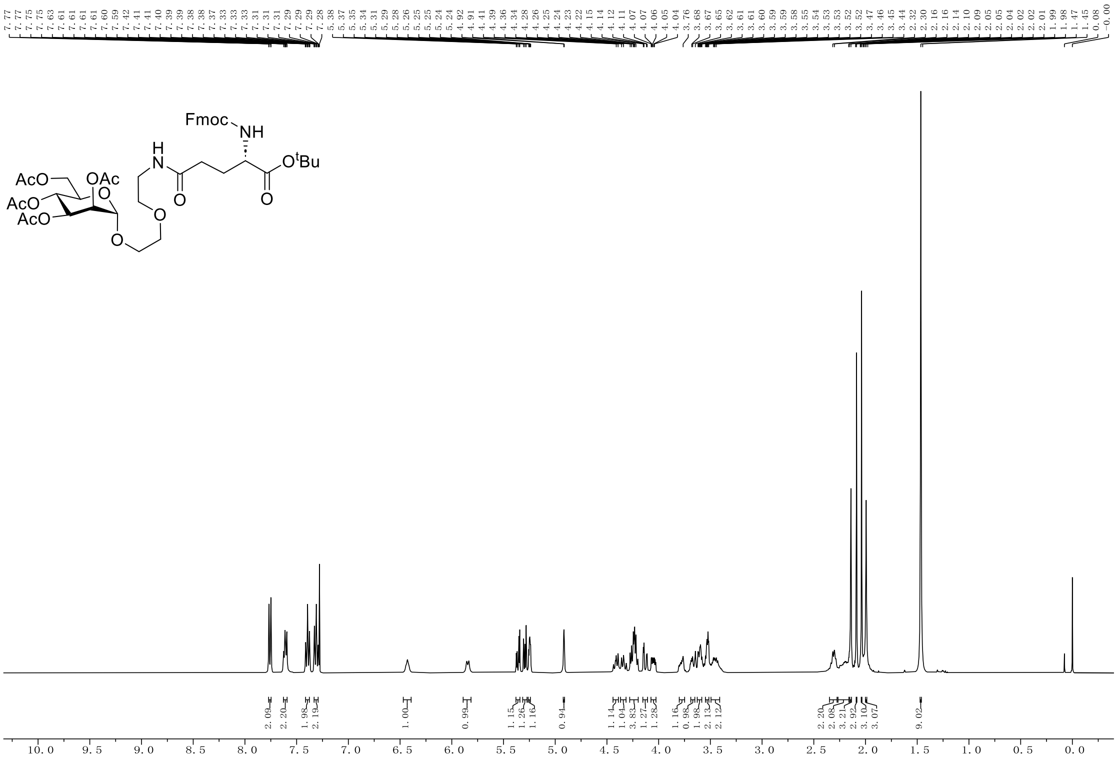

Figure S115. ^{1}H NMR (400 MHz, $CDCl_3$) spectrum of compound **28**.

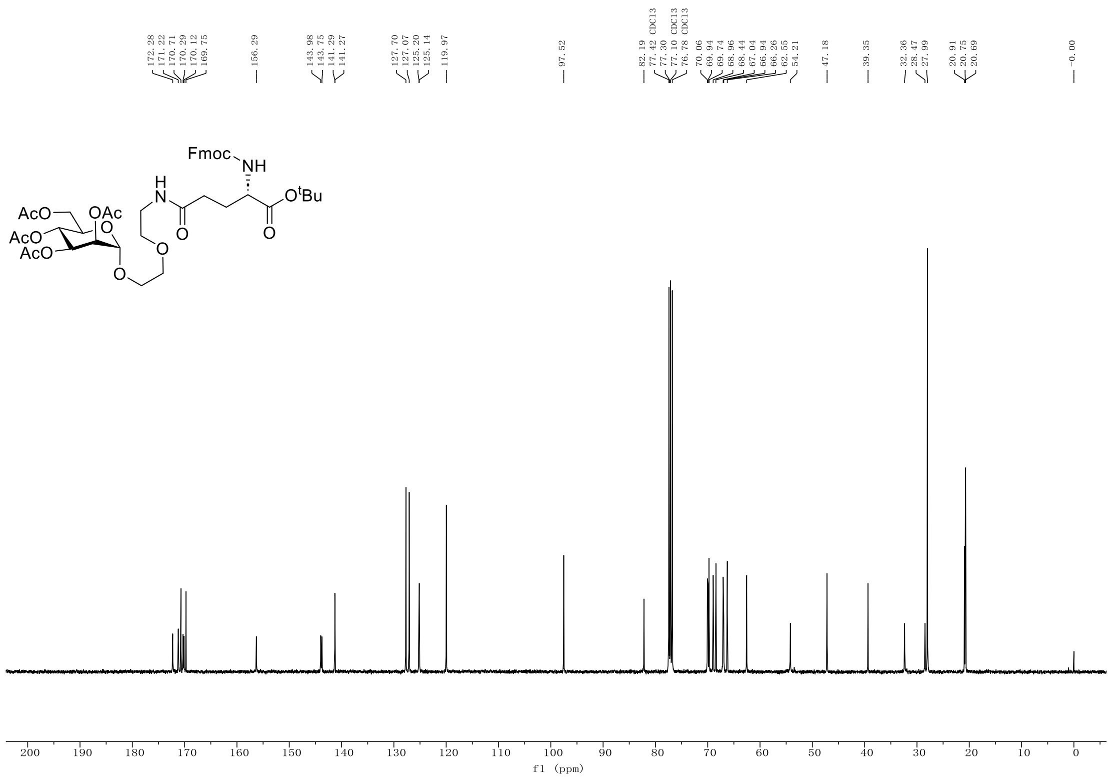

Figure S116. $^{13}C\{^{1}H\}$ NMR (101 MHz, $CDCl_3$) spectrum of compound **28**.

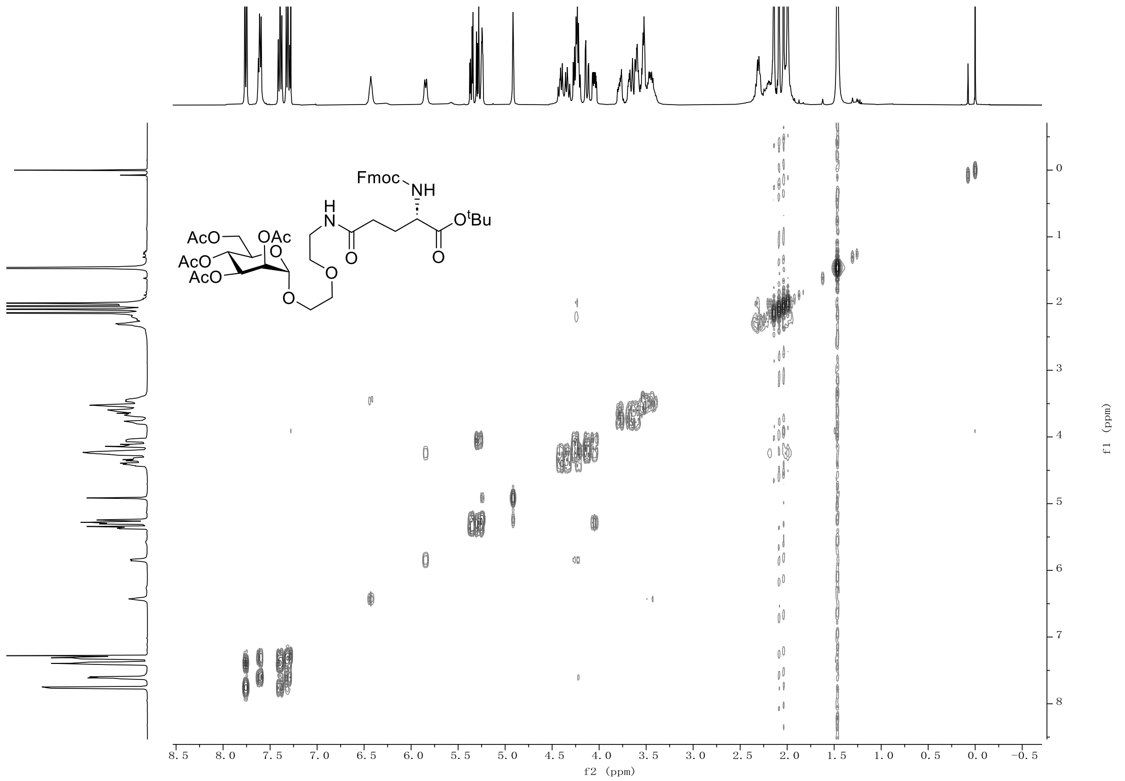

Figure S117. ^{1}H-^{1}H COSY NMR (400 MHz, $CDCl_3$) spectrum of compound **28**.

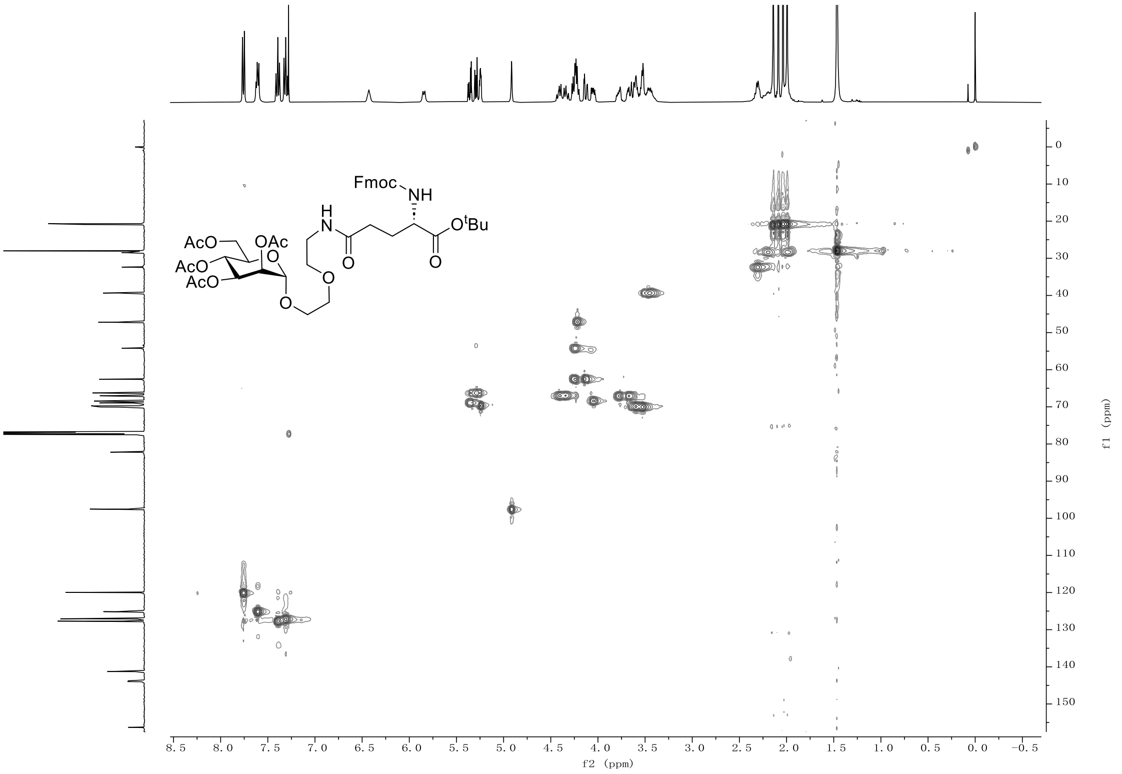

Figure S118. HSQC NMR (400/101 MHz, $CDCl_3$) spectrum of compound **28**.

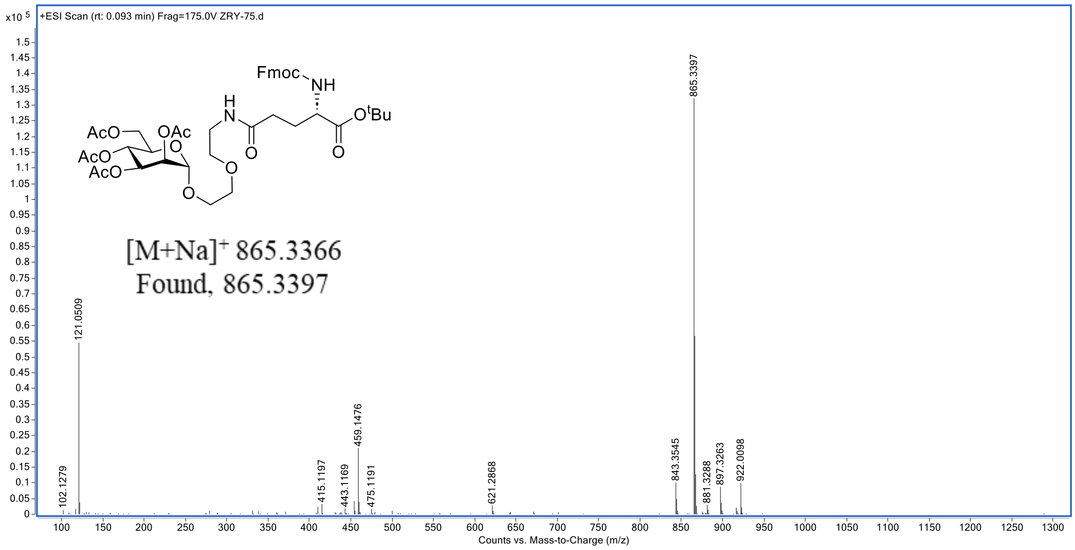

Figure S119. HRMS (ESI) spectrum of compound **28**.

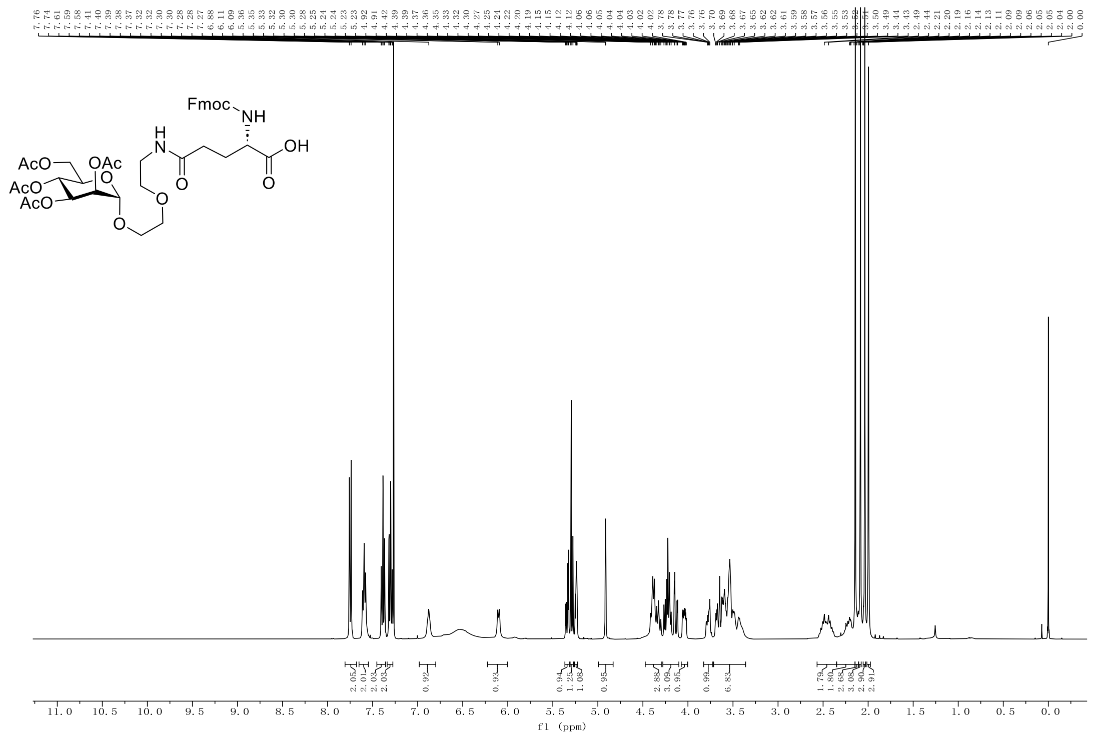

Figure S120. ^{1}H NMR (400 MHz, $CDCl_3$) spectrum of compound **29**.

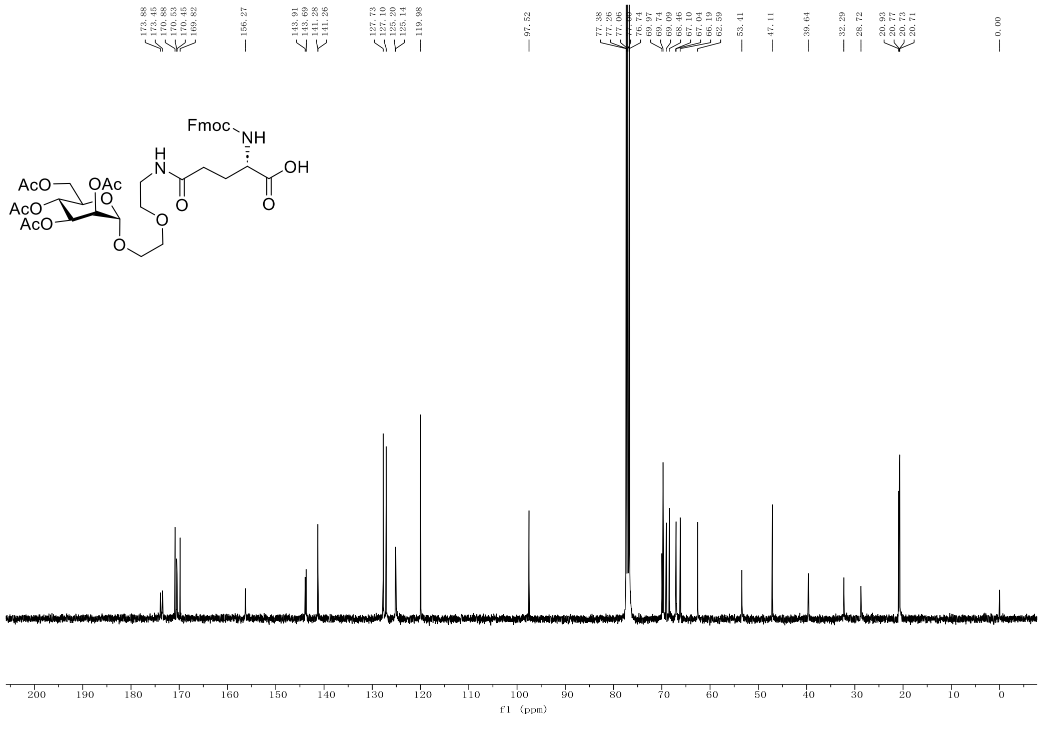

Figure S121. $^{13}C\{^{1}H\}$ NMR (101 MHz, $CDCl_3$) spectrum of compound **29**.

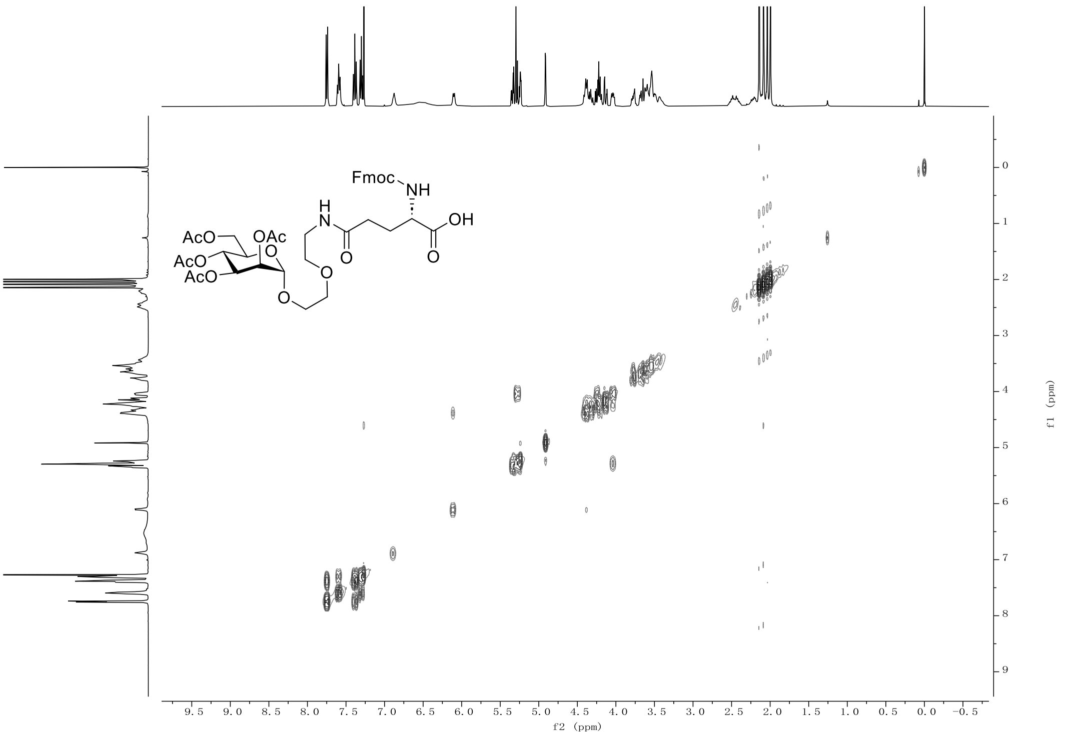

Figure S122. ^{1}H-^{1}H COSY NMR (400 MHz, $CDCl_3$) spectrum of compound **29**.

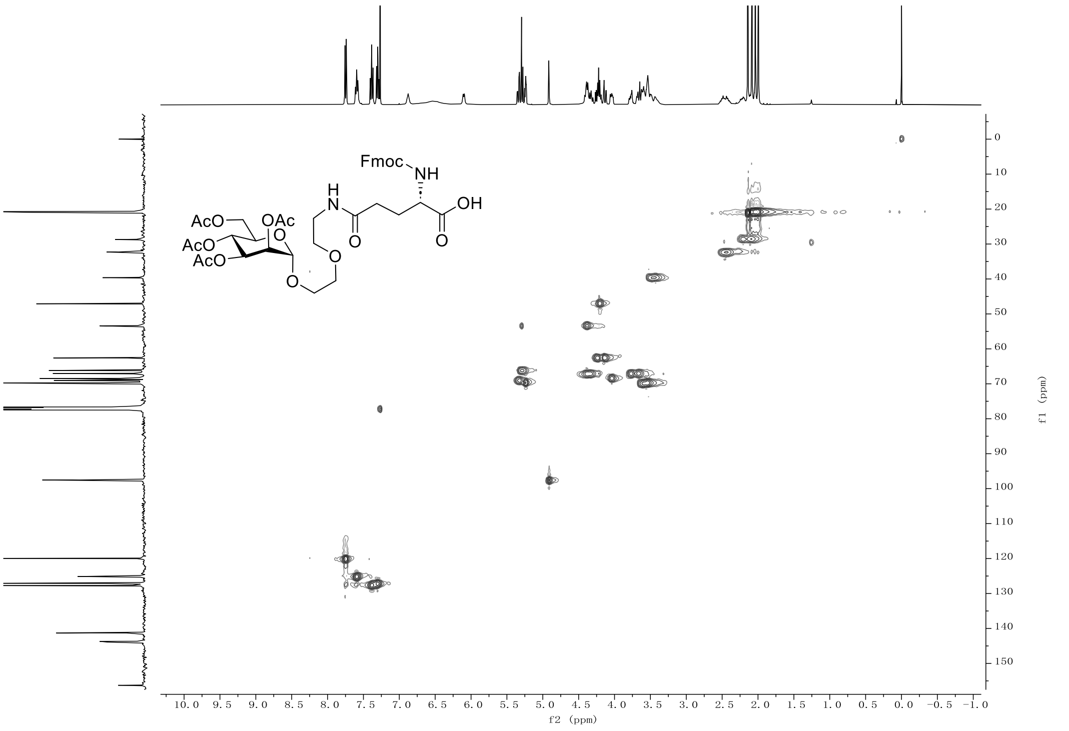

Figure S123. HSQC NMR (400/101 MHz, $CDCl_3$) spectrum of compound **29**.

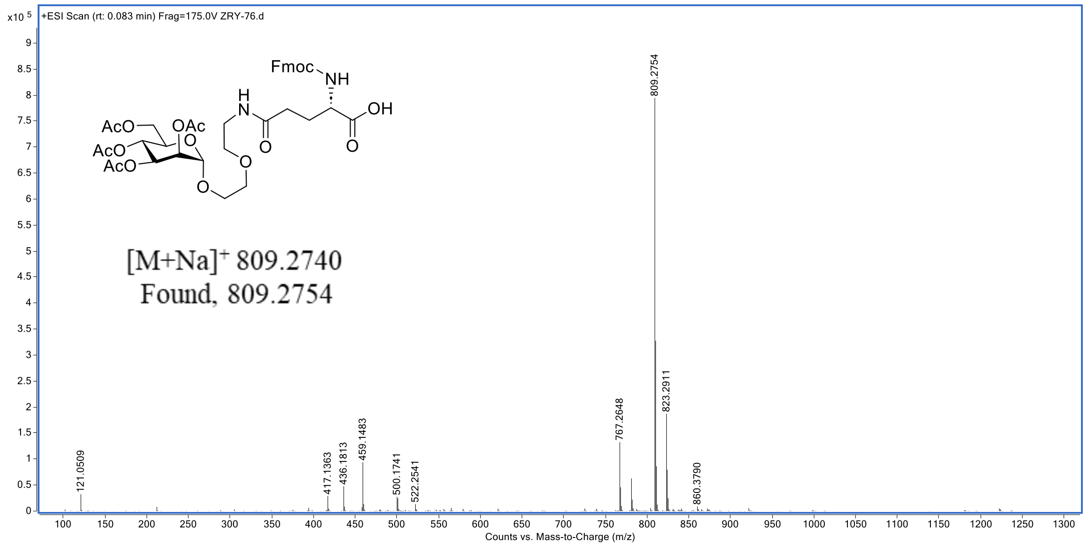

Figure S124. HRMS (ESI) spectrum of compound **29**.

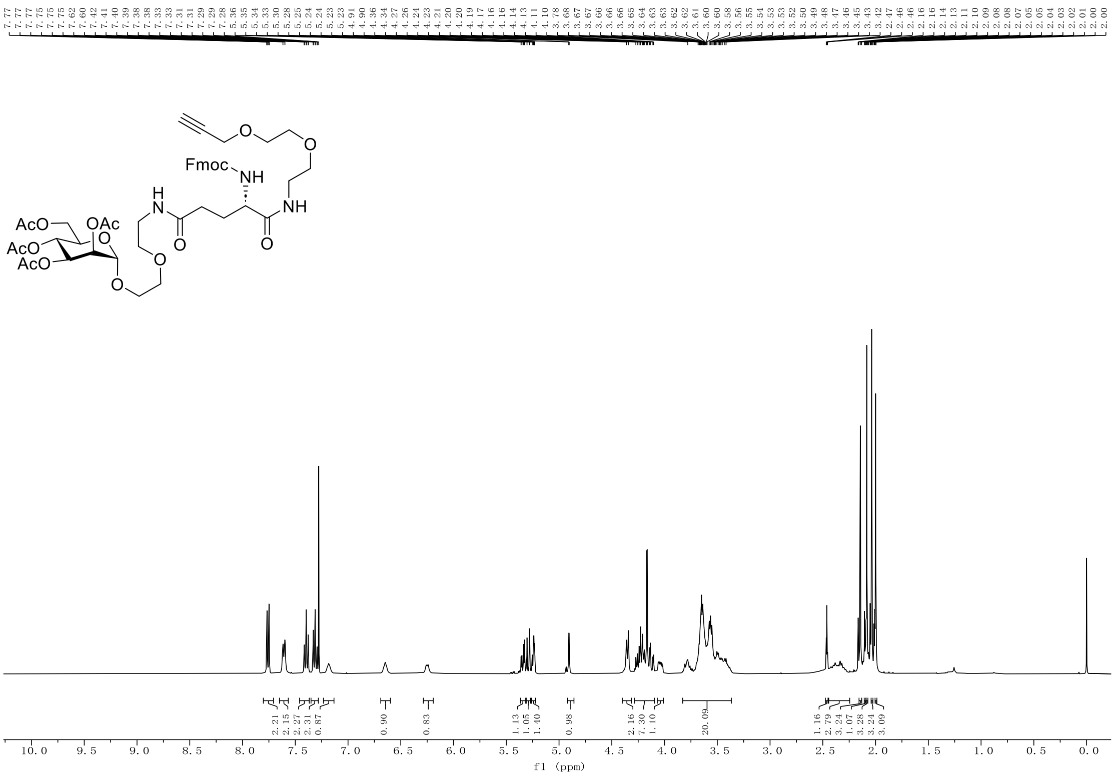

Figure S125. ^{1}H NMR (400 MHz, $CDCl_3$) spectrum of compound **30**.

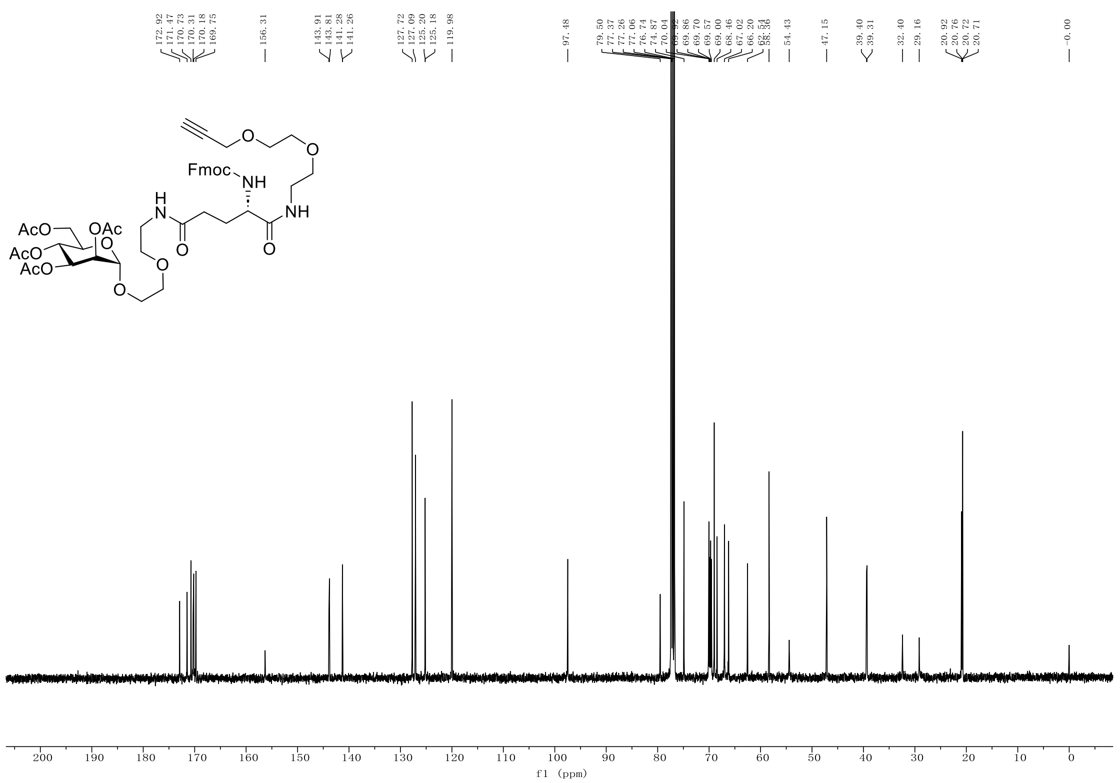

Figure S126. $^{13}C\{^{1}H\}$ NMR (101 MHz, $CDCl_3$) spectrum of compound **30**.

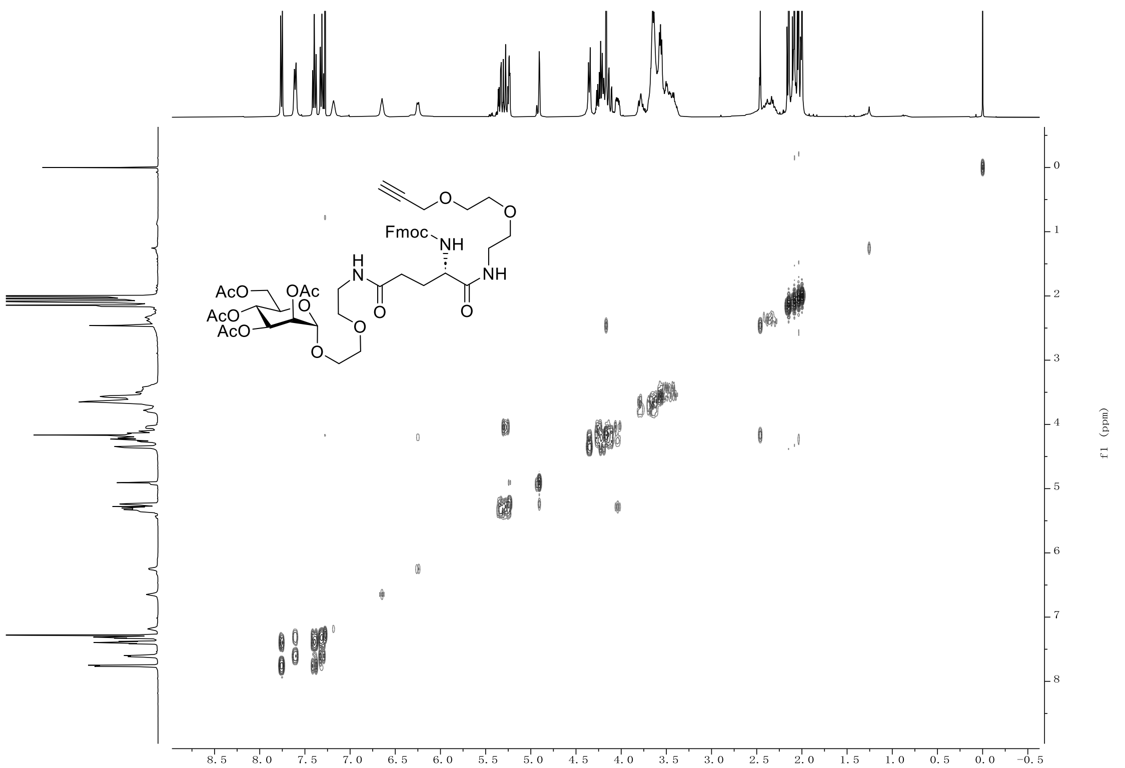

Figure S127. ^{1}H-^{1}H COSY NMR (400 MHz, $CDCl_3$) spectrum of compound **30**.

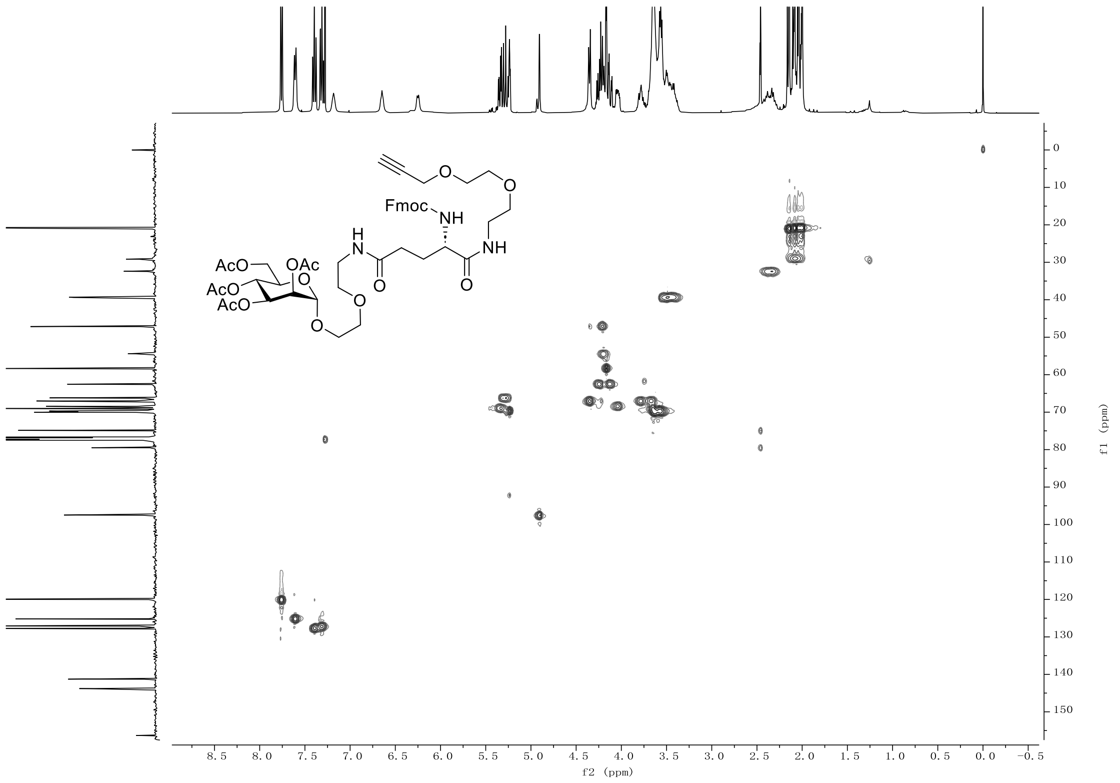

Figure S128. HSQC NMR (400/101 MHz, $CDCl_3$) spectrum of compound **30**.

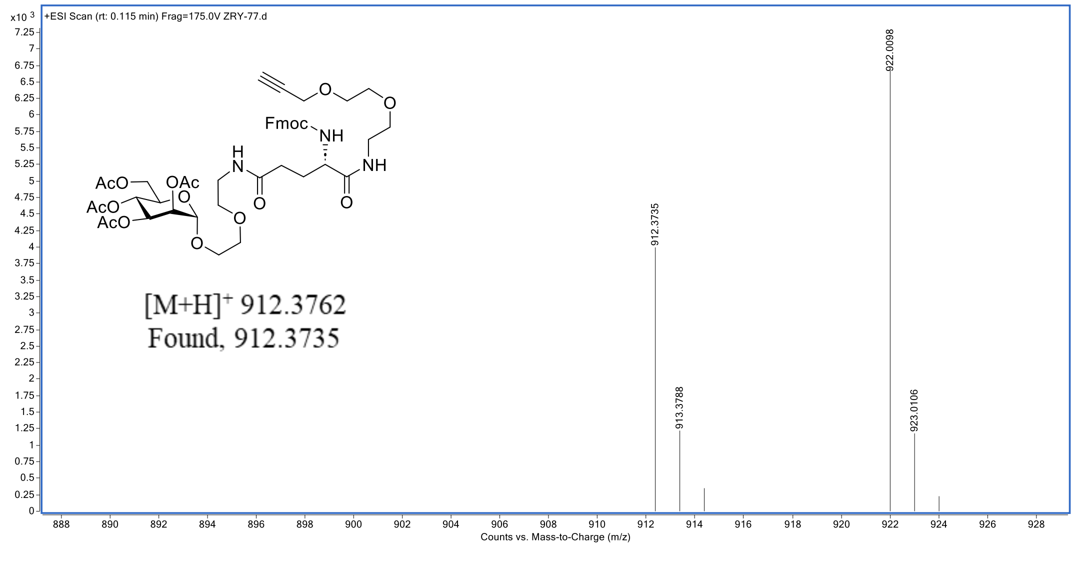

Figure S129. HRMS (ESI) spectrum of compound **30**.

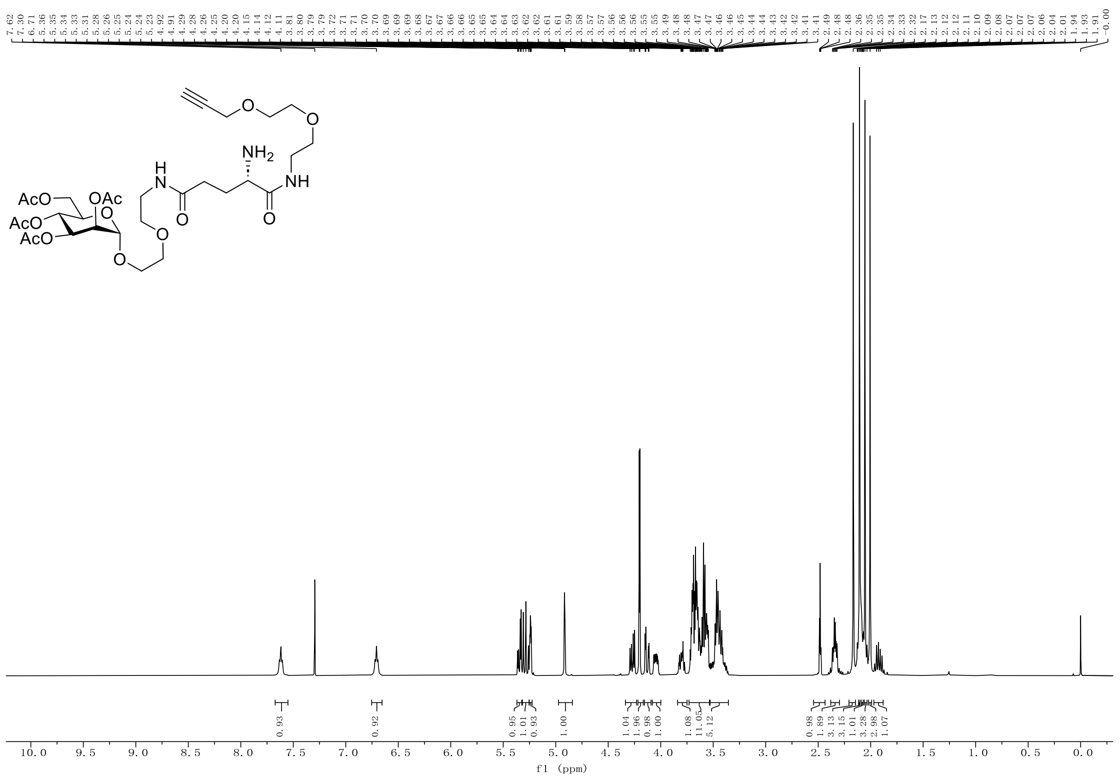

Figure S130. ^{1}H NMR (400 MHz, $CDCl_3$) spectrum of compound **31**.

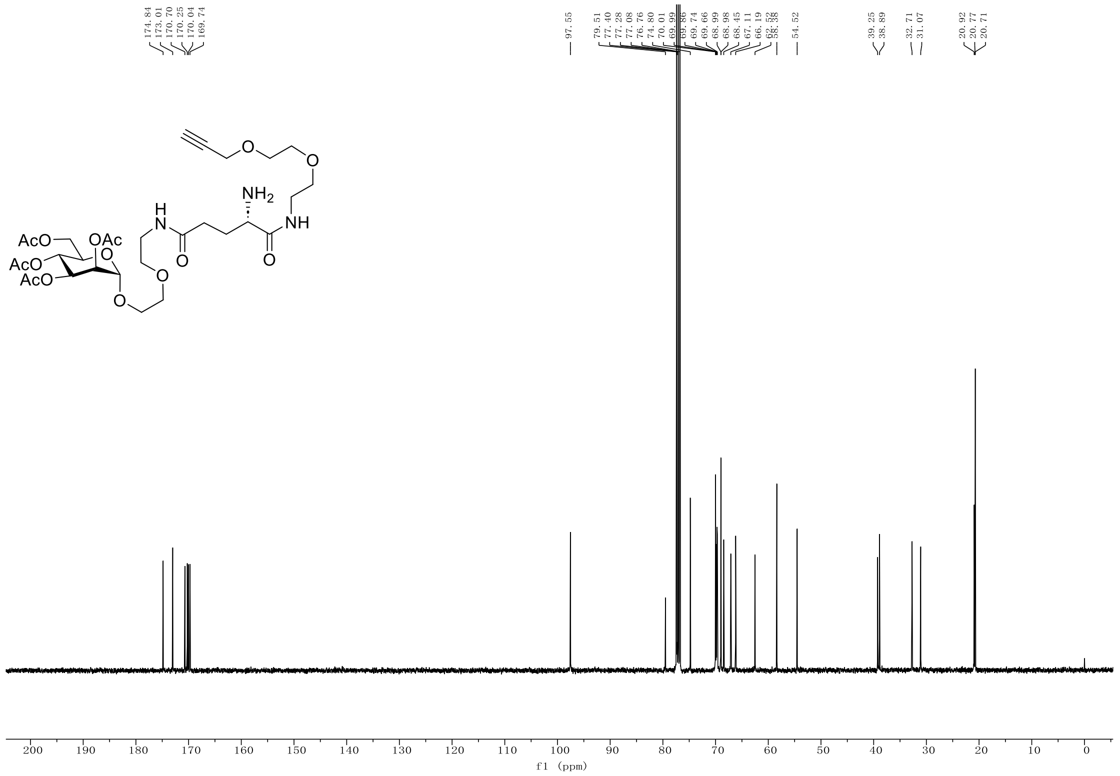

Figure S131. $^{13}C\{^{1}H\}$ NMR (101 MHz, $CDCl_3$) spectrum of compound **31**.

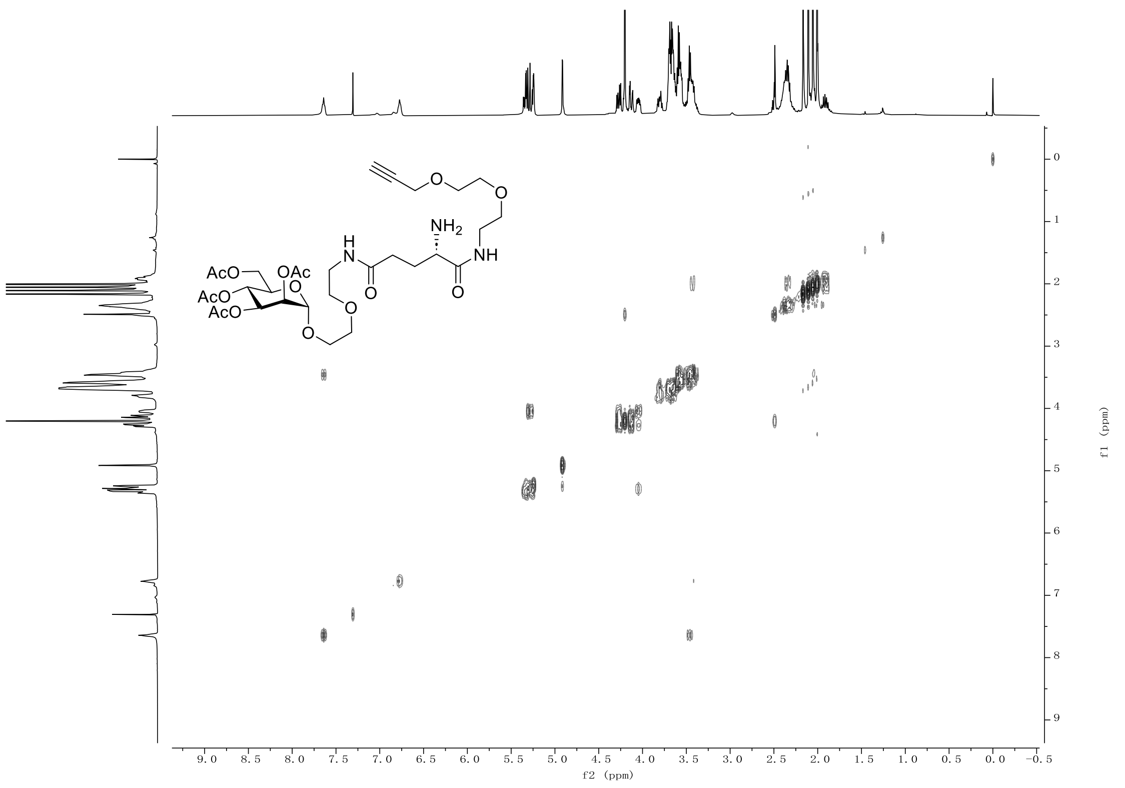

Figure S132. ^1H-^1H COSY NMR (400 MHz, $CDCl_3$) spectrum of compound **31**.

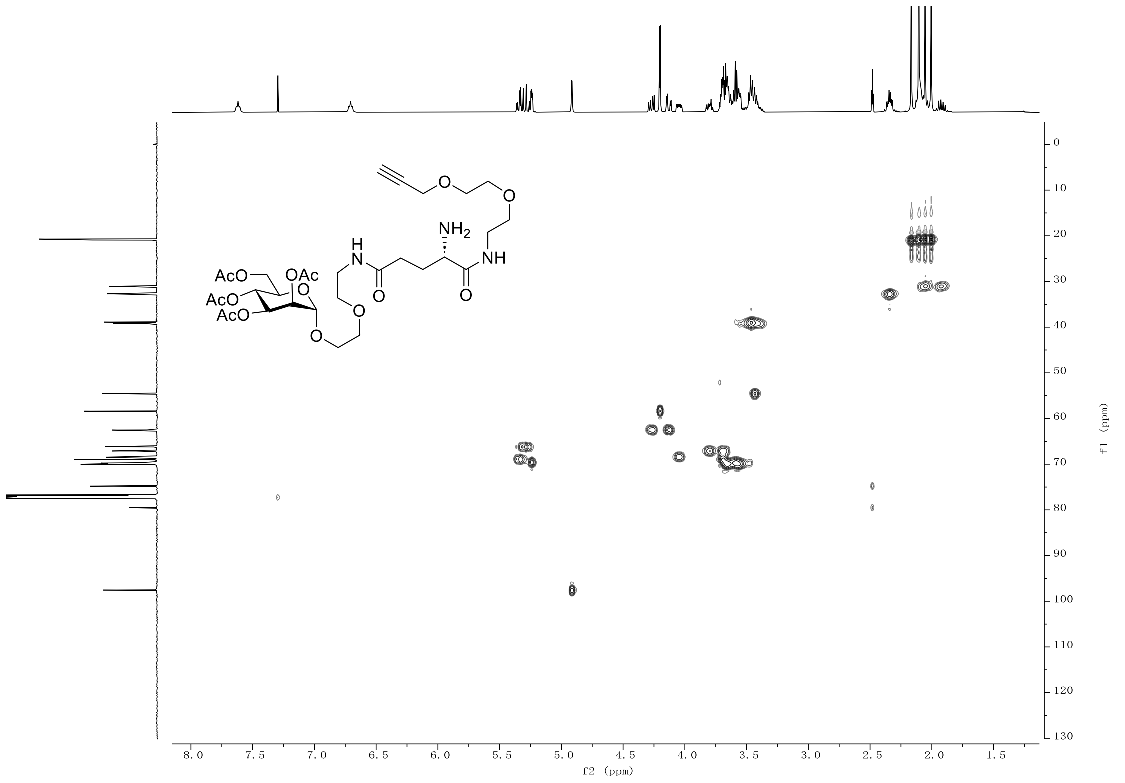

Figure S133. HSQC NMR (400/101 MHz, $CDCl_3$) spectrum of compound **31**.

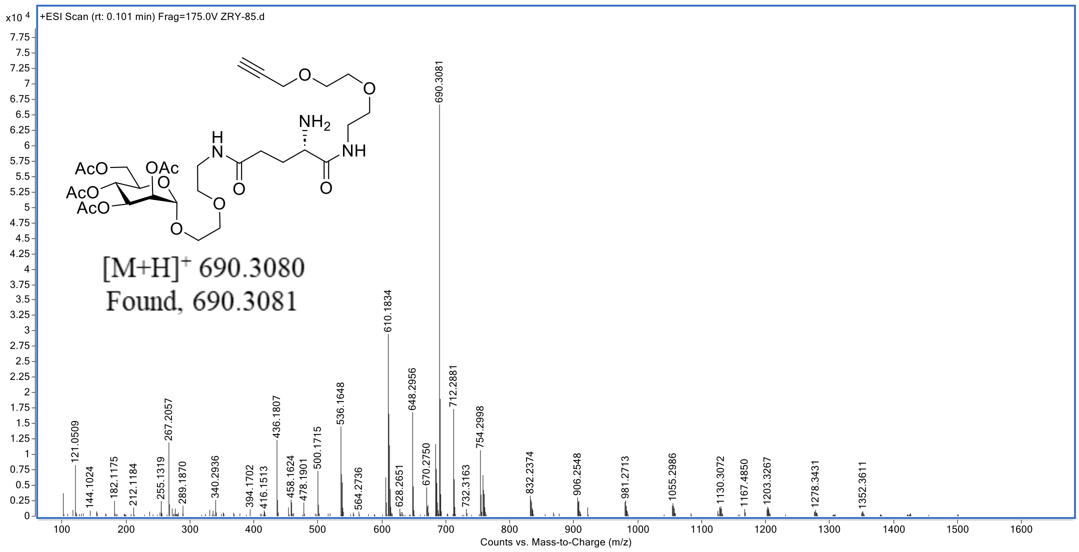

Figure S134. HRMS (ESI) spectrum of compound **31**.

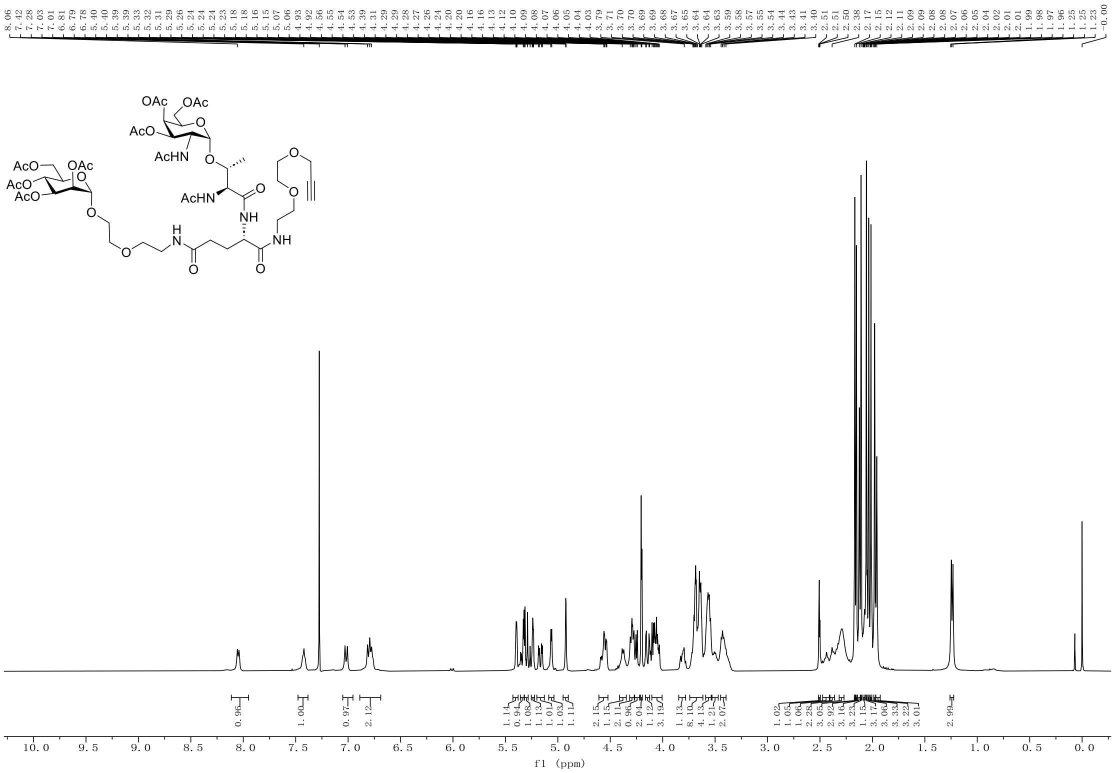

Figure S135. ^{1}H NMR (400 MHz, $CDCl_3$) spectrum of compound **32**.

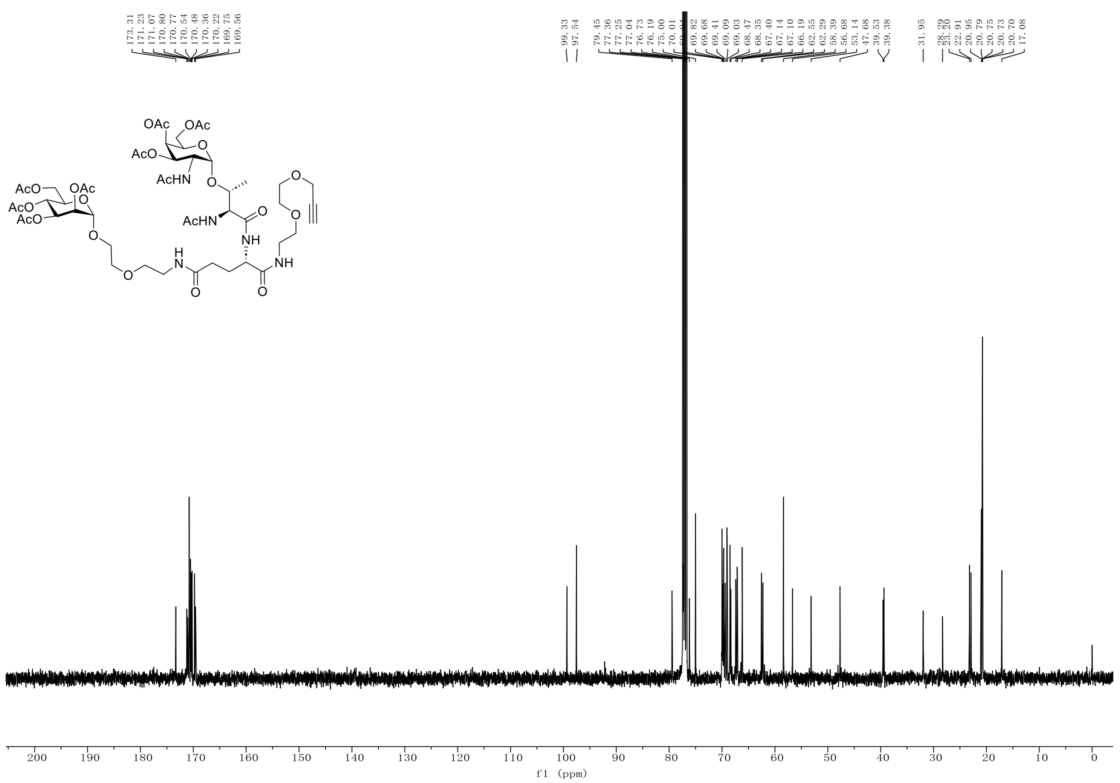

Figure S136. $^{13}C\{^{1}H\}$ NMR (101 MHz, $CDCl_3$) spectrum of compound **32**.

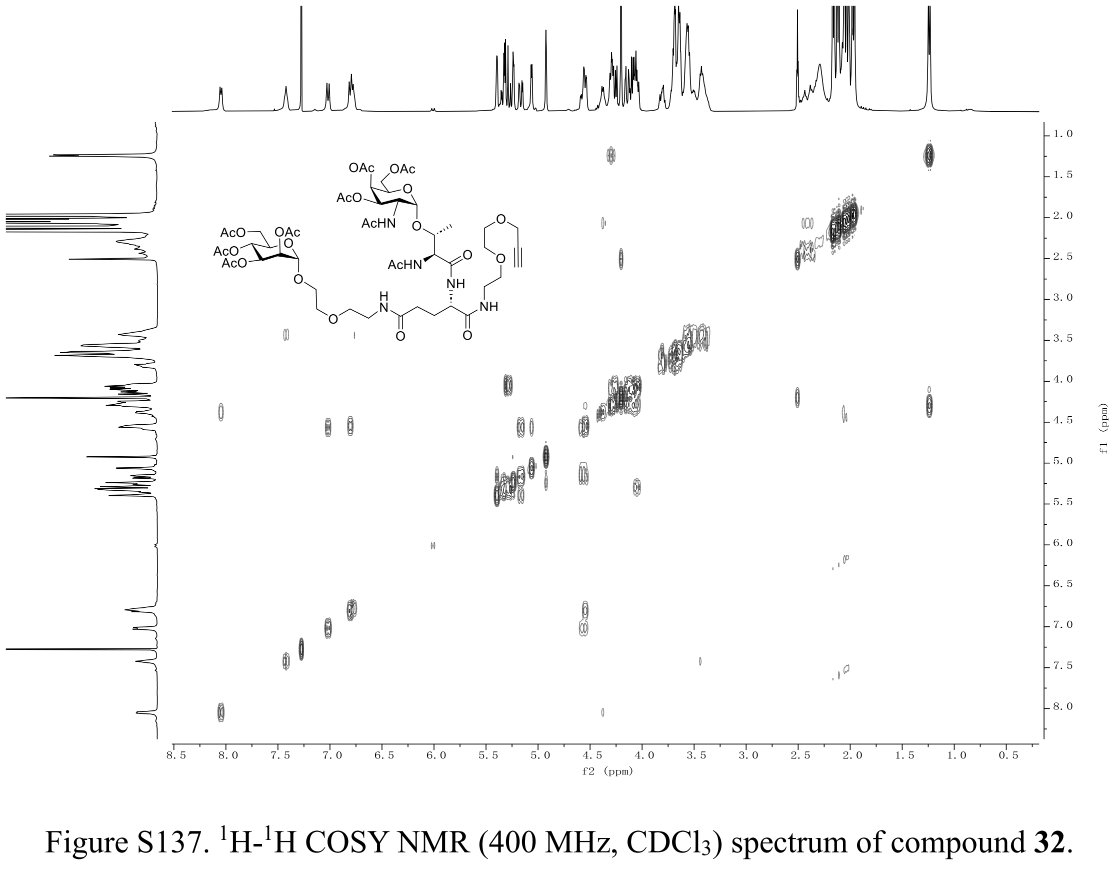

Figure S137. ^{1}H-^{1}H COSY NMR (400 MHz, $CDCl_3$) spectrum of compound **32**.

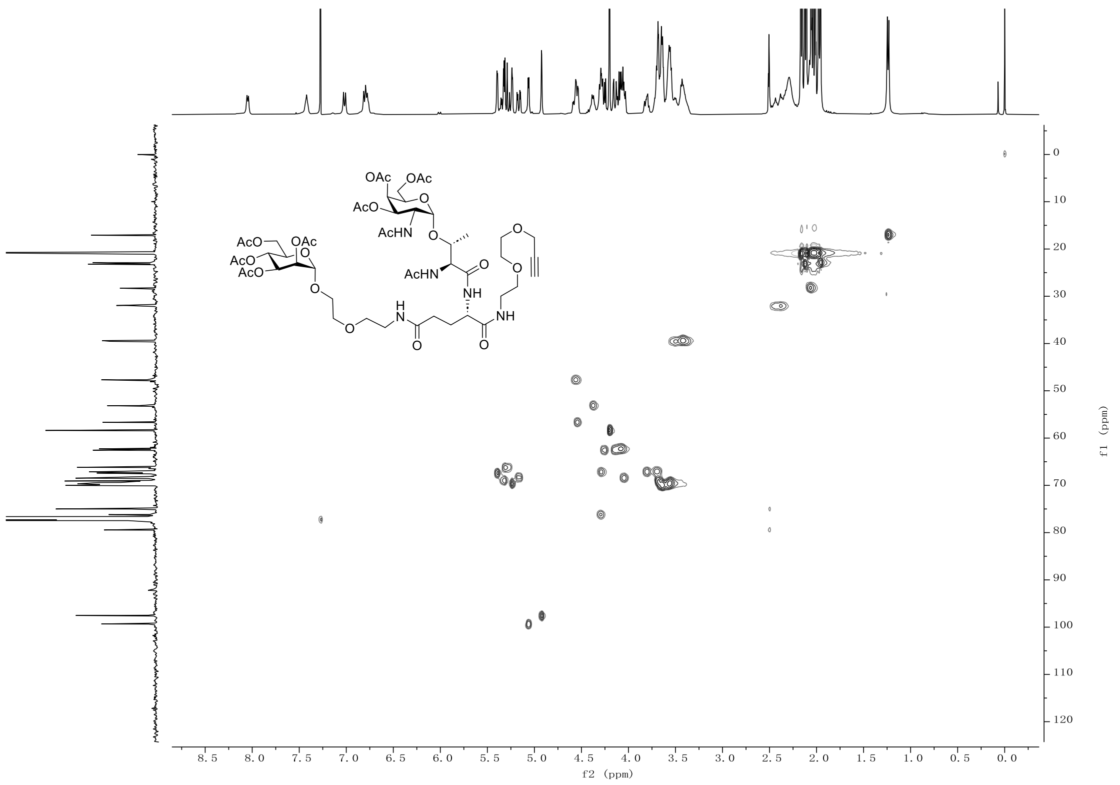

Figure S138. HSQC NMR (400/101 MHz, $CDCl_3$) spectrum of compound **32**.

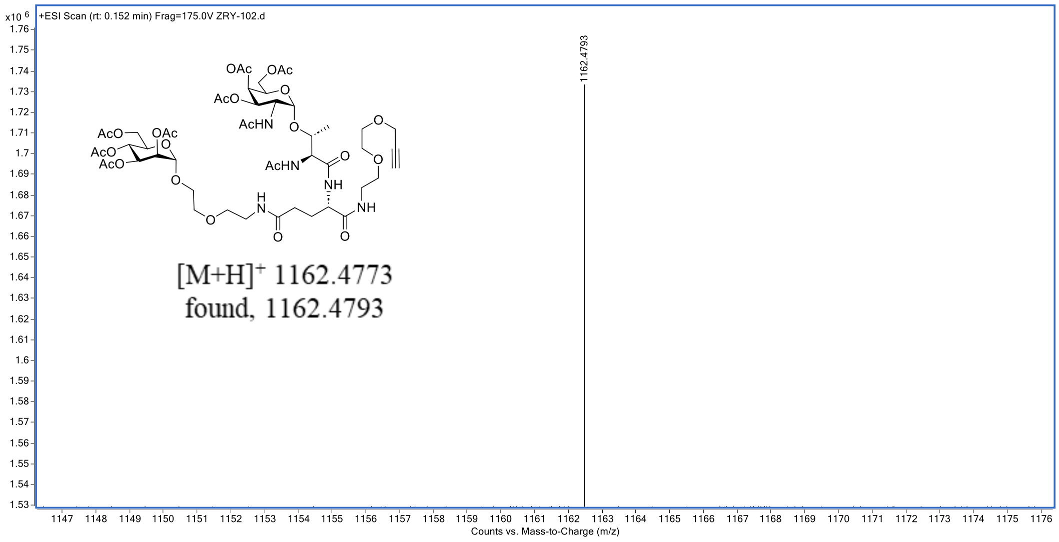

Figure S139. HRMS (ESI) spectrum of compound **32**.

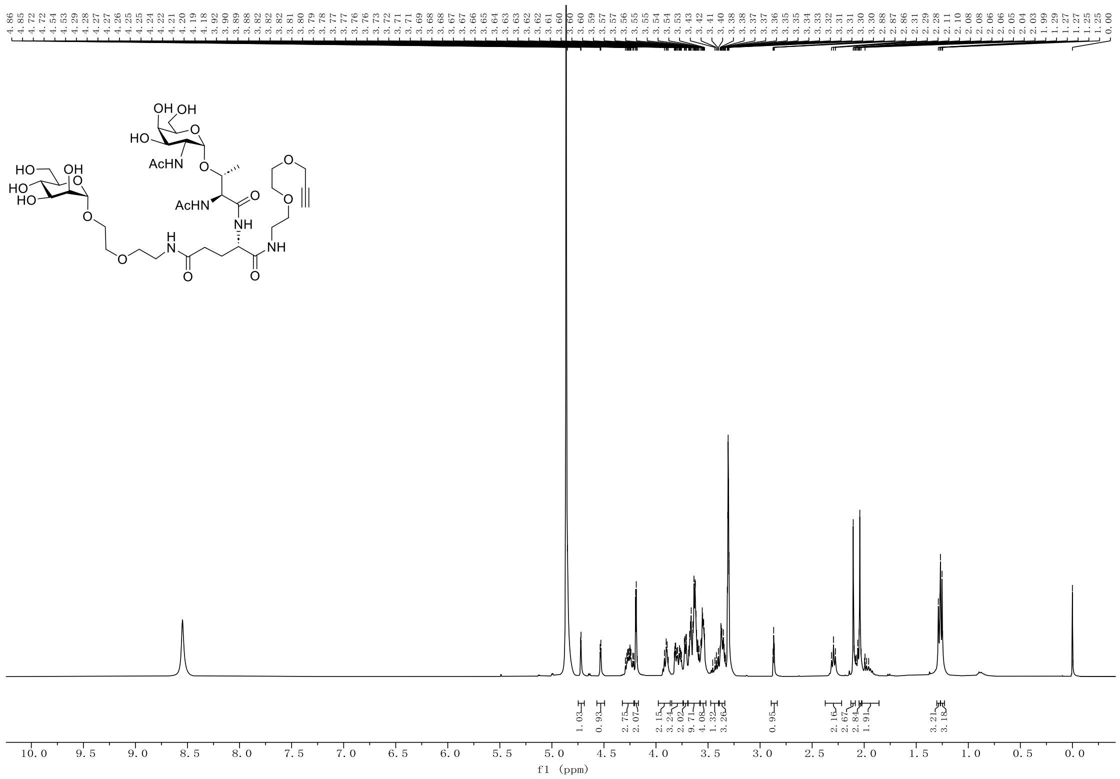

Figure S140. ^{1}H NMR (400 MHz, CD_3OD) spectrum of Compound **33**.

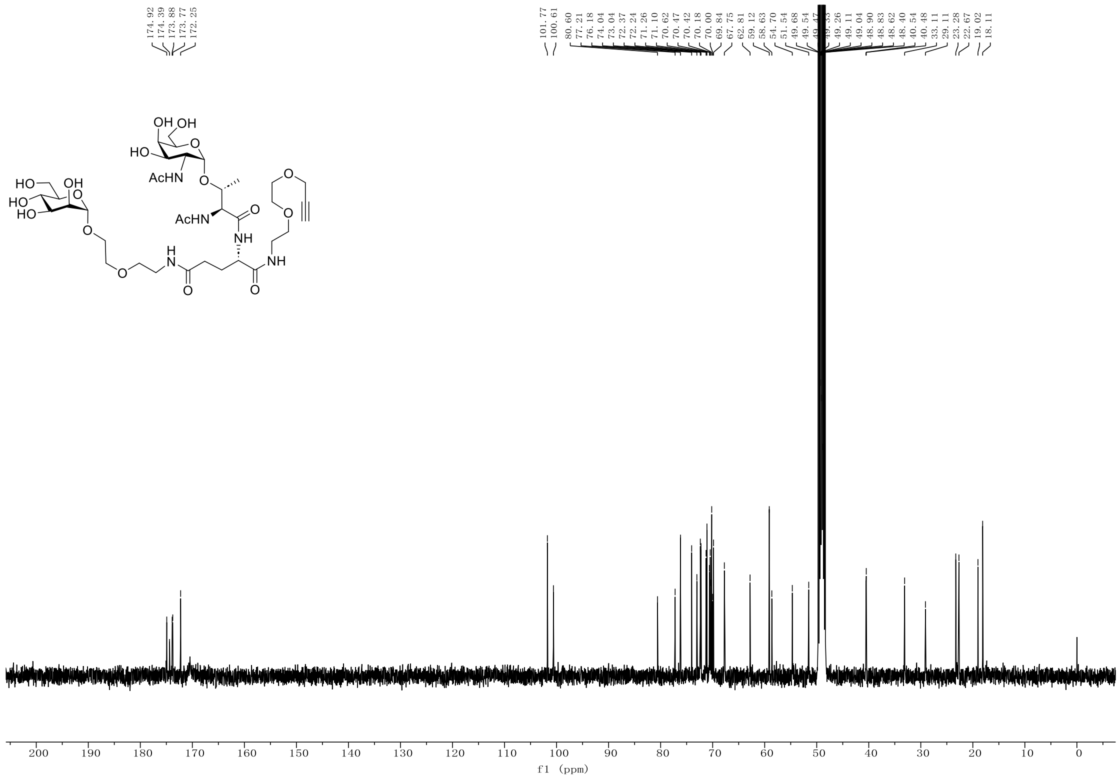

Figure S141. $^{13}C\{^{1}H\}$ NMR (100 MHz, CD_3OD) spectrum of compound **33**.

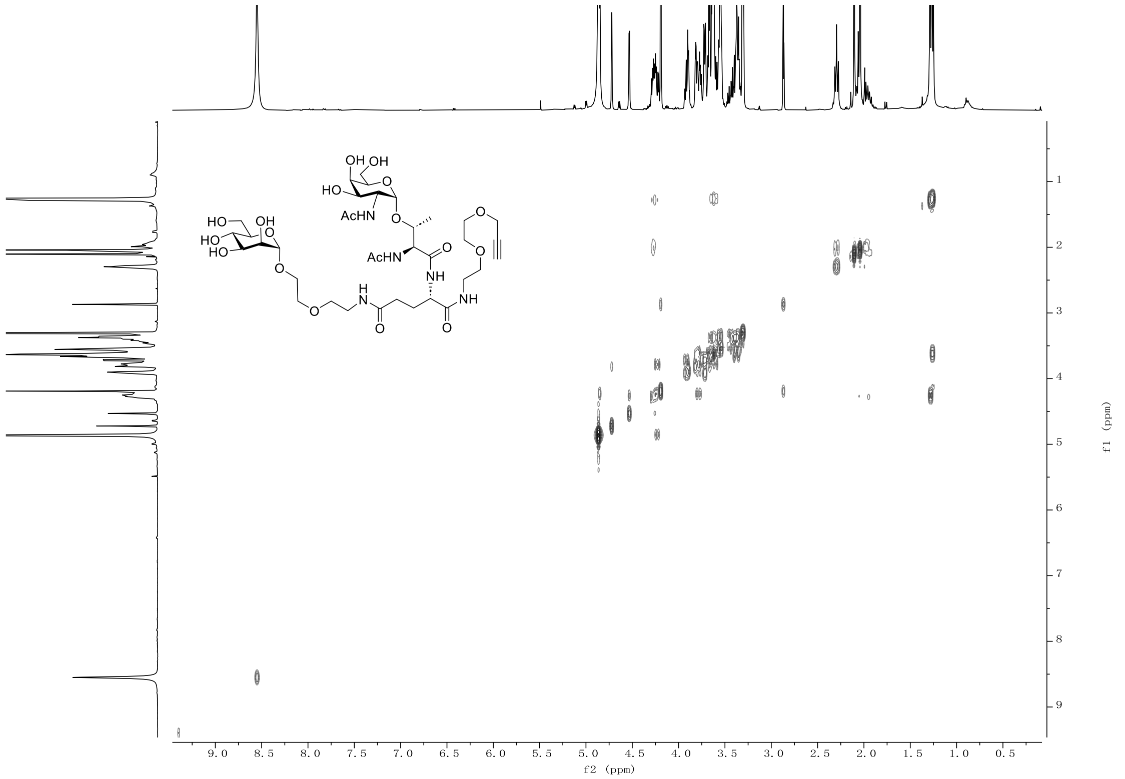

Figure S142. ^{1}H-^{1}H COSY NMR (400 MHz, CD_3OD) spectrum of compound **33**.

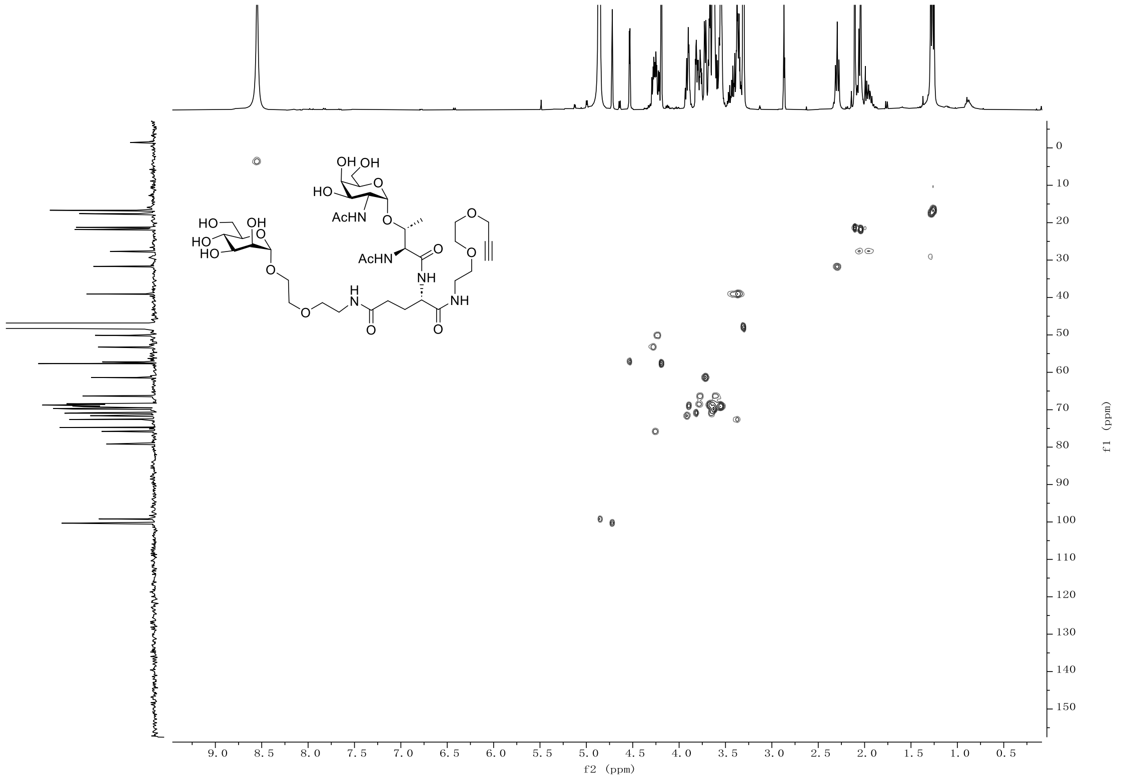

Figure S143. HSQC NMR (400/101 MHz, CD_3OD) spectrum of compound **33**.

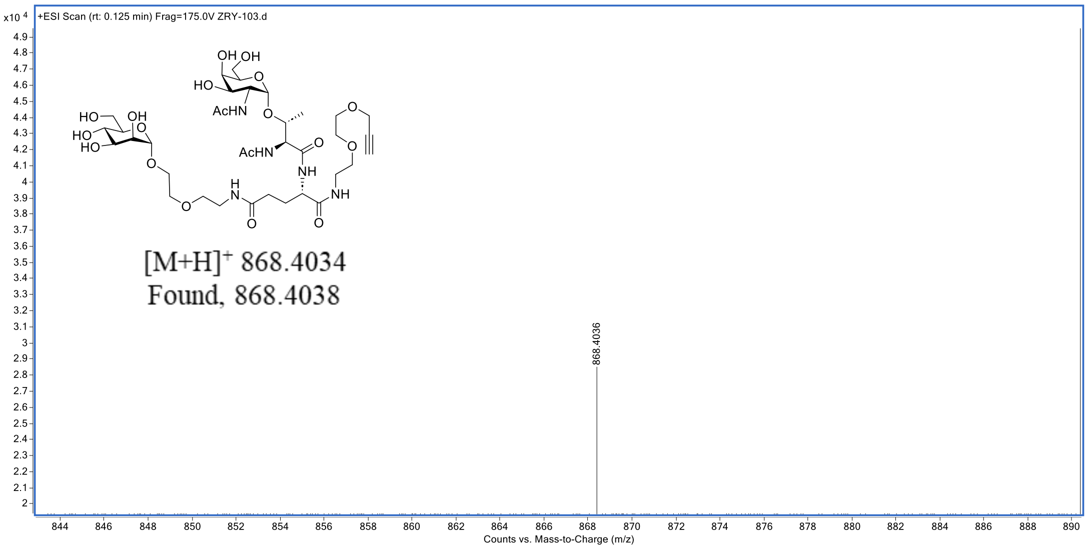

Figure S144. HRMS (ESI) spectrum of compound **33**.

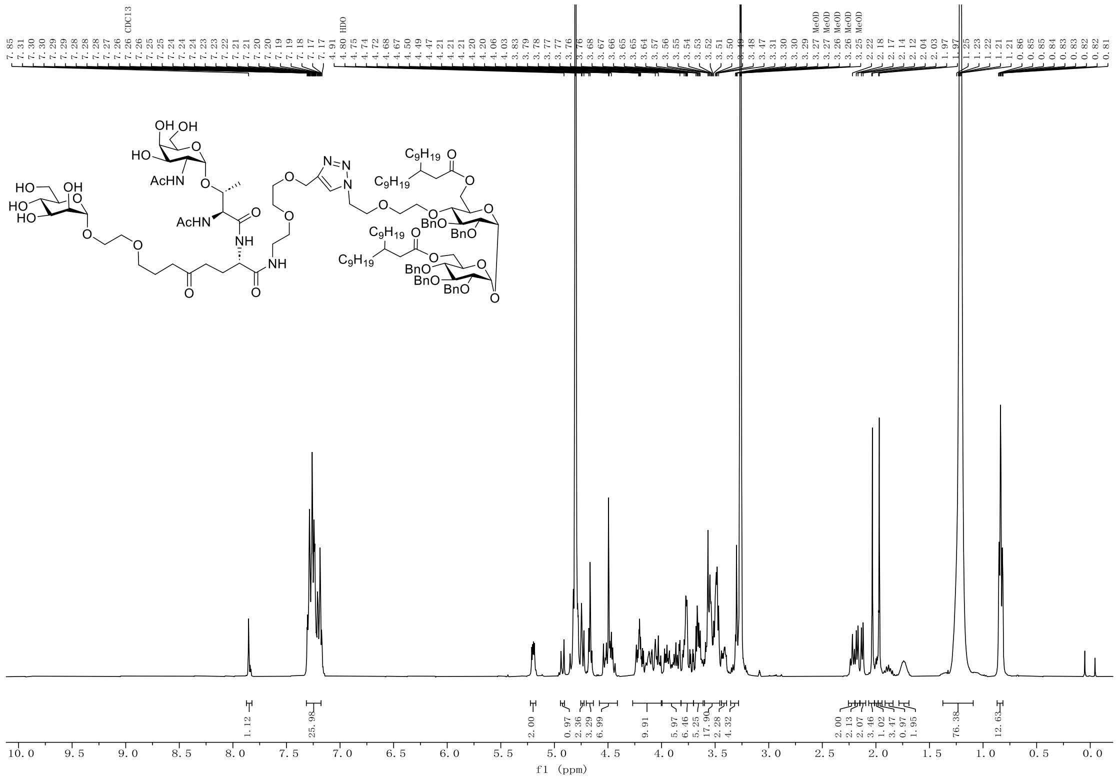

Figure S145. ^{1}H NMR (400 MHz, $CD_3OD/CDCl_3$) spectrum of compound **34**.

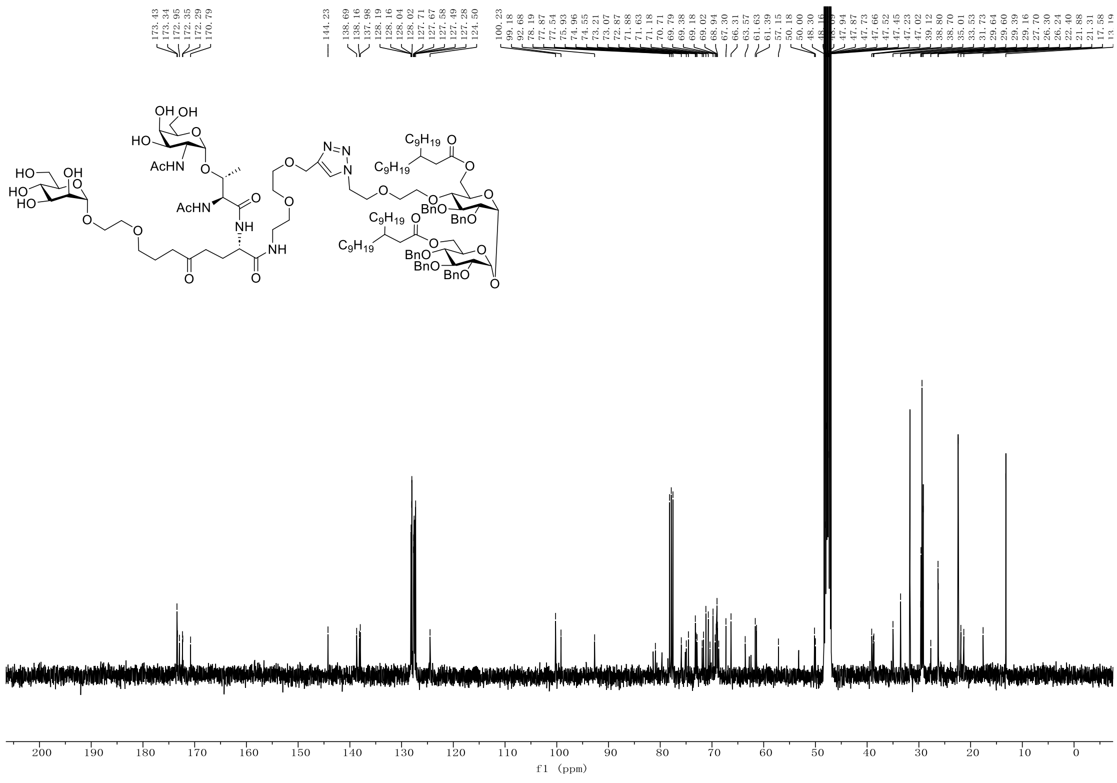

Figure S146. $^{13}C\{^{1}H\}$ NMR (101 MHz, $CD_3OD/CDCl_3$) spectrum of compound **34**.

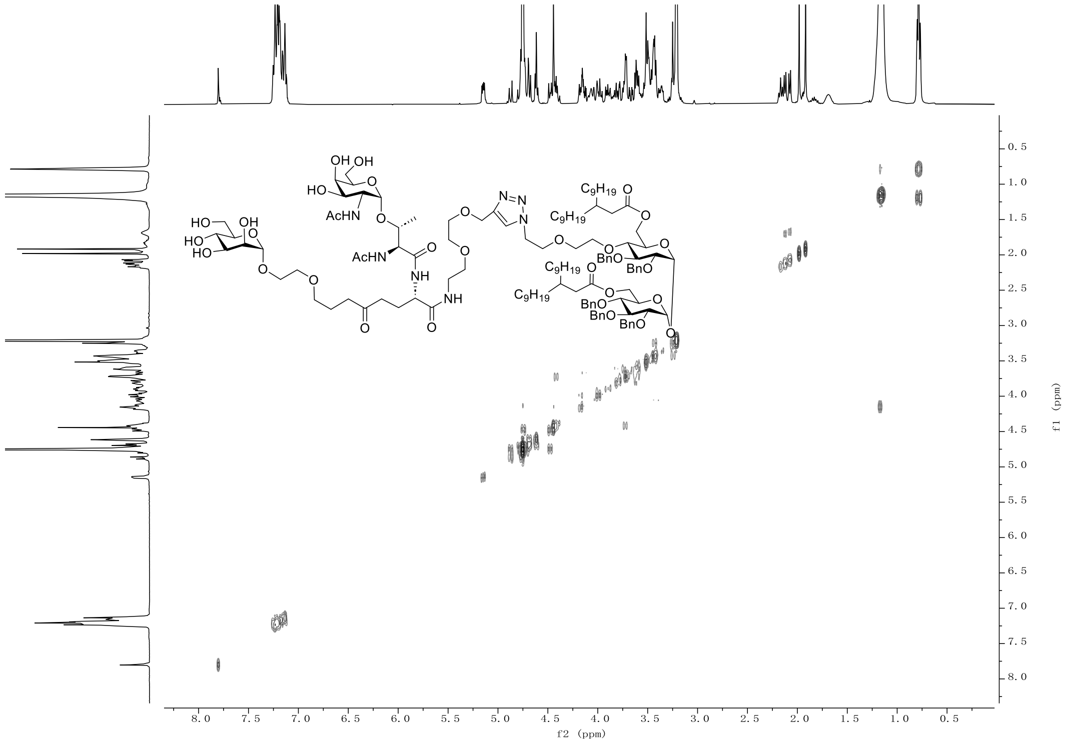

Figure S147. ^{1}H-^{1}H COSY NMR (400 MHz, $CD_3OD/CDCl_3$) spectrum of compound **34**.

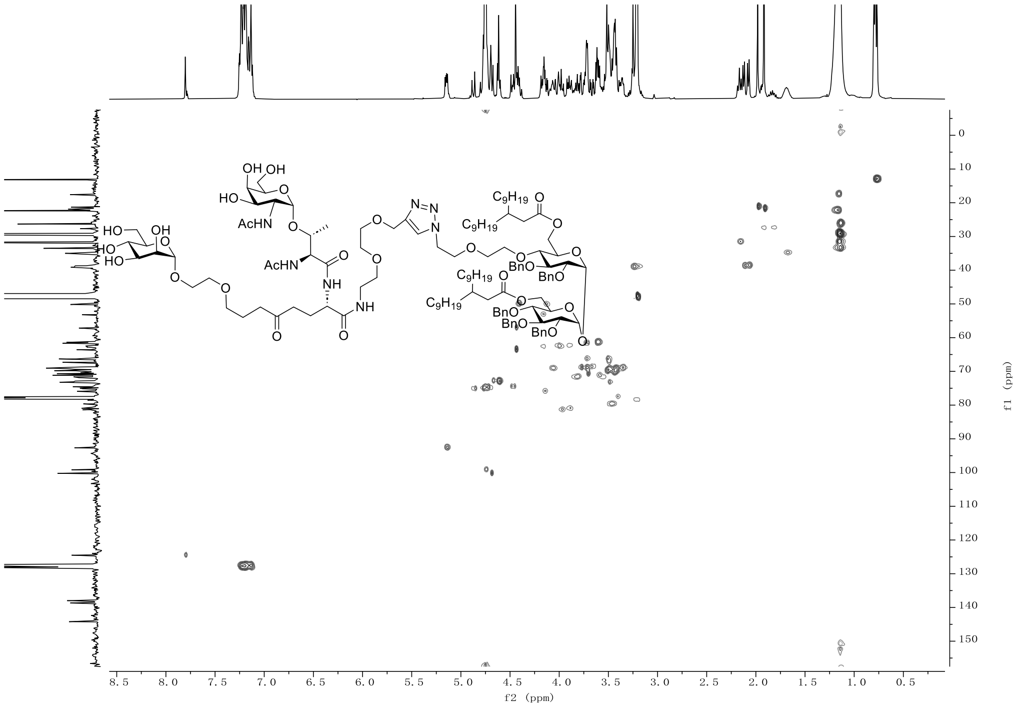

Figure S148. HSQC NMR (400/101 MHz, $CD_3OD/CDCl_3$) spectrum of compound **34**.

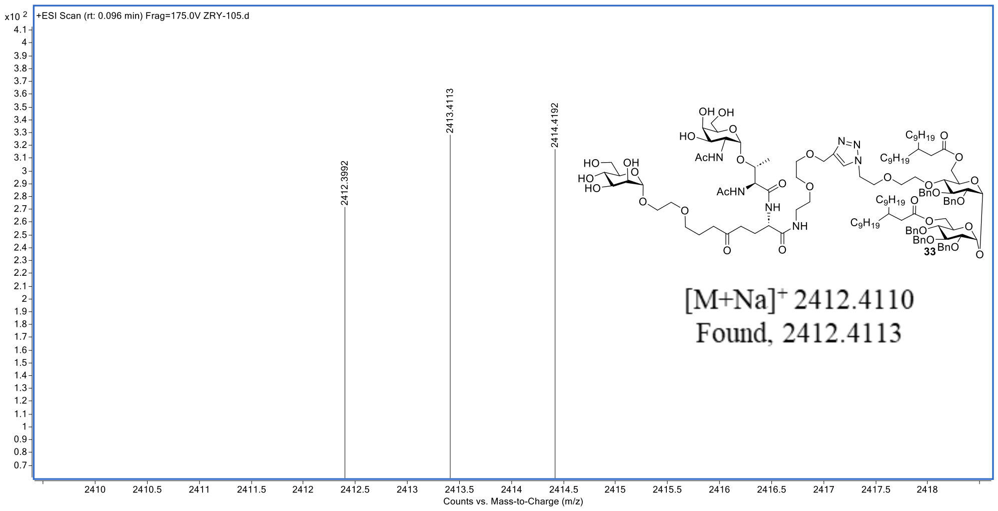

Figure S149 HRMS (ESI) spectrum of compound **34**

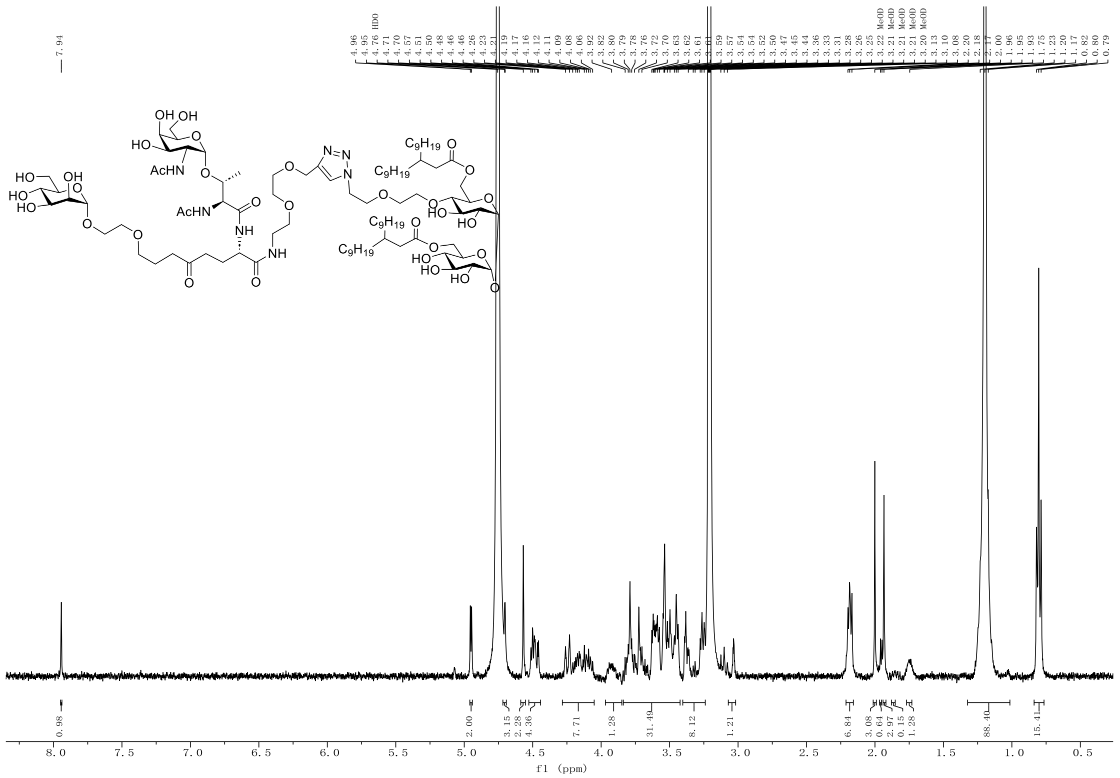

Figure S150. ^{1}H NMR (400 MHz, CD_3OD) spectrum of compound **MVT**.

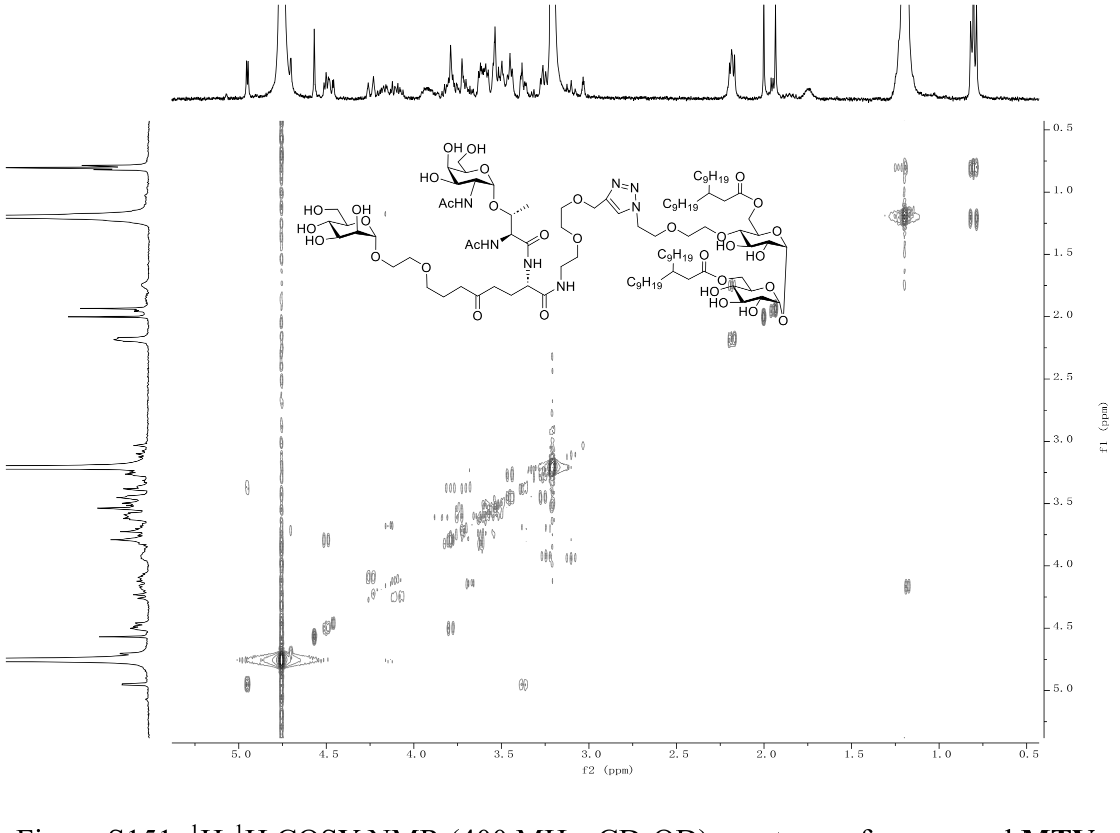

Figure S151. ^{1}H-^{1}H COSY NMR (400 MHz, CD_3OD) spectrum of compound **MTV**.

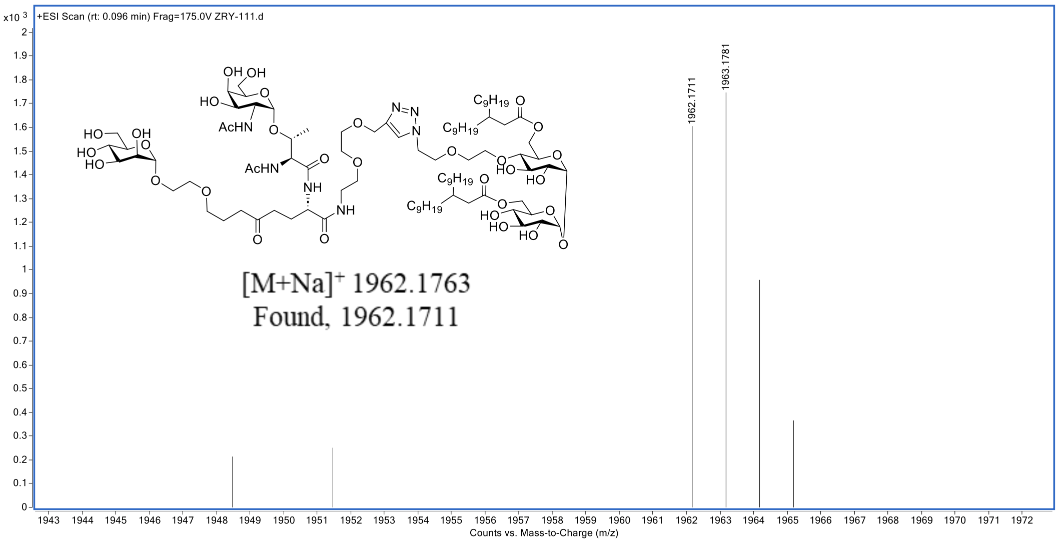

Figure S152. HRMS (ESI) spectrum of compound **MVT**.

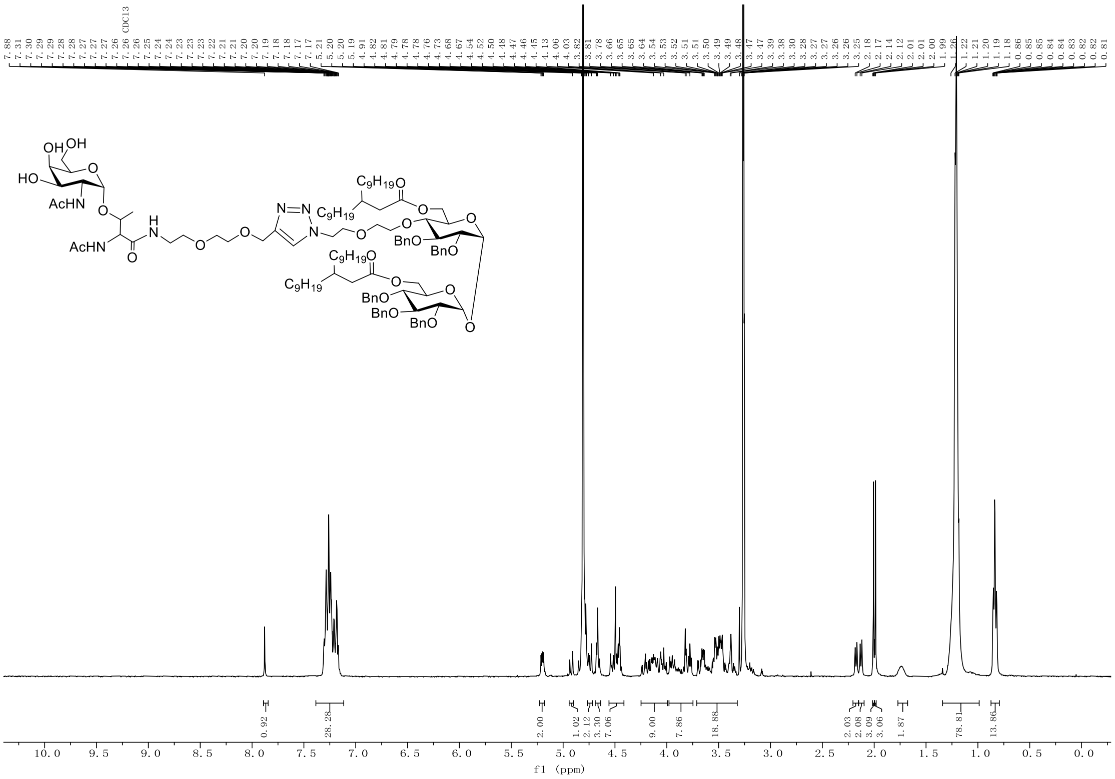

Figure S153. ^{1}H NMR (400 MHz, CD_3OD) spectrum of compound **37**.

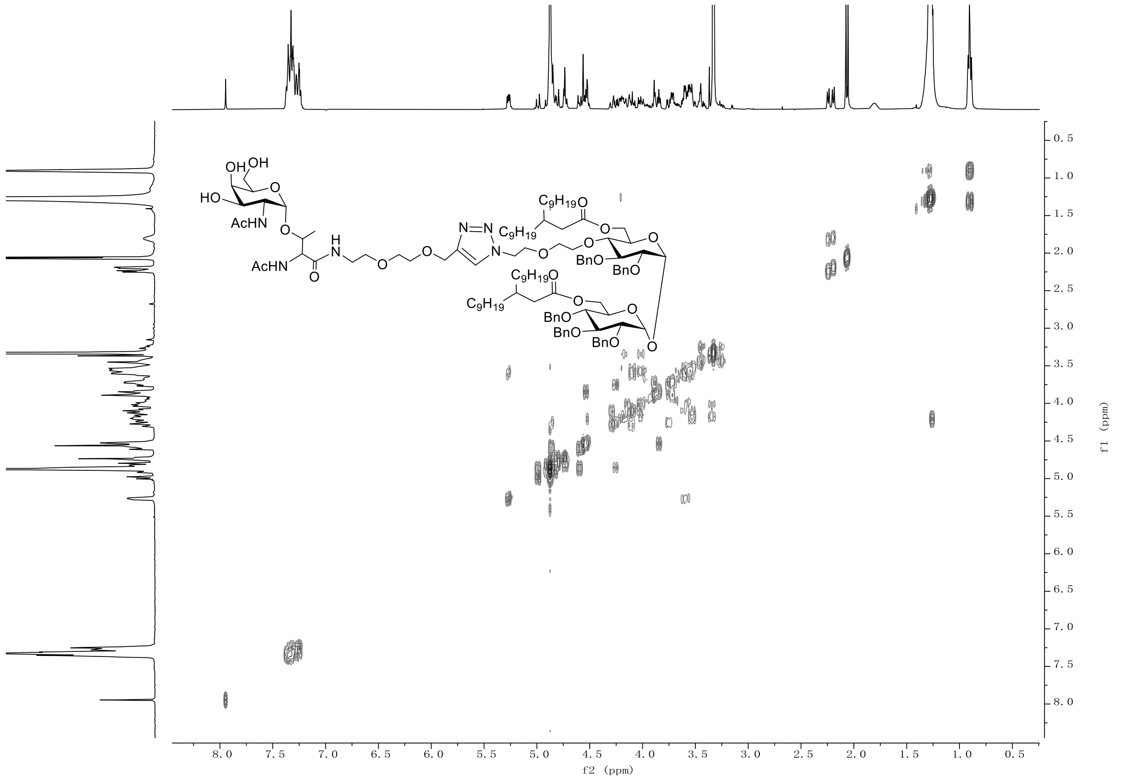

Figure S154. ^1H-^1H COSY NMR (400 MHz, CD_3OD) spectrum of compound **37**.

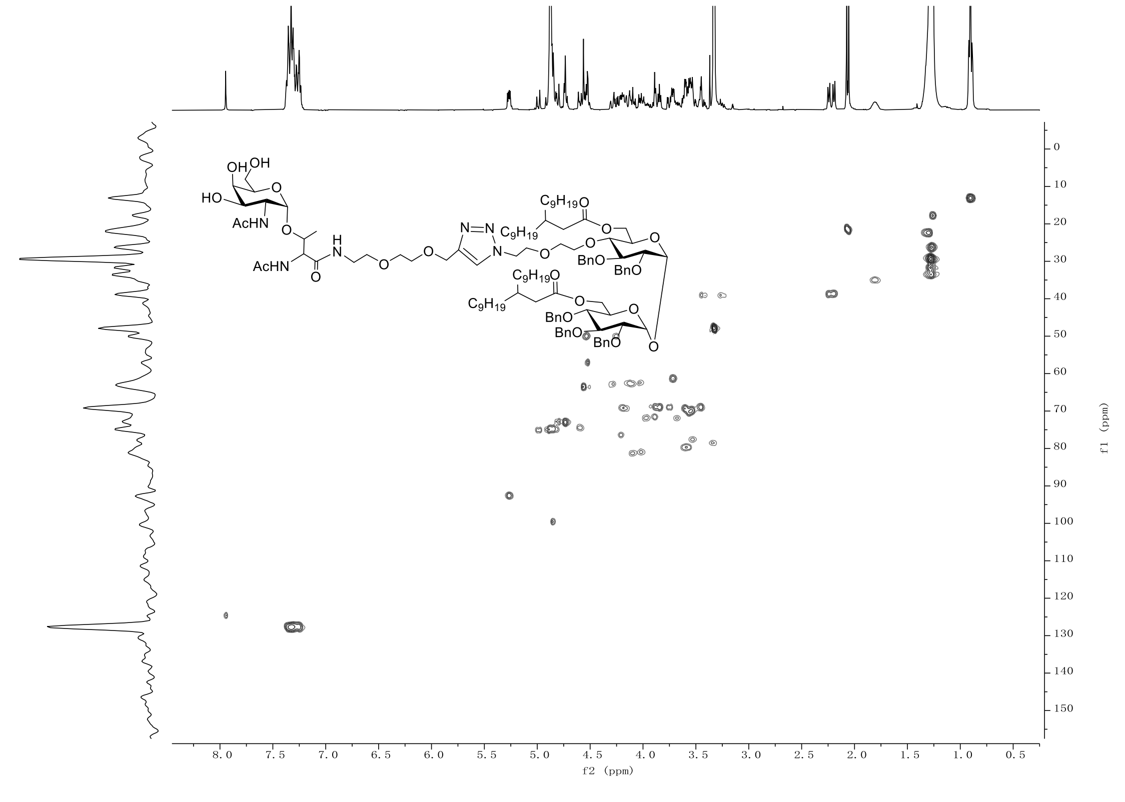

Figure S155. HSQC NMR (400/101 MHz, CD_3OD) spectrum of compound **37**.

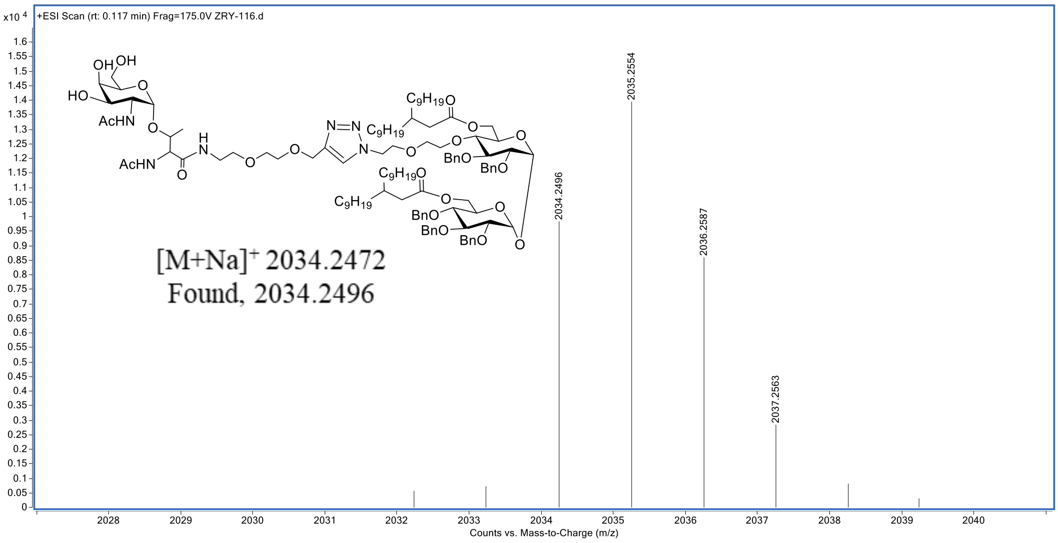

Figure S156. HRMS (ESI) spectrum of compound **37**.

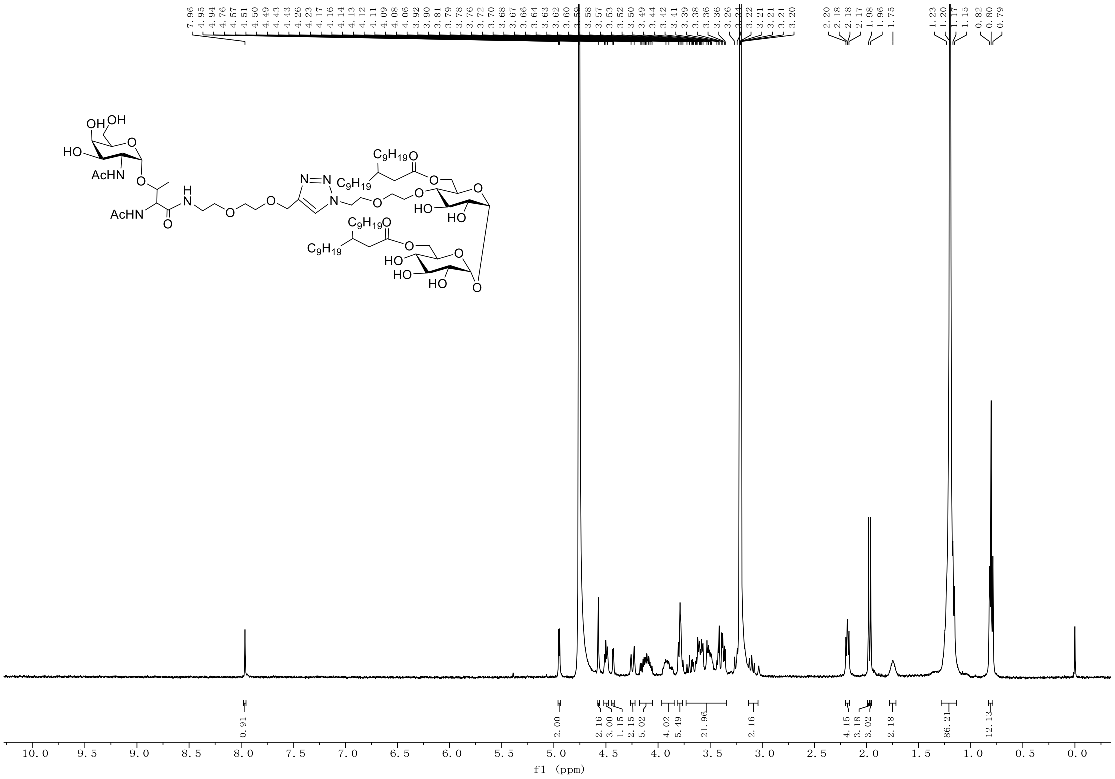

Figure S157. ^{1}H NMR (400 MHz, CD_3OD) spectrum of compound **VT**.

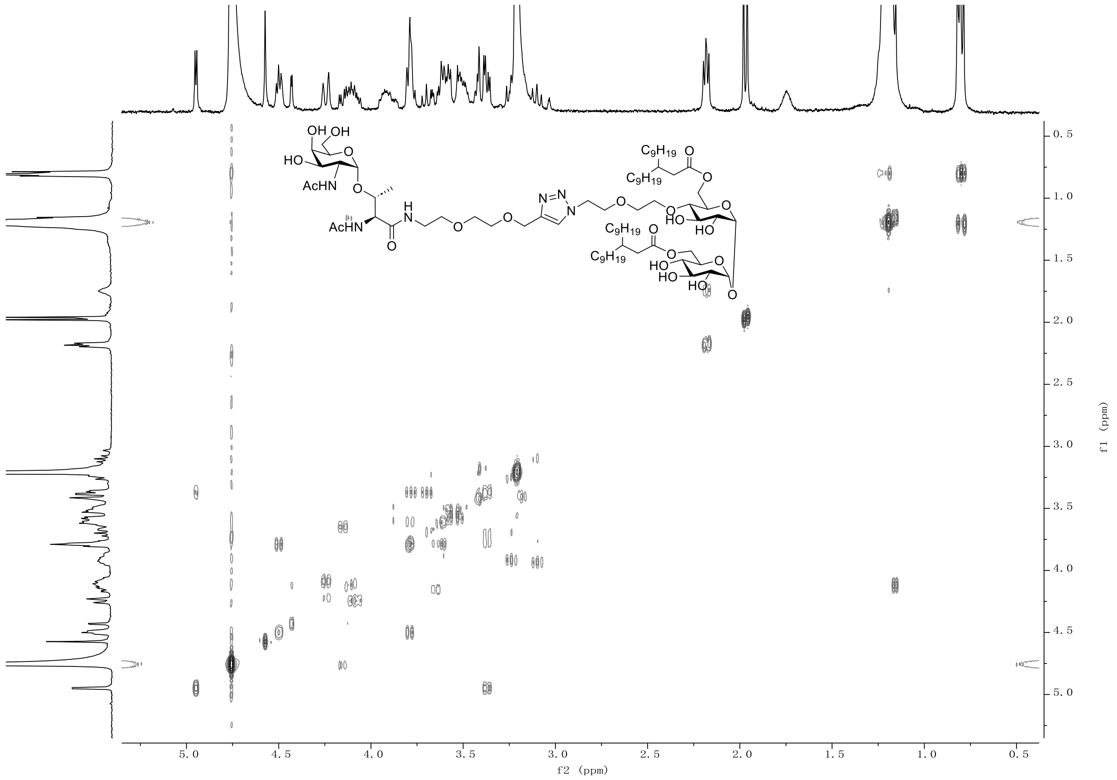

Figure S158. ^1H-^1H COSY NMR (400 MHz, CD_3OD) spectrum of compound **VT**.

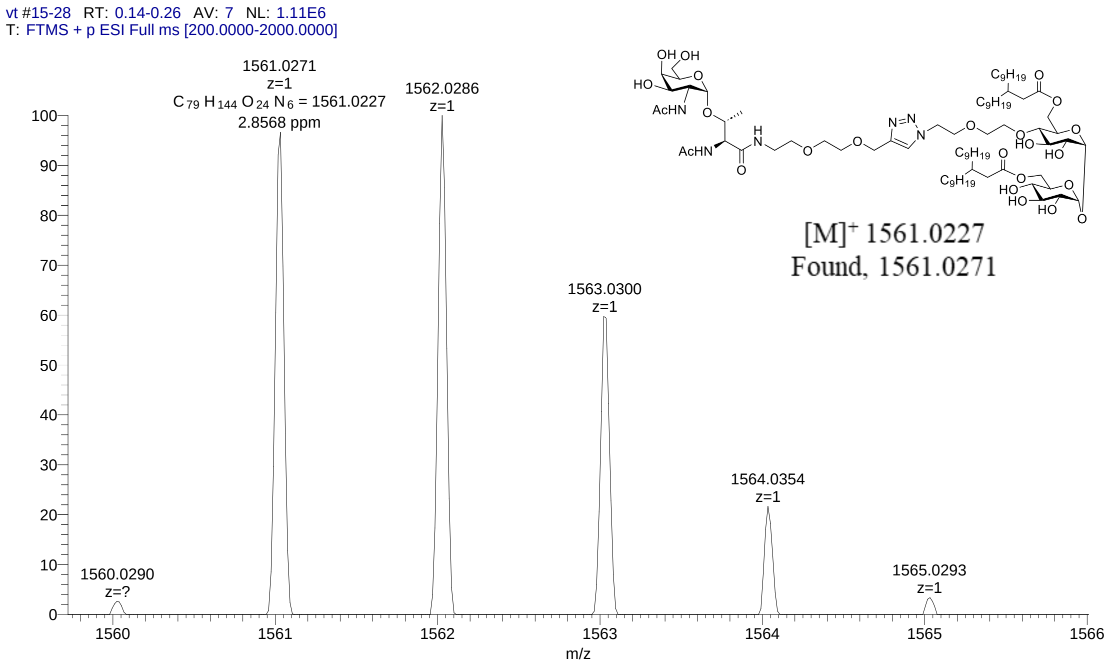

Figure S159. HRMS (ESI) spectrum of compound **VT**.

4. Reference

[1] D. Yang, X. Luo, Q. Lian, L. Gao, C. Wang, X. Qi, R. Zhang, Z. Liu, G. Liao, Fully synthetic Tn-based three-component cancer vaccine using covalently linked TLR4 ligand MPLA and iNKT cell agonist KRN-7000 as built-in adjuvant effectively protects mice from tumor development, *Acta Pharm. Sin. B* **2022**, *12*, 4432-4445.

[2] D. Yang, X. Li, J. Li, Z. Liu, T. Li, P. Liao, X. Luo, Z. Liu, W. Ming, G. Liao, Fully Synthetic TF-Based Self-Adjuvanting Vaccine Simultaneously Triggers iNKT Cells and Mincle and Protects Mice against Tumor Development, *J. Med. Chem.* **2024**, *67*, 17640-17656.
